# Pregnant and postpartum women’s experiences of the indirect impacts of the COVID-19 pandemic in high-income countries: a qualitative evidence synthesis

**DOI:** 10.1186/s12884-024-06439-6

**Published:** 2024-04-11

**Authors:** Annie Tan, Amanda Blair, Caroline SE. Homer, Robin Digby, Joshua P. Vogel, Tracey Bucknall

**Affiliations:** 1 School of Nursing and Midwifery, Deakin University, Geelong, Australia; 2 Maternal, Child and Adolescent Health Program, Burnet Institute, Melbourne, Australia; 3 Centre for Quality and Patient Safety Research, Institute of Health Transformation, Geelong, Australia; 4 Melbourne School of Population and Global Health, The University of Melbourne, Melbourne, Australia; 5 Alfred Health, Melbourne, Australia

**Keywords:** COVID-19 pandemic, Maternal and newborn health, Qualitative synthesis, Women’s experiences

## Abstract

**Background:**

Pregnant and postpartum women’s experiences of the COVID-19 pandemic, as well as the emotional and psychosocial impact of COVID-19 on perinatal health, has been well-documented across high-income countries. Increased anxiety and fear, isolation, as well as a disrupted pregnancy and postnatal period are widely described in many studies. The aim of this study was to explore, describe and synthesise studies that addressed the experiences of pregnant and postpartum women in high-income countries during the first two years of the pandemic.

**Methods:**

A qualitative evidence synthesis of studies relating to women’s experiences in high-income countries during the pandemic were included. Two reviewers extracted the data using a thematic synthesis approach and NVivo 20 software. The GRADE-CERQual (Confidence in the Evidence from Reviews of Qualitative research) was used to assess confidence in review findings.

**Results:**

Sixty-eight studies were eligible and subjected to a sampling framework to ensure data richness. In total, 36 sampled studies contributed to the development of themes, sub-themes and review findings. There were six over-arching themes: (1) dealing with public health restrictions; (2) navigating changing health policies; (3) adapting to alternative ways of receiving social support; (4) dealing with impacts on their own mental health; (5) managing the new and changing information; and (6) being resilient and optimistic. Seventeen review findings were developed under these themes with high to moderate confidence according to the GRADE-CERQual assessment.

**Conclusions:**

The findings from this synthesis offer different strategies for practice and policy makers to better support women, babies and their families in future emergency responses. These strategies include optimising care delivery, enhancing communication, and supporting social and mental wellbeing.

**Supplementary Information:**

The online version contains supplementary material available at 10.1186/s12884-024-06439-6.

## Background

As of February 2024 SARS-CoV-2 has infected over 774 million people, and 7 million deaths have been attributed to coronavirus 19 (COVID-19) infection [1]. Maternal and newborn health services are essential for pregnant and postpartum women, and the COVID-19 pandemic significantly altered provision and access to routine care. Reduced services, limited face-to-face care, transition to virtual and remote care, and limited access to maternity care providers were commonly cited as barriers to accessing quality care by pregnant and postpartum women [2–6]. Additionally, reduced lengths of stay within hospitals and restrictions on support people imposed by health facilities have impacted women receiving care and placed an additional burden on nursing and midwifery staff [7–9]. This had significant impacts on pregnant and postpartum women’s emotional and psychosocial wellbeing.

Pregnant women and their babies were at an increased risk of adverse effects if she contracted SARS-CoV-2 [10, 11]. The direct impacts of the COVID-19 pandemic were largely focused on the clinical manifestations of SARS-CoV-2 such as symptoms, risk factors, management and treatment, as well as adverse maternal and newborn outcomes [12–15]. However, at a wider level, the impacts of policy changes, health system reforms and changes to maternity care services indirectly affected the provision of care for all women giving birth over this time period. Women’s experiences of the transition from pregnancy to motherhood were also impacted. For example, in many countries, pregnant women were encouraged to homestay at home, receive care through telehealth rather than face-to-face and reduce face-to-face education [16, 17]. Isolation from family, friends and peers has negatively impacted women’s mental health, with increased levels of anxiety, depression and stress globally [18–21].

Since the beginning of the pandemic, there has been a plethora of qualitative studies on women’s experiences [19, 22–26] – the significant volume of papers highlights the need for a clear synthesis. Reviews of qualitative evidence have reported pregnant women’s experiences of social support [27], as well as highlighting the challenges they faced as they embraced motherhood during the pandemic [28]. Collating the evidence in a systematic and transparent manner will allow policymakers to consider the indirect implications of public health restrictions on the physical, emotional, and psychosocial health and wellbeing of pregnant and postpartum women.

Qualitative evidence synthesis (QES) is an approach that can systematically collate qualitative data in a transparent manner to inform policy and practice [29]. Findings from a QES can enable a richer interpretation of a particular phenomenon and enable a greater understanding of individual experiences, views and beliefs [30]. This QES aimed to explore, describe and synthesise the experiences of pregnant and postpartum women living in high-income countries during the first two years of the COVID-19 pandemic. This research method allows a deeper understanding of their views and experiences during this time. It also facilitates identification of areas of improvement for maternity care services, to ensure high-quality care is available at all times.

## Methods

A QES was undertaken to identify, evaluate and summarise findings from qualitative studies providing a cohesive and transparent documentation of the contextual variations, stakeholder preferences and experiences to ultimately influence policy and practice [31, 32]. This type of synthesis integrates diverse perspectives, which is needed to capture the complexity of the indirect impacts of the COVID-19 pandemic on pregnant and postpartum women’s experiences. This QES was structured to include findings from qualitative studies, as well as qualitative findings from mixed-methods studies. Emphasis was placed on including different types of qualitative evidence that can potentially enrich a synthesis, such as narrative data from qualitative components of mixed-methods studies or free-text from questionnaires [29].

We followed the relevant Cochrane guidelines [29] and used the “Enhancing transparency in reporting the synthesis of qualitative research” (ENTREQ) statement to guide our approach and reporting (Supplementary 1, S1) [33]. In addition, the Preferred Reporting Items for Systematic Reviews and Meta-Analyses (PRISMA) guidelines for reporting the different phases of identifying studies was used as recommended by the ENTREQ statement (S2) [34]. The protocol and systematic review were not registered.

### Eligibility criteria

We defined “indirect impacts of the pandemic on women”, to mean the impact of regulations, recommendations and public health measures enforced by governments as a response to the COVID-19 pandemic had on pregnant and postpartum women and their newborns. We adopted the World Health Organization’s definition of the postpartum period beginning immediately after birth of the baby and extending to six weeks (42 days) after birth [35].

Participants within these studies were those who were pregnant or within the postpartum period, of childbearing age (15-49 years), and received any type of maternity care during the COVID-19 pandemic. Studies of women with pre-existing comorbidities were also eligible, as well as those focused on migrants, refugee populations or ethnic minority groups. To facilitate exploration of findings from women of diverse backgrounds we have used the term ‘culturally and linguistically diverse (CALD) populations’. We focussed on women living in high-income countries (HICs). Studies were included if they were conducted in countries listed in the Organisation for Economic Co-operation and Development (OECD) [36] and Human Development Index (HDI) list of “Very high human development” list [37]. This allowed for similar contexts and countries to be compared.

Eligible study designs were those that addressed the indirect impact of COVID-19 using qualitative methodologies, including phenomenology, ethnography, grounded theory studies and case studies. We also included any study that obtained data through qualitative methods for data collection such as, interviews, focus groups, online forums and document analysis.

The decision to limit the eligibility based on year of publication, to only include studies published in the first two years of the COVID-19 pandemic (1^st^ Jan 2020 – 1^st^ Jan 2022) was to emphasise the impact of the stricter restrictions and lockdowns during this time period. Globally, public health measures to reduce spread and transmission included, mandatory quarantine, limiting movement, lockdowns, closure of schools and workplaces and shielding of vulnerable populations. These measures were significantly harsher during the first two years and subsequently relaxed as vaccine roll-outs occurred and infection rates began to decline [38, 39]. The Oxford Coronavirus Government Response Tracker reported a stringency index which reiterates the trend of harsher restrictions implemented by governments throughout 2020-2022 time period and reflects the gradual decline after this date [40].

### Search strategy

Six electronic databases (EBSCO Medline, Embase, APA PsycInfo, CINAHL and Maternity and Infant Care (MIDIRS)) were searched to identify all qualitative research articles published between 1^st^ January 2020 – 1^st^ January 2022. Search strategy included terms such as, “pregnan*”, “postpartum”, “mother”, “views”, “experiences”, “opinion*”, “indirect”, “COVID-19”, “coronavirus”. The search strategy was reviewed by a university librarian (S3). Search hits from each of the databases were imported into Endnote 20 which was then used as our reference library. References were imported into Covidence for screening [41].

### Study selection and sampling framework

**Fig. 1:**
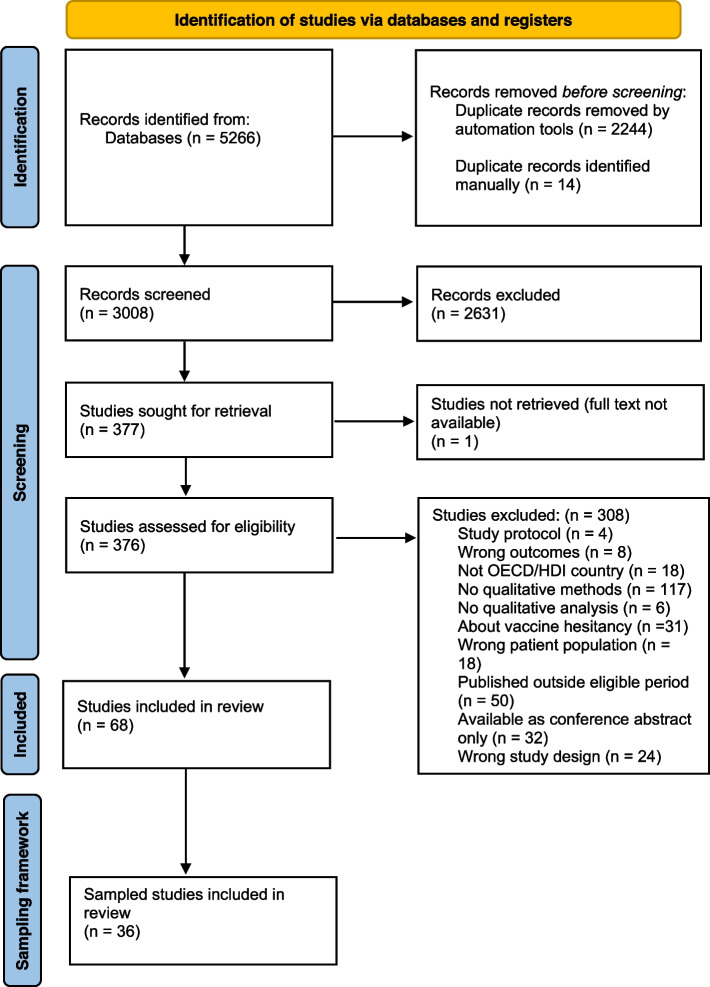
Reporting of adapted PRISMA flowchart of included and sampled studies in accordance with PRISMA and ENTREQ guidelines [33]

Two review authors (AT, AB) independently screened titles, abstracts and full texts for inclusion, with any conflicts resolved by discussion or consulting a third author. Reasons for exclusion are described within PRISMA flowchart (Fig. 1). Sixty-eight studies were included following full-text review. The Cochrane guidelines for QES highlight that for reviews with large amounts of primary studies (50 or more) can result in a high volume of data, which can threaten quality of the synthesis. In such situations, a sampling framework can enhance the quality and diversity of the papers and ensure the number of studies and amount of data are manageable [42–44]. A QES worked example by Ames et al., 2019 was used as a guide to develop the sampling framework for data richness [45]. Two independent reviewers scored included studies from 1 to 5 based on the criteria outlined in Table 1, to ensure that the sampling framework was reliable and replicable. Any conflicts were resolved by discussion, or a third review author was consulted. Studies with a score ≥4 were included for data extraction and are referred to as ‘sampled’ studies (S4).

**Table 1:** Sampling framework - using data richness as an indicator of quality, adapted from Ames et al. 2019 [45]

**Score**	**Measure**	**Example**
1	Very little qualitative data presented. The findings that are presented are fairly descriptive	For example, a mixed-methods study using open ended survey questions and generalised qualitative results with little analysis
2	Some qualitative data presented that relate to the synthesis objective	For example, a limited number of qualitative findings from a mixed-methods or qualitative study, often short descriptive analysis
3	A reasonable amount of qualitative data that relate to the synthesis objective	For example, a typical qualitative research article in a journal with a smaller word limit and often using simple thematic analysis, or content analysis
4	A good amount and depth of qualitative data that relate to the synthesis objective	For example, a qualitative research article in a journal with a larger word count that includes more context and setting descriptions and a more in-depth presentation of the findings, this may also include mixed-methods studies that have a significant qualitative component in addition to quantitative results
5	A large amount and depth of qualitative data that relate to the synthesis objective	For example, from a detailed ethnography or a qualitative research article with theoretical approach

### Quality assessment

The Critically Appraisal Skills Program (CASP) tool for qualitative research was used by two independent review authors (AT, AB) to assess methodological limitations of sampled studies (S5) [46]. Any disagreements were resolved through discussion, or when required, a third review author was consulted. Sampled studies were graded as no or very minor, minor, moderate or severe concerns with methodological limitations.

### Data extraction and synthesis

A “Characteristics of sampled studies” table was created in Excel and details are reported in Table 2. Two independent reviewers familiarised themselves with the sampled studies and extracted key themes using Braun and Clarke’s reflexive approach to inductive and deductive thematic analysis [47]. Data were managed using NVivo 20 [48]. This was an iterative process as many of the themes and sub-themes overlapped and were relevant in many aspects throughout the perinatal period (Table 3). The findings were developed iteratively, and periodically shared with the broader team to evolve our interpretation. Any quotes taken from studies were selected as they reiterated findings, and provided additional depth and meaning to review findings.

**Table 2:** Characteristics of sampled studies

**Ref #**	**Author/Year**	**Study design**	**Study aims/objectives**	**Participants**	**Data collection time frame and method**	**Data analysis**
**01**	Anderson et al. 2021 United Kingdom (UK) [16]	Mixed methods	**Aim:** This project aimed to explore pregnant women’s understanding of the behavioural restrictions and their perceived ability to comply, as well as the most concerning impacts of the measures	Pregnant women *N*=31 Primiparous *N*=20	24 Apr - 4 May 2020 Telephone or video call interview	Framework analysis
**02**	Atmuri et al. 2022 Australia [49]	Qualitative descriptive study	**Aim:** This original research aimed to study the perspectives of pregnant women in Australia in relation to the impact of the COVID-19 pandemic on their pregnancy experience.	Pregnant women *N*=15 Primigravida *N*=10	1 Jun - 19 Jun 2020 Telephone or video call interview	Thematic analysis - Braun and Clarke's
**03**	Aydin & Aktas 2021 Turkey [50]	Qualitative descriptive study	**Aim**: To investigate the pregnancy experiences of women during the COVID-19 pandemic from the perspectives of pregnant women using a qualitative research method and fill the gap in the relevant field.	Pregnant women *N*=14 Primiparous *N*=10	Dec 2020 Telephone interview	Thematic analysis - Braun and Clarke's; Lincoln and Guba's Evaluative Criteria
**04**	Brown & Shenker 2021 UK [51]	Mixed methods study	**Aim**: to understand how the COVID‐19 pandemic affected their (women with infants under the age of 1 year) infant feeding attitudes, choices and outcomes.	Mothers who had breastfed their baby (0-12 months) at least once *N*=1219 Primiparous (*N*=713)	May - Jun 2020 Survey data (open-ended questions)	Thematic analysis
**05**	Charvat et al. 2021 United States (USA) [52]	Qualitative study - CNSM theory	**RQ1**: What themes related to received social support emerge in women’s stories of their pregnancy during the COVID-19 pandemic? **RQ2**: How does social support contribute to the tone of women’s narratives about their pregnancy during the COVID-19 pandemic?	Pregnant women *N*=11 Primiparous (≈*N*=11)	N/A Telephone or video call interview	Thematic analysis - Tracy 2019 's two-step phronetic iterative approach
**06**	Costa et al. 2021 UK [53]	Qualitative study	**Aim**: to assess the impact of the Covid-19 pandemic on new parents of children born with CL/P in the UK prior to or during the initial ‘wave’ in the first half of 2020	Parents (mothers & fathers) *N*=14 Primiparous (*N*=10)	Jan – Jun 2020 Telephone or video call interview	Thematic analysis - Braun and Clarke
**07**	Davis et al. 2021 Australia [54]	Mixed methods study	**Aim**: to explore the relationship between emotional health and wellbeing and support needs of perinatal women during the COVID-19 pandemic, and to understand their experiences and need for support.	Perinatal women *N*=14	Nov 2020 – Feb 2021 Telephone or video call interview	Thematic analysis - Braun and Clarke
**08**	DeJoy et al. 2021 USA [55]	Qualitative study - interpretive phenomenological approach	**Aim**: to understand the lived experiences of pregnant people who switched their planned place of birth from hospital to community settings as a result of the COVID-19 pandemic.	Pregnant women *N*=17	May - Oct 2020 Telephone or video call interview	Theoretical framework - interpretive phenomenological analysis
**09**	Dove-Medows et al. 2022 USA [56]	Mixed methods study	**Aim**: to use a mixed-methods approach to explore the perspectives of a sample of Black women in the Midwestern United States to learn about their experiences of care in the prenatal, birth, and postpartum periods during the COVID-19 pandemic	Pregnant women (at least 20 weeks gestation) *N*=16	May - Jun 2020 Telephone or video call interview	Thematic analysis – Braun and Clarke
**10**	Farrell et al. 2021 USA [57]	Qualitative study	**Objective**: to examine the impact of COVID on patients' access and utilization of prenatal genetic screens and diagnostic tests at the onset of the COVID‐19 pandemic in the United States.	Pregnant women *N*=40 Primigravida (*N*=15)	May - Jul 2020 Telephone or video call interview	Iterative and progressive process consistent with Grounded Theory
**11**	Fumagalli et al. 2022 Italy [58]	Qualitative study - interpretive phenomenological approach	**Aim**: To explore childbearing experiences of COVID-19 positive mothers who gave birth in the months of March and April 2020 in a Northern Italy maternity hospital.	COVID-19 positive mothers (postpartum) *N*=22 Primiparous *N*=7	Mid Jun - End Jul 2020 Telephone or video call interview, face-to-face	Thematic analysis - an interpretive phenomenological approach
**12**	Green et al. 2022 Canada [59]	Qualitative study	**Objective**: 1) capture the principal worries reported by treatment-seeking pregnant and postpartum women with a principal AD to determine what proportion of their worries were specific to COVID, 2) identify and characterize the content of both non-COVID and COVID related worry, 3) identify the impact of COVID on the lives of pregnant and postpartum women	Pregnant women (treatment seeking patients) *N*=35 Postpartum women *N*=49	Apr – Oct 2020 Semi-structured diagnostic assessments to determine worries Duration unknown	Content analysis
**13**	Harrison et al. 2021 UK [60]	Cross sectional survey - mixed methods	**Aim**: to collect population-based data on women’s experiences of being pregnant and giving birth in England (2020 NMS) or in the UK (social media survey) during the first wave of the Covid-19 pandemic	Postpartum women *N*=4,611 (NMS survey) *N*=1,622 (Social media survey)	NMS survey: Nov - Mar 2020 Social media survey: 27 Nov 2020 - 26 Feb 2021 Online open-ended survey	Thematic analysis
**14**	Jackson et al. 2022 UK [61]	Qualitative study	**Aim**: to explore UK women’s postnatal experiences of social and healthcare professional support during the COVID-19 pandemic	Postpartum women *N*=24 Primiparous *N*=12	T1: April - 20 May 2020 T2: April - 16 July 2020 Telephone or video call interview	Recurrent cross-sectional thematic analysis
**15**	Jackson et al. 2021 UK [62]	Qualitative study	**Aim**: to contribute towards the existing literature-base, by exploring the postpartum psychological experiences of UK women during the COVID-19 pandemic	Postpartum women *N*=24 Primiparous *N*=12	T1: 22 Apr - 20 May 2020 T2: April - 16 Jul 2020 Telephone or video call interview	Modified recurrent cross-sectional thematic analysis
**16**	John et al. 2021 UK [63]	Qualitative study	**Aim**: to explore the experiences of pregnancy, childbirth, antenatal and postnatal care in all women belonging to ethnic minority communities and to identify any specific challenges that these women faced during the SARS-CoV-2 pandemic	Pregnant women *N*=9 Postpartum women *N*=7 Nulliparous(*N*=2)	Dec 2020 – Jan 2021 Telephone interview	Thematic analysis with qualitative interpretive approach
**17**	Joy et al. 2020 Canada [64]	Qualitative study - feminist poststructuralism	**Aim**: to examine parents’ experiences of the postpartum period during the mandated health protection orders in response to the COVID-19 pandemic	Postpartum women *N*=68	May - Jun 2020 Online open-ended survey	Discourse analysis
**18**	Keating et al. 2022 Ireland [65]	Qualitative study	**Aim**: to understand the lived experience of pregnancy during the pandemic and the effects on the women’s home life and social support system as well as learn from the effects that the hospital restrictions and measures had on women and their families.	Pregnant women *N*=8 Postpartum women *N*=6 Primiparous *N*=9	Apr - Jul 2020 Telephone or video call interview	Constant comparative method
**19**	Kolker et al. 2021 Canada [66]	Qualitative study	**Aim**: to explore pregnant and postpartum individuals’ lived experience during the COVID-19 pandemic to better understand their psychological and emotional responses and behaviours, with a focus on specific strategies to ameliorate distress	Postpartum women *N*=12 Primiparous *N*=8	Jul - Oct 2020 Telephone or video call interview	Thematic pattens and relations
**20**	Kynø et al. 2021 Norway [67]	Qualitative study	**Aim**: to explore parental (mothers and fathers or other caregivers) experiences for families hospitalized with an infant in the NICU at Oslo University Hospital during the absolute visitation ban period due to the COVID-19 crisis	Parents of infants in NICU/SCN *N*=9 Primiparous N=7	26 Jun 2020 - mid Jun 2021 Semi-structured interviews	Thematic analysis
**21**	Linden et al. 2022 Sweden [68]	Qualitative study - phenomenological reflective lifeworld approach	**Aim**: to gain a deeper understanding of how women not infected by SARS-CoV-2 experienced pregnancy during the COVID-19 pandemic in Sweden	Postpartum women *N*=14 Primiparous *N*=9	Mar - Apr 2021 Telephone or video call interview	Phenomenological reflective lifeworld approach – Dahlberg et al
**22**	Meaney et al. 2021 Ireland, UK, USA [9]	Cross sectional survey - mixed methods	Aim:to assess pregnant women’s satisfaction with antenatal care and social support in addition to examining the stress-reduction strategies employed by pregnant women during the COVID-19 pandemic	Pregnant women *N*=573 (558 open ended responses) Nulliparous *N*=241	16 Jun - 17 Jul 2020 Online survey with open-ended questions	Qualitative content analysis with an inductive approach
**23**	Mizrak Sahin & Kabakci 2021 Turkey [69]	Qualitative study	**Aim**: to understand the experiences of pregnant women during the COVID-19 pandemic	Pregnant women *N*=15 Primiparous *N*=12	Not stated Online semi-structured questionnaire with open-ended questions conducted by telephone	Content analysis - Graneheim and Lundman's
**24**	Ollivier et al. 2021 Canada [70]	Qualitative study - feminist post structuralism	**Aim**: to gain an in-depth understanding of parenting experiences in Nova Scotia during the first wave of the COVID-19 pandemic (March-June 2020)	Postpartum women *N*=68 Primiparous *N*=35	May - Jun 2020 Online survey with open-ended questions	Feminist poststructuralist discourse analysis
**25**	Panda et al. 2021 Ireland [71]	Qualitative study	**Aim**: to gain insight and understanding of women’s views and experiences of maternity care during the COVID-19 pandemic in Ireland	Postpartum women *N*=19 Primiparous *N*=8	15 Sep - 23 Oct 2020 Telephone or video call interview	Thematic analysis
**26**	Rhodes et al. 2020 UK [72]	Mixed methods study	**Aim**: to gain insights into the attitudes and experiences of expectant and recent parents (with babies under 24 weeks of age) during the COVID-19 pandemic; to investigate whether Baby Buddy is meeting users’ needs during this time; and to identify ways to revise the content of Baby Buddy to better support its users now and in future	Pregnant women *N*=14 Primiparous *N*=15	15 Apr - 31 May 2020 Telephone or video call interview	Thematic analysis
**27**	Rice & Williams 2021 Canada [73]	Qualitative study - social constructivist approach	**Aim**: to examine how people in Canada have been affected by policies aimed at limiting interpersonal contact to reduce SARS-CoV-2 transmission while giving birth in hospital and during the first weeks postpartum	Postpartum women *N*=57	Jun 2020 - Jul 2021 Telephone or video call interview	Thematic analysis using a social constructionist research paradigm
**28**	Rice & Williams 2022 Canada [74]	Qualitative study	**Aim**: To examine the impact of pandemic policy changes on experiences of pregnancy and birth, thereby identifying barriers to good care; to inform understandings of medicalization, care, pregnancy, and subjectivity during times of crisis; and to critically examine the assumptions about pregnancy and birth that are sustained and produced through policy.	Pregnant women *N*=8 Postpartum women *N*=59 Primiparous (*N*=32)	March 2020 - January 2021 Telephone or video call interview	Thematic analysis using a social constructionist standpoint
**29**	Riley et al. 2021 UK [75]	Qualitative study	**Aim**: to understand the impact of COVID-19 restrictions on women’s pregnancy and postpartum experience.	Pregnant women *N*=5 Postpartum women *N*=20 Primiparous *N*=15	Jul – Aug 2020 Telephone or video call interview	Thematic analysis - phenomenological focus
**30**	Saleh et al. 2022 USA [76]	Mixed methods study	**Aim**: to explore women’s experiences of being pregnant, giving birth, and parenting during the COVID-19 pandemic, in the United States, specific to the year 2020	Postpartum women *N*=32	Jan – Dec 2021 Telephone or video call interview	Thematic analysis - Hamilton’s rapid appraisal technique
**31**	Silverio et al. 2021 UK [77]	Qualitative study	**Aim**: to explore the experiences of women in South London, UK, receiving antenatal care both before and during the pandemic, and who subsequently gave birth and received postnatal care during the pandemic	Postpartum women *N*=23 Primiparous *N*=13	Mar - Aug 2020 Telephone or video call interview	Template analysis
**32**	Snyder & Worlton 2021 USA [78]	Qualitative study -cross-sectional phenomenological study	**Aim**: to explore perceptions of social support among breastfeeding mothers during the COVID-19 pandemic	Postpartum women *N*=29 Primiparous *N*=10	Mar – Jun 2020 Telephone or video call interview	Analysis of immersion and crystallisation, and then a thematic comparison of the two groups
**33**	Spatz & Froh 2021 USA [79]	Case series report	**Aim**: to better understand the ways in which new families experience pregnancy and lactation during the COVID-19 pandemic and the implications for maternal – child nurses and other health care providers, we share the experiences of three healthy first-time mothers	Postpartum women *N*=3 Primiparous *N*=3	Mar 2020 Telephone or video call interview	Thematic analysis – Braun and Clarke
**34**	Stirling Cameron et al. 2021 Canada [80]	Qualitative study - constructivist grounded theory	**Aim**: to present data on the postnatal experiences of resettled Syrian refugee women in the context of COVID-19. More specifically, to elucidate refugee women’s experiences accessing postnatal formal health services and informal social supports during the first six months of the COVID-19 pandemic	Postpartum women *N*=8	Aug - Sep 2020 Telephone or video call interview	Grounded theory - Charmaz, Corbin and Strauss, and constant comparative analysis
**35**	Sweet et al. 2021 Australia [81]	Qualitative study	**Aim**: to describe childbearing women’s experiences of becoming a mother during the COVID-19 pandemic in Australia.	Pregnant women *N*=9 Postpartum women N=18 Parity 0 (*N*=2) Parity 1 (*N*=3)	Jun 2020 Telephone or video call interview	Thematic analysis – Braun and Clarke
**36**	Sweet et al. 2022 Australia [82]	Qualitative study	**Aim**: to explore and describe childbearing women’s experiences of receiving maternity care during the COVID-19 pandemic in Australia	Pregnant women *N*=9 Postpartum women *N*=18 Parity 0 (*N*=2) Parity 1 (*N*=3)	Mar - Jun 2020 Telephone or video call interview	Thematic analysis

**Table 3:** Overarching themes, sub-themes and coding tree

**Theme**	**Sub-theme**	**Accessibility codes**	**Barrier codes**	**Illustrative quotes**
Dealing with public health restrictions	1.1. Limited support networks from health care system and providers		- Lack of support - Missing out on experiences	“When we went into lockdown I couldn't get hold of the midwife, all my appointments and training was cancelled, the hospital didn't have any information, and everybody was panicking. It took about 4 weeks for information to get to me. I didn't see my midwife from week 24 and had different ones every time because of COVID.” (Harrison 2021) [60]
	1.2. Balancing exposure risk and need for healthy behaviours	- Wanting to reduce risk - Acknowledging restrictions and its influence on health decision-making - Needing to plan ahead	- Delay in healthcare seeking	“I was very anxious leading up to the birth. Just because everything was changing, you didn’t know what was happening, the hospital policies kept changing. At one point the hospital I was delivering at said your partner could only stay for two hours after the delivery. That made me really upset and I didn’t know if I could end up having a c-section or what would happen.” (Rice 2022) [74] “I had a slight bleeding at first, I didn't know what to do, should I go or not? If I go, there is a risk of getting a virus … I postponed going to the doctor as much as possible.” (Aydin &Aktas 2021) [50]
	1.3. Missing out on social opportunities		- Missing out on traditions that usually occur during pregnancy, e.g. baby showers - Missing interactions with family and friends	“I think I just feel like we’re not celebrating as much being pregnant. I know that sounds kind of funny, but it almost feels like I’m not pregnant because, I don’t know, not many people are seeing me.” (Charvat 2021) [52]
	1.4. Breastfeeding challenges and triumphs	- Undisrupted time to establish breastfeeding	- Difficulties in establishing breastfeeding with limited healthcare provider support - Stopping breastfeeding early	“I was able to breastfeed without feeling really exposed and thinking about it now I think if there had been people around, strangers marching in and out of the ward I would have had the curtains pulled all the time.” (Panda 2021) [71] “We are unable to have face‐to‐face support to help me to feed my baby who is struggling to gain weight due to possible tongue tie which is unable to be treated. Because of the pressure to have him gain weight or be admitted to hospital and having little support with improving his latch and expressing milk I have had to top up with formula which is something I have not wanted to do and did not need to do with my first child.” (Brown & Shenker 2021) [51]
Navigating changing health policies	2.1. A birthing experience filled with uncertainty and unknowns		- Limited knowledge during the pandemic - Loss of autonomy - Uncertainties about early discharge - Attempting, but often failing to plan ahead	“It was really abrupt… they said ‘your choices are now limited. You are choosing between not the scenarios you wanted, but this is where we are, and you need to get it together’” (Silverio 2021) [77]
	2.2. Reduced support and partner presence in healthcare settings		- Partners not present during antenatal appointments - Partners not being about to support mothers post-birth	“How did it come to that it was allowed for fathers to be with the mothers but not their infants? It is tough to have a premature infant, and being the only parent with access, or being the one who is not allowed to have a father who feels that he is not a father [crying]. It was really tough! All the questions and things he was wondering. Such practical things with the infant’s development, but also how to know the infant; feeling safe and not feeling scared every time he visited the infant… We were never allowed to be there together…” (Kyno 2021) [67]
	2.3. Transitioning to telehealth, virtual and remote care	- Being able to access telehealth	- Receiving remote care post-birth - Questioning the quality of care	“There have been many improvements with digital meetings, which meant that you didn’t have to take time off work. So, I think it has been a much more positive experience, easy accessibility” (Linden 2022) [68] “I never had a six week postpartum appointment … I have not had a doctor look at me since I left the hospital … you’re on a virtual call and you can’t really explain things, you’re taking pictures that aren’t clear, it’s really, it’s not helpful.” (Kolker 2021) [66]
	Barriers to accessing health services		- Limited access to non-essential healthcare providers, e.g. physiotherapists, chiropractors, gym and pools - Delayed or reduced healthcare provision - Limited information about where to get support	“My two-week follow-up with the OB was over the phone. They would not see me in-person, which I was really upset about because I would rather have a doctor see if everything is healing properly.” (Rice 2022) [74] “Everyone is stressed, and all the services are stretched, you just don’t want to feel like you want to not waste their time, but normally you would be able to go to someone or go to a service like your midwife and ask them and they would have time to speak with you […] but because they are so stretched now you just don’t feel like you want to…” (Anderson 2021) [16]
Adapting to alternative ways of receiving social support	3.1. Accessing support through different avenues	- Using social media to connect with others - Having remote emotional support from family and friends - Valuing increased partner presence		“Thanks to lockdown my partner is here 24-7… even though he is working… we actually had the beauty of sharing both of our love with the baby, so if I am tired I can take a nap and he can take care of the baby for half-an-hour” (Riley 2021) [75]
	3.2. Desiring connection with family and friends		- Needing practical support - Inability to interact with family and friends physically	“It was really rough for the first few weeks – my parents live a few hours away so they didn’t meet him until he was 6 weeks old, and the plan had originally been that mum would come up and support us, obviously that didn’t happen” (Sweet 2021) [81]
Impacts on own mental health	4.1. Managing anxiety due to virus-related fears and concerns		- Emotionally dealing with the unknown impact on mother and baby - Negative impact on mental health - Being anxious about accessing hospitals	“I want to do what’s best for me. But, at the same time, my anxiety about being in a doctor's office or being in a space where I know there are potentially sick people nearby… I didn’t want to be there” (Farrell 2021) [57]
	4.2. Feeling lonely and isolated		- Increased levels of depression - Being alone and away from friends and family - Feeling abandoned by the public health system	“You haven’t got any friends or family that can necessarily come into your home and support you in case, they, you know, also contract or are carrying it. So, erm. Yeah. It is isolation” (Jackson 2022) [61]
Managing the new and changing information	5.1. Constantly changing advice and information	- Adapting to the constantly changing rules and public health restrictions	- Drowning under the overload of information	“The rules kept changing kind of minute by minute as they got more information, and it was really unsettling” (Sweet 2022) [82] “I’m an information seeker for sure but I found, with COVID, it was just almost too much information” (Kolker 2021) [66]
	5.2. Inadequate information from healthcare providers		- Lack of information caused anxiety and worry - Felt poorly communicated with and to - Dealing with contradictory information	“When we went into lockdown I couldn't get hold of the midwife, all my appointments and training was cancelled, the hospital didn't have any information, and everybody was panicking. It took about 4 weeks for information to get to me” (Harrison 2021) [60] “Contradicting statements saying yes it can get to your baby through the womb and no it can’t, and so there was not much information that was 100% certain” (Sweet 2021) [81]
Being resilient and optimistic	6.1. Self-help strategies to overcome challenges of the pandemic	- Being more conscious and taking control of their mental health - Being an advocate for yourself and your baby - Weighing up the risks of breaking public health restrictions - Strengthening coping strategies		“You have to be your own best advocate because nobody is really advocating for you which sounds really depressing – and just sort of be mindful of the fact that the system is overstretched so … don’t be afraid to be annoying” (Kolker 2021) [66] “I think that’s what a lot of mums are doing behind closed doors, sort of weighing up… you’ve gotta weigh up risks, a lot of people at this point now. Some people have moved in with their mums...” (Jackson 2022) [61]
	6.2. Making the most out of the positive encounters	- Spending more time to bond as a family unit - Valuing fewer disruptions to new motherhood - Enjoying the peaceful health services		“It’s been great… we have this opportunity to bond as a family and he [partner] is here for every moment during the newborn stage! It has been amazing not having to worry about visitors coming and going and cleaning out home and me worrying about breastfeeding in front of others - instead we have a very relaxed atmosphere for everything!” (Joy 2020) [64]
	6.3. Information seeking and desire for more information	- Utilising online and public resources	- Wanting more information	“You do feel a little bit stressed in there, and I think probably one thing that maybe could be improved is just that extra information of what you are doing with the COVID stuff in terms of precautions, what it’s going to look like when I come in to have bubs, just what to expect” (Atmuri 2022) [49] “It was pages of reading, there’s no videos, no nothing, and then you have all these questions, and you try to call to find out some answers and no one can give you any answers and there was no one to talk to” (Davis 2021) [54]

Extracted data were populated into two tables for analysis. The first table collated quotes and author interpretations of findings (S6), whilst the second table summarised these into review findings (Table 4).

**Table 4:** Summary of qualitative review findings table including GRADE-CERQual assessment of confidence

**Overarching theme**	**Sub-theme**	**Summarised review finding**	**GRADE-CERQual Assessment of confidence**	**Explanation of GRADE-CERQual Assessment**	**Reference ID #**
1. Dealing with public health restrictions	1.1. Limited support networks from health care system and providers	Social distancing meant that women could not have frequent face-to-face interactions with healthcare providers. Women expressed feelings of neglect, being forgotten and missing out on opportunities to share experiences with other people in similar situations.	High confidence	Minor concerns regarding methodological limitations (reflexivity, aim and research design, data saturation, data analysis, new areas of research), No/Very minor concerns regarding coherence, No/Very minor concerns regarding adequacy, and Minor concerns regarding relevance (some studies did not clearly indicate age of babies or studied specific sub-populations of women).	[1, 2, 4, 7, 9, 12–18, 21–26, 28, 32, 33, 36]
	1.2. Balancing exposure risk and need for healthy behaviours	Stressors of the pandemic evoked reactive health behaviours such as shielding in their homes (staying at home), limiting movement outdoors and avoiding healthcare settings. Heightened anxiety and changing health restrictions especially around labour and childbirth times influenced women’s decision making.	High confidence	Minor concerns regarding methodological limitations (reflexivity, recruitment, data saturation, data analysis and new areas of research), No/Very minor concerns regarding coherence, No/Very minor concerns regarding adequacy, and Minor concerns regarding relevance (some studies did not clearly indicate age of babies or studied specific sub-populations of women).	[1, 3, 9–11, 13–16, 18, 21, 23, 25, 31, 33–36]
	1.3. Missing out on social opportunities	Women mourned the loss of rituals and traditions during their pregnancy and postnatal period, missing out on the ability to celebrate new life with family and friends. Women also mourned the missed opportunity to meet other mothers and babies, missing out on social interactions that would have been usual pre-pandemic.	High confidence	Minor concerns regarding methodological limitations (aim, data saturation, reflexivity, analysis, in-depth discussion, statement for new areas of research), No/Very minor concerns regarding coherence, No/Very minor concerns regarding adequacy, and Minor concerns regarding relevance (some studies did not clearly indicate age of babies or studied specific sub-populations of women).	[4, 5, 7, 9, 12, 14, 17–19, 22–26, 29, 31–36]
	1.4. Breastfeeding challenges and triumphs	Breastfeeding infants during the pandemic had its own set of challenges and triumphs. For women who were struggling to establish breastfeeding and needed additional support, this was limited due to limited face-to-face consultations. Women able to establish a breastfeeding routine described the lockdown and public health measures as a blessing in disguise as they had more undisrupted time to bond with their babies and were able to feed on demand.	High confidence	No/Very minor concerns regarding methodological limitations, No/Very minor concerns regarding coherence, No/Very minor concerns regarding adequacy, and No/Very minor concerns regarding relevance.	[4–6, 13, 15, 17, 19, 24–27, 29, 31–33, 36]
2. Navigating changing health policies	2.1. A birthing experience filled with uncertainty and unknowns	Limited knowledge surrounding the impacts of the virus affected how women accessed healthcare and the trust they had in healthcare providers. Limited education, uncertainty about plans, choice of pain management and support people during labour and visitors on the postnatal ward caused stress and anxiety. Women were unable to plan ahead or maintain control over their labour and childbirth.	High confidence	Minor concerns regarding methodological limitations (design, data saturation, reflexivity and new areas of research), No/Very minor concerns regarding coherence, No/Very minor concerns regarding adequacy, and No/Very minor concerns regarding relevance.	[2, 5, 8, 11, 13, 19, 22, 28, 29, 31, 35, 36]
	2.2. Reduced support and partner presence in healthcare settings	The policies implemented limiting support people and partners throughout the perinatal period were described by women as unfair, anxiety and stress provoking, and traumatic. Women expressed their sadness and grief in partners missing milestones throughout pregnancy, as well as missing their advocators and support in labour and childbirth. On the postnatal ward, midwifery support was available, described as both lovely for mothers who coped well, and lacking compassion for mothers who felt minimally supported. Available support was appreciated but it was not the same as having their partners.	High confidence	No/Very minor concerns regarding methodological limitations, Minor concerns regarding coherence (some contributing studies included the additional impacts of lack of support from partners and barriers to receiving additional support), No/Very minor concerns regarding adequacy, and Minor concerns regarding relevance (some studied specific sub-populations of women and some focused on different levels of support).	[1, 2, 7, 9–16, 18–22, 25–31, 34–36]
	2.3. Transitioning to telehealth, virtual and remote care	Telehealth, virtual and remote care increased accessibility and allowed women the option of receiving care without the burden of attending hospitals and healthcare settings. It was accepted as “better than nothing”, however many women considered it “useless” and “inappropriate” for appointments to check pregnancy milestones, assess breastfeeding and inadequate when women and newborns needed to be checked physically. Many questioned the quality of care provided, found it challenging to navigate and lacked the support and reassurance from healthcare providers.	Moderate confidence	No/Very minor concerns regarding methodological limitations, Minor concerns regarding coherence (some studies included positive experiences of telehealth), Minor concerns regarding adequacy (some contributing studies provided thin data), and Minor concerns regarding relevance (some studied specific sub-populations of women and some focused on different levels of support).	[1, 2, 6–8, 13, 14, 16, 18, 19, 25, 26, 28, 31–34, 36]
	2.4. Barriers to accessing health services	Closure of non-essential services and educational classes, as well as constantly changing healthcare policies reduced the support women received. Healthcare providers were hard to reach, would often delay, postpone or cancel appointments. Post-birth, women were not provided with enough information about how and where to access support if required and felt that the onus was placed on themselves to stay informed. The biggest concern for new mothers was whether or not their newborns were thriving given the lack of check-ups.	High confidence	Minor concerns regarding methodological limitations (unclear research design, data analysis, reflexivity statement, lacked an in-depth discussion and did not provide statement for new areas of research), No/Very minor concerns regarding coherence, No/Very minor concerns regarding adequacy, and Minor concerns regarding relevance (some studies did not clearly indicate age of babies).	[1, 3, 6–8, 13–16, 19, 24–26, 28, 29, 31, 32, 34, 36]
3. Adapting to alternative ways of receiving social support	3.1. Accessing support through different avenues	Virtual technologies were used to access remote emotional support from family and friends, and social media platforms were used to access peer support. For some women, they had increased support from partners as they were at home more. Although supports were readily available remotely and online, almost all women reported that, “it was not the same” as being able to be physically present with people.	Moderate confidence	Minor concerns regarding methodological limitations (aim, data saturation, data analysis, reflexivity, discussion and lacking statement for new areas of research), Minor concerns regarding coherence (some findings were extrapolated from sub-population groups), No/Very minor concerns regarding adequacy, and Minor concerns regarding relevance (some studies did not clearly indicate age of babies or studied specific sub-populations of women).	[1, 3–7, 11, 14, 15, 17, 19–21, 24–26, 29–31, 35]
	3.2. Desiring connection with family and friends	Women felt sadness and heartbreak that their family and friends were not able to be physically present during their pregnancy and early postnatal period. They desired and needed practical support from their families and friends, for their mental health, physical wellbeing and as carers for older children. Postnatal women felt they were an afterthought in the government’s plan to return to a COVID-safe society.	High confidence	No/Very minor concerns regarding methodological limitations, No/Very minor concerns regarding coherence, No/Very minor concerns regarding adequacy, and Minor concerns regarding relevance (some studies did not clearly indicate age of babies or studied specific sub-populations of women).	[1, 2, 4, 6, 9, 12–15, 17, 19, 21–24, 26, 27, 29, 33–35]
4. Dealing with impacts on own mental health	4.1. Managing anxiety due to virus-related fears and concerns	Limited information about the adverse effects of COVID-19 infection on pregnant women, the unborn fetus and newborns caused heightened levels of anxiety, stress, fear and worry. The mental health burden increased as there was constant concern about the risk of infection and transmission. Attending hospitals or healthcare settings for check-ups were considered high-risk and dangerous settings.	Moderate confidence	Minor concerns regarding methodological limitations (study design, recruitment, data analysis, reflexivity, in-depth discussion and lacking statement for new areas of research), No/Very minor concerns regarding coherence, No/Very minor concerns regarding adequacy, and Minor concerns regarding relevance (some studies did not clearly indicate age of babies or studied specific sub-populations of women).	[1–3, 7, 9–12, 15, 16, 18, 19, 21–28, 31, 33–36]
	4.2. Feeling lonely and isolated	Social isolation from family, friends, peers and society placed a burden on the mental health of pregnant and postpartum women. The increased sense of loneliness and isolation, and the inability to see or meet with family and friends was believed to increase depression in women. The inability to receive adequate supports from care providers, further exacerbated the isolation women felt and had an impact on their physical wellbeing. Some described this experience as being forgotten or abandoned by the health care system.	High confidence	Minor concerns regarding methodological limitations (research design, recruitment, data saturation, reflexivity, data analysis, in-depth discussion and lacking statement for new areas of research), No/Very minor concerns regarding coherence, No/Very minor concerns regarding adequacy, and Minor concerns regarding relevance (some studies did not clearly indicate age of babies or focused on different levels of support and behaviours).	[1–3, 7–9, 12–16, 18, 21, 22, 24–27, 29, 30, 32–36]
5. Managing the new and changing information	5.1. Constantly changing advice and information	Managing conflicting information during the pandemic was challenging. The constant change to public health restrictions and hospital protocols and policies meant that definitive answers for labour and childbirth could not be given to women as they changed frequently. Women received non-specific information from hospitals and healthcare providers and felt they were not well informed, yet at the same time were overwhelmed by the information from governments, hospitals with their updated protocols and media reporting COVID-19 related news.	High confidence	No/Very minor concerns regarding methodological limitations, No/Very minor concerns regarding coherence, No/Very minor concerns regarding adequacy, and No/Very minor concerns regarding relevance	[5, 6, 8, 11, 13, 19, 20, 22–24, 29–31, 35, 36]
	5.2. Inadequate information from healthcare providers	Limited information about the adverse effects of infection combined with poor communication of these unknowns placed a greater distrust in maternity care providers and impacted women’s ability to make informed decisions. Women had to navigate contradictory information within policies and protocols, with discrepancies in information provided at the community level, to what was being enforced in hospital settings. Women would have preferred if uncertainties were better communicated, information was consistent and clear for the lay person	Moderate confidence	No/Very minor concerns regarding methodological limitations, Minor concerns regarding coherence (experiences of navigating information and communication from healthcare providers varied), No/Very minor concerns regarding adequacy, and Minor concerns regarding relevance (some studies did not clearly indicate age of babies, studied specific sub-populations of women or focused on different responses to the pandemic).	[1, 2, 5–7, 11, 13, 14, 16, 19–24, 26, 29–31, 33–36]
7. Being resilient and optimistic	6.1. Self-help strategies to overcome challenges of the pandemic	Women described having resilience and optimism in their reactions to public health measures and challenges during the pandemic. Women highlighted the need be conscious of and take care of their mental health and made active decisions to protect this. Women commented on their need to be advocates, encouraging other women to speak up to ensure they were receiving the best care for themselves and their newborns. Different coping strategies were used to ensure women preserved their mental and physical wellbeing.	High confidence	Minor concerns regarding methodological limitations (recruitment, data saturation, reflexivity, data analysis, in-depth discussion and lacking statement for new areas of research), No/Very minor concerns regarding coherence, No/Very minor concerns regarding adequacy, and Minor concerns regarding relevance (some studies did not clearly indicate age of babies, studied specific sub-populations of women or focused on different support levels and behavioural responses).	[1–3, 5, 7, 11, 12, 15, 18–21, 23–27, 31, 33, 35, 36]
	6.2. Making the most out of the positive encounters	Despite the challenges of the pandemic, women found silver linings throughout the perinatal period. Adherence to public health restrictions meant women had fewer social obligations allowing them to settle into their role as a mother and establish routines with their newborn. Fewer disruptions allowed families time to bond with the newborn and establish successful breastfeeding. For some, health services were described as a peaceful place to be.	Moderate confidence	Minor concerns regarding methodological limitations (aim, recruitment, data saturation, reflexivity, in-depth discussion and lacking statement for new areas of research), Minor concerns regarding coherence (some silver linings were regarded as a negative experience for some women), No/Very minor concerns regarding adequacy, and No/Very minor concerns regarding relevance.	[1–4, 6, 7, 11, 15, 17, 18, 20, 25–27, 29, 30, 33, 35, 36]
	6.3. Information seeking and desire for more information	Information overload, filtering and weighing up options impacted their mental health. Women were careful to ensure the information they encountered was accurate and was not misleading.	High confidence	No/Very minor concerns regarding methodological limitations, No/Very minor concerns regarding coherence, No/Very minor concerns regarding adequacy, and No/Very minor concerns regarding relevance	[1, 2, 5–7, 16, 19, 21, 23, 24, 26, 29–32, 35]

### Assessment of confidence in the review findings (GRADE-CERQual)

The GRADE-CERQual tool assesses the confidence in review findings from qualitative evidence syntheses [83]. Lewin et al., 2018 state that that “the approach has been developed to support the use of findings from qualitative evidence syntheses in decision-making, including guideline development and policy formulation” [83]. The GRADE-CERQual Interactive Summary of Qualitative Findings (iSoQ) online platform was used to manage and assess confidence in review findings [84].

Confidence in review findings was determined based on four criteria: methodological limitations, coherence, adequacy and relevance [83]. For each criterion, review authors determined if there were no or very minor, minor, moderate or serious concerns. An overall GRADE-CERQual assessment of confidence was placed on the findings, levels included: high, moderate, low and very low confidence. Review findings are considered at the highest confidence level and downgraded as there are greater concerns for each individual criterion (Table 4). This process was conducted by two authors, with any disagreements resolved through discussion and consulting other authors.

### Managing our own reflexivity

Throughout the conceptualisation, data collection and analytical process, the authors considered their own individual views and beliefs about maternity care during the COVID-19 pandemic. As clinicians and researchers working on maternity care (including during the pandemic), we recognised that the COVID-19 period impacted indirectly on women and babies, including their experiences of care, their own anxieties and worries. We are public health professionals with diverse backgrounds including nursing and midwifery, maternal and newborn health, epidemiology and qualitative health research. We met regularly, both to explore the findings and the processes but also to ensure that we separated our individual experiences and beliefs on the interpretation of the analysis and the findings. Employing a systematic and transparent approach to the analytical process, such as including reflection notes after analysing each sampled paper, facilitated collaborative discussions, ensure objectivity and reduced the impact of personal biases.

## Results

A total of 36 studies contributed to the synthesis of qualitative evidence to understand pregnant and postpartum women’s experiences during the first two years of the pandemic. There were six over-arching themes: (1) dealing with public health restrictions; (2) navigating changing health policies; (3) adapting to alternative ways of receiving social support; (4) dealing with impacts on their own mental health; (5) managing the new and changing information; and (6) being resilient and optimistic. Seventeen sub-themes were developed within these 6 themes and illustrative quotes are presented in Table 3 to demonstrate theme development. Themes were categorised to differentiate major disruptors to the pregnancy and postpartum period and sub-themes aimed to categorise the indirect impacts that occurred within the major themes.

### Characteristics of contributing studies

After applying the sampling framework (data richness score ≥4), 36 sampled studies were included for data extraction and analysis. Thirteen out of 36 studies had a score of 5 [49, 52, 57, 58, 61–63, 66, 68, 69, 71, 77, 82], with the remainder scoring 4 [9, 16, 50, 51, 53–56, 59, 60, 64, 65, 67, 70, 72–76, 78–82] (S5). Most studies (*N*=27/36, 75%) used specific qualitative methodologies, six were mixed-methods studies, two were cross-sectional studies, and one was a case series report.

Studies were conducted across nine countries, almost one-third (*N*=10/36, 28%) of studies published from the UK, Canada (*N*=7) and the USA (*N*=7) (Table 2). Country-specific responses to the pandemic largely included border closures, mandatory lockdowns and restrictions on movement; it is interesting to note that Sweden did not mandate this but instead enforced social distancing practices [68]. Additionally, some studies reported on a specific sub-population of pregnant and postpartum women, for example women from ethnic minority groups, those with pre-existing comorbidities, and those who were COVID-19 positive. Some studies also included results from women with babies who were greater than 6 months of age, and any findings directly from these participants were omitted from analysis where possible.

The number of participants in studies that conducted interviews ranged from 3 to 84, and studies using qualitative data from open-ended questions or survey data included responses from 16 to 4,611 participants. Where demographic data were available, approximately 1,192 women were primiparous (having their first baby) and approximately 8,017 women were surveyed or interviewed postpartum. Sampled studies were generally of high quality and assessment of methodological limitations indicated that 29 studies were assigned “no or very minor concerns”, six studies were assigned “minor concerns”, and one study was assigned “moderate concerns”. When available, quotes obtained from studies have included additional demographic data. Factors included pregnant or postpartum status at time of data collection, parity and geographical location.

### Theme 1: Dealing with public health restrictions

The rapid introduction of public health restrictions has had adverse effects on mental health, social isolation, and the pregnancy experience. Women had to navigate these restrictions and adapt accordingly, realising quickly that their pregnancy and postpartum experience was going to be very different from their expectations.

#### *Sub-theme 1.1.* Limited support networks from health care system and providers (High confidence)

Support networks were limited. Women felt that they were *“on their own”, “unimportant or irrelevant”* or treated as *“second class citizens”* after birth, because of a lack of physical supports from healthcare providers [51, 60–62, 70, 72, 74]. Limited or no access to physical and social support networks was commonly cited as a reason for deteriorating mental health.

#### *Sub-theme 1.2.* Balancing exposure risk and need for healthy behaviours (High confidence)

Women balanced COVID-19 exposure risks by shielding, either because of health providers recommendations [16, 69] or because they felt it was needed to protect their baby [50, 68, 71, 77, 81]. Women delayed or postponed antenatal appointments [50, 57, 69, 72, 82], opted for induction of labour [74], or waited until labour was quite advanced before attending hospital [60, 61, 77]. These decisions were due to pandemic-induced fear, and the perceived risk of infection in a high-risk environment such as the hospital [16, 56, 80].

#### *Sub-theme 1.3:* Missing out on social opportunities (High confidence)

Women felt sad, unseen and heartbroken that they were not able to have social opportunities, especially sharing their newborns with family and friends [9, 54, 56, 61, 66, 70, 71, 75, 76]. On postnatal wards, women with older children were disappointed that their nuclear families could not visit and bond with their newborn in the early postpartum period [56, 59, 80, 82]. While this was disappointing for many, one woman described still feeling well-supported, “*we were supposed to have a baby shower, the weekend after everything shut down … definitely got a lot of gifts in the mail and people who drop things off …. [we] feel like even though he’s being born in this super crazy time and he doesn’t necessarily get to meet people in person, that they are excited about him and want to support us” (USA)* [52]. Primiparous women felt that they missed the opportunity to share many “firsts” with extended families - one woman said, “*this is my family’s first grandchild so it just breaks my heart they will miss her whole babyhood” (postpartum, Canada)* [64].

#### *Sub-theme 1.4*: Breastfeeding challenges and triumphs (High confidence)

Women that struggled with the lack of support around breastfeeding said, "*when it came time for breastfeeding, I had no idea what to do or any challenges that could come. There were so, so, so many questions and I felt so confused during everything” (postpartum, primiparous, UK)* [60]. Lactation consultations through virtual remote care was considered inadequate by most women [51, 66, 71, 73, 75], especially when practical hands-on education and assistance was needed [51, 53, 72, 77]. These challenges led some women to cease breastfeeding early [51, 62, 73].

Conversely, public health restrictions enforcing women to stay at home allowed some women to practice responsive breastfeeding, without concern for social obligations or visitors [51, 62, 64, 71, 75, 79]. Some women valued this flexibility - “*there’s no right or wrong way. You know, at the end of the day the ultimate goal is that my baby needs to be fed.… you know, feed him breast milk, breast milk, or formula. He’s fed. He’s happy. Sweet. That’s done. Job done! The important thing is actually [to] be kind to yourself, you know?” (postpartum, primiparous, UK)* [62].

Challenges and triumphs were felt by both multiparous and primiparous women [51]. The difference between experienced and first-time mothers was stark in some studies, highlighted by multiparous women who felt ‘knowledgeable’ and ‘had the experience’, and sharing empathetic messages towards primiparous women with limited breastfeeding support [62, 78].However, the lack of face-to-face breastfeeding support meant that first-time mothers and experienced mothers also faced hardships. As one mother recounts her sadness: “*I had virtual appointments [with lactation consultants], which I found totally useless… I was devastated that it wasn’t working with [the new baby] because it was something I was really looking forward to” (postpartum, multiparous, Canada) * [73].

### Theme 2: Navigating changing health policies

The ever-changing nature of the pandemic created periods of uncertainty. Women and their families were expected to accept and adapt to changing health policies which directly impacted their antenatal, labour and birth and postnatal experiences.

#### *Sub-theme 2.1:* A birthing experience filled with uncertainty and unknowns (High confidence)

Many women reported that, given the constantly changing policies, they were unsure what to expect for their labour and birth [9, 49, 60, 75, 77]. Limitations included not being able to have a water birth, use a bath or the shower, access nitrous oxide gas during labour [49, 74, 82] and others could not have their desired support people present [60, 77]. In some cases, women opted for medicalised interventions to retain a sense of control - choosing a caesarean birth to ensure their partner was present at birth [60, 74]. Women struggled with the prospect of early discharge, lacking confidence and fearing reduced support at home, with some feeling pushed out of the hospital [49, 53, 60, 74]. Some women chose to leave hospital early due to the lack of support or poor experiences while in hospital [60]. Conversely, some women welcomed early discharge, wanting to be away from the hospital and to be reunited with family members [62, 80]. Women who tested positive to COVID-19 early in the pandemic described additional challenges, such as a lack of certainty on how care was going to be managed [77]. They felt this restricted their autonomy over their labour and birth choices.

#### *Sub-theme 2.2:* Reduced support and partner presence healthcare settings (High confidence)

Due to the public health restrictions in hospitals, women often missed having their partner and family supports [16, 49, 57, 66, 71]. For example, “*one of my coping mechanisms is having my partner there to hear the same things I am hearing because I kind of shut down sometimes when I get too upset. It’s always good to have that second person listening… and walking out with strength of unity*” *(pregnant, primiparous, Australia)* [49]. The inability for some women to have their partners present negatively impacted women’s birthing experience [53, 70, 79, 80], confidence on the postnatal ward and many expressed the sense of being “*robbed of this experience*” *(pregnant, UK)* [75].

#### *Sub-theme* 2.3: Transitioning to telehealth, virtual and remote care (Moderate confidence)

Public health restrictions limited face-to-face health care appointments with a maternity care provider [54]. Negative telehealth experiences were expressed predominantly by first-time mothers [71], with many saying, “*over the phone just doesn’t do it… you don’t get to look into somebody’s eyes and to trust them and for them to say, you’re okay*” *(postpartum, Ireland)*, adding to their anxieties. This was felt similarly by CALD women as there was a disconnect with health care providers using virtual methods and this was exacerbated for women who were not able to access interpreters [80]. Positive encounters with telehealth were associated with the increased accessibility to health services and generally preferred by multiparous women [54, 65, 68]. Whilst many were glad that telehealth services were available, this woman highlighted the inequities, “*I think I would question the accessibility of that. Not everyone has a smartphone and expecting people to be able to receive a video call is not necessarily the most inclusive thing*” *(postpartum, primiparous, UK)* [77] indicating that some women may have fallen through the gaps of maternity care.

#### *Sub-theme* 2.4: Barriers to accessing health services (High confidence)

The closure of so-called non-essential services, such as, physiotherapists, chiropractors, pools and gyms indirectly impacted women [66, 74]. This often increased women’s anxiety, stress, feelings of helplessness and frustration [16, 54, 60, 74] and incidence of postnatal depression [82]. This also limited opportunities to receive reassurance from healthcare providers, reducing women’s confidence [49, 71, 72, 77]. Typically, women accessed networks for information and support, such as, family and friends with midwifery clinical expertise, or referred to recent pregnancy experience [52, 68, 75, 79]. Women had to advocate strongly for physical assessments for themselves and their newborns [74].

Additionally, women from CALD populations were challenged in accessing culturally appropriate care with changes to interpretation services, *“it creates like a…a gap in communication where if something you express is not clearly understood so maybe they could be left with some misinterpretation” (UK)* [63]. Another example of the inequities faced by CALD women was expressed by this woman who did not receive interpretation services during appointments, “*sometimes they explained things to me by using signs and I understand a little English but it’s hard to understand medical terms and they didn’t use an interpreter for this*” *(postpartum, multiparous, Canada)* [80].

### Theme 3: Adapting to alternative ways of receiving social support

Support networks, such as, family and friends, peer support groups (e.g. mother’s groups), and formal support from maternity care providers provide the foundation for a healthy and positive pregnancy and postpartum period. The COVID-19 pandemic forced women to adapt and seek support in different ways.

#### *Sub-theme* 3.1: Accessing support through different avenues (Moderate confidence)

Support from family and friends was accessed in different ways, for example, utilising video call technologies to be able to see faces helped with the grief of not being able to be present [16]. Women who were able to establish pregnancy and mother’s groups during the pandemic were grateful that they had these supports. Alternatively, women created or sought support through online social media platforms [61, 68, 70, 81], to share a sense of camaraderie that they were not alone in their experiences [52, 77]. In these forums, women shared information about COVID-19 developments, updates to hospital policies, and utilised others as sounding boards for advice. Some women reported greater support from partners who had transitioned to working from home [51, 62, 64, 66, 75]. Although virtual technologies allowed women to bridge the gap of social distancing, they wanted the physical connection with others.

#### *Sub-theme* 3.2: Desiring connection with family and friends (High confidence)

Women felt they needed intergenerational support to raise their newborns, and this was especially important during difficult times. Many had planned for parents to come and support them [81], as they believed that, “*the older generation have more experience on what babies need or what they feel… with my other two [children]… they knew exactly what would make them feel better*” *(pregnant, multigravida, Australia)* [49]. Some women struggled without the additional support, the lack of sleep impeded their physical wellbeing [61, 73, 75], and the isolation from family impacted their mental health [9, 49, 56, 60, 61, 73]. In some cases, women were able to “*quarantine with family*”, providing women with a “*strong support network*” *(postpartum, Canada)* as they transitioned into motherhood [59]. Gradually, as public health restrictions eased, women from the UK felt government responses did not consider new mothers and babies and they called for “social bubbles” for families to receive the additional support [62, 72]. The loss of informal support networks was apparent for some CALD women. As this woman said, *“it was really hard during COVID. In Syria I had my family… but to give birth here with no one with me?! I needed someone with me, my neighbours, my friends… I felt like I was drowning” (postpartum, multiparous, Canada)* [80].

### Theme 4: Dealing with impacts on their own mental health

The COVID-19 pandemic placed a significant toll on pregnant and postpartum women’s mental health at all stages of the pandemic. Public health strategies failed to include protective measures for mental health, as such many women reported increased levels of fear, anxiety, stress, loneliness and depression.

#### *Sub-theme* 4.1: Managing anxiety due to virus-related fears and concerns (Moderate confidence)

Women often experienced anxiety exacerbated by the pandemic, for example, “*as a new mom you are already so nervous, so adding a pandemic to that pile of anxiety and worry*” *(postpartum, Canada)* [70]. This was related to possibility of infection, particularly in hospital and healthcare settings [9, 56, 57, 69, 82], and the need to protect their unborn or newborn baby [50, 72, 80]. Some faced additional challenges as migrants from another country, “*I found it very hard when you’re coming to the country without knowing anyone and the coronavirus, lockdown was very difficult, I was very depressed. I was very anxious… I feel worried a lot*” *(UK)* [63].

#### *Sub-theme* 4.2: Feeling lonely and isolated (High confidence)

Loneliness and isolation were commonly reported as women faced motherhood alone without their usual support systems. One woman said, “*it was quite sad that I couldn’t even share my pregnancy experience with anyone, and I feel like I missed out*” *(postpartum, Australia)* [54]. Feelings of loneliness was especially felt by mothers who were not able to have their partners present during birth or postnatally [61]. Women were not able to build supportive peer networks in their antenatal and postnatal periods [49, 62, 73–75, 78, 81], with one woman saying, “*there’s nothing like just meeting people or, just naturally building friendships when you go to baby groups” (postpartum, multiparous, UK) * [62] emphasising the importance of developing social relationships. Cancellation of appointments and lack of face-to-face care added to feelings of “*abandonment*” and “*being forgotten*” [9, 60, 62, 70, 72, 73].

### Theme 5: Managing new and changing information

Due to the novelty of COVID-19 and lack of information about adverse effects, maternity care services had to rapidly adapt as new data came to light. Women described the need to search, access and filter useful information, a process which was challenging for many.

#### *Sub-theme* 5.1: Constantly changing advice and information (High confidence)

The constantly changing advice was distressing [82]. These changes meant a lot of uncertainty, one woman said, “*at 34 weeks I had a telephone appointment and I tried to ask what the changes in hospitals were, because of COVID and talk about the birth plan. She basically said, ‘everything is changing so quickly there is no point in us even talking about that now. Wait until your next appointment’*” *(postpartum, primiparous, UK)* [77]. This limited women’s ability to adequately plan and prepare for the birth. Some women described following the updates from government officials and hospitals overwhelming [66]. As restrictions eased, women described the frustrations they had with the slow adaptations by health services, “*when I got to the hospital, they didn’t know about the restrictions having been lifted … That was really frustrating because I was like why? Why does this hospital not know?” (Australia)* [82] and the differences between health services, “*restrictions have still not been lifted in ‘Hospital A’ whereas they have been eased in both ‘Hospital B’ and ‘Hospital C’*” *(pregnant, multiparous, Ireland)* [9].

#### Sub-theme 5.2: Inadequate information from healthcare providers (Moderate confidence)

Women felt there was not enough information from healthcare providers, “*I think there was a lot of confusion; there was no good communication about what was happening to appointments. You weren’t really sure; were they happening on the phone [telehealth], when were you going to get the call? There was very little communication. So, I always felt a bit uneasy about that…*” *(postpartum, primiparous, UK)* [77]. Some information was contradictory [60] for example, “*I’ve found the disconnect between the information that my GP was getting and that the [hospital] was getting – they weren’t getting the same*” *(Australia)* [82]. Women wanted clear information that was easily accessed by the lay person [9, 16, 54, 61, 65–68, 75]. They also wanted uncertainty to be acknowledged, “*it would have been useful to have some generic information that went out to women in that situation… statements from a medical professional to put people’s minds at ease*” *(postpartum, Australia)* [54].

### Theme 6: Being resilient and optimistic

Many women were self-reliant and took it upon themselves to remain positive and proactive throughout the perinatal period.

#### *Sub-theme* 6.1: Self-help strategies to overcome challenges of the pandemic (High confidence)

Women developed their own strategies to find solace and support [77]. When asked what advice they had for other women in similar situations, advocacy for oneself was frequently reported [66, 67, 70, 71, 77, 79, 81, 82]. In contrast, another woman regretted not voicing her concerns, “*I have naively trusted that the hospital gives me the information I need … Then I realized afterwards that there were many moms who were much angrier than me and said much more; insisted much more… and I simply did not; I regret it a bit*” *(postpartum, Norway)* [67]. Women reported coping using different strategies, such as being outdoors and active [16, 52, 54], limiting news and access to social media platforms [54, 69, 70, 81], seeking professional help [58, 73], informing themselves about the virus [58, 71], drawing on their own faith and religion [52, 69] and self-reassurance [50, 52, 62]. Many complied with public health restrictions, however there were some women that decided their mental health and physical wellbeing was more of a priority and broke public health restrictions to seek support from family and friends [62, 66, 73]. Despite the challenges faced during the pandemic, some women reported high resilience, positive childbirth and postnatal experiences, and feeling empowered by their ability to overcome challenging circumstances [54, 58, 74].

#### *Sub-theme* 6.2: Making the most out of the positive encounters (Moderate confidence)

The lack of visitors on the postnatal ward and in homes was described by women as “*pleasant*”, “*relaxing*” and a “*blessing in disguise*” as women were able to recover and establish undisrupted routines with their newborns [54, 71, 72]. A commonly reported positive outcome of limiting social obligations was the ability to establish successful breastfeeding, one woman said, “*I was inundated with visitors with my first child and often could not feed responsively… With my second child, there is none of that pressure and I can really see an enormous difference both is his feeding and in my mental health*” *(postpartum, UK)* [51]. Women also described health services as “*peaceful*”, as there were fewer people in waiting rooms, appointments were quick, social distancing was enforced and use of PPE limited the possibility of transmission [16, 49, 71, 75, 81, 82].

#### *Sub-theme* 6.3: Information seeking and desire for more information (Moderate confidence)

Women obtained information from official government documents, guidelines released by professional bodies, the news, social media and platforms run by professional academics [53, 66, 68, 72, 81]. Reasons to seek information included: to clarify any uncertainties about risk and infection, keep up to date with COVID-19 guidelines and to be informed about changes to hospital policies [49, 52, 66, 69, 77]. Even once women were provided with information, poor communication and follow up left women feeling dissatisfied [54]. One woman shared advice about engaging with different information sources – “*you can’t just trust them – you’ve got to decipher through what’s true and what’s not… Is that actually having a positive influence on me, and my mental and physical health, or not? And if it’s a no, well why am I engaging in this*?” *(Australia)* [81].

## Discussion

This QES synthesised data from 36 sampled studies on pregnant and postpartum women’s experiences from high income countries during the COVID-19 pandemic. Findings were categorised under six overarching themes and 17 review findings to understand their experiences as the pandemic unfolded. Women had to navigate the transition from pregnancy to motherhood, whilst also adapting to the complexities of the COVID-19 pandemic. High to moderate confidence was placed in these review findings, indicating the strength of the evidence.

This review highlights that pregnant and postnatal women across high-income countries faced similar yet inherently unique experiences and challenges. During the pandemic, primiparous women faced moderate-to-high prenatal stress levels, as they recounted their first pregnancy experience during a time of significant uncertainty [85–87]. On the other hand, some evidence highlighted that multiparous women were ‘adaptive’ and felt ‘prepared’ [66, 71, 77]. However this was not experienced universally - many experienced mothers facing difficulties [9, 73, 80]. The COVID-19 pandemic and associated public health restrictions across high-income countries disrupted access and quality of care for many pregnant and postpartum women.

Reduced health service capacity and the transition to remote and virtual care due to pandemic restrictions have been heavily criticised [8, 88]. In many contexts, women had not received high quality maternity care during the pandemic and described overtly negative experiences [35, 89, 90]. Women were unable to access usual supports, had limited birth choices and reduced postpartum care which resulted in stress and anxiety. These are clearly widespread experiences, regardless of context, and highlights some of the structural weaknesses and vulnerabilities of maternity care systems. This was evident in the findings for pregnant and postpartum women of culturally and linguistically diverse backgrounds. The lack of culturally appropriate care, including access to interpretation services, doulas and being unable to have their support person present are known to impair maternal health and wellbeing [56, 63, 80]. These factors are key elements of respectful maternity care as they help provide information, enable women’s agency and ensure emotional and social support is available [91, 92]. Health restrictions should not limit this service for women during times of unrest, as women and babies thrive in culturally respectful maternity services [93]. We note however that CALD women continue to be an under-represented group - only three of the 36 sampled studies reported evidence specifically for CALD groups [56, 63, 80]. The lack of diverse perspectives included in the evidence base makes it more difficult for culturally sensitive and community-responsive policies to be developed. Further research with women from diverse backgrounds are warranted to ensure they are not unduly disadvantaged in future pandemics [94].

A key finding was the reduced presence of partner and social support throughout the pregnancy and postpartum periods. Partner support and strong connections with extended support networks reduces stress and anxiety, and can be a positive influence on the woman and her experience [95–97]. In the trade-off between the risk of transmission and spread of disease, expectant fathers and partners were frequently left out [98, 99]. Similarly, studies of families and partners of intensive care unit patients during the COVID-19 pandemic reported being physically and emotionally unable to support partners and families [100, 101]. Close family members are essential to the recovery of patients upon discharge and partners are integral to a safe and positive pregnancy, intrapartum and postpartum experience for mothers. To ensure that maternity care services can adequately respond in the future, recommendations for some degree of flexibility for women given the long-term psychosocial impact that a negative experience would have on the woman and family unit has been sought [8, 87, 88].

Pregnant and postpartum women’s experiences were not universally negative. Another key finding in this review highlights the resilience and optimism that some women felt. Some women perceived this time as a “blessing in disguise” – referencing the ability to stay at home, having fewer disruptions to breastfeeding, and embracing newfound time as a family unit [64, 66, 71]. Coping strategies reported in this study are supported by other evidence of protective factors against stressors of the COVID-19 pandemic [102–104].

Maternity care services need to continue delivering care during public health emergencies. There is no possibility of delaying or postponing care; and women require care over an extended period of time. Enforced lockdowns limited movement and fear of contracting the virus in hospitals lead to delays in healthcare seeking (e.g. when there is reduced fetal movements). The pandemic altered the provision of services and women’s access to care and, as a result, some countries have reported changes to the incidences of stillbirth and preterm birth [105–107].

Understanding women’s experiences, their preferences and satisfaction with maternity care services are essential to a safe and positive pregnancy, labour and childbirth and postpartum period. Many maternity models of care such as woman-centred and midwifery-led care places the woman at the centre of care and her experience, focusing on woman’s health needs, expectations and aspirations [108, 109]. These models have proven to return high levels of satisfaction and are beneficial to the psychological and physiological recovery of the woman [110, 111]. The COVID-19 pandemic has disrupted these models of care for women who were pregnant and gave birth during the pandemic. Pressures on the maternity care system and service delivery did not facilitate the midwife-woman relationship, resulting in poorer clinical outcomes [112]. Supporting women throughout their perinatal period is essential so women and their babies are able to emerge from the experience feeling prepared, safe and satisfied [113, 114].

Moving forward, as maternity care systems adapt to a post-pandemic structure, considerations need to be made to ensure maternity services can adequately respond to future health crises. Our QES has shown that the impacts of COVID-19 went far beyond the direct impacts on women who were infected with SARS-COV2. All women giving birth over the pandemic, especially in the first two years, were indirectly impacted and as a result experienced a lack of autonomy during their pregnancy and childbirth, barriers to accessing face-to-face care and loss of social supports. This highlights the need to consider women’s views and experiences in developing policies for future responses to pandemics or public health emergencies.

We recommend that policy makers and maternity care services should: 1) optimise care delivery to maintain face-to-face care when possible and facilitate the presence of chosen support people; 2) enhance communication channels between maternity care services and women to minimise misinformation, stress and anxiety; and 3) support social and mental wellbeing to ensure women have access to adequate social support and mental health services are well resourced.

### Strengths and limitations

The rigorous and systematic methodology of this QES in selecting studies for inclusion allowed us to analyse experiences of a heterogenous cohort of pregnant and postpartum women during the COVID-19 pandemic. When we started the review, the abundance of published work of women’s experiences was overwhelming, therefore strict eligibility criteria were used to ensure that findings could be obtained and compared across studies. This study was therefore limited to experiences of women in high-income countries and cannot be generalised to low- and middle-income countries.

Studies were subject to a sampling framework to ensure that a diverse, yet data rich sample of studies contributed to the development of review findings. This had its own set of limitations as the sampling framework is not a validated tool and may be biased by the user’s own interpretation. Additionally, the search strategy was limited to the first two years of the pandemic. While it is possible research was published outside of this two-year period, we felt that it was unlikely that different experiences would be reported. An updated search (December 2022) was conducted to determine if any new themes emerged, however no new themes emerged and therefore did not warrant the addition of any new studies. Almost all studies that used interviews to collect qualitative data did so via remote methods. Telephones and video conferencing tools were popular methods to conduct interviews, adhering to social distancing guidelines. Whilst this increased accessibility for participants from diverse geographical locations, there may be concerns about the depth of data obtained and exclusion of participants that are unable to access these technologies. A further consideration is the limited number of studies exploring the experiences of women from diverse backgrounds. This prevented us from more critically examining what factors and circumstances shape women’s experiences and responses.

## Conclusion

Women’s pregnancy and postpartum experience during the COVID-19 pandemic showcased similarities despite different contexts. This QES has collated the experiences of women from high income countries sharing insight into the challenges faced and resilience of pregnant and postpartum women. The COVID-19 pandemic has exacerbated many systemic shortfalls of the maternal and newborn health system – a system that is essential to the health and wellbeing of women and babies. The review findings have highlighted areas within this period where strategies to inform policy and practice could be optimised to allow for better access to care and support for women in their journey to motherhood. Future pandemic preparedness strategies need to maximise face-to-face care, optimise communication channels to combat misinformation and anxiety, include a flexible approach to public health restrictions for women and their families by allowing formal and informal support networks to be readily available and accessible, and to ensure maternal mental health is a priority.

### Supplementary Information

**Supplementary Material 1. **

**Supplementary Material 2. **

**Supplementary Material 3. **

**Supplementary Material 4. **

**Supplementary Material 5. **

**Supplementary Material 6. **

## Data Availability

All data generated or analysed during this study are included in this published article [and its supplementary information files]. Additional information is available from the corresponding author on reasonable request.
