# Supplementary material for: Pregnant and postpartum women’s experiences of the indirect impacts of the COVID-19 pandemic in high-income countries: a qualitative evidence synthesis
Source: BMC Pregnancy Childbirth. 2024 Apr 11;24:262. doi: 10.1186/s12884-024-06439-6 (PMC11007880; doi:10.1186/s12884-024-06439-6)
Supplement: Supplementary file 3 — Supplementary Material 3. [file 12884_2024_6439_MOESM3_ESM.docx]

**Supplementary file 3. Search Strategy**

A reference search will be conducted on EBSCO Medline in consultation with an accredited librarian and will be adapted for included databases. EBSCO Medline was searched on July 25, 2021 and an updated search was conducted on December 2, 2022.

| **#** | **Search Terms** | **Results** |
| --- | --- | --- |
| S30 | S11 AND S17 AND S29   - Limit to humans only and from Jan 2020 | 539 |
| S29 | S18 OR S19 OR S20 OR S21 OR S22 OR S23 OR S24 OR S25 OR S26 OR S27 OR S28 | 3,589,431 |
| S28 | MH attitude | 48,746 |
| S27 | TI belie* OR AB belie* | 307,690 |
| S26 | TI attitude* OR AB attitude* | 158,072 |
| S25 | TI percep* OR AB percep* | 318,312 |
| S24 | TI understand* OR AB understand* | 1,270,893 |
| S23 | TI "point of view" OR AB "point of view" | 44,186 |
| S22 | TI expectation* OR AB expectation* | 93,355 |
| S21 | TI opinion* OR AB opinion* | 100,079 |
| S20 | TI perspective* OR AB perspective* | 350,488 |
| S19 | TI experience* OR AB experience* | 1,147,311 |
| S18 | TI view* OR AB view* | 492,060 |
| S17 | S12 OR S13 OR S14 OR S15 OR S16 | 180,388 |
| S16 | MH "COVID-19" | 82,040 |
| S15 | TI pandemic* OR AB pandemic* | 88,880 |
| S14 | TI coronavirus OR AB coronavirus | 60,229 |
| S13 | TI "SARS-Cov-2" OR AB "SARS-Cov-2" | 38,266 |
| S12 | TI "COVID-19" OR AB "COVID-19" | 119,405 |
| S11 | S1 OR S2 OR S3 OR S4 OR S5 OR S6 OR S7 OR S8 OR S9 OR S10 | 1,353,354 |
| S10 | MH "Mothers" | 46,514 |
| S9 | MH "Prenatal Care" | 29,250 |
| S8 | MH "Postpartum Period" | 26,869 |
| S7 | MH pregnancy | 902,860 |
| S6 | TI antenatal* OR AB antenatal* | 39,401 |
| S5 | TI newborn* OR AB newborn* | 166,809 |
| S4 | TI mother* OR AB mother* | 226,945 |
| S3 | TI postnatal* OR AB postnatal* | 113,608 |
| S2 | TI postpartum* OR AB postpartum* | 55,937 |
| S1 | TI pregnan* OR AB pregnan* | 520,438 |
