# Supplementary material for: Pregnant and postpartum women’s experiences of the indirect impacts of the COVID-19 pandemic in high-income countries: a qualitative evidence synthesis
Source: BMC Pregnancy Childbirth. 2024 Apr 11;24:262. doi: 10.1186/s12884-024-06439-6 (PMC11007880; doi:10.1186/s12884-024-06439-6)
Supplement: Supplementary file 4 — Supplementary Material 4. [file 12884_2024_6439_MOESM4_ESM.docx]

**Supplementary file 4. Data richness score of sampled studies (N=36)**

| **Reference ID #** | **Author and Year** | **Data richness score** |
| --- | --- | --- |
| 01 | Anderson 2021 | 4 |
| 02 | Atmuri 2021 | 5 |
| 03 | Aydin & Aktas 2021 | 4 |
| 04 | Brown & Shenker 2021 | 4 |
| 05 | Charvat. 2021 | 5 |
| 06 | Costa 2021 | 4 |
| 07 | Davis 2021 | 4 |
| 08 | DeJoy 2021 | 4 |
| 09 | Dove-Medows 2020 | 4 |
| 10 | Farrell 2021 | 5 |
| 11 | Fumagalli 2021 | 5 |
| 12 | Green 2021 | 4 |
| 13 | Harrison 2021 | 4 |
| 14 | Jackson 2021a | 5 |
| 15 | Jackson 2021b | 5 |
| 16 | John 2021 | 5 |
| 17 | Joy 2020 | 4 |
| 18 | Keating 2021 | 4 |
| 19 | Kolker 2021 | 5 |
| 20 | Kyno 2021 | 4 |
| 21 | Linden 2021 | 5 |
| 22 | Meaney 2021 | 4 |
| 23 | Mizrak Sahin & Kabakci 2021 | 5 |
| 24 | Ollivier 2021 | 4 |
| 25 | Panda 2021 | 5 |
| 26 | Rhodes 2020 | 4 |
| 27 | Rice & Williams 2021a | 4 |
| 28 | Rice & Williams 2021b | 4 |
| 29 | Riley 2021 | 4 |
| 30 | Saleh 2022 | 4 |
| 31 | Silverio 2021 | 5 |
| 32 | Snyder & Worlton 2021 | 4 |
| 33 | Spatz & Froh 2021 | 4 |
| 34 | Stirling Cameron 2021 | 4 |
| 35 | Sweet 2021 | 4 |
| 36 | Sweet 2022 | 5 |
