# Supplementary material for: Pregnant and postpartum women’s experiences of the indirect impacts of the COVID-19 pandemic in high-income countries: a qualitative evidence synthesis
Source: BMC Pregnancy Childbirth. 2024 Apr 11;24:262. doi: 10.1186/s12884-024-06439-6 (PMC11007880; doi:10.1186/s12884-024-06439-6)
Supplement: Supplementary file 6 — Supplementary Material 6. [file 12884_2024_6439_MOESM6_ESM.docx]

**Supplementary file 6. Summary of summaries of sampled studies**

| **Coded data from Nvivo** | **Summary statements (of authors)** | **Summary of summaries (AT)** |
| --- | --- | --- |
| ****Dealing public health restrictions**** |  |  |
| 1.1. Limited support from health care system and providers | | |
| Riley 2021  Reference 1 - 0.26% Coverage  Participants also “missed out on some of the antenatal classes and things [they] could be doing with new mums” … which provides the opportunity for their child “to get some interaction with other babies  Jackson 2021a  Reference 1 - 0.29% Coverage  “Not too much support for people after you’ve had a baby, really. After you’ve been discharged from the midwife and the health visitors, that’s kind of it. You’re on your own.”  Jackson 2021b  Reference 1 – 0.52% Coverage  “I think they could’ve thought a lot more clearly about the support, new mums-in terms of checking that emotionally they’re okay and also checking their home environment. I actually think they’ve just kind of stopped it without really thinking about the implications of that and I think that, you know, they should’ve continued home visits in a relatively safe way, and yeah, having a proper six week check with the GP I think was important and more ways of checking on the health, you know, the progress of your baby. I just think having such a long period of time without any contact or any checks on the baby at all is dangerous [laughter]”  Reference 2 - 0.47% Coverage  “There’s nothing like just meeting people or, you know, just naturally building friendships when you go to baby groups and things. There was people that you’d see at every group and you’d just start to get, you know, become friends because you were always there and things so erm, just- especially some people would really struggle probably to meet new people, anyway. So erm that must be really challenging… It’d be great if they could start [baby groups] again, especially for non-mobile babies ‘cause you could do the social distancing.”  Reference 3  “I do feel like we’ve been let down, to be honest. I do feel like we’ve been let down. That and new mums, ‘cause even from a safeguarding perspective, it’s just not fair, that support. I don’t really understand why it’s not [sigh] yeah, why it’s not been prioritised to this extent.” (Participant 16, T2).  Ollivier 2021  Reference 1 - 0.30% Coverage  “I also feel as if moms are being treated as second class citizens as they no longer oﬀer 6 weeks postpartum check-ups meaning that we are left to decide if things seem normal or not after giving birth.”  Panda 2021  Reference 1 – 0.49% Coverage  “I do remember like we had the breastfeeding groups [after a previous birth]. That I would’ve went to, like weekly [facilitated by local PHNs]. And that was great because you’d be able to get the baby weighed… just reassurance that you were on track in terms of weight gain… That is deﬁnitely something that, you know, I mean COVID has taken away.”  Sweet 2022  Reference 1 - 0.12% Coverage  “I really didn’t get proper support from the child health nurses until week five. … I actually got a bit of post-natal depression this time as well”  Reference 2 - 0.15% Coverage  “I wasn’t told about mother’s groups, I did find about them myself but I know that quite a few of the others in my mother’s group had to search for themselves as well, they weren’t told about it”.  Anderson 2021  Reference 1 – 0.61% Coverage  “All the classes have been cancelled, and you can’t just go… you feel like you haven’t got time to sit in there and have a chat and ask these questions that you want to ask”  Atmuri 2021  Reference 1 - 0.32% Coverage  “You can’t attend some yoga or birth classes so you can’t meet some other mums as well. You can’t ask advice or experience from them. Because of this, I have to do it by myself.”  Reference 2  “Maybe setting up something like a support group, maybe an online support group . . . for mums that have babies in a pandemic that probably do feel isolated.”  Davis 2021  Reference 1 - 0.18% Coverage  “For six months, there’s a whole group of mothers from late February to March/April that were just forgotten about and those mothers, some of them now at nine months make no connections with any other mothers because they were just left.”  John 2021  Reference 1 - 0.29% Coverage  “So COVID kind of, umm, robbed me of that experience which I’m used to back at home, where I used to attend my clinics… So clinics gave you that… that… that, umm, avenue of socialisation, meeting another mothers, new mothers……creating, umm, friendships… and… and also, you know, a continuance after birth”  Reference 2 - 0.26% Coverage  “I could sense that she was busy and she could have rather been off, you know, not having that one extra person popping into triage at that point. For me, it was just a case of, well, you know, I need this, so I can only apologise, but there’s nowhere else I can go.”  Mizrak Sahin & Kabakci 2021  Reference 1 - 0.15% Coverage  ‘I cannot go [to the hospital] right now, but I contact my doctor by  phone, and s/he asked me not to come unless I had a problem’  Silverio 2021  Reference 1 - 0.26% Coverage  “…it’s just a bit more on you that it maybe would have been otherwise, because all these clinics are not running and the regular appointments are not happening, or at least not face-to-face. Maybe that’s what I would say, is that the support is there and really good, but I think generally it is on you to enquire”  Reference 2  “I would say: don’t have any expectations for the midwife care up until the point of labour. At the time you feel cheated out of these appointments. You keep looking at this book that tells you, you should have been seen in all these weeks and you are thinking, I have been seen face-to-face twice. You feel a bit cheated and anxious that they maybe are missing out on something…”  Ollivier 2021  Reference 1 – 0.56% Coverage  “I feel my mental health is suﬀering because of missing connection with other ﬁrst time moms”  Harrison 2021  Reference 1  “When we went into lockdown I couldn't get hold of the midwife, all my appointments and training was cancelled, the hospital didn't have any information, and everybody was panicking. It took about 4 weeks for information to get to me. I didn't see my midwife from week 24 and had different ones every time because of COVID.”  Reference 2  “The midwife appointments were cancelled. I was made to feel unimportant and irrelevant.”  Reference 3  “I have not had a postnatal check-up - I phoned re: this and was told they weren’t doing this ‘unless there was a problem... was there a problem?’ Given that I am not a healthcare professional I couldn’t tell you if my uterus was in the right place, if my stomach muscles had knitted together again, or if my mental health was in the right place. This was frustrating.”  Meaney 2021  Reference 1  “To have had classes as a ﬁrst time mom, not just have classes cancelled and feel abandoned by the system. Medically I know I’m ﬁne which is one thing but I’ve felt angry that nothing was put in place to replace the classes online. I would have liked more time with midwives to chat and get to know them or a doula that could be there on the day.” (P206; Ireland, 35–39 yrs, nulliparous, third trimester)  Rhodes 2020  Reference 1  “I had an episiotomy and I’m still in pain 7 weeks later. The doctor said no six-week checks … eventually the doctor gave me a call but I haven’t had an examination. I feel as if I have been short changed.“ [Baby 7 weeks, age 30-34 years, White British]  Reference 2  “I didn’t get the aftercare I was expecting and I’m in agony but not able to get real help from the NHS. Just feels like I had my baby and then abandoned.” [Free text]  Reference 3  “I just feel like new moms and parents have just been completely left out of the Government’s mindset and their exit strategy really.” [Baby 3 months, age 30-34 years, White British]  Reference 4  “I don’t think I am being supported. The Government should be giving us more information, like where do you go to get your baby weighed…there were 8 weeks when she wasn’t weighed – quite a worry really.” [Baby 12 weeks, age 30-34 years, White British]  Brown 2021  Reference 1  “The lack of support has really upset me. Sad, sad situation and not what I hoped it would be like at all. I researched so much before baby was born and cannot access any of the support I thought I could.”  Spatz & Froh 2021  Reference 1  “Sadness of not being able to have those experiences due to social distancing”  Linden 2021  Reference 1 – 0.65% Coverage  “It’s sad that parent groups didn’t take place, during pregnancy, to meet people that way. But, now there is a lot on the internet… so I have gotten in touch with a mother via the internet, so that you get the social interaction…”  Dove-Meadows 2020  Reference 1  Participant (P): You know, I couldn’t even have a baby shower because of everything.  Interviewer (I): Were you planning on one and did  you have to make it like a virtual one or did you just cancel it completely?  P: I just canceled it completely.  Green 2021  Reference 1  “My appointments with my midwife have been more spaced out than it would have been if COVID-19 was not happening [Pregnant #26].”  Reference 2  “I can no longer attend my perinatal yoga class which I really enjoyed [Pregnant #27].”  Rice 2021b  Reference 1  “I’m still not that healthy, and the follow up isn’t great. My six-week postpartum check-up for stitches was a phone call. I thought I had an infection, so I called the OB office like once every couple of days for about four weeks and they kept saying “We’ll get back to you, we’ll get back to you, we’ll get back to you.” So, I finally was able to get a hold of my family doctor who gave me a prescription because I ended up having a uterine infection. (…). Finally, after calling every two days for four weeks, hospital personnel said, “Instead of a phone call at eight weeks postpartum we can look at your stitches real quick.” So, I did get somebody finally to look at them and they were fine… And since then, I’m still trying to get blood work done. I’m on a waitlist to get blood work done because for some reason I still can’t get up holding [my daughter]. I just have zero strength, zero energy.”  Snyder 2021  Reference 1  ‘‘Having that community of women of being able to sit down every week and talk about how things are going or just someone that’s in a similar situation’’ (31, Caucasian, nurse). | Participants felt they had to ‘get on with it’ following the birth of their child with little professional support. [Riley 2021]  Diminished care, distress, and desertion Many respondents at T2 received very little healthcare professional support in the early postnatal period, which exacerbated feelings of loneliness. Respondents expressed deep sadness and disappointment in being unable to share their life transition through face-to-face interactions with family, friends, and other new mothers. [Jackson 2021a]  Many of the participants acknowledged that the perinatal period is a particularly vulnerable and disruptive time in a woman’s life and expressed that they would have liked a more active Governmental response to informing new mothers of where to find support during this difficult time… Criticisms were also aired regarding insufficient structural support, and concerns were raised regarding the potential consequences of such disrupted support. Other frustrations regarding COVID-19 restrictions included the closure of parenting support groups. Many participants expressed a desire for support groups to be reinstated. [Jackson 2021b]  Mothers also expressed that they did not always feel supported and recognized by health care providers, which added to their stress … This quote demonstrates the frustration felt by the majority of mothers in our study. Again, feeling left behind and struggling to ﬁgure out what is ‘normal’. Figuring out what is normal is stressful and an important part of the postpartum period in non-pandemic times. Having limited access to postpartum services created an extreme situation for many of the mothers in our study. [Ollivier 2021]  Mother and baby groups after birth, many of which are facilitated by PHNs who provide infant feeding support also, were now unavailable to women who would have considered joining these. [Panda 2021]  The changes in postnatal healthcare caused much frustration and dismay [Sweet 2022]  Loss of maternity care. While some women felt well supported by their midwives, many reported cancelled appointments and classes, face-to-face appointments feeling rushed and stressed and feeling unable to ask questions or share positive emotions. Telephone appointments felt less personal, more removed. They also felt acutely the loss of antenatal classes – for the important information they were missing out on and the chance to meet other pregnant women. [Anderson 2021]  Many women described that they valued peer support from other mothers during pregnancy and post-partum, together with intergenerational support from parents… Women were solution focused and proposed greater utilisation of the internet medium for online video hospital tours and support groups. [Atmuri 2021]  The majority of women highlighted that they needed increased access to support, particularly face-to-face services. Most frequently, women discussed their needs to access Child Health Nurses (CHN) once they had their babies, as women believe they are important for mums’ emotional support as well as the child’s development. Some women expressed their frustration about delays in appointments, with one mum reporting delays of up to 2–3 weeks to see a CHN [Davis 2021]  Most women reported feeling isolated during their pregnancy due to features specific to the SARS- CoV-2 pandemic. This was particularly a problem for those who felt that they would have benefitted from the presence of a companion when important information relating to their pregnancy was being relayed to them. Although most women were understanding of the limitations posed by the pandemic, a significant proportion expressed loneliness exacerbated by not being able to engage with their usual pregnancy support networks, and did not feel that virtual groups mitigated this effect… Participants who were satisfied with the maternal health system made reference to respectful communication. This was particularly important during episodes of high maternal stress (box 1: Respect, P15). Participants concurred that community midwives and health visitors were considerate in their routine communication (box 1: Respect, P1). A significant majority of the group conversely felt that they received disrespectful care during unsolicited hospital visits. [John 2021]  During the interviews, pregnant women were warned by their doctors not to go to the hospital unless there were mandatory examinations, tests or any difﬁculties. [Mizrak Sahin 2021]  In addition, women discussed how reduced frequency of antenatal care appointments made them feel, with provision of virtual care not equated to the in-person care they had either expected or wanted… Whilst not favoured in the postnatal period as much as it had been accepted for certain aspects of antenatal care, virtual care was tolerated as a better alternative to no care at all, which many postnatal women faced [Silverio 2021]  Overall, mothers felt that they were missing out on social connections and commonly described feeling isolated, alone, helpless, like a burden to others, and very exhausted trying to independently manage being a new parent without support from others or the comfort in knowing that others were experiencing similar struggles… The importance of socialization for mothers is often overlooked and not recognized as a vital part of the postpartum period. However, when it is associated with improving mental health, building conﬁdence and ensuring self-eﬃcacy, socialization becomes vitally important… Socialization for mothers was also of concern. Many expressed missing their mom groups, coﬀee chats, or parent-baby classes, all of which were opportunities they would usually use to connect with other parents to help their mental health and share common experiences, questions, or concerns. [Ollivier 2021]  The participants also mourned the loss of other rites of passage like childbirth preparation classes that they had hoped to experience with partners. One participant reported that she was watching YouTube videos because the childbirth classes were cancelled. [Dove-Meadows 2020]  Perinatal women also reported changes to perinatal medical supports as a result of COVID, including less frequent and altered perinatal medical visits, as well as being unable to participate in perinatal groups. [Green 2021]  Many hospitals changed to provide antenatal classes online. One woman felt that doing pre-recorded classes took away from the experience as it was less individualized. [Keating 2021]  Under the theme maternity care impacted by COVID-19 (see Table 3), some women reported dissatisfaction with changes to the maternity services which were implemented to reduce the risks of transmission of the virus. Women reported, “how the virus has ruined everything and changed maternity care”, whereby routine antenatal appointments and antenatal/parenthood preparation classes were postponed, cancelled or telemedicine clinics were provided in lieu of in-person appointments. Women reported that antenatal care is “vital” with antenatal classes considered “an essential service” that needed to be promoted in order to empower women to maintain their health and wellbeing during pregnancy. [Meaney 2021]  Missing out on antenatal groups was also disappointing for women, although some were attending existing groups on the web or had found new online support groups or forums to compensate…  Without antenatal and postnatal groups and visits to family and friends, respondents felt that they were missing out on opportunities to share experiences and learn from others. [Rhodes 2020]  By contrast, many women summarised their experience of postnatal care in the community as feeling “forgotten” or “abandoned” and described the reduction of professional support as increasing their stress as new parents. They drew particular attention to midwives and health visitors replacing face-to-face visits with telephone calls and reducing the number of visits or offering no postnatal contacts at all; the difficulty of contacting health professionals when their advice was needed; the closure of health visitors’ clinics where babies could be weighed and checked; reduced access to other services such as breastfeeding support; and GPs cancelling the postnatal check-ups for mother and baby or replacing face-to-face checks with remote consultations. This had left some new mothers worried that their own physical recovery and mental health had been neglected, unable to access contraception, stopping breastfeeding sooner than they wanted, and unclear about whether they were caring for their baby appropriately. Some had experienced serious health consequences with undiagnosed infections in their caesarean wound, perineal tear or episiotomy site. The advice and reassurance from health professionals was particularly missed by first-time mothers and those who were cut off from family support by distance or legal restrictions on contact with other households because of Covid-19. [Harrison 2021]  Many participants talked about missing meeting other breastfeeding mothers and socialising in baby groups or out with friends. Sometimes, this was about asking others questions or seeking reassurance, but often, it was just about connection and feelings of community. Many talked about the isolation they felt, which was impacting their well‐being and mental health. [Brown 2021]  The mothers described how they had planned to spend time with support groups or with other new mothers in their social circle but reported sadness of not being able to have those experiences due to social distancing. [Spatz & Froh 2021]  As we have demonstrated, many women across Canada received reduced hands-on prenatal and postpartum care during the pandemic due to COVID-19 policies and practices, even in regions with few or no COVID-19 cases. In some instances, such as with the participant who nearly died of a postpartum hemorrhage in hospital, neglect posed far more immediate risk than possible COVID-19 infection. [Rice 2021b]  Many women expressed missing out on the friendship and companionship of other pregnant women. All group activities such as parent education classes that would normally take place during pregnancy were cancelled due to the pandemic and expectant couples were referred to official healthcare information webpages instead. Despite this, some women found alternative ways of getting in touch with other pregnant women, for example on social media. [Linden 2021]  Elements related to a desire for increased emotional support networks were most common. This was frequently related to a desire for in-person peer-to-peer support, especially from mothers who had multiple children. One mother reported missing her weekly breastfeeding support group. [Snyder & Worlton 2021] | **[Lack of support available to women]** Women reported not feeling well supported as new mothers in the early postnatal period. The lack of Government directives and support from health services meant that women struggled to cope, feeling lonely and frustrated with their experiences. Many women criticised government’s response to the management of maternal health care services, with limited healthcare professional support available to those that needed it and felt they missed out on physical support, education and social opportunities. Some women were reportedly told by their healthcare providers not to come to the healthcare setting, only to enquire during emergencies. The reduced frequency of appointments also made them feel neglected and forgotten. (Riley 2021, Jackson 2021a, Jackson 2021b, Ollivier 2021, John 2021, Mizrak Sahin & Kabkaci 2021, Silverio 2021, Harrison 2021, Green 2021, Rhodes 2021, Rice 2021b)  **[Missing out on the experience: closure of antenatal and postnatal groups]** Prior to the COVID-19 pandemic, mother and baby groups provided an opportunity for new mothers to meet peers and develop social groups for their baby and themselves. Majority of women shared a desire for these groups to be reinstated to reconnect with others in the same situation, utilising these groups to ask questions, seek advice, and share experiences, both positive and negative. Due to the closure of antenatal classes, mother and baby groups, pregnant and postpartum mothers felt that they missed out on the opportunity to meet other mothers in similar situations. Women reported that they were disconnected and isolated from society with increasing public health restrictions such as social distancing, and lockdowns. The inability to share experiences with others increased anxieties and negatively affected the mental health of many women. Those that were not able to experience this felt left behind and disconnected from their peers, losing the opportunity for new friendships and continuation after birth. Mothers described the need for socialisation and the positive impacts that it could have had on their experience and ability to develop networks outside family and friends, connect with peers and develop social networks for their babies. (Jackson 2021b, Panda 2021, Sweet 2022, Davis 2021, Olliver 2021, Anderson 2021, Atmuri 2021, Riley 2021, Linden 2021, Dove-Meadows 2020, Keating 2021, Meaney 2021, Brown 2021, Spatz & Froh 2021, Rhodes 2020, Snyder & Worlton 2021) |
| 1.2. Balancing exposure to risk and need for healthy behaviours | | |
| Anderson 2021  Reference 1 - 1.07% Coverage  “So I have had a midwife appointment over the phone rather than my midwife, and I am having less appointments than I would normally have which is quite worrying, but you have to weigh up the risk of having the appointment and catching the virus, exposing yourself in a high-risk environment like a hospital.”  Reference 2 - 0.11% Coverage  One said her obstetrician told her to “basically go home, stay home, don’t go out, don’t see anyone”  Sweet 2021  Reference 1 - 0.04% Coverage  “all you want to do is keep safe” … “we kind of came up with our own plan to stay safe”  Reference 2  One participant said, “we’d actually pulled our kids out a week earlier from school, because we kind of saw the writing on the wall, we’ve got a baby coming, obviously we were like we don’t want to risk it ” (P23) .  Reference 3  Similarly, another was vigilant about minimising exposure, she said “I knew before he was born that I didn’t want him meeting people straight away –I wanted to continue with social distancing even with family, grandparents–I was quite panicked about it ”(P7) .  Aydin & Aktas 2021  Reference 1 - 0.20% Coverage  As I said, I had a slight bleeding at first, I didn't know what to do, should I go or not? If I go, there is a risk of getting a virus … I postponed going to the doctor as much as possible….  Reference 2  “A worldwide pandemic has been declared. I was worried about going out, and I didn't want to go anywhere … Other than my general health, I am also pregnant, I have a baby inside me. I have to protect it too, so I stayed home…” (P6)  Reference 3  “… The doctor told me to come back two weeks later. I went one month later and generally postponed my appointments. Once, my liver enzymes increased, so I had itchiness. But I didn't go to the doctor. The doctor got angry with me for not going to the control….” (P7)  Reference 4  “Unfortunately, I could not go to have tests like detailed ultrasound and triple screening during this period. At that time, the virus was very intense in Trabzon … our appointments were constantly being cancelled. It scared me a lot, so I decided not to go to the hospital.”  Farrell 2021  Reference 1 - 0.75% Coverage  “If all of this [COVID‐19] wasn't going on, considering this is my first pregnancy and how I am, I probably would have went through with everything possible under the sun […]. But because it's [COVID] going on, I just kind of was like, ‘Do I really need this done? Do I want to go through with that and be back at another appointment?’ […] ‘No. I don't really want to come back. I'll just come back in a month. I'll skip it.’”  Reference 2 - 0.42% Coverage  “I probably would have considered testing later, in the early spring when I was still more newly pregnant. I feel like that was right at the height of the pandemic, or at least it felt like it was […] If the option for later had been there, I probably would have gone to it later”  Reference 3 - 0.46% Coverage  “I think it is a more high anxiety thing. […] So going anywhere is stressful, especially where there is sick people. So I guess when I have to go anywhere, whether it is for the genetic testing or not, I don't think that is a deterrent for me having to go to the hospital and considering if I was going to have the genetic testing […], or to go to the hospital to have testing done. There is definitely a level of stress that comes along with that”  Harrison 2021  Reference 1 - 0.03% Coverage  “I was told that my husband would not be allowed into ward and therefore we waited at home during labour for too long. My daughter was born in the bath.”  Reference 1 - 0.09% Coverage  “I was continually rejected from maternity triage on the grounds that they did not want people constantly coming and going from triage due to COVID-19 …I was finally 'let in' to hospital when I had reached 8cm dilation, with just a fading dose of paracetamol for this extremely progressed labour… I think I am probably a little traumatised by going into labour for the first time and feeling as though I will not reach the hospital in time.”  Jackson 2021a  Reference 1 – 0.30% Coverage  “I was like having contractions and erm… every time I thought about going into hospital, it was slowing down and- I don’t want [labour] to go on for ages. The more I was like worried about it, the longer it was.”  Reference 2 – 0.31% Coverage  “I ended up saying [to healthcare team] well you know, “I don’t want to be induced on that particular day, can we postpone this by a week?” so hopefully within a week [baby] would make an entrance all by himself.”  Mizrak Sahin & Kabakci 2021  Reference 1 - 0.18% Coverage  ‘I never went [to the hospital] after the pandemic started, so when I called the hospital, I couldn't reach my doctor, I couldn't ask what  to do, so I never went there'  Reference 2  “I actually had a planned sugar loading test. The doctor said that I should have it, but I didn't do it . . . I am afraid of going to the hospital and being infected by a contaminated surface.” (P11)  Reference 3  “I don't have a particular doctor yet I was in the process of research, so I put it on hold now as this pandemic started, so I'll take care of it later.” (P1)  Reference 4  “I used to visit [the hospital] regularly before, like, once in every three weeks, and now the last time I went, my doctor told me not to come as s/he accepted patients with COVID. I have an examination, so I will go to the hospital only for it.” (P10)  Silverio 2021  Reference 1 - 0.90% Coverage  “I didn’t mean to labour at home for so long, and the reason I did was because the hospital kept saying I wasn’t in established labour because my contractions weren’t at the right time that indicates that you’re in established labour. And it’s really hard to know when you’re a ﬁrst-time mum if you are or aren’t, and if the hospital’s telling you that you aren’t, you just go with it. [Sigh] And obviously, by the time I had got there I could have been at risk of having him on the way there, for instance; I mean, I was 9 [and a half] centimetres. So, I think that needs… I don’t know what the solution is, but reviewing that, because saying “Right, ”and the only reason I was putting it oﬀ was because I didn’t want to go in and my husband being turned away, and it was that fear. So, I don’t know what the solution is to that, but if there’s a better way of advising women when to come into hospital, because I can imagine I’m not the only one that’s put it oﬀ because of not wanting to go in and then losing their birthing partner, whoever they may be.”  Reference 2 - 0.41% Coverage  “I’d also at that point spoken to my Midwife and they said that both of us would have our temperatures checked when we arrived. So, I was actually more concerned about my husband catching COVID, because obviously if I had it, they would [laughs] let me in regardless, whereas if he had it, he wouldn’t be there for the birth. So, we had been really, really anxious about going out, and I think by my 30-week appointment we hadn’t been out for at least a month, we hadn’t left the house”  Sweet 2022  Reference 1 - 0.16% Coverage  “I missed a lot of my appointments in the last part of my pregnancy due to COVID concerns, … potentially having to quarantine for 2 weeks, get the scan, come home, and quarantine for another 2 weeks”.  Reference 2  “from my window I could see into the COVID testing clinic … and I could see people coughing– I thought those people all had the virus and that’s why they were there … I was scared to leave the hospital thinking what if I walk past someone who’s infected and myself and my only baby gets sick”  Rice 2021b  Reference 1 - 0.62% Coverage  “I believe if I’m induced and we’re in hospital, my husband can be there from the time I’m induced until one hour after delivery, then he has to leave. And then he can’t return to the hospital until the baby and I are ready to leave the hospital. If I naturally go into labour, I actually have to go into triage into the hospital alone, so he can’t come in with me. I have to go in alone and get assessed. If I’m in labour, then he’s allowed to enter the hospital only when I’m actually ready to deliver. So, he can’t actually be there supporting me until they’re ready to put me into the delivery room.”  Reference 2  My obstetrician said “FYI, it just so happens that I’ve got a spot for an induction. If you would like to go on Saturday, you can go Saturday.” I was like, “You know what, let’s do it,” because with COVID-19, I just wanted to get him and make sure we were home safe (…). I think I was just so tired, so over it at the end, and so sad of being kind of isolated, that probably didn’t help my decision. But the doctor had said “Listen, I have this spot if you want an induction, and we want to get him out. It’s up to you.” (…), I think that mothering instinct kicked in, and I thought if I can just get him out and get him here with us, I can keep him safe. Whereas, if I had to wait another week or two weeks, and the numbers were outrageous and the hospital was inundated with COVID-19 patients, would I want to be there at that time? Probably not, so I thought I would just take that [induction spot]. [My obstetrician] said that the baby was healthy enough, he was term enough, that he could be out and managed without a NICU stay.”  Linden 2021  Reference 1 - 0.43% Coverage  “I think it was quite a lot [due to] that the uncertainty made me withdraw even more, like then I ignore everything so I do not have to feel that I have done something risky or stupid”  Reference 2  “We are not going to meet anyone […] we felt pretty quickly on our daughter that she did not like the situation of just being at home, at home, at home […] It is not so easy to explain to a barely three-year old” (Interview no. 7, I-para)  Panda 2021  Reference 1  “Going out and doing the food shopping I stopped entirely I was kind of not really getting out because being pregnant I was very conscious that I had to be that little bit more careful.” (ID 13)  Fumagalli 2021  Reference 1  “I was reassured by the fact we were all home together with no contact with the outside world.” (W16)  Reference 2  “You go at the emergency department and if you don’t have it [the virus] you risk to be infected, if you have it you risk to infect other people.” (W14)  Reference 3  “I asked to give birth at home […] I was afraid of the hospital because I had never been admitted to hospital before, I had to give birth and there was an ongoing global emergency.” (W12)  Jackson 2021b  Reference 1  “I felt almost guilty for going to appointments, I didn’t feel guilty, but I was made to feel kind of like I shouldn’t be there and it was all of a bit of an extra inconvenience, and that wasn’t term that wasn’t a good way to feel.” (Participant 20, T2).  Rhodes 2020  Reference 1  “I’ve been recommended to attend the ‘birth afterthoughts’ service to deal with my feelings over difficult birth but am wary of attending any hospital appts that are not absolutely necessary. Similarly baby is supposed to be referred to paediatric cardiac specialist- I’ve heard nothing...and hesitant to chase for same reasons.” [Free text]  Dove-Meadows 2020  Reference 1  “... It’s all over the news everywhere, so it’s pretty scary because thinking like, uh, I could go to the hospital for a check-up and come out with something that’s potentially life-threatening”  John 2021  Reference 1  “She can’t phone the GP to describe what is the problem. And because of the Corona, she understands that it’s not easy to get an appointment. So she tried to read on the internet how she can help herself”  Stirling Cameron 2021  Reference 1  “It was hard because I didn’t know how the situation was in the hospital, I was scared that the hospital might be an epicenter of the pandemic. I was scared that the virus spreads easily in the hospital.”  Snyder & Worlton 2021  Reference 1  “Mainly it was probably a lot more stressful um I don’t know if that has to do anything with my supply going down and like work all included in there, I’m not sure how that affected it but it’s a lot more stressful I know that. Because for the longest time I was just really like not wanting to leave the house and if I had to like any kind of symptom like if I had a little itch in my throat I’m like oh my goodness do I have something? Am I going to pass it on to her through breastfeeding?” | Many were taking extra precautions such as limiting  their healthcare appointments and engaging in other behaviours that did not relate to social distancing but that aimed to reduce risk of exposure to Covid-19. These included washing and quarantining shopping, quarantining post or asking partners to do this before items came into the house… By contrast, some women reported a minority of friends or family who did not adhere to the rules, though this did not influence their own social distancing behaviours. Women chose to reaffirm and explain the rules or keep a distance, with one participant reporting losing friends over this. [Anderson 2021]  The pandemic raised the participants’ awareness of risk and the need for infection prevention measures in their everyday lives. Keeping themselves and their family safe became an important daily activity. Participants described experiencing and managing fear mongering from others. With so much unknown about the risk of COVID-19 in pregnancy, the women described pressures from other people around them. [Sweet 2021]  Some of the women who had prenatal visits at state hospitals reported that they postponed them because of the risk of coronavirus either with their own decisions or at the request of their doctors… Primary school graduate pregnant women living in the village with extended family who had antenatal visits in state hospitals stated that their appointments were generally cancelled, and then they decided not to go. [Aydin & Aktas 2021]  Yet, for some, the pandemic presented too much of a threat. For these participants, the decision for testing was either delayed or deferred, even when it may have been seen as a value before the pandemic. As described by this participant who decided against testing during the pandemic… At the same time, some participants spoke of the intent or decision to postpone prenatal genetic testing. For some, this decision pertained to the status of the pandemic at the time of the available window for testing. This was a time with rapidly evolving information about COVID‐19 in addition to shifting policies with respect to healthcare delivery and COVID‐prevention among healthcare systems and communities. For this participant, the factors occurring at the onset of the pandemic would have affected decisions about test timing… Participants who sought prenatal genetic testing despite the concerns of COVID‐19 reported the anxiety they experienced in the process. Participants were conscious of the risk of exposure to the virus when presenting for testing. For some participants, their concern was heightened due to the  location where they had to present for testing. One concern pertained to being in a healthcare facility where many other patients were seeking care and the choices those patients may make concerning infection‐prevention approaches (e.g., social distancing, wearing masks) [Farrell 2021]  There was a general concern for the safety of perinatal appointments being held at hospitals, which had a significant impact on delaying contractions due to associated anxieties. For this respondent, such anxieties resulted in attempting to delay  planned labour induction in the hopes that the mother would be able to have a homebirth instead of being admitted to hospital. [Jackson 2021a]  Some of the pregnant women interviewed postponed their control upon the recommendations of their physicians, while some pregnant women did not attend pregnancy controls at their own discretion. These pregnant women often made such a decision as they could not contact any medical staff or they were afraid of getting infected with the virus… During the interviews, pregnant women were warned by their  doctors not to go to the hospital unless there were mandatory examinations, tests or any difﬁculties. [Mizrak Sahin & Kabakci 2021]  Whilst P5, an island resident with limited local healthcare services, said “I missed a lot of my appointments in the last part of my pregnancy due to COVID concerns, … potentially having to quarantine for 2 weeks, get the scan, come home, and quarantine for another 2 weeks”. She decided that the restrictions were more burdensome than the potential benefit of the ultrasound scan. When close to her due date, P5 had to negotiate with the authorities to stay in short-term rental accommodation close to her place of birth venue, rather than the government approved quarantine hotel which was much further away. [Sweet 2022]  Wendy from Mississauga opted for a scheduled induction despite having had a fast and uncomplicated childbirth with her first child. Her reasoning was thus… [Rice 2021b]  One woman skipped midwifery appointments in fear of being infected at the maternity care clinic. She later had to seek care from a psychologist because of these intense fears of being infected, a fear that affected her everyday life and all aspects of her pregnancy… All women were worried about their partner getting sick with COVID-19 or another infection close to the birth of the baby, and thus not being allowed to enter the hospital during the birth. Therefore, some families decided not to take any risk of being exposed to the virus and decided to isolate themselves during the last few weeks of pregnancy. Some women took this in their stride and thought of it as a way to relax before the birth, whilst other women expressed that the last few weeks of the pregnancy were very stressful stripping away the peace and quiet that they would have needed to prepare for the arrival of their baby. [Linden 2021]  Some women had tried to avoid being alone during labour by staying at home for as long as they could bear it, and some reported that they had been ‘turned away’ by the hospital in early labour because of Covid-19. This had led to some babies being born at home without a health professional present, or very shortly after arriving at the hospital. Women who arrived at hospital in advanced labour described the pain and humiliation of having to find their way alone from the door of the hospital to the labour ward, where the progress of their labour was assessed before their partner was admitted. [Harrison 2021]  Furthermore, COVID-19 associated restrictions prevented women from seeking and having face-to-face access to the support of other mothers, female friends and joining mother and baby groups. This postpartum isolation was an extension of antepartum isolation where many women described conﬁning themselves to their home while pregnant to protect themselves from COVID-19 transmission. Although most women were accepting of this as part of the reality of the pandemic, women were impacted both emotionally (sadness) and physically (being extra careful and isolating) as a result… This postpartum isolation was an extension of antepartum isolation where many women described conﬁning themselves to their home while pregnant to protect themselves from COVID-19 transmission. Although most women were accepting of this as part of the reality of the pandemic, women were impacted both emotionally (sadness) and physically (being extra careful and isolating) as a result… Routine GP care was disrupted during COVID-19, both antenatally and postnatally. Some women were happy to avoid their GP surgery as they viewed it as a potential site for COVID-19 transmission. [Panda 2021]  The women referred to their home as a physically and emotionally safe environment during the ‘pandemic’ pregnancy: physically safe in terms of feeling protected from potential contagion and emotionally safe in terms of sharing the childbearing event with their partner... Before testing positive, most women perceived the hospital as a frightening and dangerous place during pregnancy, when they were worried about COVID-19 transmission. For this reason, they were resistant to accessing healthcare facilities if not extremely necessary. Prior to testing positive a woman requested a home birth, as she feared hospitals during the pandemic emergency. [Fumagalli 2021]  Many women felt vulnerable and chose to cocoon despite the national recommendation that this was not necessary. While the initial “lockdown” message was clear, the gradual easing of restrictions brought new challenges. [Keating 2021]  By contrast, some women reported a minority of friends or family who did not adhere to the rules, though this did not influence their own social distancing behaviours. Women chose to reaffirm and explain the rules or keep a distance, with one participant reporting losing friends over this. [Jackson 2021b]  A small but meaningful number of respondents had experienced postponed or cancelled hospital appointments for themselves or their baby, which had caused substantial anxiety. [Rhodes 2020]  The fears even extended to the short time spent in a hospital setting during the pandemic. One participant said [Dove-Meadows 2020]  On the contrary, some women delayed seeking medical help due to apprehensions surrounding contracting SARS- CoV-2 [John 2021]  Janis reported that she was afraid that when she went out for her appointments, she would bring germs home which made her fearful of in person visits. Janis stated when she went to the paediatric visit, she did not sit down and she immediately undressed herself and child upon return home and showered herself and bathed the baby. [Spatz & Froh 2021]  Women were particularly hesitant to visit the hospital, or other healthcare clinics, fearing that it was a hotspot for the virus. Fear of COVID-19 was a severe enough that one participant skipped certain postnatal appointments at the hospital. [Stirling Cameron 2021]  Mothers report a heightened stress related to their breastfeeding journey due to the inability to receive in-person lactation support as well as stress related to how COVID-19 could influence themselves or their baby. This stress was common for both new mothers and mothers with multiple children. For instance, one mother stated, [Snyder & Worlton 2021] | **[Reducing risk]** Due to the unknown severity of a COVID-19 infection on the pregnant woman and unborn fetus, many women were recommended by HCPs or taking it upon themselves to take extra precautions to social distance and limit the possibility of transmission. Women shielded at home, or limited movement outside of home, often balancing the moral dilemma, between the risk of contracting the virus in a high-risk environment, such as hospitals and supermarkets, and receiving routine care. Some women with older children mitigated risk of infection prior to births by keeping children from school. This woman described her thoughts on keeping infection transmission to a minimum by reducing the need to meet others, including members. For some women, attending doctors appointments for testing and check-ups was stressful and increased their anxiety about possible infections. Additional stressors included concerns that the virus would be transmitted via breastmilk. (Anderson 2021, Farrell 2021, Sweet 2021, Sweet 2022, Panda 2021, Aydin & Aktas 2021, Linden 2021, Fumagalli 2021, Keating 2021, Spatz & Froh 2021, Snyder & Worlton 2021)  **[Delayed health seeking]** Some women described behaviours that delayed healthcare seeking, such as not wanting to go to see or avoiding doctors and postponing appointments in a hospital setting during their pregnancy, fear of getting the virus. Others described instances in which they were not able to contact health care providers, therefore chose not to engage with health services. In some circumstances, women described being turned away from hospitals and health services due to public health measures, impacting their ability to receive timely care. Women were not able to enter hospitals freely, and were required to delay admission until they were close to birth. (Aydin & Aktas 2021, Farrell 2021, Mizrak Sahin & Kabakci 2021, Sweet 2022, Harrison 2021, John 2021, Panda 2021, Stirling Cameron 2021)  **[COVID-19 influenced health decision-making]** One of the public health restrictions that was enforced in many countries was limiting the presence of support people during labour, with the exception being during active labour. Some women attempted to delay labour inductions to ensure that partners would not be turned away, or have a home birth instead of being admitted to hospital. In similar instances, women reported opting for inductions after an uncomplicated childbirth to ensure that her partner was able to be present for the labour. In addition, due to the lack of contact with healthcare providers, women were late to react to increasing contractions and the need to present to hospitals earlier. In their social networks, women reported the difficulty in managing family and friends that did not adhere to social distancing requirements and felt awkward navigating these conversations. Other feelings of guilt and fear were experienced by some participants as they attended health facilities or as they considered reaching out for help. (Harrison 2021, Jackson 2021a, Silverio 2021, Rice 2021b, Linden 2021, Anderson 2021, Fumagalli 2021, Jackson 2021b, Rhodes 2020, Dove-Meadows 2020) |
| 1.3. Missing out on social opportunities | | |
| Charvat 2021  Reference 1 - 0.34% Coverage  “I think I just feel like we’re not celebrating as much being pregnant. I know that sounds kind of funny, but it almost feels like I’m not pregnant because, I don’t know, not many people are seeing me.”  Davis 2021  Reference 1 - 0.12% Coverage  “It was quite sad that I couldn’t even share my pregnancy experience—as scary as it was, I couldn’t share that with anyone, and I feel like I missed out.”  Dove-Meadows 2020  Reference 1 - 0.35% Coverage  “You know, I couldn’t even have a baby shower because of everything.”  Reference 2  My two-year-old ... he’s not used to being away from me ... so I mean like how’s he gonna handle three days without seeing me?” Plus I’ll be by myself for three days, so ... I mean you got the staff, but it’s not—it’s not your family.  Meaney 2021  Reference 1 - 0.12% Coverage  “Feeling pretty unseen and uncelebrated by most friends and family as we anticipate the arrival of our ﬁrst child, thanks to the pandemic and social isolation.”  Riley 2021  Reference 1 - 0.33% Coverage  “I imagined the last few months to be getting ready with everyone, baby shower and having a bit of a holiday before she arrived”  Reference 2 - 0.14% Coverage  Participants commented on being unable “to go shopping and look at stuﬀ, you always want to go and feel the baby clothes”  Reference 3  “everyone says it take a village to raise a baby and it’s true and we’ve not had our village around us ” (P13, Age 34, PP 7 weeks)  Joy 2020  Reference 1 - 0.48% Coverage  We were supposed to ﬂy to Alberta to see my family at the end of March but had to cancel the trip. This is my family’s ﬁrst grandchild so it just breaks my heart they will miss her whole babyhood. I also feel so alone with the baby. I have nobody here to help me ﬁgure out what is normal or how to progress through these early days. Although people can video chat it isn’t the same. I just want somebody to be in the room with me and the baby to see the things she can do and help me with things.  Stirling Cameron 2021  Reference 1 - 0.28% Coverage  “My kids at home were so upset that they couldn’t visit me at the hospital and see the baby. They had been so excited during my pregnancy saying that they are waiting to visit me in the hospital after I give birth and to hold the baby, they wanted to bring me flowers but of course because of COVID none of that happened.”  Ollivier 2021  Reference 1 - 0.21% Coverage  “I am so sad that having spent years trying to have a baby and now that we ﬁnally do I can’t share her with anyone.[…] This is my family’s ﬁrst grandchild so it just breaks my heart they will miss her whole babyhood.”  Reference 2 - 0.18% Coverage  “My family is missing all of her milestones. […] I don’t have anyone to share the little joys with like her ﬁrst taste of a new food or the ﬁrst time she rolls over. It’s heart breaking.”  Green 2021  Reference 1 - 0.08% Coverage  “I worry that because my family has not been able to see my family, that she will have trouble later on bonding with them”  Reference 2 - 0.10% Coverage  “My daughters (especially my newborn) aren't able to meet her grandparents and if this goes on much longer, I do not know if she will be able to bond with them.”  Panda 2021  Reference 1 - 0.26% Coverage  “…For my husband’s parents it was their very ﬁrst grandchild …But to me I felt like that was the one thing I wanted my family and especially close family and grandparents, to be able to hold him. Because I felt like the journey to get to that stage had been a long time coming.”  Rhodes 2020  Reference 1 - 0.08% Coverage  “I’m due to go back in November, but I am thinking of taking the full 12 months as I haven’t been able to do stuff with her.”  Kolker 2021  Reference 1  And it’s everybody’s first grandkid so they’re really definitely sad about missing out on this time… It just feels like they’re just babies for such a short time and … I don’t know, I feel like because we got to see what it was like before the lockdown, just seeing how much joy she brings everyone and how happy everybody is, and having them not be able to see her just really sucks. [P1]  Saleh 2022  Reference 1  “It’s just kind of sad. You want grandma, grandpa, everybody to be able to hold your child and just love on them ... I feel like now it’s almost like you’re living in fear.”  Brown & Shenker 2021  Reference 1  “My mother is wonderful and a huge supporter of breastfeeding. I was really looking forward to her coming to visit after my baby was here. She cannot come and whilst we can video message it's just not the same as having your mum close by. I feel I need her, not just to help but emotionally and I'm struggling without this support. It makes everything feel so much harder.”  Jackson 2021a  Reference 1  “I think week two to four was peak tiredness and then that’s the point where I’d really loved either my mum, my mother-in-law, or my own family to sort of step in and be able to help out a bit more.” (Respondent 10, T1).  Reference 2  “It’s [new motherhood] just been sadness, really…The people who you are close to and would usually rely on as most forms of support can’t be part of the things that’re a massive deal to me…he [baby] turned his head for a noise or, or when I’m singing a certain song he does this really cute thing…it’s like, “Aw I’ll just go to my mum’s and show – Oh, no. I can’t”. It’s the realisation, isn’t it?”  Reference 3  “There’s so many things that I feel like we’ve missed out on in terms of, you know, him [baby] meeting his family and…other than immediate family, no one’s even met him, who would have by now…and at this point in time we don’t know when they will either.”  Keating 2021  Reference 1  “It would have been nice to meet other mums who were due their baby at the same time. So all that social element was totally gone”[P10]  Reference 2  “every week my family are like “aw send us a picture of your bump”” P12  Sweet 2021  Reference 1  “It was really rough for the ﬁrst few weeks –my parents live a few hours away so they didn’t meet him until he was 6 weeks old, and the plan had originally been that mum would come up and support us, obviously that didn’t happen”  Sweet 2022  Reference 1  “the only visitors I wanted was the girls [other children] to meet their sibling, so they couldn’t do that”.  Mizrak Sahin & Kabakci 2021  Reference 1  'To be home alone during the day, I would normally go to my family, but I mean, in the process, everyone is staying at home . . . and no one can come to our place because of the virus, you know, because of my pregnancy. I'm on my own.' (P1) ‘My family would visit me as they live close, and I normally go every three or four weeks, it has been almost two and a half and three months since I went, which affects me.’ (P15) ‘I am alone now, and there will be no one at birth. My mother would support me but she stayed there in Istanbul, and the bus service was stopped.’ (P9)  Snyder & Worlton 2021  Reference 1  ‘‘you can’t really go anywhere, it’s one thing to talk over the phone or on the computer it’s just more difficult that way’’ | Many of the participants highlighted the events cancelled or altered (e.g., baby showers), missing the fun of “showing off” their pregnancy, and mourning the loss of the family connections usually made during a pregnancy (e.g., mom visiting after the baby is born). Grace explained how she struggled with missing out on the pregnancy. [Charvat 2021]  Frequently women from both the high and the low groups mentioned their feelings of isolation, particularly from family but also from friends and peers. [Davis 2021]  The participants described this loss of rituals not only  in terms of general infant preparation, but also in terms of how the infant shower represented the opportunity to celebrate the new infant and mourning that this was no longer possible. [Dove-Meadows 2021]  During the pandemic, pregnant women relied heavily on informal supports reporting that they depended on their partner, family and friends to help alleviate stress. However, as illustrated below, women reported how they felt that this support was limited. [Meaney 2021]  The ﬁnal few months of pregnancy were not as anticipated for participants and several spoke about missing out on ‘typical’ pregnancy related activities. [Riley 2021]  This mother believed that sharing the experiences of having a new baby was important and felt sadness that she was not able to do this on account of the public health orders in place. Feeling ‘so sad’ revealed the meaning this experience held for this mother and that it was very signiﬁcant. This was further emphasized as the participant gave more context to her experiences. [Joy 2020]  Other participants were looking forward to enjoying more intimate moments with their husbands and children, such as having their children visit in the hospital, which was not possible because of COVID-19, leaving women and their families disappointed [Stirling Cameron 2021]  Socialization between babies and extended family was also considered to be important for babies’ development. Two participants explained what it meant to them to share their baby with family members. [Ollivier 2021]  Three postpartum women reported concern that their infants would be unable to meet important loved one's which would affect the bond that they would later develop… Family/loved ones missing out [1 (4%), 3 (6.8%)]. Worries in this category were related to concern that others would miss out on pregnancy and postpartum milestones. Specifically, one pregnant woman reported concern about not being able to have a baby shower/ celebration welcoming the baby. [Green 2021]  Social distancing, COVID-19 restrictions, and concern about elderly or vulnerable family members meant that many women did not have opportunities to introduce their baby to loved ones that they might have otherwise done had the pandemic not existed and which they would have looked forward to. [Panda 2021]  The sense of missing out was expressed much more strongly by postnatal respondents who felt that they were being deprived of all the fun activities they had looked forward to doing during maternity leave, such as play dates, baby classes, and seeing more of family and friends. Some respondents thought a UK-wide extension to maternity leave was needed, while those who were able to extend their maternity leave were considering doing so. [Rhodes 2020]  Participants expressed challenges bonding with the baby due to fears and anxieties generated from the pandemic as well as sadness that baby bonding with family would be delayed. “We had a name for [older son], but this baby, we didn’t have a name until like two weeks after it was born and we didn’t even engage ourselves with baby names and talking about names… so we were kind of scared to even make the plans…I would have wanted to do shopping for the new baby. That didn’t happen. I cried all the way to (the hospital).” [Kolker 2021]  Expectations vs reality [Saleh 2022]  Additionally, in contrast to those who felt shielded from negative family interference, others felt isolated and missed the emotional support they would receive from caring and supportive relatives. [Brown & Shenker 2021]  Most respondents felt the initial lockdown was extremely isolating, due to restrictions on between-household socialising, and fears of spreading and contracting COVID-19. This was especially difficult during the early postnatal period whereby women would have otherwise been receiving much needed emotional and practical support from friends and family during this transition to parenthood… Lost postnatal experience For women who had had their babies during initial set of lockdown restrictions, it was not uncommon for mothers to express sadness concerning the lost opportunity for extended family to bond with the new baby, and the inability to share infant milestones with family… There was a commonly reported sense of sadness in connection with family and friends having lost irretrievable, precious time with their infants [Jackson 2021a]  Women described the “new normal” of a pregnancy during a pandemic. They found it difficult to engage with other pregnant women which added to a sense of isolation. Some felt there was less excitement surrounding the pregnancy. [Keating 2021]  The government-imposed social restrictions impacted on participants’ experience in the home, with reduced visitors and loss of support further exacerbating the sense of aloneness. [Sweet 2021]  All participants who gave birth in a hospital experienced the visitor restrictions on the wards. [Sweet 2022]  Although modern technology enables video calls with family and friends, the respondents, especially those with young babies, felt that they were suffering considerably from the loss of face-to-face interaction. Without antenatal and postnatal groups and visits to family and friends, respondents felt that they were missing out on opportunities to share experiences and learn from others. [Rhodes 2020]  All mothers reported grief and sadness related to social distancing. They reported planning to have their family members involved in their child’s life and helping with newborn and household duties during the immediate postpartum time at home. Two mothers in particular talked about friends and families making window or door visits. Janis said these visits made her cry and she told friends and family members to stop coming because it just made me feel worse. Stella described glass door visits as being heartbreaking. [Spatz & Froh 2021]  Multiparous women reported the difﬁculties in maintaining and managing the relationship with other kids at home, especially in the case of young children. The required prolonged separation ampliﬁed the common worries in case of a sibling’s birth, especially when the sudden hospitalisation was unexpected and not accompanied by any explanation or goodbye from the mothers to the children. Not being able to see other kids increased the women’s sense of loneliness [Fumagilli 2021]  Family support is important in pregnancy and postpartum process in Turkey, and usually the mother of the pregnant woman lives with her in this process and helps her to make the process more comfortable. However, due to the pandemic, the families of many pregnant women were unable to live with them due to national measures such as travel restrictions, the ban of going out of for people over the age of 65, and the process was more difﬁcult for these pregnant women [Mizrak Sahin & Kabakci 2021]  Related to emotional support, husbands and boyfriends were the most common current source of emotional support through in-person listening followed by family members providing support telephonically or over the Internet. Almost all mothers reported they were unable to see family and friends in-person to the extent they desired and wanted more in-person opportunities for emotional support. [Snyder & Worlton 2021] | **[Missing out on traditions]** Typical traditions that women missed celebrating included baby showers, and enjoying holidays before the birth of the baby. Additionally, not being able to physically enter shops to purchase baby items and clothes was also missed by women. Women mourned the loss of these rituals and their indication that an infant was to be expected and celebrated. Postnatal women felt that they had missed opportunities to do fun activities with their babies, such as play dates through parents groups, classes and seeing more of family and friends (Charvat 2021, Dove-Meadows 2021, Riley 2021, Keating 2021, Meaney 2021)  **[Missing out on interacting with family and friends]** Many women described the distress and sadness in not being able to share their pregnancy experience, labour and postnatal period with close family and friends. The inability to be physically close to others, was felt immensely by majority of mothers, family members not being able to see mothers experience their pregnancy, support mothers when needed, or interact with the newborn due to public health restrictions and social distancing measures. For women who could not see family and friends on the postnatal ward, they reflected on the missed opportunity to bond, and said the care they received was adequate, but not the same as family. Families who stated that their newborns were their family’s first grandchild found it especially difficult to navigate public health restrictions and social distancing. In addition, mothers with older children felt they missed the opportunity for siblings to interact with the newborn during the early postnatal period whilst mothers were still in hospital. Family members missed out on the opportunity to bond with the new families, and newborns and this was expressed throughout all papers. (Charvat 2021, Meaney 2021, Joy 2021, Stirling Cameron 2021, Ollivier 2021, Green 2021, Panda 2021, Kolker 2021, Saleh 2022, Brown & Shenker 2021, Jackson 2021a, Keating 2021, Sweet 2021, Sweet 2022, Mizrak Sahin & Kabakci 2021, Rhodes 2020, Spatz & Froh 2021, Davis 2021, Snyder & Worlton 2021) |
| 1.4. Breastfeeding challenges and triumphs | | |
| Sweet 2022  Reference 1 - 0.16% Coverage  “Breastfeeding is really tough … lactation consultants weren’t able to do business which makes it harder”  Harrison 2021  Reference 1 - 0.06% Coverage  "I was completely clueless on so many things that I was heavily relying to learn about in my classes. When it came time for breastfeeding, I had no idea what to do or any challenges that could come. There were so, so, so many questions and I felt so confused during everything.”  Jackson 2021b  Reference 1 - 0.43% Coverage  “I think I’m going to give up breastfeeding probably sooner than I would’ve done…because I’m knackered [laughter] erm…in-in all honesty… just ‘cause I just feel like without the support in the day and without being able to get out and about and see, you know, see my family and stuff, I think it’s…the breastfeeding is getting a little relentless. Erm so yeah I think co- in the sense that, because of, because of lockdown erm- because of COVID, I’m prone to not breastfeed as much.”  Reference 2 - 0.22% Coverage  “There’s no right or wrong way. You know, at the end of the day the ultimate goal is that my baby needs to be fed. End of. Erm you know, feed him breast milk. Breast milk, er formula. He’s fed. He’s happy. Sweet. That’s done. Job done! You know what I mean? The important thing is actually be kind to yourself, you know?”  Reference 2 - 0.51% Coverage  “Yeah – actually we- so thinking that breastfeeding rates are gonna go up a bit ‘cause people aren’t feeling as much pressure well, ‘cause they can’t go out. So, they’re staying at home and they’re just feeding. However, I think that’ll only be true for people who aren’t really struggling because, like, some of them will hopefully seek help and get it and then be able to persevere. But I think it’ll take quite a high level of resilience and want for those ones who are really struggling, because, yeah, it is-it can be very difficult.”  Reference 4 - 0.34% Coverage  “I’ve got the baby to feed so often and just been, you know, sat there doing nothing else but feed the baby at times so [husband] does everything else. If he wasn’t there, going back to work and things like that when the kids are back to school- there’d’ve been no way for me to carry on [with breastfeeding] ‘cause it was taking so much of my time.”  Reference 1 - 0.07% Coverage  “One good thing about lockdown is that had all the time in the world to be able to breastfeed, get it right  Brown & Shenker 2021  Reference 1 - 0.24% Coverage  “I've found breastfeeding quite difficult due to problems with latching, nipple tears/bleeding and lots of pain. Being able to stay home and concentrate on getting the feeding right is the only reason I persisted. If I'd had lots of visitors or pressure to meet other mums/family/friends, I'm not sure I would have managed as I've only just got the hang of feeding 5 weeks in!”  Reference 2 - 0.17% Coverage  “Newborn lost a lot of weight due to tongue tie and bad latch. Breast feeding class cancelled due to COVID. Husband not permitted in hospital when breast feeding advice was given and I was recovering from giving birth so struggled to take in information. When midwife identified low weight, we were put on a feeding plan with formula, and I was advised I may not be able to breast feed. I expressed a lot to ensure I could build up my supply and had very sore nipples. After contacting 111 we thought I had thrush, and I was given cream. Turns out I had bad positioning which was identified via video call two weeks post birth. Face‐to‐face support e.g. somebody physically helping you to position and latch your baby is far more effective than a zoom video call on a mobile device.”  Reference 4 - 0.27% Coverage  “We are unable to have face‐to‐face support to help me to feed my baby who is struggling to gain weight due to possible tongue tie which is unable to be treated. Because of the pressure to have him gain weight or be admitted to hospital and having little support with improving his latch and expressing milk I have had to top up with formula which is something I have not wanted to do and did not need to do with my first child.”  Reference 1 - 0.21% Coverage  “Being able to go to clinics/clubs and get used to feeding in public with other like‐minded mum's or getting help with positioning in different situations is what is missing. This overall makes me feel like I would stop breast feeding sooner than planned as once not confined to my home I do not know how to do it with confidence.”  Reference 2 - 0.27% Coverage  I was inundated with visitors with my first child and often could not feed responsively due to their discomfort with feeding or them wanting to comfort my daughter when she was upset. She had poor growth and I felt enormous pressure from my in laws in particular to supplement with formula. With my second child, there is none of that pressure and I can really see an enormous difference both is his feeding and in my mental health  Rhodes 2020  Reference 1 - 0.11% Coverage  “I was struggling breastfeeding. I would have gone to breastfeeding group, but that’s been cancelled…. I was in pain and I felt let down, but I felt really guilty asking for help.”  Reference 1 - 0.13% Coverage  “Feeding is a challenge at the moment… it’s been painful. I was engorged and he was crying, and I was upset that he was upset, so my partner went to get some formula, but Tesco’s isn’t 24 hours anymore.”  Silverio 2021  Reference 1 - 0.25% Coverage  “I was also referred by my health visitor for a breastfeeding Zoom call. That was ridiculous. I needed to see someone face-to-face because they have to check your position, your latch and whether your baby has tongue tie. Feeding support has to be there face-to-face and it needs to be available”  Rice 2021a  Reference 1 - 0.58% Coverage  “I didn’t cope [with the lack of support person] very well. The second night was horrific. I cried pretty much the entire night. I was trying to breastfeed but it wasn’t working, so after the second night, I basically decided to formula feed [the baby]. And you can’t even really see anyone’s faces because they were all wearing masks. You didn’t really know ... who you were talking to. My problem was I couldn’t hear what they were saying because I couldn’t lip read.” (Hard of hearing mother)  Reference 2 - 0.63% Coverage  “I’ve noticed a lot of people are quitting breastfeeding [because of] lack of support during the pandemic. … I tried [to breastfeed] for a couple of weeks, but I couldn’t get a lactation consultant, or I never had a visit from Public Health, or they’re in the city. When you go to the [breastfeeding] clinics, maybe you realize, “Oh, wow, everybody has pain when they first start to nurse” or “Everybody feels this way!” But when you’re a first-time mom, home, with people technically not allowed to visit....”  Reference 1 - 0.40% Coverage  I had a couple of virtual appointments [with lactation consultants], which I found totally useless. I was so desperate to make it work because I had such a wonderful experience nursing my first kid and I was ... devastated that it wasn’t working with [the new baby] because it was something I was really looking forward to.  Riley 2021  Reference 1 - 0.18% Coverage  Breastfeeding services attempted to support new mothers in other ways through “zoom calls ”, but “having someone look at it through a camera…just didn’t cut it”  Reference 1 - 0.24% Coverage  “Yet for a minority of participants, isolation from others was considered key “to the success that [they] have had with breastfeeding this time…there has been nowhere to go to, no one to see, no one coming here”  Reference 2  “The fact they were like no, if you want to breastfeed you will have to stay in and the night shift can see if we can get you breastfeeding, I was like no, not another night without visitors or my husband… I just said I am going to give him a bottle, let me give him a bottle, and let me go home with my husband…then I just ended up formula feeding which I am ﬁne with”  Joy 2020  Reference 1 - 0.33% Coverage  It’s been great . . . we have this opportunity to bond as a family and he [partner] is here for every moment during the newborn stage! It has been amazing not having to worry about visitors coming and going and cleaning out home and me worrying about breastfeeding in front of others - instead we have a very relaxed atmosphere for everything!  Panda 2021  Reference 2 - 0.24% Coverage  “I was able to breastfeed without feeling really exposed and thinking about it now I think if there had been people around, strangers marching in and out of the ward I would have had the curtains pulled all the time and I wouldn’t have put my foot outside you know.”  Reference 3  “I do remember like we had the breastfeeding groups [after a previous birth]. That I would’ve went to, like weekly [facilitated by local PHNs]. And that was great because you’d be able to get the baby weighed… just reassurance that you were on track in terms of weight gain… That is deﬁnitely something that, you know, I mean COVID has taken away” (ID 05)  Reference 4  “The lactation consultant…is more important with COVID. Because you don’t have your mum or your granny…around you…to…help and correct you, you’re on your own. It’s hard to…zoom…with private lactation consultants.” (ID 03)  Reference 5  “I was a bit concerned about the latch and all that stuﬀ…… so I was really just doing that all, that was just me on my own trying to work all that stuﬀ out. So that was diﬃcult like.” (ID 08)  Spatz & Froh 2021  Reference 1 - 0.24% Coverage  They reported that having to be home was a plus for breastfeeding. Stella stated you can just be at home and only in a bra all the time.  Reference 2 - 0.15% Coverage  “and stayed at home, there was nothing else to do but just breastfeed all of the time.”  Kolker 2021  Reference 1  “We don’t have breastfeeding classes to go to and you don’t have in person… I was going to have a lactation consultant come to my home … I did it, virtually, once or twice and it was awkward and hard. I wanted someone right there with me. It’s just such a personal thing and they need to be right there to see so closely with the baby.” [P5]  Ollivier 2021  Reference 1  “We had one meeting with public health before they reassigned our nurse to the COVID team and we were dumped. The breastfeeding clinic cut her tongue tie but did a phone follow up instead of in person due to COVID and dumped us even though we were still having feeding issues. (Participant #8)”  Costa 2021  Reference 1  “I think (my son) would have been assessed a bit more by the cleft nurses had they been able to do home visits… I personally would have been happier with a lot more physical support with feeding. Especially in the early weeks… because it is really different from feeding a child without a cleft.”  Snyder & Worlton 2021  Reference 1  “I’ve been wanting to, like, talk to a lactation consultant again but,  like, with the pandemic it’s been so hard because you know like  everything, everybody, is obviously shut down and it’s just like I  want to be able to talk to someone but I can’t. Like it would be  easier to have someone be there and physically be like right now  try moving him this way now try this way like” (23, Caucasian,  unemployed)  Reference 2  ‘‘I just feel really isolated so that kind of discourages me from breastfeeding because I’m just so exhausted because like I have formula to make his food I could just pour him some formula and like be done even though I know that’s not what’s best for him’’ (23, Caucasian, unemployed).  Reference 3  ‘‘It was hard for me to know if it was because of like COVID but  I really didn’t get help at all at the hospital. She did latch on  pretty easily but no one came in to help or you know anything  like that. I never got any support at the hospital’’ (31, Caucasian,  teacher).  Reference 4  “To be very honest the pandemic really started at the end of my  maternity leave and so it’s been somewhat of a blessing in disguise because my job allowed me to work from home and so it just feels like it’s been a maternity leave and I feel like it’s given me a lot of time to be home and to be at a slow pace and have a longer period of time to figure out you know my baby and nursing has become so much more easier and I think it helped just having so much time just to practice instead of having to figure it out in the workplace at such a quicker pace and so I’ve honestly enjoyed the time that I’ve had at home these last few months just because of that” (27, Caucasian, Church Director).  Reference 5  ‘‘it’s impacted me personally for the better just because I haven’t  been able to return back to work and I’ve been able to breastfeed on demand which is easier than pumping and trying to come up with milk to feed’’ (37, Caucasian, unemployed).  Reference 6  “I think you know being able to put him to breast as often as I  can to maintain that supply so we didn’t have to leave the  house. I think that’s just kind of what kept me going you know  like people were really struggling to find formula there for a  couple weeks” (31, Caucasian, nurse).  Reference 7  ‘‘If this was my first baby I definitely think it would’ve been a lot harder and just with the whole pandemic in general. I mean it’s been challenging because of the pandemic but it would’ve been a lot more challenging if it would have been our first baby’’ (31, Caucasian, teacher).  Reference 8  ‘‘With my other one, having that support and weekly consultation with a lactation consultant and everything was really what probably got me through our journey and kept me nursing um if I would have had that problem with my one now and not having that support then I we probably would’ve stopped by now to be honest’’ (35, Caucasian, physical therapist) | Inadequate access to postnatal care was raised by all the participants who were postpartum at the time of the interview. In-home postnatal visits were minimal or not offered at all, and postnatal services such as maternal, child and family health visits or new parent classes were, in many cases, cancelled as deemed non-essential services, which disappointed participants.  Lactation consultants were another service deemed non-essential in some states. [Sweet 2022]  Some first-time mothers whose antenatal classes had been cancelled were disappointed that staff were not willing to educate them about baby care and breastfeeding. [Harrison 2021]  Additional parenting responsibilities and restrictions  on social support in light of schools and breastfeeding support groups being closed was also a noted breastfeeding barrier… For such women it was deemed essential to stay kind to yourself… Breastfeeding practice was reportedly easier for  some mothers due to reduced visitations, allowing women to more easily persevere and response-feed their babies… One participant, who self-disclosed that she was a breastfeeding peer support worker, noted the pivotal role of breastfeeding confidence and healthcare professional support in determining breastfeeding success during the COVID-19 pandemic… Having one’s partner at home was an invaluable sup-  port for sharing parenting responsibilities, which allowed women to persist with breastfeeding practice  [Jackson 2021b]  [Neg] Many participants talked about a slower pace of life and nowhere to go or need to be having a positive impact on how much time they could spend focusing on feeding their baby. This was particularly helpful for some mothers who were struggling with issues such as latch. They believed that if they had been pressured to be out and about meeting people, they would still have been struggling or in pain and would have stopped breastfeeding… The most common disadvantage of the lockdown was a lack of face to‐face breastfeeding support when mothers had difficulties. Some reported having to describe issues over the phone or from across a room, feeling that their health professional did not want to come near them. For issues such as latch, mothers really missed having someone who could look at what was happening up close and support them to make small changes.  [Pos] Many participants talked about a slower pace of life and nowhere to go or need to be having a positive impact on how much time they could spend focusing on feeding their baby. This was particularly helpful for some mothers who were struggling with issues such as latch. They believed that if they had been pressured to be out and about meeting people, they would still have been struggling or in pain and would have stopped breastfeeding. For some mothers, fewer visitors meant that they were more relaxed and had more time to focus on their baby and their own recovery rather than hosting a stream of people wanting to see their baby. For others, it meant fewer unwanted comments. This helped get breastfeeding off to a much better start.  [Brown & Shenker 2021]  Although some respondents had been able to speak to their health visitor on the telephone, others reported having no health visitor service at all. Concerns about the baby’s weight gain were frequently voiced, as were breastfeeding issues by those who had given birth just before or during lockdown. [Rhodes 2021]  However, where accessibility was not an issue, virtual care was often discussed as an inappropriate medium through which to conduct health checks, especially postnatally, during which time both newborns and new mothers required attention. [Silverio 2021]  Lack of support in hospital and at home influenced some women to stop breastfeeding sooner than they had wished to (Box 4). Online breastfeeding supports were uniformly experienced as unhelpful. The participant felt they had to make a choice between staying at the hospital alone to receive breastfeeding support or to go home with their partner and choose to use formula. Participants also talked about their struggles to obtain breastfeeding support upon returning home due to COVID-19 restrictions and their subsequent isolation from health care professionals. [Rice 2021a]  Similar to the participants above, many of the participants in this study, found the period during  the COVID-19 pandemic to be a time for quiet enjoyment of their baby and their new family as they were required to stay at home. Using the lens of feminist poststructuralism, we can see how these mothers were challenging social norms and expectations of new mothers. Challenging social ideals was a way of using their agency as they clearly articulated their beliefs about how being at home with their babies was very positive and for some a ‘blessing’. Many participants believed that this was a time for more personal and intimate family enjoyment. A time where the immediate family could not only enjoy each other but also their new baby. [Joy 2020]  Being alone in the hospital became too much for one participant… The participant felt they had to make a choice between staying at the hospital alone to receive breastfeeding support or to go home with their partner and choose to use formula. [Riley 2021]  Many hospitals also transitioned to virtual post-birth appointments. Some participants discussed how having virtual meetings with their lactation consultant prevented them from receiving the best care for their child. For example, Gina was having trouble with her baby latching and getting her breast in the right position. She discussed feeling overwhelmed with the process of learning how to breastfeed, especially because she was not given hands-on assistance, and crying due to her frustration. Although the changes made by medical institutions were intended to prevent illness and coronavirus exposure, they also reduced the participants’ access to medical care and information. [Chavarat 2021]  Other participants had planned to attend a hospital breastfeeding class or in-home breastfeeding support following delivery with either a lactation consultant or a midwife, however these too were cancelled due to pandemic restrictions. This left several of the first time parents with many questions regarding breastfeeding with very few opportunities for support. [Kolker 2021]  Public health orders during COVID-19 made accessing information and support very diﬃcult for mothers in our study. Many participants said they were concerned that they did not have the right information about how to best care for their baby, including how to safely breastfeed. With unclear information, mixed messaging, and unknown reliability of certain information sources, parents had to make diﬃcult decisions regarding what sources and who to trust when it came to caring for their new baby. The issue of navigating shifting resources is exempliﬁed by one participant, who stated: [Ollivier 2021]  In particular, women who were breastfeeding narrated feeling more relaxed, comfortable and less exposed as a result of the visiting restrictions in the hospital… Many women talked about their diﬃculty in accessing support for breastfeeding, which was ‘daunting’ and ‘frustrating’ for women. Women described being sent breastfeeding booklets from the hospital and being directed to websites for further information. For some in rural areas this type of information was ‘useless’ as their internet connections were unstable. Mother and baby groups after birth, many of which are facilitated by PHNs who provide infant feeding support also, were now unavailable to women who would have considered joining these... A few women described having video consultations with a private lactation consultant, although many described these as being insuﬃcient and inappropriate to meet their needs. The lack of breastfeeding support and directed advice on where to go if experiencing breastfeeding issues was recounted by women as being stressful and frustrating, with women having to work through many of the issues on their own [Panda 2021]  Only one of the mothers had the opportunity to see an International Board-Certiﬁed Lactation Consultant (IBCLC) because she gave birth at the beginning of the pandemic before social distancing guidance became the standard. Janis had multiple telehealth visits for breastfeeding assistance and she liked the experience because she did not have to leave the house. Although Stella said the telehealth visit for lactation was awkward because her partner had to hold the computer at an angle for the IBCLC to see the latch. She was not conﬁdent that the visit was accurate or effective. [Spatz & Froh 2021]  Most participants, however, reported reduced (or no) in-person support from the CL/P team, particularly with regard to feeding [Costa 2021]  Some mothers were uninterested in using telelactation. For instance… Most mothers reported being unsatisfied with the amount of appraisal support they received… Support networks decreased from the time of delivery with mothers reporting a decrease in lactation support within the hospital… More than half of mothers reported that the pandemic increased the duration of their maternity leave, which they credited with positively influencing their breastfeeding journey… In addition, some mothers credited the early fear of formula shortages as their motivation for continuing to breastfeed… The majority of mothers had more than one child (n = 19). Many mothers believed that if the pandemic had occurred during their first breastfeeding journey they would have experienced even more challenges. [Snyder & Worlton 2021] | **Challenges:**  **[Difficulties in establishing breastfeeding with limited HCP support]** For some women, they did not re receive in-person lactation consultations post-birth and felt that the decrease in support made them feel frustrated and lonely. Many women felt that online “Zoom” or telehealth sessions with lactation consultants were an inappropriate medium and “useless” in providing helpful and supportive breastfeeding advice. Sometimes there was limited information in where to access these platforms, and if accessible, many were disappointed in the lack of F2F care. This left women feeling confused and abandoned. Women requesting lactation services demanded more F2F care, to see what was happening up close, and to support mothers make small changes to improve their breastfeeding experience. Multiparous women showed empathy towards first-time mothers, sharing the difficult in establishing breastfeeding without additional support. (Sweet 2022, Harrison 2021, Brown 2021, Rhodes 2021, Silverio 2021, Rice 2021a, Riley 2021, Spatz & Froh 2021, Charvat 2021, Kolker 2021, Ollivier 2021, Panda 2021, Costa 2021, Snyder & Worlton 2021)  **[Early cessation]** Breastfeeding during the pandemic was challenging for some women; the limited access to breastfeeding support both from professionals and family members reduced confidence and meant that they would cease breastfeeding much earlier than anticipated. Some women felt that due to lockdowns, and being confined to home, they felt over-burdened by breastfeeding, with some suggesting that they are overwhelmed with the constant feeding. As a result they have turned to formula feeding instead. Others felt the pressure of returning to work contributed to early cessation. (Jackson 2021b, Brown 2021, Rice 2021a, Riley 2021, Rhodes 2020, Snyder & Worlton 2021)  **Triumphs**  **[Undisrupted time to establish breastfeeding]** Due to the restrictions placed on movement, women spent more time at home, with fewer visitors and obligations to visit others. This meant that women could establish a routine of on-demand feeding with their newborns, in the comfort of their own homes. Women also attributed their successful breastfeeding to reduced and flexible work arrangements, more support from partners working from home, as well as being able to relax in a calmer environment with just their immediate families. For women that accessed online lactation support, some valued this the mother did not have to leave the house. (Jackson 2021b, Brown & Shenker 2021, Riley 2021, Joy 2020, Panda 2021, Spatz & Froh 2021, Snyder & Worlton 2021)  **(Results paragraph [Being kind to yourself]** Some women recognised that breastfeeding itself was a stressful task, in addition to living in a pandemic where support was limited, that women needed to be less harsh on themselves if breastfeeding was not working out. Being kind to oneself was encouraged by women, to reduce the stressors of unplanned early cessation. (Jackson 2021b) |
| Navigating changing health policies2.1. Birthing experience filled with uncertainty and unknowns | | |
| Atmuri 2021  Reference 1 - 0.21% Coverage  “I wish that I knew, had a picture in my head, of what I was going to be walking into . . . I guess there’s a little bit of anxiety about getting lost and just, yeah, the idea of not knowing is a little  disappointing”  Reference 2 - 0.15% Coverage  “I’d like to have a water birth and I don’t know if that’s still possible. I don’t know if water births are allowed again? Or being in the water during labour?”  Reference 3  ““I was a little bit worried about being sent home early . . . because this is my ﬁrst baby. I’m also going home on my own . . . I don’t have a partner to help me or to help me look after me or the baby or anything . . . and even just breastfeeding - so that was a bit of a worry.”  Reference 4  “What if something happens with me not straight away but it could be a lot of medical challenges and obviously I don’t want newborn to be exposed to situation where it may get affected.”  Meaney 2021  Reference 1  “The unknown about what it will look like in January when I give birth. Hoping my husband and mom can be there. Also worried if I were to have the virus they would keep the baby from me. Just a lot of unknowns, especially with pregnant women being in the high-risk category now.”  Fumagalli 2021  Reference 1 - 0.28% Coverage  “I was expecting to get to the end of pregnancy, relax and sort out the last few bits for the baby’s arrival and I found myself locked at home instead.”  Reference 2 - 0.05% Coverage  “Deﬁnitely the wrong time to be pregnant . . . a difﬁcult time.”  Reference 1 - 0.34% Coverage  “When I tested positive everyone was still unsure of what would happen or how to approach the disease. Everyone’s uncertainty worried me a lot, especially the one of healthcare professionals.  Potential vertical transmission and safety measures in case of COVID were still a doubt”  Harrison 2021  Reference 1 - 0.04% Coverage  “My biggest fear that caused me a lot of anxiety and depression was whether I would have to give birth alone … I didn’t even know until the day whether my partner was allowed in.”  Reference 2 - 0.03% Coverage  “We chose to try for a home birth to avoid going into hospital and to ensure my partner would be present for the whole birth process.”  Reference 3 - 0.03% Coverage  “Due to Covid my husband was unable to be with me in the induction ward, so I decided to go ahead with a c-section to get the baby out and go home with him.”  Reference 4 - 0.13% Coverage  “Because I have a history of quick births, the midwife advised I should consider a home birth. After the pandemic started I was informed my local hospital’s maternity services were closed and I couldn't have a home birth, and that I had to go to a hospital 40 minutes drive away (I don’t own a car). This made my pregnancy very difficult with the worrying how I'd get there, what if baby came too fast again (he did), would my partner get to be with me. As I suffer from an anxiety disorder and OCD [obsessive compulsive disorder] I really struggled with the change to my plans and the not knowing what to expect.”  Reference 5  “The midwives on the Postnatal Ward were so unkind that I ended up discharging myself. Two days later I was so unwell, I had to go to A+E and stay in hospital for 3 days with my baby. Had my birth partner been allowed to stay this wouldn't have happened.”  Silverio 2021  Reference 1 - 0.46% Coverage  “…I was kind of calling my Midwife… on almost a daily basis [laughs] leading up to my labour, just because things were changing and you were hearing things in the news so regularly, and I was concerned that I could go into labour tomorrow and not know what to expect. So yes, it was conﬁrmed that I wouldn’t be able to have my husband with me until established labour, which I think did impact my labour quite dramatically.”  Reference 2 - 0.76% Coverage  “…at 34 weeks I had a telephone appointment and I tried to ask what changes are there in the hospital, because of COVID and talk about the birth plan. She basically said, ‘Everything is changing so quickly there is no point in us even talking about that now. Wait until your next appointment.’ My next appointment wasn’t until 38 weeks and I remember thinking that was really strange because I could technically have had the baby by 38 weeks. It felt like I was being brushed oﬀ a bit and that made me quite anxious because it felt like I hadn’t had the chance to have that conversation. At 38 weeks I had a face to face appointment and that was supposed to be where we talk about the birth plan, but when she checked for the baby’s heart rate, she said it was a bit faster than it should be. She sent me straight to the assessment unit again, so we never had the chance to talk about the birth plan.”  Reference 3 - 0.26% Coverage  “It was really abrupt. They deﬁnitely said, ‘Your choices are now limited. You are choosing between not the scenarios you wanted, but this is where we are, and you need to get it together.’ … There was a deﬁnite ‘This is a crisis, take the choices you have on the table, don’t complain about it’, attitude.”  Sweet 2022  Reference 1 - 0.15% Coverage  “that was kind of the final straw when … they said that I wasn’t able to use gas because that had been part of my plan for so much of my pregnancy. … I remember being really upset when I read that”.  Reference 2 - 0.10% Coverage  “the biggest worry I had of being pregnant in COVID was for whatever reason some [one] decided you can’t have gas in labour”…. “like you know they’re telling us that we’re not allowed to use the gas … like it definitely put a sort of fear factor towards the end for me”.  Reference 2 - 0.34% Coverage  “because everything was so busy and the midwives and doctors were so unsure of what was going on and they hadn’t been given a lot of information I could sense that everyone was a little bit stressed and a bit like – ‘well we don’t know what’s going on’ they weren’t outwardly trying to show that, you could sense that everyone was a little bit stressed, so it wasn’t a nice feeling – it kind of made me feel a little bit like – not safe”  Reference 3  “My sister’s godmother is actually a … midwife, so when this all started … if things were going to get worse and the hospitals are going to shut down … I did have a chat to her that if we have to go homebirth, I need her to come over. But we’d never planned for homebirth before that”.  Rice 2021b  Reference 1 - 0.28% Coverage  “Given the lack of availability of any other kind of pain management, I had to have an epidural. I’m grateful that exists, it’s just not what I had hoped for. I had hoped to use other types of measures (…). I’d hoped to sit in a tub with warm water and take showers and be able to walk up and down the hall. But you’re confined to your room, so you can’t really walk around.”  Reference 2 - 1.29% Coverage  I sometimes wonder if [the obstetricians] were like, “Okay he’s big enough, he’s healthy, we could just put her up in a room but we could also just take the baby out now.” (…) There were discussions amongst the nurses, and people were talking about it in the hospital that things were going to change quickly. As soon as we got home things started to lockdown, borders started closing. So, preparations were already being made as to how us as a community and the medical community would deal with this pandemic. So, I’ve wondered… I say, “emergency C-section,” it wasn’t an emergency like if they don’t get him out, he’s not going to survive and there was no harm to me either. They were like, “Okay, we could take him now or we could wait, but we might as well just take him now, we know he’s big.” I sometimes wonder if that was a consideration because they knew things were about to change. As implied in the account above, Olivia suspected that she was urged to choose a caesarean quickly as a means of controlling workflow in the hospital, in anticipation of an upsurge in patients due to COVID-19. While Olivia reflected that she would likely have had a caesarean eventually due to complications with her pregnancy, she expressed regret that she “Wasn’t given a choice.” Her tone was wistful when she remarked: “Maybe I didn’t question it enough. COVID was probably on my mind too at that point. I had put my faith in the doctors and the surgeons.”  Reference 3  “It felt a little like they were rushing us out of the hospital. I’m not saying that to complain because I was okay with leaving sooner (…). It still felt like we were sort of pushed out the door quickly from the hospital.”  Riley 2021  Reference 1 - 0.22% Coverage  A “fear of the unknown…was the worst ” (P2, Age 32, PP 6 weeks) which was perceived to be “hard enough, just in normal life, let alone being pregnant and giving birth through it all as well ”  Costa 2021  Reference 1  “They let us out quite quickly. I only stayed in one night, but it was difﬁcult, especially after having a C-section, because I was trying to do everything on my own.”  Charvat 2021  Reference 1  “For the virus, honestly, I bring it up . . . and they’re very, er, not [open] about it. They just tell me that, “Oh, you’re going to have to wear a mask. We’re going to have to test you.” But they don’t really tell me [anything]. And [my obstetrician] makes me feel comfortable as he tells me not to worry. He says I’m not high risk, etc. But no . . . my OB doesn’t talk much about the actual virus.”  Sweet 2021  Reference 1  “I feel like I’ve needed to ask for more information, around… the early signs of labour and all that sort of stuﬀ, just because I am a ﬁrst-time mum and I don’t know what to expect ” (P26).  DeJoy 2021  Reference 1  One participant explained her fears of having a caesarean birth during a pandemic this way, “We’re going to force people into more surgeries that use more resources in a risky environment and have longer hospital stays? Tell me how that’s logical.”  Kolker 2021  Reference 1 - 0.37% Coverage  One participant described, “It’s a loss that you cannot really talk to people that you feel you lost because you lost enjoying the pregnancy. You, in a nutshell, lose yourself. You know, in this whole scare, you know, trying to sanitize and do everything, you forget and you do not really experience your pregnancy yourself.”  Stirling Cameron 2021  Reference 1  “I didn’t see my children for two days, I wanted them to come to the hospital so I could see them, but it wasn’t possible because of COVID. My little baby girl was crying all the time without me… I just wanted to be discharged from the hospital and go home to them.” | Women perceived that their preparedness for birth and motherhood was negatively impacted by the cancellation of face-to-face birth and parenting education. This created a sentiment of uncertainty in women as they reﬂected on birth and the post-partum period…. Most women identiﬁed gaps in communication and were information seeking. In relation to birthing options, women sought clariﬁcation on any limitations to water birth and comfort options such as water immersion… Concerns around early discharge Some women viewed the potential for early discharge as a risk to conﬁdence in the early post-partum period, particularly, where additional support was limited in the context of community stay at-home restrictions… Some showed forethought and anticipated the implications of needing early unplanned medical assistance at home. A lack of reassurance surrounding this concern created uncertainty and worry. [Atmuri 2021]  Women were aware that they were deemed to be a vulnerable population who may be at risk if they were to be infected, but that little was known or communicated to them about these risks.  Women’s responses indicated how the dearth of  evidence and lack of information related to outcomes exacerbated these fears. [Meaney 2021]  Living the experience of a ‘pandemic’ pregnancy was reported by women as strenuous and difﬁcult, with one woman recalling frequent cries during the last month before birth. The worries were ampliﬁed by the fact that they felt having a ‘double responsibility’, not only for themselves but also for their child… Being pregnant and the idea of giving birth during a pandemic shaped feelings of chaos and uncertainty. The women recognised an overall sense of chaos within the global, national and local macro-environments in terms of COVID-19 disease, symptomatology, transmission, treatment and maternal/neonatal outcomes. [Fumagalli 2021]  Many women described how their pregnancies had been overshadowed by anxiety about whether their partner would be allowed to be present and whether they would be able to give birth at the place and in the manner they had chosen. Their stress was increased by uncertainty: policies changed over time and were inconsistent between different providers. [Harrison 2021]  Service reconﬁgurations extended to the intrapartum period, about which pregnant women were often warned by midwives… Whilst some women were reassured and told sensitively that their birth experience would be diﬀerent, others did not receive such supportive care [Silverio 2021]  As labour and birth approached, changes in women’s care options became evident. These varied based on the time during the pandemic in which they gave birth. First things to be implemented were the support person restrictions and use of PPE. Soon after, pain relief options such as nitrous oxide (gas) and water immersion were restricted. These changes caused great concern for the participants and heightened anxiety about labour… This uncertainty and poor communication led to sense of anxiety… Some participants spoke about reassessing their maternity care model because of the pandemic. For some this was either out of fear of health service closures, the risk of exposure to the virus in healthcare settings, or the restrictions placed on care provision.  [Sweet 2022]  The reduction in non-surgical, non-pharmaceutical options to facilitate an uncomplicated pregnancy, labour, and birth extended to the hospital setting as well, as pandemic policies limited the pain management options available in hospital. For example, baths were closed in many hospitals to reduce sterilization duties for staff, and opportunities to advance labour and manage pain through movement were limited by policies restricting labouring women to their birthing rooms. Furthermore, policies limiting the number of support persons meant that doulas were not present, depriving labouring people of coaching and support. Finally, nitrous oxide, which many women viewed as a milder form of pain relief compared to epidurals and narcotics, was prohibited in most hospitals due to fears of aerosol transmission of COVID-19. This left only epidurals and opioid narcotics for pain control. Carla (first-time mom, Kingston) describes this predicament… While motivations likely vary among obstetricians, the reflections of interviewees such as Olivia (second-time mom, Halifax) suggest that some physicians may be offering interventions due to their own COVID-19-related concerns... Scaled-back postpartum care in hospital Many women who gave birth in hospital reported that the post- partum care they received there was either reduced compared with their previous experience(s) or was inadequate. For instance, some, like Rebecca, a second-time mother from Toronto, were discharged from hospital unexpectedly soon after giving birth [Rice 202b]  Concern and uncertainty were exacerbated by media coverage [Riley 2021]  Some women viewed the potential for early discharge as a risk to conﬁdence in the early post-partum period, particularly, where additional support was limited in the context of community stay at-home restrictions… Some showed forethought and anticipated the implications of needing early unplanned medical assistance at home. A lack of reassurance surrounding this concern created uncertainty and worry. [Atmuri 2021]  Participants also felt their time in hospital had been very brief due to reduced turnaround times. Some felt they hadn’t had enough time to recover from the birth [Costa 2021]  …many participants’ stories included a lack of informational support from their healthcare providers regarding how the COVID-19 virus would affect both their pregnancy and the birth. Grace discussed that her obstetrician did not give her concrete information about how the virus could affect her or her developing fetus [Charvat 2021]  Whilst most participants spoke of seeking information about the pandemic and its possible impact, some participants spoke of more general information needs about pregnancy and birth, as they felt these were not adequately covered in their telehealth appointments. [Sweet 2021]  Others felt that a hospital birth might force them into accepting unwanted interventions, which were particularly dangerous during a time of high health care resource use. Most participants in the study were sceptical of interventions, but some also worried about how providers overworked by the pandemic might coerce them into a caesarean birth or be stretched too thin to care for them appropriately if they did have surgery. [DeJoy 2021]  Participants reported a profound sense of loss due to  COVID-19. Their experience of pregnancy and postpartum was not aligned with their hopes and dreams. [Kolker 2021] | **[Limited knowledge during early stages of pandemic]** The limited knowledge available about the impact of the virus on pregnant women and their unborn babies affected the relationship between healthcare providers and women, reducing trust. The lack of communication meant women felt ill-informed, and unprepared for birth. This was also partly due to the cancellation of face-to-face birth and parenting education, women were unable to navigate hospital surroundings, felt uneasy entering hospitals, and unclear about their birthing plans. For women advocating for their birthing plans, they faced many challenges in receiving information about the hospital policies, and were often told by midwives to enquire closer to the delivery date as policies and restrictions may change. Women who were exposed to the virus, and tested positive found themselves in greater unfamiliar territory as healthcare providers were uncertain about management. An unclear response from HCPs increased anxiety in women. (Atmuri 2021, Fumagalli 2021, Harrison 2021, Silverio 2021, Sweet 2022, Riley 2021, Meaney 2021, Charvat 2021)  **[Planning ahead]** Due to the constantly changing health policies at the beginning of the pandemic, e.g. cancellation of water births, limiting nitrous oxide during pregnancy, women felt the need to plan ahead and take back the control they lost due to the pandemic. Women opted for home births, caesarean sections or planned inductions so that they could have some sort of control over who was present during the labour, as well as the different pain management options. For some women, especially first-time mothers, antenatal care via telehealth did not adequately prepare women for labour and birth, this meant mothers needed to seek out additional information about preparing for labour and childbirth. (Harrison 2021, Silverio 2021, Sweet 2022, Rice 2021b, Atmuri 2021, Sweet 2021)  **[Loss of autonomy]** Many women described the anxiety surrounding their birthing plans, querying whether they would be enacted in the manner they had chosen and whether or not their partner or support person could be present for the birth. This stress was exacerbated by the uncertainty of the pandemic, whether or not policies would change over time, and the mixed messaging from healthcare providers. Some women were not able to exercise autonomy over their births, with some women regretfully feeling pressured or persuaded by their healthcare providers to have caesarean sections or epidurals to aid labour and childbirth. (Harrison 2021, Silverio 2021, Rice 2021b, Sweet 2022, DeJoy 2021, Kolker 2021)  **[Early discharge]** Discharge policies from hospitals were described as both a blessing and a curse. Women were able to discharge early to reunite with families, however some felt that the turnaround time was too quick and they felt they were being pushed away from the hospital. The possibility of early discharge reduced the confidence in mothers as some feared that they would not be able to seek medical assistance when required during the early postpartum period. In extreme cases, women discharged themselves early for the lack of care in hospital. (Harrison 2021, Atmuri 2021, Costa 2021, Stirling Cameron 2021) |
| 2.2. Reduced support and partner presence in healthcare settings | | |
| Silverio 2021  Reference 1 - 0.27% Coverage  “I’m not clear that the policy on fathers not being able to be present until you’re four centimetres dilated is a necessary decision. ”  Reference 2 - 0.05% Coverage  “Found ward after delivery very difficult with no partner/help. Hadn't slept in 4 days, bleeding profusely, hadn't eaten in 30 hours. Really struggled with baby on own without shower, toilet, food, water. Needed help!”  Reference 3 - 0.03% Coverage  “My husband could not be with me while I was being induced, we were scared he might miss the birth so he sat in the car in the hospital car park for 11 hours.”  Reference 4 - 0.07% Coverage  “Partner not allowed in until I & baby were unwell and needed emergency c-section … I had complications during labour, these in themselves were scary, but doing it alone and my concerns being completely dismissed by one midwife was awful. My husband was there for less than an hour total. I was traumatised, alone and frightened.”  Reference 5 - 0.02% Coverage  “Not having my partner there. Did not enjoy crying on my own in a dark room whilst contracting.”  Harrison 2021  Reference 1  “It was horrendous. I have never felt so alone and vulnerable in my life. I understand it is a busy time for everyone and stressful due to covid, but we weren't treated with care or kindness afterwards - almost like the midwives were forgetting we'd given birth in a pandemic. I was offered no breastfeeding help, which led to damage to one of my nipples and poor milk supply … I was talked down to, scared, alone, sleep deprived, and offered none to very little help. I could not wait to leave the hospital. I firmly believe the aftercare plays a part in my postnatal depression. It was traumatic, and I do not use that word lightly.”  Reference 2  “Found ward after delivery very difficult with no partner/help. Hadn't slept in 4 days, bleeding profusely, hadn't eaten in 30 hours. Really struggled with baby on own without shower, toilet, food, water. Needed help!”  Reference 3  “Maternity ward not enough emotional and physical support and understanding, considering women are without support of partner, friends and family at this time with Covid restrictions. Mothers get no respite whatsoever from the care of their newborns, creating exhaustion and anxiety beyond the norm. Physical support is also lacking, such as help in dressing etc., for mums recovering from stitches or caesareans. Midwives seemed unaware of the full true impact of partners being missing from the ward.”  Reference 4  “There was no privacy for us to bond with the baby as a couple before my husband had to go. He was asked to leave an hour after baby was born, and I was left alone, completely numb from the chest down and unable to even lift my baby out of the cot or change his nappy or do anything for him.”  Reference 5  “On the ward during lockdown was hell. I was starved, dehydrated, lonely, sleep deprived and it wasn’t related to my baby. It was like I was in isolation in prison. No music, no tv, no daylight or fresh air. Just me and baby... curtains closed due to covid. Baby who was slightly unwell. Lots of different people touching my baby when they’ve been in contact with covid positive mums. No communication. No explanation. No daddy.”  Reference 6  "I was completely clueless on so many things that I was heavily relying to learn about in my classes. When it came time for breastfeeding, I had no idea what to do or any challenges that could come. There were so, so, so many questions and I felt so confused during everything.”  Kyno 2021  Reference 2 - 0.67% Coverage  “How did it come to that it was allowed for fathers to be with the mothers but not their infants? It is tough to have a premature infant, and being the only parent with access, or being the one who is not allowed to have a father who feels that he is not a father [crying]. It was really tough! All the questions and things he was wondering. Such practical things with the infant’s development, but also how to know the infant; feeling safe and not feeling scared every time he visited the infant. . .. We were never allowed to be there together. . . We could not accomplish any safety together. . .. It has been hard that I know more than him; that I must teach him in everything. I must retell everything, that we are not equal in a way.”  Fumagalli 2021  Reference 1 - 0.23% Coverage  I regret the fact that my husband couldn’t be present at birth. He’s a very important ﬁgure and he would’ve been of support. And I would have liked to share this moment with him really. (W11) And then there was all the sadness of going back home and not being able to see him [partner], he couldn’t touch the baby and all those things. (W22)  Reference 2 - 0.14% Coverage  I arrived at the emergency department and I had to say goodbye to my husband and go in on my own [ . . . ] so I went in and he stayed outside in the car park [woman cries]. He saw her [baby] only on video. (W17)  Reference 1 - 0.13% Coverage  I was sorry cause my partner has not seen the baby anymore during pregnancy after the anomaly scan. It’s a missing piece, you can’t fully share these moments without being together. (W10)  Riley 2021  Reference 1 - 0.22% Coverage  The absence of partners was described as “a piece of jigsaw missing ” (Participant 19, Age 34, PP 8 weeks) and meant they felt they had “been robbed of [the experience of their] ﬁrst child ”  Sweet 2021  Reference 1 - 0.22% Coverage  One woman described how she feared she would be alone in labour, as “because with all these restrictions …my husband can’t come for labour … because someone has to look after our son”  Anderson 2021  Reference 1 - 1.23% Coverage  “The midwife appointments we do mostly from the car, but I have to go in to have my blood pressure checked, and then the worst thing was last week my scan, my husband wasn’t allowed to come in with me so I had to go in to do my first scan on my own which was horrible. But yeah everything has either been done in the car or with masks on and by myself.”  Reference 2  “birth partners being allowed in hospitals and not being able to stay after even if they are allowed for the birth as result of Covid. […] is one thing I’m am anxious about more so that the risk of catching it.”  Atmuri 2021  Reference 1 - 0.40% Coverage  “One of my coping mechanisms is having my partner there to hear the same things I am hearing because I kind of shut down sometimes when I get too upset and I don’t listen to everything. So, it’s always good to have that second person listening . . . and walking out with strength of unity.”  Reference 2 - 0.26% Coverage  “I always have a slight fear when I go into the hospital . . . that my  husband – he’ll get a temperature at delivery and won’t be able to join me.”  Kolker 2021  Reference 1 - 0.21% Coverage  Another loss frequently expressed by the participants was the  partner missing out on prenatal visits and imaging visits, “I think for him it was just kind of sad not to be super part of the experience it was just kind of sad for him not to get to see the ultrasounds and stuff.”  Linden 2021  Reference 1 - 0.86% Coverage  “It was probably the first difficult experience of the pandemic. That you had to come to the hospital and if there was anything negative or in any way any deviation, then they would call my partner. So, he sat […] at the cafe and waited and held his breath… and I was up there by myself”  Panda 2021  Reference 1 - 0.24% Coverage  “You know going through something and he wasn’t allowed be there. You know, like, to hold your hand, or to tell you that it would be ok. To reassure you that everything was going to be okay. It’s just, I found that very hard, yea it was, that was quite diﬃcult now. ”  Rice 2021a  Reference 2 - 0.50% Coverage  “I was so sick, I needed help. I needed someone to walk me to the bathroom, and I needed help getting out of bed. At the time, your support person was only allowed to be with you for 4 hours after your child was born. So ... that was incredibly, incredibly hard being in the hospital by yourself, especially not feeling well and with a new baby. I almost felt like I was in jail, honestly, at times.”  Rice 2021b  Reference 1 - 0.13% Coverage  “I felt like I was put in a room with a baby and I was forgotten about. (…) I think that there needed to be more compassion, especially where I was alone without my support person”  Saleh 2022  Reference 1 - 0.09% Coverage  “It still affects me though, because I didn’t have anybody there that I wanted, I had to trust a stranger.”  Davis 2021  Reference 1  “To do it alone is terrifying”  Jackson 2021b  Reference 1  “I think they should’ve put tests…as soon as possible they could’ve put testing in place for partners, that are women who are in labour, so the partner can come in… to be on your own in labour is horrendous. It’s just…it’s, that’s-nobody should have to go through that, I don’t think.”  Keating 2021  Reference 1  “I didn’t experience the ‘not having your partner there’… but my god it just terrifies me, the idea that I wouldn’t have anybody that was on my side who knew me while being so very vulnerable which you are in labour”  Reference 2  “it was a team effort and I felt that had been taken away by having to go to the appointments… by myself”  Reference 3  “I would have benefitted hugely from having him being able to go to the NICU… He would have asked questions”  Reference 4  “It’s this whole thing of… seeing partners as visitors as opposed to partners”  Reference 5  “chances are your birth partner is a member of your household”  Meaney 2021  Reference 1  “Having so many less appointments due to covid restrictions. My boyfriend not being allowed to attend any appointment or ultrasound for our baby” (P22; Canada, 30–34 yrs, nulliparous, third trimester)  Reference 2  “Not having my husband as a visitor after our baby is born is honestly a horriﬁc thought, it’s the only thing I would change. But it’s the worst possible thing that could happen to me.”  (P106; Ireland, 25–29 yrs, nulliparous, third trimester)  Stirling Cameron 2021  Reference 1  “No one could accompany me to the hospital because of COVID. I was alone. My husband drove me to the hospital, but I was all by myself during delivery. My husband helped me carry my things with me to the hospital, but other than that I was all by myself.”  Reference 2  Other participants who delivered without support people had similar feelings, describing their births during COVID as “hard,” “scary,” and “lonely.”  Reference 3  “My kids at home were so upset that they couldn’t visit me at the hospital and see the baby. They had been so excited during my pregnancy saying that they are waiting to visit me in the hospital after I give birth and to hold the baby, they wanted to bring me flowers but of course because of COVID none of that happened.” (Participant 6).  Reference 4 - 0.44% Coverage  “When I entered the surgical room I cried. I was so lonely. I was scared. I needed someone to be with me to ease my stress. So, the interpreter, who I thank from the bottom of my heart, told me not to worry and told me that she will be there for me.”  Sweet 2022  Reference 1  “just be prepared, come June your husband may not be able  to come in” leaving her thinking she would “face labour alone”.  Reference 2  P24 said, “that’s what … affected me the most and freaked me out, that I was going to be alone, because I had a bad experience previously of being in hospital and … I was like ‘oh crap I’m going to have to do this all by myself’ and I can’t handle that”  Reference 3  “he missed a lot of it. I think it really affected the bond before baby was born, like he didn’t really connect”.  Farrell 2021  Reference 1  “The nuchal translucency and the blood draw, the screening test… for that I was still pretty nervous about the health of the fetus. I was more worried about finding out by myself”.  John 2021  Reference 1  “I had to go to all my appointments on my own.”  Rhodes 2020  Reference 1  “We’ve looked at the videos (on Baby Buddy) of the baby moving in the womb. It’s good for my husband as he can’t go to my scans. He hasn’t even heard the heartbeat …. I worry he isn’t bonding with the bump.” | However, restrictions were often viewed as arbitrary and, therefore, changes to birth plans, including presence of birth partners, were frequently reported as unnecessarily frustrating [Silverio 2021]  However, many more women described the intense loneliness, anxiety and emotional strain of being separated from their partner shortly after birth (often within an hour), and the sadness this had caused their partners who missed out on their child’s earliest days. Many women said they had found it hard to cope physically without the support of their partner and other visitors, particularly if they were recovering from a difficult birth or a caesarean section. They described the staff on postnatal wards as too busy to give practical help with baby care or to assist post-operative mothers with personal care such as showering. Many women reported Covid-19 hospital policies where their birth partner was only permitted to be with them when the mother was in active labour, defined by the hospitals as at least 4cm dilation of the cervix. They described distressing experiences of labouring alone in a setting they had not chosen, with staff too busy to give adequate support, while their partners waited many hours outside to avoid the risk of missing the birth. A few women had not been permitted any birth partner at all. [Harrison 2021]  How did it come to that it was allowed for fathers to be with the mothers but not their infants? It is tough to have a premature infant, and being the only parent with access, or being the one who is not allowed to have a father who feels that he is not a father [crying]. It was really tough! All the questions and things he was wondering. Such practical things with the infant’s development, but also how to know the infant; feeling safe and not feeling scared every time he visited the infant. . .. We were never allowed to be there together. . . We could not accomplish any safety together. . .. It has been hard that I know more than him; that I must teach him in everything. I must retell everything, that we are not equal in a way. [Kyno 2021]  Depending on when women tested positive and were quarantined, missing the partner’s physical presence occurred throughout the antenatal, intrapartum and postnatal period, with culmination at birth… Some women described their labour and birth as unfulﬁlled,  reporting feelings of bitterness and melancholy due to not having been able to share this experience with their partner. The separation from the partner at birth was lived as extremely distressing. Fathers were only involved in the parental relationship via technological means. Women reported the need to make up for the non-shared experience and lost time by telling events to the partner in every detail. [Fumagalli 2021]  The coronavirus pandemic had a considerable impact on participants’ and their partner’s experiences of pregnancy, birth and missing out on several aspects associated with a ‘typical’ pregnancy. [Riley 2021]  The restrictions on visitors in homes caused logistical challenges for some participants who needed support with care for younger children. [Sweet 2021]  Women expressed that they valued the role of a partner or support person in antenatal visits and advocated for their inclusion… There was apprehension about the possibility that their partner  or support person could be absent at birth or post-partum. [Atmuri 2021]  Participants reflected on how the public health mandated separation from family resulted in missing out on much needed support for them and the baby. Another loss frequently expressed by the participants was the partner missing out on prenatal visits and imaging visits. [Kolker 2021]  Not having their partner with them during maternity care appointments was a major concern for many women and this caused some anxiety. Women described feelings of being left alone in vital pregnancy related decision-making. Not being able to fully share pregnancy-related experiences with their partner, like ultrasound check-ups, affected them negatively. Most women were able to handle this however, many worried that they would be alone if they had to receive any bad news. Especially, the anatomy scan was perceived as a critical moment in the pregnancy that they feared having to face by themselves. [Linden 2021]  While women described their midwives as being ‘lovely’ and very supportive during their early labour experience, for most, this was not enough. They were aware that midwives had to care for many women, and they ‘needed’ their partner. This need was described in terms of having their partner with them for reassurance and constant companionship, and for the simple yet important gestures such as hand holding and back rubbing [Panda 2021]  However, for women whose deliveries entailed medical intervention, the lack of postpartum support in hospital resulted in suffering. For example, the second quote in Box 1 provides the experience of a woman whose husband was required to leave shortly after she gave birth via emergency caesarean delivery, leaving her alone, and the third quote conveys the experience of a woman who was hard of hearing and had long-standing clinically diagnosed struggles with anxiety; her husband was required to leave the hospital 4 hours after she gave birth to her second child by scheduled caesarean delivery. [Rice 2021a]  Furthermore, many interviewees who remained longer in hospital struggled to get care, especially if their birth partners had been required to leave shortly after the birth [Rice 2021b]  This reality-based experience was impacted by hospital policies continuously changing during an unfolding pandemic, and by individual biologic and medical factors of the delivering mother/neonate dyad. [Saleh 2022]  A major worry for women was potentially not being able to have a support person at medical appointments and at the birth. One participant in the ‘low’ group had to labour by herself and she strongly believed that this should not have been allowed. Even for women in the high group they emphasised the importance of a support person being there for the whole journey. [Davis 2021]  Consequently, respondents feared the potential consequences of discontinued face-to-face healthcare professional support on infant and maternal wellbeing. Respondents felt considerations had not been made regarding the unique needs of new mothers, such as allowing partners to be present at essential healthcare appointments and during labour. (author interpretation only) [Jackson 2021a]  A final suggestion made by participants to improve the support available to new mothers during the early postnatal period included greater provisions to be put in place for allowing partners to be present during labour. [Jackson 2021b]  One of the most distressing aspects of the change in  care during the pandemic was the restrictions on partners attending hospital visits, particularly the anomaly scan. One woman described attending out of hours with reduced fetal movements and found this experience particularly difficult to go through alone. While none of the hospitals prevented partners from attending the birth of their children, the potential for tighter restrictions on partners caused a lot of anxiety. Women feared losing their birth partner as an advocate while they were in labour and after the birth. Women described that by partners missing out on appointments, he/she felt disconnected from the process… Strict NICU restrictions during the initial lockdown meant that partners were not permitted to visit. One woman who was affected by these restrictions felt this added to the stress of having an unwell baby. Not having the baby’s father present in the NICU added to concerns about how he would bond with the infant. [Keating 2021]  Women felt that support from their signiﬁcant other was diminished during this pregnancy due to restrictions put in place by the maternity hospital. Women reported both sadness and anxiety that their signiﬁcant other could not be present as a support or to participate in antenatal appointments, at birth or during the postpartum stay in the hospital. [Meaney 2021]  Many participants who delivered during the pandemic discussed the impacts that hospital restrictions and larger public health measures had on their birthing experiences. A culmination of factors, including limited access to childcare, restrictions on support people, and the unavailability of doulas, meant that many women laboured and delivered alone, or with fewer supports than they would have liked. Several women in this study gave birth during the early months of COVID-19 with no support people, as their husbands took care of their children while they were in the hospital… Not only did this interpreter ensure that the participant was able to communicate with her healthcare team, but she also acted in lieu of the participant’s support people, providing comfort and support [Stirling Cameron 2021]  Limitations on support people for women in labour and birth varied based on the trajectory of the pandemic at the time, health services’ own policies, and government restrictions. The most described situation was being limited to only one person and that person was not able to alternate with anyone else. All the participants giving birth in hospitals described concern, fear, or disappointment about not having their support persons of choice with them in labour… Some participants were pleased they had completed their antenatal care in person before the restrictions commenced, while others spoke of attending their antenatal care by themselves, as partners and children were not permitted, resulting in distress and worry. [Sweet 2022]  Women were particularly troubled that partners were not able to come to the scan appointments, experiencing this as an acute loss, with one worrying about the impact on partner-child bonding. [Anderson 2021]  Participants described how experiences like having a partner listen to the infant’s heartbeat during appointments were missed. (Author interpretation only) [Dove Meadows 2020]  As a result of visitor restriction policies, many participants underwent ultrasound‐based screening and diagnostic testing procedures without a partner or support person. This was a concern for women who wanted to share the experience of seeing the fetus with a partner. Yet, for many, the concerns pertained to the possibility of learning about a fetal abnormality or demise by themselves during periods when visitor restrictions were in place... For participants in this study, the fear of being alone was unexpected—something most did not anticipate or fully realize the implication of until the time of the ultrasound appointment. [Farrell 2021]  Perinatal women also noted being unable to have support personnel present at pregnancy and postpartum appointments and the resulting emotional impact of this change. (Author interpretation only) [Green 2021]  Most women reported feeling isolated during their pregnancy due to features specific to the SARS- CoV-2 pandemic. This was particularly a problem for those who felt that they would have benefitted from the presence of a companion when important information relating to their pregnancy was being relayed to them [John 2021]  The greatest disappointment for pregnant respondents appeared to be that their partners were missing out on antenatal appointments. Women worried that less involvement might adversely affect their partner’s bonding with the baby. [Rhodes 2020] | **[Impact of reduced partner presence on women]** An immense sense of unfairness was interpreted from the responses of women. Partners were unable to attend pregnancy appointments, be present during labour and unable to support women and enjoy their babies on the postnatal wards. Some women had to deliver alone, and as a result increased anxiety and stress, as well as an overwhelming sense of sadness for mothers that were not able to have their support people present. Many feared the possibility of their partners getting a temperature or COVID-19 in the lead up to the birth, adding to the already present anxieties. Additionally, many felt that these policies were unfair and for fathers with babies in neonate intensive care units, did not take into account that fathers were an essential part of the family unit, with an equal right to be with the baby, as much as the mother. One mother’s response adequately summarised this issue, saying they “felt robbed of their experience”. Partners were described as advocators for women and by limiting their presence during the perinatal period had a significant negative impact on mothers. (Harrison 2021, Kyno 2021, Fumagalli 2021, Riley 2021, Atmuri 2021, Linden 2021, Silverio 2021, Jackson 2021a, Keating 2021, Meaney 2021, Stirling Cameron 2021, Sweet 2022, Dove Meadows 2020, Green 2021, Sweet 2021, Davis 2021, Jackson 2021b, Anderson 2021)  **[Inability to support women in the antenatal period]**  Women feared being alone for their antenatal appointments and need the support if bad news needed to be delivered. Women also described how new fathers during the pandemic missed the opportunity to create new memories during pregnancy appointments, ultrasound scans, be advocates for the mother if the pregnancy needed to be closely monitored. Not having their partners present for antenatal checks negatively affected women, causing anxiety and stress. Some women also feared that reduced partner presence during antenatal checks would adversely affect their bonding with the baby (Linden 2021, Farrell 2021 John 2021, Fumagalli 2021, Anderson 2021, Riley 2021, Kolker 2021, Panda 2021, Keating 2021, Meaney 2021, Sweet 2022, Rhodes 2020)  **[Inability to support women post-birth]** For some mothers, partner’s presence post birth was limited to a few hours per day, limiting the ability for partners to provide the physical support for mothers recovering from birth. For some families in addition to hospital policy, due to the restrictions, there was an even greater negative impact on their experience as they could not rely on anybody to take care of older children, so partners had to remain home. Some mothers reported they cried the whole time and sought early discharge from hospitals so that they were able to reunite with their families. Midwifery support was available, described as both lovely for mothers who coped well, and lacking compassion for mothers who felt minimally supported, however it was not the same as having their partners present. (Harrison 2021, Sweet 2021, Rice 2021a, Rice 2021b, Saleh 2022, Meaney 2021, Stirling Cameron 2021, Anderson 2021, Silverio 2021, Keating 2021) |
| 2.3. Transitioning to telehealth, virtual and remote care | | |
| Silverio 2021  Reference 1 - 0.51% Coverage  “So, I’ve had no Health Visitor follow-up. Just to know that your baby’s healthy, I just ﬁnd it hard that people weren’t physically seeing her, it was all done by phone. As I say, I don’t know what she should weigh, and I’m weighing her myself which might not be accurate, and you’ve just got to hope that she’s doing okay. And yes, you just need to sort of wing it really. So yes, I think the ﬁrst six weeks were very isolating and very hard, and then I think yes, I don’t know, maybe once the baby starts getting slightly easier to cope with, maybe you just get on with it a bit more; I don’t know.”  Reference 2 - 0.36% Coverage  “They were trying to do the appointments very quickly, which I understand. I understand it was for the safety of me and them, but there was that sense that if I did have any questions I couldn’t really sit there and ponder them. I had to really be thinking do I have any questions because I have to ask them because they are going to try and get me out as soon as possible. That felt a little strange. It all felt very clinical”  Reference 3 - 0.38% Coverage  “I think I would question the accessibility of that. Not everyone does have a smartphone and so expecting people to be able to receive a video call is not necessarily the most inclusive thing. Although where it is available, it would be nice.”  Reference 4 - 0.14% Coverage  “I ended up doing a phone appointment where I was in the waiting room and the doctor was in his room [laughs] which was the weirdest GP appointment I have ever had.”  Rice 2021b  Reference 1 - 0.75% Coverage  “At least two thirds, maybe three-quarters of my visits until the third trimester were virtual, which is okay for answering questions and stuff, but I noticed that I wasn’t gaining weight myself. I wasn’t going into the clinic and weighing every time, they weren’t checking fundal height [a measurement of fetal growth]. I remember there being a lot more checks, a lot more physical exam stuff during my first pregnancy. [This time around] I felt a little bit like the onus would have been on me to identify anything weird going on.”  Davis 2021  Reference 1 - 0.04% Coverage  “I didn’t have one successful phone call appointment.”  Reference 2 - 0.11% Coverage  There were differing opinions on these support services; several women were quite positive about telehealth: “Telehealth option is amazing.”  Jackson 2021a  Reference 1 - 0.25% Coverage  “[Midwives] want to get you off the phone as soon as possible’ cause they have such a high number of people they’ve got to deal with erm so you do feel a little bit rushed.”  Reference 2 - 0.28% Coverage  “I’ve spoken to a health visitor a couple times and it was just like [imaging speaking to health visitor], “I know you’ve gotta tick a box, but that is really pointless. Wasting my time and yours.”  Atmuri 2021  Reference 1 - 0.27% Coverage  “You don’t have that physical connection with someone or just being, knowing that they can physically see you and assess you. I had a miscarriage only a few months before I actually fell pregnant again . . . I just feel like that being delayed (the physical) it just made me more anxious”  DeJoy 2021  Reference 1 - 0.92% Coverage  “That distance, and that whole disconnect from somebody actually being able to see me and pick up on all the external cues. You know, when you sit down with somebody, they can tell there’s more going on when there’s more going on, even if you’re not saying it? So, it was just like that, kind of impersonal.”  Rhodes 2020  Reference 1 - 0.15% Coverage  “My antenatal care is now much more rushed and stressful. There is no time to talk to the midwife and it very much feels like a quick process to check my urine and blood pressure only. This makes me feel less connected and more anxious”  Sweet 2022  Reference 1 - 0.25% Coverage  “She [the midwife] would call us, tell us ‘I’m out the front in the car, strip the baby off naked and bring her to the front door, I’m going to leave the scales on your front doorstep, put the baby on the scales, tell me what it says, and then I’ll pick the scales up and disinfect them’ … it was definitely really weird”.  Reference 2 - 0.11% Coverage  “like they gave information on [how] to buy or hire a thing to take your own blood pressure before the appointment … I don’t think that’s ideal”  Reference 3 - 0.09% Coverage  “… I went to a chemist, like they don’t do it [blood pressure] on you, but they had a machine you could use yourself”,  Reference 4 - 0.06% Coverage  I have a fetal Doppler at home, so I’d just listen myself to the heart rate”.  Reference 5 - 0.13% Coverage  “Well my biggest concern is you can’t listen to the fetal heart rate over the phone and that’s sort of my early pregnancy worries because of my [miscarriage] history”  Reference 6 - 0.24% Coverage  “Having to perform their own assessments left women feeling concerned that they may miss providing relevant information, for example, “she’d [the midwife would] give me lots of information, asked me all the questions … but it was definitely always playing at the back of my mind like, what am I missing I guess”  Linden 2021  Reference 1 - 0.46% Coverage  “There have been many improvements with… digital meetings, which meant that you didn’t have to take time off work…. So, I think it has been a much more positive experience, easy accessibility…”  Costa 2021  Reference 1 - 0.39% Coverage  “My health visitor (and I), we weren’t ever in (physical) contact with each other. She had to stand and wait outside… All of these times  she’s been coming to my house, but she’s actually not laid eyes on (my son).”  Reference 2  “I was grateful that the appointment could still go ahead.”  Kolker 2021  Reference 1 - 0.19% Coverage  “I never had a six week postpartum appointment … I have not had a doctor look at me since I left the hospital … you’re on a virtual call and you can’t really explain things, you’re taking pictures that aren’t clear, it’s really, it’s not helpful.”  Panda 2021  Reference 1 - 0.14% Coverage  “And over the phone just doesn’t do it like. You don’t get the same, to look into somebody’s eyes and to trust them and for them to say, you’re okay.”  Stirling Cameron 2021  Reference 1 - 0.43% Coverage  “I found the services I got when I delivered during COVID much better than those with my firstborn baby here. I liked the services while delivering and the period after. They kept calling me, checking on me and asking how I was doing.” Similarly, participant seven said telephone appointments were “much easier” than in-person visits.  John 2021  Reference 1 - 0.17% Coverage  But they said, no you can’t come, you have to send a picture and you have to email us. Which is sometimes, you know, is not easy to do with the phone. Because I’m not a doctor.  Keating 2021  Reference 1  “I just find that was much better in a home setting than in the hospital setting when there’s so much going on”  Anderson 2021  Reference 1  “All the classes have been cancelled, and you can’t just go… you feel like you haven’t got time to sit in there and have a chat and ask these questions that you want to ask”  Harrison 2021  Reference 1  “I wish all appointments were face to face as you can't have your blood pressure or baby's heart rate checked over the phone. My pre-eclampsia wasn't picked up until I went into labour at 36 weeks, as my last couple of appointments had been via phone.”  Snyder & Worlton 2021  Reference 1  ‘‘Everyone has still been available via telehealth options or via phone conferences so I don’t think those resources have lessened at all’’ | Whilst not favoured in the postnatal period as much as it had been accepted for certain aspects of antenatal care, virtual care was tolerated as a better alternative to no care at all, which many postnatal women faced…  In relation to the reduction of antenatal and postnatal care, women often mentioned virtual care was sometimes used as an alternative, however, was recognised as not being accessible to all. [Silverio 2021]  For most participants, most prenatal care appointments took place virtually. This meant that much of the routine monitoring (e.g., blood pressure, fetal growth, and weight) that would ordinarily have been carried out by their physician or midwife became their own responsibility to manage at home. [Rice 2021b]  Women discussed a range of virtual supports they utilised during this period including telephone, videoconferencing (telehealth), social networks, online applications, text messaging, and videos. [Davis 2021]  Several respondents spoke of feeling that available healthcare professional support was time restricted. Worries were raised concerning potential implications of missed face-to-face healthcare visits to infant safety... Virtual healthcare was insufficient in meeting postnatal needs and was often perceived as more of a ‘tick box’ obligation rather than having received quality care [Jackson 2021a]  Women acknowledged the role of telehealth in minimising the risk of COVID-19 transmission but they did perceive telehealth as a compromise of their pregnancy experience, with some describing it as impersonal due to limited physical connection with clinicians and care feeling rushed. [Atmuri 2021]  Others noted that receiving prenatal care via telemedicine contributed to their isolation. One participant captured the sentiment of participants who felt that telemedicine, although a necessary safety precaution, added to their feelings of isolation. She had experienced a prior traumatic birth and reported anxiety due to the following [DeJoy 2021]  Pregnant respondents often expressed their concern and disappointment at receiving what they regarded as suboptimal antenatal care. Although some respondents pointed out that telephone appointments reduced their risk of contracting COVID-19, many worried about the aspects of care they might be missing. Indeed, a common sentiment was that of being disregarded or sidelined. [Rhodes 2020]  The increased use of infection protection measures including personal protective equipment (PPE) was also discussed and left some women feeling bemused. Infection prevention measures were increased during home visits as a result of the pandemic. Explained how her postnatal home visit was conducted... Many participants spoke of being asked to find ways to perform physical assessments, such as have their blood pressure assessed before telehealth appointments or weighing their baby on household scales. [Sweet 2022]  One of the things that the women found positive from the COVID-19 pandemic was that communication with the midwife through digital meetings was made possible. For some women digital appointments increased accessibility and flexibility. [Linden 2021]  Once participants had left hospital and returned home, many felt that essential community care, such as support from health visitors, had been adversely affected, which had a negative impact on parents’ postnatal experiences… Due to social distancing measures, many multidisciplinary consultations were moved online or were delivered over the telephone. For some participants, this new method of communicating with CL/P teams was described as limiting… Some participants talked about remote consultations positively, expressing gratitude that the support was available at all [Costa 2021]  Patients reported less access to IUD insertions and pelvic floor physiotherapy during pregnancy and postpartum as well. The quality of postpartum care was also compromised. [Kolker 2021]  Routine GP care was disrupted during COVID-19, both antenatally and postnatally. Some women were happy to avoid their GP surgery as they viewed it as a potential site for COVID-19 transmission. Antenatally, cancelled GP appointments resulted in longer intervals between antenatal assessments. This was worrisome for some women who felt that four weeks between antenatal assessments was too long. Postnatally, telephone consultations were used by some GPs, particularly for the routine two-week infant assessment. Many women were left non assured by these telephone consultations, and their preference would have been for their GP to see them and their baby in person. [Panda 2021]  Janis had multiple telehealth visits for breastfeeding assistance and she liked the experience because she did not have to leave the house. Although Stella said the telehealth visit for lactation was awkward because her partner had to hold the computer at an angle for the IBCLC to see the latch. She was not conﬁdent that the visit was accurate or effective. Janis speciﬁcally reported that she was planning to go to support groups regularly both for the support and also so she could weigh the baby. Due to social distancing, she purchased a scale for home use so she could weigh her son at home. Another interesting shared experience is that two of the mothers had a postpartum visit conducted via telehealth prior to the time of being interviewed by the authors. For both of these mothers, there was no option to have an in-person visit. Using telehealth, the perineum was not examined, so there was no assessment of whether the mother was healing well from the birth. [Spatz & Froh 2021]  Telehealth appointments alleviated the need for some women to find childcare and transportation to attend in-person appointments. [Stirling Cameron 2021]  (Theme – barriers to access of care) In circumstances where participants did seek physical consultations, they experienced barriers and often had to repeatedly call in order to be seen [John 2021]  Many hospitals changed to provide antenatal classes online. One woman felt that doing pre-recorded classes took away from the experience as it was less individualized… Some women had virtual or telephone consults which they felt de-personalized the experience with one woman describing a phone consultation with her obstetrician at 16 weeks as “tokenistic.” [Keating 2021]  Telephone appointments felt less personal, more removed. [Anderson 2021]  Women pointed out that telephone appointments meant they had missed out on important and reassuring aspects of antenatal monitoring such as blood and urine tests, and checks on the baby’s heartbeat, position and growth. This had led in some cases to serious maternal health conditions remaining undiagnosed, or emergency caesarean following undiagnosed breech position. [Harrison 2021]  Although the majority of women stated they wanted access to in-person lactation information, two first-time mothers did not feel it made a difference. [Snyder & Worlton 2021] | **[Accessibility]** Telehealth and remote care was adopted by healthcare providers as a means to reduce face-to-face contact during the pandemic. Some mothers preferred telehealth for its ease of use, increased accessibility, and reduced the need to find external childcare when attending appointments. Many were grateful that telehealth was still available, however, some pointed out that telehealth may not be equitable for all, as not everyone has the same access to smart technology to attend virtual appointments. For some services, such as family planning and physiotherapy, women were not able to access these as they had been indefinitely closed or limited in their ability to service women. (Silverio 2021, Linden 2021, Spatz & Froh 2021, Stirling Cameron 2021, Davis 2021, Keating 2021, Anderson 2021, Snyder & Worlton 2021)  **[Questioning the quality of care]** Whilst women adapted to using virtual care for their appointments, many questioned the quality of care given healthcare providers were not able to physically assess women, e.g. weight gain, fundal height growth, etc. They felt that the onus was on the woman/mother to ensure that she could check her own blood pressure and keep track of her baby’s growth. This was not equitable as not everyone had the same access to medical equipment. In extreme cases, the lack of routine antenatal face-to-face care appointments “in some cases to serious maternal health conditions remaining undiagnosed, or emergency caesarean following undiagnosed breech position” (Harrison 2021). Women also reported that the virtual care appointments felt impersonal, and even rushed with little opportunities to ask questions. During telehealth consultations, taking pictures and videos for their healthcare provider was described as awkward and not ideal. Many found this challenging and an inadequate response to care. Additionally, some described the experience as not meeting standards, with women describing the need to physically see healthcare providers to trust them to say that they were doing okay. For high-risk women, the delay in seeing a healthcare provider F2F increased anxieties around the current pregnancy. Women were not able to garner a sense of reassurance from their healthcare providers and felt disconnected in a time where support was needed most. (Silverio 2021, Rice 2021b, Jackson 2021a, Sweet 2022, John 2021, Davis 2021, Atmuri 2021, DeJoy 2021, Rhodes 2020, Kolker 2021, Panda 2021, John 2021, Harrison 2021)  **[Remote care post-birth]** Many women felt that the community care after birth was lacking in that the healthcare providers were unable to conduct proper assessments on the mother and the baby due to the public health restrictions. Mothers felt that virtual appointments were impersonal, and they were only being conducted to tick a box and useless in assessing whether or not the baby’s growth was adequate, a general sense of women feeling unsupported throughout the process. For women that received socially distanced care from midwives in the postnatal period, some described the experience as “weird”, as innovative methods were being used to conduct assessments on the newborn and others had a poor experience stating that their health visitor never “laid eyes on her son” (Sweet 2022). The inability for healthcare providers to provide a safe environment for questions and reassure parents that they were doing the right things, increased stress and anxiety, especially for new mothers. (Silverio 2021, Rice 2021b, Davis 2021, DeJoy 2021, Sweet 2022, Jackson 2021a, Costa 2021) |
| 2.4. Barriers to accessing health services | | |
| Stirling Cameron 2021  Reference 1- 0.39% Coverage  “Before COVID, many Syrian women were offered doulas by [immigrant support group], but I was not offered one. Because of COVID, they stopped this service.”  Kolker 2021  Reference 1 – 0.40% Coverage  I wasn’t able to go to pelvic floor physio which really was a bummer for a while because I think I have a lot of problems that would probably be helped by that and it just kind of sucked to – again, it’s just one of those things where it’s like everything you read, “Here’s what you should do to help with these things. You just can’t do those things.” So it was kind of upsetting for a while to feel like I know I needed help with this and needed some help with the healing side of things and I just couldn’t get it.  Davis 2021  Reference 1 - 0.16% Coverage  “That doesn’t help me when I’ve got something I’m worried about right now.”  Reference 2  “For six months, there’s a whole group of mothers from late February to March/April that were just forgotten about and those mothers, some of them now at nine months make no connections with any other mothers because they were just left.”  Jackson 2021a  Reference 1 - 0.35% Coverage  “I did NCT [National Childbirth Trust – a charity which provides antenatal classes] so I’ve been able to do the face-to-face sessions about halfway through and what was really frustrating, and has impacted me now, is towards the end when the pandemic kind of started, my last sort of interaction won’t have face-to-face.”  Reference 2 – 0.27% Coverage  “We had no home visits at all from any health professionals which [sigh] is okay, but you do worry about the fact that the baby’s environments aren’t being checked…obviously we know it’s okay but [laughter] they [health visitors] don’t.”  Panda 2021  Reference 1 – 0.44% Coverage  “It’s just the whole after care, it hasn’t been there do you know. That you don’t get any visits, … even if you just got, …he [baby] hasn’t been weighed; you don’t know how he’s getting on …is he coping okay …is he hitting his milestones that kind of thing.”  Rice 2021b  Reference 1 – 0.39% Coverage  “My two-week follow-up with the OB was over the phone. They would not see me in-person, which I was really upset about because I would rather have a doctor see if everything is healing properly. And then, with my daughter, I just wanted to make sure she was gaining weight, and for the doctor just to check her and make sure there is no jaundice or anything (…) I think she was three weeks, and then they finally agreed to see her in person”  Reference 2  “I reached out to an acupuncturist and he started protocols to encourage the baby to turn. I only got one appointment with him and then his clinic had to shut down…I reached out to my chiropractor because I had heard really good evidence about chiropractic and helping with breech babies. I got to see her once and then her clinic had to shut down. (…) I had been going to the pool and swimming quite a bit. I had been told it could be really helpful to encourage the baby, but the pool shut down. Suddenly all of these options that I had were just gone and I felt quite frustrated and helpless. (…) I really wanted to do whatever I could to try to have a vaginal birth. Like, COVID sort of changed the world and it felt like it changed the control that we had over our pregnancy and the birth of our child as well.”  Harrison 2021  Reference 1 - 0.13% Coverage  "It was horrendous. I have never felt so alone and vulnerable in my life. I understand it is a busy time for everyone and stressful due to covid, but we weren't treated with care or kindness afterwards - almost like the midwives were forgetting we'd given birth in a pandemic. I was offered no breastfeeding help, which led to damage to one of my nipples and poor milk supply … I was talked down to, scared, alone, sleep deprived, and offered none to very little help. I could not wait to leave the hospital. I firmly believe the aftercare plays a part in my postnatal depression. It was traumatic, and I do not use that word lightly.”  Meaney 2021  Reference 1 - 0.11% Coverage  “Having so many less appointments due to covid restrictions. My boyfriend not being allowed to attend any appointment or ultrasound for our baby”  Reference 2 - 0.27% Coverage  “To have had classes as a ﬁrst time mom, not just have classes cancelled and feel abandoned by the system. Medically I know I’m ﬁne which is one thing but I’ve felt angry that nothing was put in place to replace the classes online. I would have liked more time with midwives to chat and get to know them or a doula that could be there on the day.”  Reference 3  “There is going to be a lot of fallout from these cancellations that no-one will really pay attention to but that will have a great effect on the women concerned e.g. back pain, pelvic ﬂoor dysfunction, breast feeding difﬁculties, PND [postnatal depression]" (P103; Ireland, 35–39 yrs, nulliparous, third trimester)  DeJoy 2021  Reference 1 - 0.45% Coverage  “That hospital [where the participant had been seeing a CNM] got closed down. So basically, that happened in February and I was 30 weeks pregnant or something and it was kind of like, okay, now enter COVID.”  John 2021  Reference 1 - 0.39% Coverage  “Because when I was explaining about my breathing, they didn’t want to see me, they were saying that they think that I’m having a coronavirus…and I really forced, yeah, I said I’m not having a coronavirus, it’s like this is… I know that this is related to my cardiomyopathy. I have really, really struggled, I can say that. I’ve been calling, calling, talking, talking, they don’t want to see me.”  Reference 2 - 0.10% Coverage  “But it was mainly up to me as well to read and ask questions…rather than getting, um, like information.”  Rhodes 2020  Reference 1 - 0.13% Coverage  “I don’t think I am being supported. The Government should be giving us more information, like where do you go to get your baby weighed…there were 8 weeks when she wasn’t weighed – quite a worry really.”  Riley 2021  Reference 1 - 0.27% Coverage  “weren’t given any information on which [health] service(s) [they] should contact and for what period of time they would support”  Reference 2 - 0.07% Coverage  “My sister-in-law is a midwife and she has been a godsend”  Reference 3 - 0.15% Coverage  Participants also relied on “friends and family for information on the changing requirements or how appointments are being conducted”  Reference 4 - 0.23% Coverage  Participants particularly found solace in friends who were also new mothers: “I had quite a lot of friends who have had babies in the last sort of 12 months or so, they have been a lifeline really”  Jackson 2021b  Reference 1 - 0.35% Coverage  “I think they could’ve done a campaign, just something to say, you know, we know it’s hard for you, we’re here. This is where you can go. This is what you can do…I think there are mums out there who do feel lonely, and they don’t know where to go.”  Reference 2 - 0.52% Coverage  “I think they could’ve thought a lot more clearly about the support, new mums-in terms of checking that emotionally they’re okay and also checking their home environment. I actually think they’ve just kind of stopped it without really thinking about the implications of that and I think that, you know, they should’ve continued home visits in a relatively safe way, and yeah, having a proper six week check with the GP I think was important and more ways of checking on the health, you know, the progress of your baby. I just think having such a long period of time without any contact or any checks on the baby at all is dangerous [laughter]”  Ollivier 2021  Reference 1  “We had one meeting with public health before they reassigned our nurse to the COVID team and we were dumped. The breastfeeding clinic cut her tongue tie but did a phone follow up instead of in person due to COVID and dumped us even though we were still having feeding issues.”  Sweet 2022  Reference 1  “My sister’s godmother is actually a … midwife, so when this all started … if things were going to get worse and the hospitals are going to shut down … I did have a chat to her that if we have to go homebirth, I need her to come over. But we’d never planned for homebirth before that”  Reference 2  “I really didn’t get proper support from the child health nurses until week five. … I actually got a bit of post-natal depression this time as well”.  Reference 3  Similarly, P9 said, “It was very [disappointing] … he hasn’t been weighed in about 3 or 4 weeks, and that was just weight, he hasn’t been measured or anything, not properly since birth. There’s a lot of gaps in the system”.  Costa 2021  Reference 1  “I feel like (my son) is trying to make more sounds now, trying to do more from a speech point of view, but he can’t. I feel a little bit anxious about that, wanting him to get surgery sooner rather than later for that reason.”  Anderson 2021  Reference 1  “Everyone is stressed, and all the services are stretched, you just don’t want to feel like you want to not waste their time, but normally you would be able to go to someone or go to a service like your midwife and ask them and they would have time to speak with you […] but because they are so stretched now you just don’t feel like you want to… everyone bangs on about save the NHS and you don’t feel like you want to go in there asking anything”  Reference 2  “All the classes have been cancelled, and you can’t just go… you feel like you haven’t got time to sit in there and have a chat and ask these questions that you want to ask”  Silverio 2021  Reference 1  “I didn’t like the fact that I had to travel out with a baby. I was lucky I had a car because I thought to myself, “If I didn’t have a car now, I wouldn’t get on a bus.” Because I remember saying to the Midwife, she said to me “Do you drive?” I said “Yes.” She goes, “Oh brilliant, you could park.” I said “Well, what happens if I don’t drive?” And she goes “Well, you’d have to get a bus.” I said, “But with a two week old baby, on a bus, during a lockdown, during a pandemic? ”I went “That’s not really safe.” and the attitude I got was like “Oh well, you just have to do it.” It wasn’t very concerned, if anything, it was “You’re making our job a little bit easier,” that was the feeling I got from the conversation, “You’re making our job a little bit easier because we don’t have to do the home visits, you can come to us in the clinic”  Reference 2  “I would say: don’t have any expectations for the midwife care up until the point of labour. At the time you feel cheated out of these appointments. You keep looking at this book that tells you, you should have been seen in all these weeks and you are thinking, I have been seen face-to-face twice. You feel a bit cheated and anxious that they maybe are missing out on something…”  Aydin & Aktas 2021  Reference 1  “Unfortunately, I could not go to have tests like detailed ultrasound and triple screening during this period. At that time, the virus was very intense in Trabzon … our appointments were constantly being cancelled. It scared me a lot, so I decided not to go to the hospital.”  Spatz &Froh 2021  Reference 1 - 0.41% Coverage  Stella further added that she believed she received adequate lactation assistance prior to discharge. She stated “I felt a little lost once I got home and found myself constantly looking things up online and asking friends for advice.”  Snyder & Worlton2021  Reference 1  ‘‘The face to face meetings because of COVID. I really miss those’’ (36, Caucasian, educator). | In addition to the restrictions on support people, several women who delivered during the first wave were unable to access doula services, as doulas were temporarily suspended with the rise in COVID-19 cases. Doulas were frequently used by Syrian newcomer women prior to the pandemic [Stirling Cameron 2021]  Patients reported less access to IUD insertions and pelvic floor physiotherapy during pregnancy and postpartum as well. The quality of postpartum care was also compromised. [Kolker 2021]  The majority of women highlighted that they needed increased access to support, particularly face-to-face services. Most frequently, women discussed their needs to access Child Health Nurses (CHN) once they had their babies, as women believe they are important for mums’ emotional support as well as the child’s development. Some women expressed their frustration about delays in appointments, with one mum reporting delays of up to 2–3 weeks to see a CHN. [Davis 2021]  Disappointment was also felt in the lost time which could have been spent attending parenting classes and interacting with other mothers [Jackson 2021a]  Women spoke to other women as a source of breastfeeding support. They spoke to friends or family who had experience of breastfeeding to determine if what they were experiencing was normal. Several women deferred to a family member who was a healthcare professional for advice on mastitis or with other baby related concerns like weight gain and tongue tie. A few women related challenges they experienced in navigating baby’s progress and gaining assurances that their baby was achieving his or her milestones to the restricted follow up appointments by the PHN and GP service. [Panda 2021]  However, many more women described the intense loneliness, anxiety and emotional strain of being separated from their partner shortly after birth (often within an hour), and the sadness this had caused their partners who missed out on their child’s earliest days. Many women said they had found it hard to cope physically without the support of their partner and other visitors, particularly if they were recovering from a difficult birth or a caesarean section. They described the staff on postnatal wards as too busy to give practical help with baby care or to assist post-operative mothers with personal care such as showering. Some first-time mothers whose antenatal classes had been cancelled were disappointed that staff were not willing to educate them about baby care and breastfeeding. [Harrison 2021]  Women felt that support from their signiﬁcant other was diminished during this pregnancy due to restrictions put in place by the maternity hospital. Women reported both sadness and anxiety that their signiﬁcant other could not be present as a support or to participate in antenatal appointments, at birth or during the postpartum stay in the hospital. Women were also aware of how restrictions on access and care differed between hospitals within the same geographic area, which added to their frustrations… Under the theme maternity care impacted by COVID-19 (see  Table 3), some women reported dissatisfaction with changes to the maternity services which were implemented to reduce the risks of transmission of the virus. Women reported, “how the virus has ruined everything and changed maternity care”, whereby routine antenatal appointments and antenatal/parenthood preparation classes were postponed, cancelled or telemedicine clinics were provided in lieu of in-person appointments. Women reported that antenatal care is “vital” with antenatal classes considered “an essential service” that needed to be promoted in order to empower women to maintain their health and wellbeing during pregnancy. [Meaney 2021]  The scaling-back of in-person care extended into the postnatal period as well… For these women, the inaccessibility of these therapies meant that biomedical technologies were their only option for managing complications. For example, Molly in Victoria had intended to access professional support to flip her breech-presenting baby. While attempting to flip the baby would have been a priority regardless of the pandemic, the risks posed by surgery and longer-term hospitalization during the pandemic made avoiding a c-section even more pressing. Although her doctor had initially given her hope that a flip might be possible, her options for achieving this soon dwindled. [Rice 2021b]  One participant explains that her search for a home birth midwife began after her small rural hospital closed [DeJoy 2021]  In circumstances where participants did seek physical consultations, they experienced barriers and often had to repeatedly call in order to be seen. On the contrary, some women delayed seeking medical help due to apprehensions surrounding contracting SARS- CoV-2. [John 2021]  A lack of clear guidelines from the government led some respondents to feel that they were a forgotten sector of the population. Although initial guidelines had suggested that pregnant women should shield, there was confusion as to whether this meant staying indoors or being allowed out to exercise and whether it was still applicable (by May) or had been superseded by new advice. Postnatal respondents were not aware of any advice directed at them as parents of young babies, and many were critical of its absence… A small but meaningful number of respondents had experienced postponed or cancelled hospital appointments for themselves or their baby, which had caused substantial anxiety. [Rhodes 2021]  Many of the participants acknowledged that the perinatal period is a particularly vulnerable and disruptive time in a woman’s life, and expressed that they would have liked a more active Governmental response to informing new mothers of where to find support during this difficult time [Jackson 2021b]  This uncertainty and lack of information was still an issue postpartum. Participants talked about how they “weren’t given any information on which [health] service(s) [they] should contact and for what period of time they would support ” (P14, Age 32, PP 15 weeks) following the birth of their child… Participants also relied on “friends and family for information on the changing requirements or how appointments are being conducted ” (P10, Age 39, PP 6 weeks) and family members who were health professionals: “My sister-in-law is a midwife and she has been a godsend” [Riley 2021]  Public health orders during COVID-19 made accessing information and support very diﬃcult for mothers in our study. Many participants said they were concerned that they did not have the right information about how to best care for their baby, including how to safely breastfeed. With unclear information, mixed messaging, and unknown reliability of certain information sources, parents had to make diﬃcult decisions regarding what sources and who to trust when it came to caring for their new baby. [Ollivier 2021]  Some participants spoke about reassessing their maternity care model because of the pandemic. For some this was either out of fear of health service closures, the risk of exposure to the virus in healthcare settings, or the restrictions placed on care provision… Inadequate access to postnatal care was raised by all the participants who were postpartum at the time of the interview. In-home postnatal visits were minimal or not offered at all, and postnatal services such as maternal, child and family health visits or new parent classes were, in many cases, cancelled as deemed non-essential services, which disappointed participants. [Sweet 2022]  …participants expressed concerns about the impact of surgical delay on their child’s longer-term speech development. [Costa 2021]  Lockdown restrictions and subsequent maternity service reconﬁgurations aﬀected routine appointment schedules and the frequency of those appointments. Antenatal care visits were found to have been either altered frequently or cancelled altogether, with women discussing their perceptions of virtual care appointments as having less value or importance… In addition, women discussed how reduced frequency of antenatal care appointments made them feel, with provision of virtual care not equated to the in-person care they had either expected or wanted [Silverio 2021]  Maternity care changes were a major concern for most  of our participants. While recognising that midwives  were doing their best in a difficult situation, many  women had experienced not only a loss of care, but a  lack of communication about changes to their healthcare, with reports that midwives have been ‘hard to get hold of’. The loss was particularly acute for women in their first pregnancy, who did not know what they were missing out on… They also felt acutely the loss of antenatal classes – for the important information they were missing out on and  the chance to meet other pregnant women. Women  wanted ways of replacing these losses, with some mentioning paying for digital antenatal classes, while others simply had no access. [Anderson 2021]  Some of the women who had prenatal visits at state hospitals reported that they postponed them because of the risk of coronavirus either with their own decisions or at the request of their doctors. [Aydin & Aktas 2021]  Although the majority of women stated they wanted access to in-person lactation information. Furthermore, one third of mothers reported concerns about being unable to obtain effective support for latch issues through telehealth. [Snyder & Worlton 2021] | **[Limited access to alternative health providers]** The health policies that reduced the capability of many healthcare providers meant that women could not access additional support and care from health professionals outside of their obstetric teams. Women who had some sort of care pre-COVID-19, such as access to doulas, physiotherapy or alternative health services, felt the drastic change when these supports were no longer accessible. For some women, their local hospitals or maternity services closed due to the pandemic, therefore women were forced to search for alternative health services, often further away and increased travel time. Many women missed these face-to-face interactions with their healthcare providers. (Stirling Cameron 2021, Kolker 2021, Jackson 2021a, Rice 2021b, Meaney 2021, DeJoy 2021, Anderson 2021, Snyder & Worlton 2021)  **[Delayed or reduced healthcare provision]** Women described the disappointment and frustrations that they had when there were cancellations and delays to appointments, or could not meet their healthcare providers face-to-face, expressing the lack of physical and emotional support for mothers and their newborns. Some women described their healthcare providers placed a significant burden on their ability to access care, with women reporting their doctors emphasised not coming into hospitals and only for mandatory examinations, tests or difficulties; some were even unwilling to treat patients with respiratory issues. Others had to repeatedly call and express their concerns before healthcare providers accepted their call for help. Not only was care of the woman lacking, many women criticised the healthcare system in the postnatal period, with many women describing no postnatal checks were conducted on their newborn, and few follow ups were completed on time. The most common 6-week home-visit was either delayed, or completed via telehealth, which many found unhelpful and useless. Mothers with newborns that required additional support, e.g. babies born with a cleft palate, described the impact of the delays to surgical interventions due to the pandemic. These events occurring on their own or in conjunction with another deteriorated women’s mental health. For some mothers, they felt that it was a burden on healthcare providers, therefore refrained from asking more questions for fear of taking up more time. (Davis 2021, Jackson 2021a, Rice 2021b, Harrison 2021, Meaney 2021, Jackson 2021b, Panda 2021, John 2021, Rhodes, 2020, Ollivier 2021, Sweet 2022, Costa 2021, Aydin & Aktas 2021)  **[Limited information about where to get support]** Many women felt that the government and healthcare providers did not provide enough information for women seeking support post-birth. Due to the limited F2F access to antenatal and postnatal services, women who were struggling or worried, did not know where to go or who to turn to for concerns. Little support was provided by healthcare providers and the responsibility was placed on mothers and their partners to seek their own information to make informed decisions during the pandemic. Some mothers accessed their informal networks to determine how and where to get more support. There was also increased concerns about newborns having limited F2F care, to check feeding habits, weights, and jaundice levels. Some women showed empathy towards other mothers, those who may not have had the sources to access more information and “fell through the gaps” of the stressed healthcare system. (Rhodes 2021, Davis 2021, Panda 2021, Riley 2021, Jackson 2021b, John 2021, Meaney 2021, Ollivier 2021, Sweet 2022, Anderson 2021, Silverio 2021, Spatz & Froh 2021)  For the results para:  **[Relying on family and friends for information]** Women described accessing support networks through their family and friends to receive advice for women during their pregnancy and postpartum period. Women who were able to access nurses and midwives through their personal networks felt relieved as they were able to ask questions and have their worries and concerns addressed. Some women also relied on friend who were also pregnant at similar times to receive information and updates about COVID-19 guidelines and hospital restrictions. (Linden 2021, Spatz & Froh 2021, Riley 2021, Charvat 2021) |
| Adapting to alternative ways of receiving social support3.1. Accessing support through different avenues | | |
| Linden 2021  Reference 1 - 0.65% Coverage  “It’s sad that parent groups didn’t take place, during pregnancy, to meet people that way. But now there is a lot on the internet… so I have gotten in touch with a mother via the internet, so that you get the social interaction…”  Charvat 2021  Reference 1 - 0.35% Coverage  “I would say that he basically stayed on the phone with me all hours of the night. Just so that I had someone there, not there with me, but someone like making sure that I was still functioning and okay. And he drew me a picture full of, like, supportive words that he brought to me in the hospital ‘cause he could bring me stuff to the lobby . . . He just couldn’t come and be there with us.”  Reference 2 - 0.41% Coverage  “My mom has really been my sounding board. She is a retired nurse. And with the possible [COVID-19] exposure, I think I called her, like crying, because I was so upset, and just . . . being a person to bounce things off of, and even just to tell you, “It’s probably gonna be OK, you know; we don’t know if it’s going to be”. But she has been very, very helpful, very strong, for me in the whole process.”  Reference 3 - 0.20% Coverage  “My sister-in-law lives locally and she’s been a really great support for me. Just being able to talk to her about what my fears and anxieties are and how being pregnant during this time sucks and is totally different.”  Reference 4 - 0.25% Coverage  “They’ll call and be like, ‘Do you need me to get you anything? I’m at the store, that way you don’t have to go out.’”  Reference 5  “She will tell me how it is, especially about, like, medical things . . . she’s been like a really good sounding board of just like, ‘Do you think I should do this?’ or ‘Do you think this is safe?’  Reference 6  “Even like gifts in the mail, we were supposed to have a baby shower, the weekend after everything shut down. And so, definitely got a lot of gifts in the mail and people who drop things off on our porch. And so that was nice to, you know, especially with this being a second baby, to feel like even though he’s being born in this super crazy time and he doesn’t necessarily get to meet people in person, that they are excited about him and want to support us.”  Reference 7  “We have a group text going on, and we can share how we’re feeling. Having people that are all in the same boat has been super helpful in just kind of navigating it, and not feeling like I’m alone.”  Riley 2021  Reference 1 - 0.30% Coverage  Some participants sought support from “several Facebook groups where …women who were pregnant and giving birth during coronavirus, loads of them, all around the world, America, Canada, there were lots of places and they were all posting their positive experiences”  Reference 2 - 0.19% Coverage  Some forums were useful as they had “diﬀerent people’s experiences on there” but also had “its negatives as well, because you get a lot of…judgemental people now”  Reference 3 - 0.19% Coverage  Some forums were useful as they had “diﬀerent people’s experiences on there ” but also had “its negatives as well, because you get a lot of…judgemental people now”  Reference 4  “thanks to lockdown my partner is here 24-7…even though he is working…we actually had the beauty of sharing both of our love with the baby, so if I am tired I can take a nap and he can take care of the baby for half-an-hour” (P16, Age 33, PP 17 weeks)  Ollivier 2021  Reference 1 - 0.20% Coverage  “I have not searched for any information but I did start an online support group for other extroverts like myself who are struggling where I post a daily challenge to help keep myself and others motivated”  Sweet 2021  Reference 1 - 0.15% Coverage  “just this morning I caught up with my local mothers’ group –so a lot of mums are using Facebook to arrange postcode mums’ groups”  Reference 2 - 0.36% Coverage  Similarly, another spoke of using technology to reach supports and overcome loneliness, she said, “Well I guess because normally you’d go out and have coﬀee and just meet with other mums …there’s been a lot of just staying at home, …so it is quite lonely sometimes, but luckily there’s Zoom and Skype and Facetime”  Reference 3  “So there were a few groups that popped up [on Facebook] with women just trying to support each other during that time ”  Reference 4  “There was even some [other women] volunteering to look after people’s children when they were meant to have ultrasounds, people they’d never met, and they were like ‘I’ve got no one to watch my kids and I can’t take them to the ultrasound because of COVID’, and people were like ‘well where do live, I can watch your kids while you go’. It’s like okay you’re going to leave your kids with someone you’ve just met on a Facebook group because you’re that desperate”  Kolker 2021  Reference 1 - 0.43% Coverage  “I have my sister, I have my aunt, but because they were moving around, the thing is, they weren’t stopping their life, so I called them on the phone, but they wouldn’t come to me. You know, for us, the community and our culture is, we get together. We do family things. We meet every time. Every week we are together, we are eating, but now that was disconnected because they would not come, they are moving around, they have kids in daycare. So that was like gone because of the COVID. So we talked on the phone. That supposedly was support, but not as much as I wanted.”  Jackson 2021a  Reference 1 - 0.11% Coverage  “We have a very active WhatsApp chat erm and yeah I mean I get a lot of stuff from the internet but it’s not-it’s not quite the same.”  Reference 2 - 0.23% Coverage  “I’ve met up with my friends a couple of times over sort of Zoom, had a glass of wine with them. But it’s not the same.”  Reference 3 - 0.30% Coverage  “They’ve [parenting groups] all been trying to do things on-line, but it just isn’t the same. You’ve gotta be there. It’s about the social interaction… and to be honest, I don’t really think baby groups are for babies, they’re for the mums.”  Reference 4  “It’s the intimacy of those conversations [on-line]. You’re losing the kind of… yeah, the connection.”  Aydin & Aktas 2021  Reference 1 - 0.21% Coverage  “We only cancelled home visits because I live in the village. Apart from that, we sat in the gardens at a distance and chatted. Or we called each other and chatted from window to window, garden to garden….”  Reference 2 - 0.18% Coverage  “There has been a change in my social life. For example, instead of meeting at home and the cafe, we met our friends in the forests on the mountain slope by keeping our distance.”  Reference 1 - 0.23% Coverage  “We could spend time together … We had breakfast together. Before the pandemic, I used to leave home early so we couldn't have breakfast together, but we are all together in this period, so it affected me positively.”  Reference 2 - 0.16% Coverage  “… Since I couldn't get out of the house, my husband tried to take care of me more. We spent more time together…He even helped me with housework in the evenings”  Joy 2020  Reference 1 - 0.33% Coverage  “It’s been great . . . we have this opportunity to bond as a family and he [partner] is here for every moment during the newborn stage! It has been amazing not having to worry about visitors coming and going and cleaning out home and me worrying about breastfeeding in front of others - instead we have a very relaxed atmosphere for everything!”  Reference 2  “He is available to help with diaper changes and to hold the baby when I need a quick break, but it isn’t what I pictured for my maternity leave”.  Brown & Shenker 2021  Reference 1 - 0.23% Coverage  “My partner has been furloughed so he is here everyday with us, he can help with nappy changes, looking after our baby and letting me sleep when I need to, basically everything I'm addition to enjoying so many special moments together seeing our baby develop, having 2 of us here all the time means there's much more time for me to focus on breastfeeding our baby.”  Jackson 2021b  Reference 1 - 0.29% Coverage  “My husband’s working from home every day or was, which is great because it means that he can cuddle [baby] for 10 minutes and I can go offand do something for my sanity round the house, even if it’s just hoovering a room. Erm, and, but when he’s at work, which he was yesterday, I find the day’s really, really long.”  Reference 2 - 0.34% Coverage  “I’ve got the baby to feed so often and just been, you know, sat there doing nothing else but feed the baby at times so [husband] does everything else. If he wasn’t there, going back to work and things like that when the kids are back to school- there’d’ve been no way for me to carry on [with breastfeeding] ‘cause it was taking so much of my time.”  Kolker 2021  Reference 1 - 0.22% Coverage  “His presence provided company and support … it was almost like a blessing in disguise.”  Reference 2 - 0.13% Coverage  “Others appreciated the postpartum support from their husbands who were working from home, “having him being able to do more of the cooking and helping me get a nap every day.”  Saleh 2022  Reference 1 - 0.05% Coverage  “We had a lot more time to just hang out altogether.”  Anderson 2021  Reference 1 - 0.63% Coverage  “technology generally has helped loads, just being able to see friends but on a screen or see colleagues but actually see their faces has made a massive difference to communication”  Panda 2021  Reference 1 - 0.09% Coverage  “So, it [introducing the baby to the family] was all done through skype and WhatsApp videos.”  Reference 2 - 0.14% Coverage  “WhatsApp is brilliant. And I don’t know what we’d all do without it. Because she [baby] sees both of them [grandparents] at least once a day [via WhatsApp].”  Davis 2021  Reference 1 - 0.15% Coverage  “It’s been really great that my husband had been able to work from home a lot throughout the year . . . So, if that’s one thing that we could keep, would be him to continue to work from home.”  Reference 2  “ . . . limiting the access to social media as well, that would be one of the ongoing things to [my] wellbeing.” (L_CW12).  Rhodes 2020  Reference 1  “My baby is having the best start to life! Full undivided access to her mummy and her daddy!! For 2 months!” [Free text]  Costa 2021  Reference 1  “I browsed the CLAPA website. It gives a lot of information, it’s packed with knowledge in terms of what to expect and […] there is also a family community where you can reach out.” (Michelle) Jackie similarly reported, “We have been using the […] online coffee chats that (CLAPA) do […]. I’ve listened to the diagnosis ones and the feeding ones and the surgery one. […] When I’ve listened to other people’s experiences it helps me […] think ‘OK, yeah, they’ve dealt with it, that’s what happens’.”  Reference 2  “I actually found (Facebook) really awful. There are some really good and helpful pieces of advice. Everyone is in the same boat trying to help each other out. But it really heightened the anxiety and (made me) compare… It’s all just more worrying.” (Lucy)  Fumagalli 2021  Reference 1  “I was reassured by the support of my parents, family and partner . . . even though there was no physical contact and we could not meet, I used to see them on videocall.” (W6)  Kyno 2021  Reference 1  One mother said that she filmed a situation displaying care in the incubator, filming the entire session. Later she enlarged the film on a computer screen to show the father the infant’s whole body (realistic size), the care, and diaper change.  Reference 2  “It is still the mothers in the hospital I ask about things if I have any questions, and the father is not in that group”  Silverio 2021  Reference 1  “Trying to talk to people. Nothing can beat face-to-face in terms of being able to go somewhere and talk to someone face-to-face, someone coming into your house and having a cup of tea or you going in someone’s house and having a cup of tea, but if that is not possible trying to talk to people, talking to people on the phone. When I came oﬀ the phone from particularly my friends and my mum –anyone who had had a baby, who understood what some of these diﬃculties felt or looked like –I always felt reassured. ” (Participant-013) | Many women expressed missing out on the friendship and companionship of other pregnant women. All group activities such as parent education classes that would normally take place during pregnancy were cancelled due to the pandemic and expectant couples were referred to official healthcare information webpages instead. Despite this, some women found alternative ways of getting in touch with other pregnant women, for example on social media. [Linden 2021]  Similarly, Tori was exposed to the COVID-19 virus while pregnant and had to grapple with the unknown effects of the virus on her developing baby. She described how she called on her mother to support her and assuage her fears after possible contact with the virus.  After Sarah was admitted to the hospital for preeclampsia, a serious pregnancy complication involving high blood pressure, she discussed how her partner emotionally supported her… Finally, Shani said that her family’s willingness to listen to her concerns was especially helpful… Because many participants wanted to limit their time in crowded places to protect themselves from getting COVID-19, their friends and family offered to go to grocery stores and other retail establishments on their behalf… Terry discussed how she talked with her sister – a nurse practitioner – to determine the activities that were safe for her to do… As is common during pregnancy, these new mothers also received gifts from those in their social  network. Gift-giving was unique during the pandemic because most in-person baby showers were cancelled, and in-person visitors were discouraged before and after the baby was born. It felt especially meaningful to the participants when people thoughtfully sent gifts during this time. Thus, receiving gifts from loved ones highlighted both instrumental and emotional support. These gifts seemed to indicate to the participants that friends and family members still cared about their pregnancy and their child, even in the wake of a global health emergency. The thoughtful and supportive gifts lessened the distance and re-established the connection between the expecting family and their support system. As such, receiving socially distant instrumental support serves a dual purpose for these women, as it not only helps satisfy physical needs (i.e., instrumental support), but also represents others’ love and care (i.e., emotional support). Mary described how it was important to talk to her pregnancy group. Being able to connect with other pregnant women helped alleviate negative feelings and reminded them that they were not navigating this challenging situation alone. Consequently, this emotional support assisted these women in coping with their pandemic pregnancy. [Charvat 2021]  First four references are quotes only… Partners who worked from home during lockdown were able to provide a degree of respite for participants. [Riley 2021]  Virtual technologies (such as telephone, Face-  Time or online platforms) were widely used by new parents to keep in contact with their loved ones and to share as much as possible with them. The importance of memory-making and celebrating the baby’s milestones was well-represented in this study. Having a baby was not only a source of pride and joy for new parents, it was something they very much wanted to share and have others be involved with, despite the ongoing pandemic. Some parents also thought of creative solutions to help promote not only their own mental health and opportunities for socialization, but those of others as well. For example, one mother created an online support group on social media with the purpose of connecting with others… Socialization that was in person meant something diﬀerent for parents when compared to online platforms or ways of connecting. For some, online connection was useful, but not as enriching as being in person and was viewed as a ‘next best thing’ solution. (second passage is just the author’s interpretation) [Ollivier 2021]  The social media groups were a way to connect with peers as explained here… With the absence of tangible support from family and friends, participants spoke of actively engaging in peer support with other mothers, both virtually and using social media… Women’s support needs were both practical/instrumental and emotional, and this raised concern for some. [Sweet 2021]  Participants reflected on how the public health mandated separation from family resulted in missing out on much needed support for them and the baby... Due to social distancing requirements, many participants had partners who were required to work at home. This enabled the partner to provide support and companionship as one participant explained. [Kolker 2021]  For many respondents, technology was invaluable in allowing mothers to remain connected with loved ones while face-to-face contact were restricted… Despite using technology to maintain intimacy with friends and  family, all respondents acknowledged that virtual communication paled in comparison with quality face-to-face interactions… Virtual communication with friends and family was perceived as lacking in intimacy compared with face-to-face communication… Virtual parenting classes have also been identified as more difficult to navigate, and less socially engaging, than face-to-face parenting classes [Jackson 2021a]  Some women stated that living in a small town or village contributed to their reduced social life being less dramatic because of the pandemic. The statement of one of the women is as follows… Staying home because of coronavirus negatively affected the psychology of some of the women and changed their social habits or the social environment… Half of the women, most of whom were employed, highlighted that the COVID- 19 pandemic had a positive effect on their spousal relationships, while the other half stated just the opposite. The statements of those whose spousal relations were positively affected revealed that they had the opportunity to spend more time with their husbands… [Aydin & Aktas 2021]  Challenging social ideals was a way of using their agency as they clearly articulated their beliefs about how being at home with their babies was very positive and for some a ‘blessing’. Many participants believed that this was a time for more personal and intimate family enjoyment. A time where the immediate family could not only enjoy each other but also their new baby… The blessing of partners included more support and help with the daily activities of their homes, including cooking and cleaning, as well as baby duties, such as changing diapers and holding. [Joy 2020]  Indeed, having additional parenting support from partners was a vital facilitator for maternal emotional wellbeing… Having one’s partner at home was an invaluable support for sharing parenting responsibilities, which allowed women to persist with breastfeeding practice… Virtual communication with friends and family was perceived as lacking in intimacy compared with face-to-face communication. [Jackson 2021b]  Due to social distancing requirements, many participants had partners who were required to work at home. This enabled the partner to provide support and companionship as one participant explained [Kolker 2021]  Mental health was tied to proximity of support from others either physical (bringing meals) or emotional. Many women recognized that COVID-19 policies allowed them to spend a greater amount of time one-on-one with their neonate and families than typical maternity leave allowed due to changes in work expectations (virtual work and social distancing). Cherished time with families and finding a new norm allowed for development of healthy coping mechanisms and reframing of the negatives of COVID-19 by some mothers. [Saleh 2022]  Home environment and access to resources had a key impact: Participants recognised how lucky they were for resources they had (e.g. access to a garden or local green space, home exercise equipment, a car to avoid public transport), or for digital technology to facilitate social contact. [Anderson 2021]  Adapting to using virtual media platforms as a means of staying connected with family and friends while being in the hospital or on returning home, for example introducing baby to the extended family via video call or zoom, was described by many women. [Panda 2021]  Depending on the working situation, some participants reported that their partner was at home for longer after the birth. Some were furloughed and had much more time to support breastfeeding and maternal recovery from both an emotional and physical perspective. Others were working from home but were still more present than they would have been if out at work all day. This shared care was felt to increase bonds between partner and baby and strengthened the new parent relationship. [Brown & Shenker 2021]  Increased work ﬂexibility was viewed as a positive aspect of the situation, both for women who had the opportunity to work from home during pregnancy and, especially, for their partners… Online social groups were useful for some women, especially via Facebook, but others found the discussions overwhelming and had to shut them down… One of the participants (CW10_high) created a virtual mother’s group in response to the lack of peer connection. This has been very successful and there are over 200 members, some of whom now meet in person. (last one is author interpretation only) [Davis 2021]  A great beneﬁt to all the mothers was having their partners at home with them due to social distancing. All had partners who were working from home due to the pandemic. They reported that this was positive because the partners got to see and experience all the ﬁrsts. [Spatz & Froh 2021]  Here, experiences were divisive; many respondents with a partner working from home really appreciated the additional time spent together. Postnatal respondents pointed out the practical benefits of sharing childcare and household tasks as well as the emotional benefits of the greater opportunity to bond as a family… Missing out on antenatal groups was also disappointing for women, although some were attending existing groups on the web or had found new online support groups or forums to compensate. Although modern technology enables video calls with family and friends, the respondents, especially those with young babies, felt that they were suffering considerably from the loss of face-to-face interaction. Without antenatal and postnatal groups and visits to family and friends, respondents felt that they were missing out on opportunities to share experiences and learn from others. (author interpretation only) [Rhodes 2020]  Participants also reported receiving remote support from community-based organisations, such as CLAPA, which appeared to be a source of information and advice… Participants also referenced using social networking sites to access information and support,… However, not all accounts of utilising social media in this way were positive [Costa 2021]  Compassionate support from the family, partner and healthcare professionals was also a reassuring factor. When women were self-isolating and no physical contact was allowed with their social network, emotional support provided ‘remotely’ by the family and partner played an important role in making them feel cared for. [Fumagalli 2021]  Although most women were understanding of the limitations posed by the pandemic, a significant proportion expressed loneliness exacerbated by not being able to engage with their usual pregnancy support networks, and did not feel that virtual groups mitigated this effect (author interpretation only) [John 2021]  Parents reported that Messenger, Snapchat, FaceTime, pictures, and films were shared with family, extended family, and close friends. Social media was used to counteract their sadness about not being able to socialize and to show the infant to family and friends. Nevertheless, contact and information to the fathers were prioritized… Some mothers established maternity groups via Messenger (Facebook), where they could ask questions, debrief, cry together, and support each other. One such group still existed months after discharge, and mothers referred to the group as something they would not be without. Hence, the mothers used each other for support during this period. [Kyno 2021]  The ﬁnal theme of this analysis covered women’s advice for other pregnant and birthing women. Largely, advice centred around replacing the face-to-face support with virtual forms of social support, and a noticeable under-reliance on healthcare professionals for this type of support: [Silverio 2021] | **[Using social media to connect with others]** For many women, access to F2F mother and baby groups was only available online, usually through social media sites. Some women created their own online forums and social media web pages to share experiences and challenges to keep other women motivated during the pandemic and connected women both internationally and locally to engage online and support each other. The ease of accessibility to online groups meant that women were able to engage with a wider group of people in similar situations, however some noted that online platforms could also be dangerous as women could be subject to negative responses. Although women widely used social media and virtual technologies to connect with others, it was evident that most women reported that “it was not the same” as being able to be physically present with others. (Linden 2021, Charvat 2021, Riley 2021, Ollivier 2021, Sweet 2021, Jackson 2021a, Anderson 2021, Panda 2021, Davis 2021, Costa 2021, Kyno 2021, Rhodes 2020, John 2021)  **[Remote emotional support from family members]** Most women described maintaining their connection with family members virtually, either by phone or video calls. Many women relied heavily on their family members to provide emotional support. They were able to support mothers by being available to assist with everyday tasks such as grocery shopping, providing meals and even gifts on doorsteps so that women did not have to expose themselves to infection and possible transmission. Whilst many overcame restrictions using virtual technologies, they acknowledged that it was not the same as having their supports in person and having the physical element of care. Women without the support of their partners on postnatal wards shared that they provided their support remotely, through video calling and sending words of encouragement. (Charvat 2021, Riley 2021, Kolker 2021, Jackson 2021a, Anderson 2021, Panda 2021, Fumagalli 2021, Kyno 2021, Silverio 2021)  **[Meeting outdoors]** A strategy that some women who were able to access it, was the ability to meet family and friends outdoors, in gardens and parks to stay connected. (Aydin & Aktas 2021)  **[Increased partner presence]** For some women, their partners were furloughed or transitioned to working from home, meaning that they were able to be more present for women during the pregnancy and postnatal period. Mothers spoke of the increased time spent with partners, ability for partners to help out a lot more at home, being present for other children, as well as being there for many of the firsts for their newborn. They cherished this increased time that the family unit was able to bond together. Some mothers took advantage of partners being home, by taking breaks when required and having extra support breastfeeding and childrearing. (Riley 2021, Aydin & Aktas 2021, Joy 2021, Brown & Shenker 2021, Jackson 2021b, Saleh 2022, Davis 2021, Rhodes 2020, Spatz & Froh 2021) |
| 3.2. Desiring connection with family and friends | | |
| Atmuri 2021  Reference 1 - 0.28% Coverage  “The older generation have more experience on what babies need or what they feel . . . with my other two [children] . . . they knew exactly what may make them feel better whereas I have to obviously be on phone and ask, ‘What do you do?’ . . . I don’t know, I will just learn as I go, I guess.”  Reference 2 - 0.35% Coverage  “Last time when my daughter was born, I invited my in-laws and  my family from overseas. It’s going to be too hard for me because my daughter she just started at school. My husband, he’s working. I have to look after my daughter and the new baby as well. It’s going to affect my life.”  Reference 3  “I hope I won’t be going through with depression because of less people around. Sometimes you just need family support or even a friend’s support, just to help you out a little bit. Because your ﬁrst time . . . you don’t really have any idea about anything.” CC  Costa 2021  Reference 1 – 0.37% Coverage  “(Support from family) has been limited due to the restrictions […]. I know that they are there for some emotional support, but in terms of how much they have been involved in (the baby’s physical) care, it has been limited because of Covid.”  Green 2021  Reference 1  “On a positive note, I have been able to quarantine with family and so I am set up with a strong support network” [Postpartum #36].  Reference 2 - 0.13% Coverage  “I am not able to see family as much and as a result I have less social support to take care of the baby. My family sometimes would offer to watch him every now and then, but now I do not have their help”  Reference 3 - 0.04% Coverage  There has been no external support from friends and family”  Reference 4  “I worry about how I am going to be able to take care of my newborn with the help of external support (i.e., my parents)” [Pregnant #31].  Reference 5  “It has been difficult not being able to see my family and friends who have been a huge support for me” [Pregnant #26].  Riley 2021  Reference 1  “Your emotions are all over the show anyway when you’ve had a baby, but …close relatives, not being able to see them, that was diﬃcult ” (P18, Age 31, PP 10 weeks).  Reference 2 - 0.29% Coverage  As some restrictions were lifted, people were able to meet “in the garden” but this was upsetting for participants who were introducing their baby to the family for the ﬁrst time: “just showing them this tiny baby, [was] the hardest thing in the world”  Reference 3  “no one else could come and help and let me have a sleep or just let me know he’s OK”  Reference 4  “Everyone says it take a village to raise a baby and it’s true and we’ve not had our village around us ” (P13, Age 34, PP 7 weeks).  Sweet 2021  Reference 1 - 0.10% Coverage  “the restrictions meant that we’ve lost some supports that we would’ve had otherwise”  Reference 2 - 0.26% Coverage  “It was really rough for the ﬁrst few weeks –my parents live a few hours away so they didn’t meet him until he was 6 weeks old, and the plan had originally been that mum would come up and support us, obviously that didn’t happen”  Reference 3 - 0.24% Coverage  “I was planning to have some support from my family …when the baby was born but my parents couldn’t make it from Canada …and my partner’s parents were planning to come down too and they’re only in Queensland”  Reference 4  “We brought my mum up here …and then they closed the regional borders, …so we had to send her home after three days, so I didn’t have that support that we had planned” (P15)  Reference 5  “There was a lot of concern around what I would do with my children when I went into labour because the plan we had was for them to go with my parents but my parents are in their seventies ” (P10).  Jackson 2021a  Reference 1 – 0.5% Coverage  “It’s [new motherhood] just been sadness, really…The people who you are close to and would usually rely on as most forms of support can’t be part of the things that’re a massive deal to me…he [baby] turned his head for a noise or, or when I’m singing a certain song he does this really cute thing…it’s like, “Aw I’ll just go to my mum’s and show – Oh, no. I can’t”. It’s the realisation, isn’t it?”  Reference 2 - 0.32% Coverage  “I think week two to four was peak tiredness and then that’s the point where I’d really loved either my mum, my mother-in-law, or my own family to sort of step in and be able to help out a bit more.”  Reference 3 - 0.27% Coverage  “Hardest was sort of my parents, who are in their 70’s, was them not having the role that they want to have with the baby, because my mum is so hands on, you know? She…was the childcare.”  Jackson 2021b  Reference 1 - 0.16% Coverage  “I understand what-why the rules are what they were, but if I could’ve, you know, like they’ve done support bubbles for single parents and anyone that’s on their own? I suppose in those early days I could’ve done with support from family.”  Brown & Shenker 2021  Reference 1 - 0.44% Coverage  “I have much less time to focus on breastfeeding now since lockdown. My partner is a police officer he is at work from the crack of dawn until late at night, sometimes staying at work for 24 hrs or more depending on how situations unfold. I am at home with an energetic 5‐year‐old who would normally be in school. I do not have time to express in between feeds or sometimes breastfeed at all because I feel I need to meet my daughters demands and run the house and basically be a single parent most of the time. Before lockdown I was able to have my mum and sisters come and stay and help out. I could also have friends to help me or my older daughter could go for play dates to allow me to focus on the baby.”  Reference 2  “My mother is wonderful and a huge supporter of breastfeeding. I was really looking forward to her coming to visit after my baby was here. She cannot come and whilst we can video message it's just not the same as having your mum close by. I feel I need her, not just to help but emotionally and I'm struggling without this support. It makes everything feel so much harder.”  Joy 2020  Reference 1 - 0.48% Coverage  “We were supposed to ﬂy to Alberta to see my family at the end of March but had to cancel the trip. This is my family’s ﬁrst grandchild so it just breaks my heart they will miss her whole babyhood. I also feel so alone with the baby. I have nobody here to help me ﬁgure out what is normal or how to progress through these early days. Although people can video chat it isn’t the same. I just want somebody to be in the room with me and the baby to see the things she can do and help me with things.”  Rice 2021a  Reference 1 - 0.79% Coverage  “I think I had a bit of postpartum depression. I do have some anxiety as well ... even before pregnancy. So, the first 2 or 3 weeks I felt like I was going crazy. I felt like I was going insane because I was so ill, I couldn’t stay awake for long, I was really weak. My incision wasn’t healing properly. At this time, my boyfriend was trying to work from home, and he wasn’t sleeping and I wasn’t sleeping. I couldn’t reach out for help from my mom. She couldn’t even come over to my house to help me out. So that was super hard for us. It was hard not being able to reach out and have the family there when you needed them the most.”  Reference 2 - 1.54% Coverage  “I have depression and anxiety, for which I am on Prozac and I have a therapist. And, thankfully, they are both fine and under control, but it was really, really difficult for the first few weeks being home and isolated and being afraid of catching COVID. And knowing that our baby doesn’t have an immune system, we don’t want to jeopardize his health or his life by letting people into our house, so we were in essentially full quarantine, and that was really difficult for me. And I know that a lot of the hormonal swings are very normal, but there wasn’t that support for when you’re crying and exhausted. My mom wasn’t there to hand the baby over to so that I could take a nap. After [my husband] went back to work, it was very difficult, physically and emotionally. And I found that, by week 6, I was crying a lot, the baby was crying, I was crying and exhausted, and really feeling the guilt of feeling like I needed help and not wanting to risk our family. How much struggle to endure for their safety versus knowing that our mental health would be so much better if we let one of our parents into the house? And that wound up being the decision that we made with my therapist because it was the simple solution. I needed my mom.”  Stirling Cameron 2021  Reference 1 - 0.17% Coverage  “It was really hard during COVID. In Syria I had my family… but to give birth here with no one with me?! It was really hard. I needed someone with me, my neighbours, my friends… I felt like I was drowning.”  Dove-Meadows 2020  Reference 1  “That one hug, that one kiss on the forehead, that one pat on the back from a family member to tell you that it’s going to be okay and you feel it. And it just heals everything overall. So, I think it may impact a lot of pregnancies. I think ... postpartum depression rates will probably skyrocket because everyone’s isolated and you’re stuck in the house with your children and you can’t get out, you know, if you’re stuck. You, you don’t want to do any harm to your kids and you don’t want to, you know, ignore them or have a mental breakdown, but I really feel like the, the rate of it is going to skyrocket. Because we’re all trapped in a way, you know, trapped in one area.”  Harrison 2021  Reference 1  “My family is all in [another country], I feel so alone, no one to help me out, I'm a young first time mum and anywhere professionally I have seeked help I have been either told to go elsewhere or been ignored entirely.”  Kolker 2021  Reference 1  “I have my sister, I have my aunt, but because they were moving around, the thing is, they weren’t stopping their life, so I called them on the phone, but they wouldn’t come to me. You know, for us, the community and our culture is, we get together. We do family things. We meet every time. Every week we are together, we are eating, but now that was disconnected because they would not come, they are moving around, they have kids in daycare. So that was like gone because of the COVID. So we talked on the phone. That supposedly was support, but not as much as I wanted.” [P3]  Meaney 2021  Reference 1  “Feeling pretty unseen and uncelebrated by most friends and family as we anticipate the arrival of our ﬁrst child, thanks to the pandemic and social isolation.” (P394; USA, 30–34 yrs, nulliparous, third trimester)  Mizrak Sahin & Kabakci 2021  Reference 1  “To be home alone during the day, I would normally go to my family, but I mean, in the process, everyone is staying at home . . . and no one can come to our place because of the virus, you know, because of my pregnancy. I'm on my own.' (P1)  Reference 2  “My family would visit me as they live close, and I normally go every three or four weeks, it has been almost two and a half and three months since I went, which affects me.” (P15)  Reference 3  “I am alone now, and there will be no one at birth. My mother would support me but she stayed there in Istanbul, and the bus service was stopped.” (P9)  Rhodes 2020  Reference 1  “I would like someone to be representing our needs to politicians so that priority is given to measures like “bubbles” that would help new families SO much rather than people being allowed to have a cleaner come!” [Free text]  Anderson 2021  Reference 1  “My family support networks are far away, they were going to come today, that can’t happen, I don’t know what services are available to me, and I am worried about that. There are probably more and more women who are at risk and feeling isolated in their homes”  Linden 2021  Reference 1  “But then of course I could not travel during the pregnancy anymore and my mother lives in Germany … I was locked up during my pregnancy… so the social part, that you do not get support from the family either [makes] you feel isolated and you feel like ‘I have no friends, we have nothing, how are we going to cope with this” (Interview no. 3, I-para).  Ollivier 2021  Reference 1  “I feel everything is harder than it needs to be, and the joy is taken out of so much because I can’t share the experiences and family ” | Many women described that they valued peer support from other mothers during pregnancy and post-partum, together with intergenerational support from parents. Some multigravida women desired access to additional support to help with childcare… In their responses, women explored the potential impact of social isolation and access to support on their mental  wellbeing. [Atmuri 2021]  Due to the restrictions imposed by the ‘stay-at-home’ order, participants were unable to rely on support in person from family and friends during their child’s ﬁrst few months of life [Costa 2021]  Most commonly, perinatal women reported that COVID had led to reduced social support from family and friends to assist with baby care, in the postpartum period… Some positive changes to support were also reported, as some women reported that partners and family were around more to help out… Further, three postpartum women and one pregnant woman worried about not having the expected support from family, friends, and loved ones during the postpartum period and were concerned about their ability to care for a newborn without them. Finally, some perinatal women worried about the frequency of and access to prenatal care and postpartum resources… Impact statements in this category pertained to changes in social and practical support, and socialization. Specifically, most women reported that COVID had negatively affected their ability to receive social support. Perinatal women reported reduced emotional and practical support, as well as a significant decrease in socialization with family and friends due to social distancing. Some positive changes to support were also reported, as some women reported that partners and family were around more to help out. [Green 2021]  Isolation from friends and family continued when home from the hospital… The restrictions and isolation from others also meant that participants were unable to take the opportunity to rest… Participants felt the absence of face-to-face support… [Riley 2021]  The government-imposed social restrictions impacted on participants’ experience in the home, with reduced visitors and loss of support further exacerbating the sense of aloneness... Participants spoke of how the social restrictions in the home and in health care settings reduced the social support received from family and friends and how this negatively impacted their experience… The constant changing social restrictions resulted in changed sup-  port situations… Yet another explained that her plans needed to change as her parents were elderly and in an at-risk group [Sweet 2021]  For women who had had their babies during initial set of lockdown restrictions, it was not uncommon for mothers to express sadness concerning the lost opportunity for extended family to bond with the new baby, and the inability to share infant milestones with family… This was especially difficult during the early postnatal period whereby women would have otherwise been receiving much needed emotional and practical support from friends and family during this transition to parenthood… Other sources of difficulty included lack of parenting support from social networks that would have been accessible in the absence of COVID-19 imposed social distancing restrictions. [Jackson 2021a]  Other criticisms included having a desire for social  support bubbles to have been established for new parents earlier in pandemic restrictions [Jackson 2021b]  In contrast to participants who felt that being home with other children reduced pressures, others found that having older children home, needing to home-school and not being able to get out and about really threatened their ability to establish breastfeeding. This was especially true for those with partners working outside the home still and was exacerbated by not being able to rely on family support… Additionally, in contrast to those who felt shielded from negative family interference, others felt isolated and missed the emotional support they would receive from caring and supportive relatives. [Brown & Shenker 2021]  Many participants believed COVID-19 had been a blessing but also a nightmare for their family.  As noted in the preceding section, the ﬁrst participant contrasted being free from the pressure to be a “super mom” against feeling as if she was living a nightmare. Using strong sentiments, she said, “In bad moments I just want to cry because of the pandemic. I am so sad that I can’t share her [the baby] with anyone”. This mother believed that sharing the experiences of having a new baby was important and felt sadness that she was not able to do this on account of the public health orders in place. Feeling ‘so sad’ revealed the meaning this experience held for this mother and that it was very signiﬁcant. This was further emphasized as the participant gave more context to her experiences. [Joy 2020]  Interviewees’ narratives suggest that having a newborn at home during the pandemic was challenging for most women (Box 2), and these challenges were compounded by lack of support. This was amplified for those with pre-existing mental health conditions and those recovering from difficult births. For example, the first 2 quotes in Box 2 convey the experience of new motherhood during the pandemic for some women with pre-existing mental health conditions. [Rice 2021a]  Participants also voiced concern about feeling isolated during the postpartum period as a result of having to be home alone with the infant without the full range of support from family and friends. [Dove Meadows 2020]  The advice and reassurance from health professionals was particularly missed by first-time mothers and those who were cut off from family support by distance or legal restrictions on contact with other households because of Covid-19. [Harrison 2021]  Participants reflected on how the public health mandated separation from family resulted in missing out on much needed support for them and the baby. [Kolker 2021]  During the pandemic, pregnant women relied heavily on informal supports reporting that they depended on their partner, family and friends to help alleviate stress. However, as illustrated below, women reported how they felt that this support was limited. [Meaney 2021]  Family support is important in pregnancy and postpartum process in Turkey, and usually the mother of the pregnant woman lives with her in this process and helps her to make the process more comfortable. However, due to the pandemic, the families of many pregnant women were unable to live with them due to national measures such as travel restrictions, the ban of going out of for people over the age of 65, and the process was more difﬁcult for these pregnant women. [Mizrak Sahin & Kabakci 2021]  In practical terms, the loss of informal childcare support (such as grandparents babysitting) meant that mothers no longer had time to themselves for mental revival or physical exercise. Several postnatal respondents remarked that their intended postpartum fitness regime had been put on hold as a consequence of having no help with childcare. [Rhodes 2020]  Participant 7 described how difficult it was to be separated from her family and local friends, comparing her emotions to the experience of drowning…  Stay-at-home orders, isolation requirements, and limitations on in-person gathering limited women’s access to informal support people after birth. This was compounded by the temporary closure of schools and daycare facilities, leaving women to care for their children and newborn without external support people, causing exhaustion, fatigue, and isolation… As all participants had already been separated from their extended family before COVID-19, as a result of forced migration and resettlement, their sense of loss was compounded by also not being able to see local friends after birth, who had become a kind of chosen family. (2^nd^/3^rd^ paras are author interpretation only) [Stirling Cameron 2021]  Pregnancy was seen as a time when women would normally seek out connection with others (friends/ family/other pregnant women). In this context, the isolation and loss of social contact during pregnancy was experienced as an acute loss… Planning arrangements around the birth were fraught as women grappled with how to plan for a parent to help look after existing children during labour or for support with the new-born, or if their partner became ill. (second para is author interpretation only) [Anderson 2021]  Social isolation was extra hard on women with parents belonging to a risk group or for those with family abroad as national borders were closed eliminating the chance of any physical meetings [Linden 2021]  Dominant social discourses surrounding mothering tend to continue to place value on the nuclear family who may not have support from extended family and new mothers are expected to be ‘super moms’, glorifying a ‘be it all, do it all’ parent when, in reality, such expectations of the ideal parent can be impossible, harmful, and damaging. The effects of a lack of in-person socialization on the mental health of mothers and babies is clearly an important issue… One parent described their experience of feeling isolated, this quote exempliﬁes the added stress of feeling like parenting was something that they had to navigate alone while emphasizing the importance of supports and the shared challenge, but also joy, of raising a baby [Ollivier 2021]  All mothers reported grief and sadness related to social distancing. They reported planning to have their family members involved in their child’s life and helping with newborn and household duties during the immediate postpartum time at home. Two mothers in particular talked about friends and families making window or door visits. Janis said these visits made her cry and she told friends and family members to stop coming because it just made me feel worse. Stella described glass door visits as being heartbreaking. (author interpretation only) [Spatz & Froh 2021] | **[Inability to interact with family and friends physically]** Women expressed their sadness in the lack of support from family and friends due to public health restrictions. Women who had family overseas or interstate described challenges in having supports readily available. However, local restrictions also meant that women could not have their support networks physically close, only being able to meet outdoors, when restrictions eased. Some women described how this impacted their cultural lifestyles, especially for families who were used to being together often for meals. As a result they felt increased social isolation which negatively impacted their postpartum experience. (Atmuri 2021, Green 2021, Riley 2021, Sweet 2021, Jackson 2021a, Joy 2020, Rice 2021a, Stirling Cameron 2021, Dove Meadows 2020, Harrison 2021, Kolker 2021, Meaney 2021, Mizrak Sahin & Kabakci 2021, Anderson 2021, Linden 2021, Ollivier 2021, Spatz & Froh 2021)  **[Need for practical support]** The inability to access their postnatal supports, people they would have relied on prior to the pandemic, e.g. grandparents and friends, to help mothers was a huge loss for mothers. Mothers described desperately needing both the emotional and the physical support for reassurance, as well as providing some relief for tired mothers. For mothers with older children, they manifested their need for their support networks to help them manage the requirements from older children and their newborns. Many described being exhausted and felt that their mental health was deteriorating. Some mothers reflected on possible alternatives to public health restrictions, seeking exemptions from the isolation and social distancing rules, and hoping for governments to extend “social bubbles” in countries and states that implemented this, to women with newborns and allocated family members. They criticised governments for not considering pregnant and postpartum women in their plans for a COVID-safe return to society. (Atmuri 2021, Costa 2021, Green 2021, Riley 2021, Sweet 2021, Jackson 2021a, Jackson 2021b, Brown & Shenker 2021, Joy 2020, Rice 2021a, Rhodes 2020, Anderson 2021) |
| Impacts on own mental health4.1. Managing anxiety due to virus-related fears and concerns | | |
| Davis 2021  Reference 1 - 0.18% Coverage  “There was no face-to-face and just calling someone on the phone, it felt it was a bit impersonal. So, I relied heavily on my family to deal with my, you know, my anxiety and my outbursts and just being frightened for those few weeks.  Farrell 2021  Reference 1 - 0.46% Coverage  “I think it is a more high anxiety thing. […] So going anywhere is stressful, especially where there is sick people. So I guess when I have to go anywhere, whether it is for the genetic testing or not, I don't think that is a deterrent for me having to go to the hospital and considering if I was going to have the genetic testing […], or to go to the hospital to have testing done. There is definitely a level of stress that comes along with that”  Reference 2 - 0.55% Coverage  “I want to do what's best for me. But, at the same time, my anxiety about being in a doctor's office or being in a space where I know there are potentially sick people nearby […] I didn't want to be there. It wasn't like, ‘Oh, I feel so comfortable and so safe here. I want to take my time, whatever and whatever.’ It was more like, ‘Ok. Let's get this done so I can leave because I don't want to be there’”  Reference 3 - 1.00% Coverage  “The nuchal translucency and the blood draw, the screening test… for that I was still pretty nervous about the health of the fetus. I was more worried about finding out by myself”  Green 2021  Reference 1 - 0.07% Coverage  “Due to COVID-19, we have really limited how often we go out, as I am anxious to leave the house unnecessarily”  Reference 2 - 0.07% Coverage  “I feel that COVID-19 has brought a sense of uncertainty for the future, and this has fuelled my anxiety”  Jackson 2021b  Reference 1 - 0.40% Coverage  “I never had anxiety before…I never, I’ve never suffered with it. But even if I like, if I see [baby]- if I go to like if I pop to the supermarket now, I get all like panicky. I feel like I’ve got to rush in and rush out as quickly as I can. I dunno if it’s ‘cause it’s just odd when you go in there now and the one-way systems and all like being a bit weird or I don’t know if I would’ve been like that anyway because I’m away from the baby, but I suppose I wouldn’t’ve overthought it before.”  John 2021  Reference 1 - 0.21% Coverage  “COVID actually is really stressful mentally…you know. I can’t go anywhere, nobody can come, which is in a pregnant woman, uh, isn’t… I’d love to go somewhere, but we couldn’t go anywhere because of the restriction.”  Reference 2 - 0.22% Coverage  “I found it very hard when you’re coming to the country without knowing anyone and the coronavirus, everyone is…doing lockdown so it was very…difficult, I was very depressed. I was very anxious, yeah, umm, I feel worried a lot.”  Kolker 2021  Reference 1 - 0.13% Coverage  “At the beginning it felt kind of scary…I didn’t want to take the baby to the grocery at all at first, so at the beginning my husband was doing all the grocery shopping.”  Reference 2 - 0.16% Coverage  Traveling on public transit also caused increased stress due to the perceived risk of exposure while pregnant. One participant shared, “I felt really stressed out... I was having panic attacks on the subway.”  Reference 3 - 0.08% Coverage  “I was very stressed about that in my pregnancy, very, and very upset at the thought of being alone.”  Reference 4  “Initially when the pandemic started, it was obviously pretty scary, because I didn’t know much about the effects that COVID could have on pregnant women, on their babies.” [P2] Another participant also voiced her concerns about the potential risks to baby, “I remember, with the COVID stuff, feeling stressed in March, thinking could this be risky to the baby, or if I got sick would I go into pre-term labour or, you know, that kind of stuff.”  Reference 5 - 0.37% Coverage  “I’ve been trying to avoid looking at the numbers as of late … Looking back I wish I hadn’t known. I think it would’ve been better for me to be kind of ignorant about it, because like it just gave me more anxiety than I was already feeling.”  Reference 6 - 0.22% Coverage  “I remember being quite pregnant, like around 33, 34 weeks pregnant, and we’re in the hospital and no one really knowing kind of how dangerous it was or what to do.”  Reference 7 - 0.21% Coverage  “So the unknown was really, really challenging. […] like no children have passed away from it but it’s really – I don’t know, it’s that unknown. It’s that fear of not understanding – like it hasn’t been around for so long that you understand and you can quantify what it is”  Ollivier 2021  Reference 1 - 0.30% Coverage  “Anxiety also plays a big part in this diﬃcult time. As a new mom you are already so nervous, so adding a pandemic to that pile of anxiety and worry”  Reference 2 - 0.23% Coverage  One participant described feeling extremely nervous and anxious, saying it was “almost enough to put you in a padded cell”. This quote represents the experiences of many mothers in our study who expressed having mental health issues.  Rhodes 2020  Reference 1 - 0.10% Coverage  “Worried about the world – thinking ahead how’s it going to be – a massive change that we are all going to have to go through – it’s quite an anxious time really.”  Reference 2 - 0.26% Coverage  I have become lazy – I can’t be bothered…lethargic. I don’t feel hungry because I am not moving enough…I can’t be bothered with cooking or cleaning now….the boys have lost their appetite – one boy is losing weight….they stay in their PJs all day, say there is no point in brushing their teeth as no one is going to smell their breath….the boys are worried that when I go to hospital I am going to die.  Reference 3 - 0.10% Coverage  “I’m scared of the unknown- how much does it affect pregnant women and unborn babies, will my new-born be at huge risk?”  Reference 4 - 0.07% Coverage  “I have had days when I break down and cry….my entire day is lying on the sofa or in bed doing absolutely nothing.”  Rice 2021a  Reference 1 - 0.79% Coverage  “I think I had a bit of postpartum depression. I do have some anxiety as well ... even before pregnancy. So, the first 2 or 3 weeks I felt like I was going crazy. I felt like I was going insane because I was so ill, I couldn’t stay awake for long, I was really weak. My incision wasn’t healing properly. At this time, my boyfriend was trying to work from home, and he wasn’t sleeping and I wasn’t sleeping. I couldn’t reach out for help from my mom. She couldn’t even come over to my house to help me out. So that was super hard for us. It was hard not being able to reach out and have the family there when you needed them the most.”  Rice 2021b  Reference 1 - 0.85% Coverage  “I was very, very anxious leading up to the birth. Just because everything was changing, you didn’t know what was happening, the hospital policies kept changing. At one point the hospital I was delivering at said your partner could only stay for two hours after the delivery. That made me really upset and I didn’t know if I could end up having a c-section or what would happen. And then, I also saw what was happening in other places like in New York and Montreal where they had no partners at delivery. I was worried that it was going to get worse and that was what was going to happen. I ended up being induced (…) [and] that definitely was partially pandemic related. I think my doctor saw that I was really anxious. I saw her for that 36-week checkup in person and she offered it. She said “I’m on call. When you’re over 38 weeks, if you want an induction, I will put you on the list.”  Anderson 2021  Reference 1  “I don’t care what anyone says, I don’t want to get sick, I hate to sound dramatic in case you’re writing down our worst fears, my worst fear is that I lose my husband”  Reference 2  “I personally I really don’t want to get this, I am even holding back tears now talking to you. No I just I don’t even want to think about what will happen if I don’t follow the procedures and the protocols that they have set in place, because I am really worried about getting it, and especially getting it whilst pregnant”  Reference 3  “I don’t feel as worried about contracting it in a way, although obviously I don’t want that and that would be awful, I am not a high risk person in terms of my health normally so I don’t feel unduly worried about that, I just feel really worried about the capacity that we have.”  Reference 4  “if it’s something I could have controlled. If I can’t control it it’s not… that’s just that, but if I could have just not gone out a few times I would have felt really guilty.”  Reference 5  “I am flitting between a gut feel which is I will be fine, I have got a very low risk pregnancy and generally fit and well, but feeling guilt just for going out on a walk, and that’s not a happy place to be in.”  Stirling Cameron 2021  Reference 1 - 0.39% Coverage  “I had to take all the precautions measurements and put on a mask and gloves. It was really hard. To go to the hospital a few days after birth, all by myself and to leave my baby at home because nobody is allowed to be with me was really very hard. I was thinking that even if I die, I will die alone. It saddened me”  Reference 2 - 0.28% Coverage  “It was hard because I didn’t know how the situation was in the hospital, I was scared that the hospital might be an epicentre of the pandemic. I was scared that the virus spreads easily in the hospital.”  Reference 3 - 0.12% Coverage  “I was scared of COVID. I was scared over my children’s health and because I had recently delivered, I was afraid of my last baby’s health.”  Aydin & Aktas 2021  Reference 1 - 0.19% Coverage  “I am very worried about my baby because if I get infected, I cannot take medication. What can I do, am I at risk of miscarriage? Or if something happens to me, can the baby survive?”  Reference 2 - 0.11% Coverage  “I am very worried … I cannot explain … I am very scared that something bad will happen to my baby and me.”  Reference 3 - 0.20% Coverage  “I am a nurse … I worked in the hospital until I was given administrative leave, so my adaptation to pregnancy was difficult. I thought that what if the virus infects me and I lose the baby…”  Reference 4 - 0.27% Coverage  “Of course, it has … There were deaths. A worldwide pandemic has been declared. I was worried about going out, and I didn't want to go anywhere … Other than my general health, I am also pregnant, I have a baby inside me. I have to protect it too, so I stayed home….”  Fumagalli 2021  Reference 1 - 0.13% Coverage  “I asked to give birth at home [...] I was afraid of the hospital because I had never been admitted to hospital before, I had to give birth and there was an ongoing global emergency.”  Reference 2 - 0.14% Coverage  “It was very hard cause when I was breastfeeding I was always afraid of holding her for a little longer for a cuddle despite the mask, gloves and washing hands . . . I wanted to avoid the contagion”  Mizrak Sahin & Kabakci 2021  Reference 1 - 0.19% Coverage  ‘We were so scared whether we heard it on social media or on television and talking to friends, you know, everyone was  spreading all this hearsay nonsense information…’  Reference 1 - 0.11% Coverage  ‘I could not help being anxious at ﬁrst; I thought if I were affected, my baby would be affected.’  Spatz & Froh 2021  Reference 1 - 0.27% Coverage  Janis reported that she was afraid that when she went out for her appointments, she would bring germs home which made her fearful of in person visits. Janis stated when she went to the paediatric visit, she did not sit down and she immediately undressed herself and child upon return home and showered herself and bathed the baby.  Sweet 2021  Reference 1 - 0.28% Coverage  “I think it was just that anxiety. … [so I was] trying to ﬁnd out as much information as I could. …I think there was a lot of anxiety on my part …I’d ask questions like ‘is this going to aﬀect my pregnancy?’ and they’d say, ‘we just don’t know’ ”.  Reference 2 - 0.08% Coverage  “I think it’s because it was unknown. …I was feeling anxious and scared”  Linden 2021  Reference 1 - 0.42% Coverage  “I just think it was more this general concern, the uncertainty, is this dangerous? […] what would happen if I got infected, is it dangerous for me? Is it dangerous for the child? […] what would happen if you ended up in intensive care and so on…”  Reference 2  “We are not going to meet anyone […] we felt pretty quickly on our daughter that she did not like the situation of just being at home, at home, at home […] It is not so easy to explain to a barely three-year-old” (Interview no. 7, I-para)  Panda 2021  Reference 1  “Going out and doing the food shopping I stopped entirely I was kind of not really getting out because being pregnant I was very conscious that I had to be that little bit more careful. ” (ID 13)  Silverio 2021  Reference 1  “I’d also at that point spoken to my Midwife and they said that both of us would have our temperatures checked when we arrived. So, I was actually more concerned about my husband catching COVID, because obviously if I had it, they would [laughs] let me in regardless, whereas if he had it, he wouldn’t be there for the birth. So, we had been really, really anxious about going out, and I think by my 30-week appointment we hadn’t been out for at least a month, we hadn’t left the house.” (Participant-023)  Atmuri 2021  Reference 1  “You do worry. You are concerned that everyone’s doing the right thing, can I go to the shops, am I putting the baby at risk?” FR  Reference 2  “I’m giving birth in a hospital where sick people are. And it’s probably – if people have COVID – they would be in the hospital. Just being in that same environment with a newborn is deﬁnitely a bit daunting.” MN.  Reference 3  “I actually stopped going to work . . . I’m a kindergarten teacher and it was just recommended that given the limited research on what would happen.” EW  Keating 2021  Reference 1  “It’s harder to know the right thing to do is…what is being responsible, what is being paranoid” P9  Sweet 2022  Reference 1  “From my window I could see into the COVID testing clinic … and I could see people coughing– I thought those people all had the virus and that’s why they were there … I was scared to leave the hospital thinking what if I walk past someone who’s infected and myself and my only baby gets sick” P3.  Dove Meadows 2020  Reference 1  “Exposure to my children, you know, I have a 16-month old and then bringing home a newborn whose, you know, immune system is brand new ... just the unknown of what can happen is pretty scary in pregnancy”  Reference 2  “If a pregnant person catches the COVID-19 then, of course, that would be extra stressful because she’ll be sick and, you know, worried about her life and the life of her child and wondering if it will pass on to the next baby ... emotionally like that’s a big toll on a woman even to think about, you know, her baby being at risk.”  Reference 3  “I don’t want the baby to catch the coronavirus ... When they’re first born, they don’t really have an immune system ... I understand that there are like procedures, you know, medical instruments ... Iguess because I have to work a lot in the environment, so I see what the environment is ... I don’t know if I wanna be on the receiving end of that.”  Reference 4  “You don’t know who’s been around who. You don’t know, you know, who you going to be in the room with that maybe been around somebody.”  Meaney 2021  Reference 1  “Thinking I might be sick (speciﬁcally with the possibility that it may be COVID-19) at any small sign or symptom and how that it will affect my baby. Also concerned that any weird feeling is a sign of miscarriage.” (P6; USA, 20–24 yrs, nulliparous, second trimester) | The greatest consternation from all women was the frustration with the lack of service access. However, this was more frequently reported by women in the ‘low’ group. Physical health services, such as ultrasound scans, took a long time or were replaced by telephone or telehealth consultations. Many of the women understood the rationale for this but reported that delays or cancellations heightened anxiety. [Davis 2021]  Heightened anxiety associated with the decision to undergo prenatal genetic testing… Participants who sought prenatal genetic testing despite the concerns of COVID‐19 reported the anxiety they experienced in the process. Participants were conscious of the risk of exposure to the virus when presenting for testing. For these women, the process of presenting for testing was a source of anxiety… Participants discussed another significant concern: fear and anxiety of being alone during the testing process. As a result of visitor restriction policies, many participants underwent ultrasound‐based screening and diagnostic testing procedures without a partner or support person. This was a concern for women who wanted to share the experience of seeing the fetus with a partner. Yet, for many, the concerns pertained to the possibility of learning about a fetal abnormality or demise by themselves during periods when visitor restrictions were in place. For participants in this study, the fear of being alone was unexpected—something most did not anticipate or fully realize the implication of until the time of the ultrasound appointment. [Farrell 2021]  The most common impact statement reported by participants was the impact of COVID on the ability to carry out normal routines and day-to-day practices. Specifically, most perinatal women reported being unable to leave the house or leaving much less often due to COVID fears and restrictions… Perinatal women also reported that COVID has had a negative impact on their mental health and that the uncertainty of the pandemic had exacerbated pre-existing anxiety. [Green 2021]  For some mothers, COVID-19 related anxieties be-  came disruptive to one’s ability to function in day-today activities [Jackson 2021b]  The detrimental effect of the pandemic on mental health during pregnancy was highlighted by all participants. Women expressed feeling more anxious, fearful and lacking autonomy in comparison with their previous pregnancies. This sentiment was particularly emphasised by the participants who had little or no family members nearby (box 4: Psychological impact, P11; P13). The majority of participants reported that they were not routinely asked about their mental health in relation to the pandemic by healthcare professionals. Fewer still were signposted to appropriate support groups for help. [John 2021]  Some participants created new routines to access food and delegated their partner as the sole person to go out to buy groceries, because of their perceived risk being pregnant or exposing their newborn baby… Traveling on public transit also caused increased stress due to the perceived risk of exposure while pregnant. Another postpartum participant decided to avoid taking the bus all together out of fear for her exposure, “You can’t take a bus because the busses are still packed. There’s no distancing on that. I have to take the baby, I’d rather not.” The feeling of uncertainty was commonly expressed due in large part to the lack of knowledge about the effects of the virus on their health or their baby’s health… Conversely, most participants reported they avoided information overload to protect their mental health. As one participant described, information in the media was causing her heightened anxiety and she found she was better off not knowing… Another participant described her feelings of fear while working in the hospital during the early stages of the pandemic. [Kolker 2021]  A common theme across the participants was that mental health concerns and considerations were at the forefront of their postpartum experience during the ﬁrst wave/phase of the COVID-19 pandemic. While many participants spoke about positive moments with their babies and families, their experiences with their own mental health were overwhelmingly negative. Several participants described feeling depressed, abandoned, lost, drained, irritable, sad, angry, and anxious. One participant referred to the accumulating nature of stressors –that is, related to being a new parent in general and also in navigating a pandemic. The majority of participants stated that their mental health had been negatively impacted for a variety of reasons including loss of socialization, mourning of the parental leave they had imagined, feeling isolated from loved ones, the eﬀects of compounded stressors, or an exacerbation of underlying mental health issues… While many parents were creative in making the best of their situation in trying to remain as social as possible, feelings of extreme isolation were a major theme in this study’s ﬁndings…. Participants described feeling “lost at sea ” (Participant #8) with no one to guide them or to help them ﬁgure out what was ‘normal’ in their journey of becoming a new parent… However, the urgency and meaning of postpartum mental health during a pandemic was not perceived by the participants as being adequately addressed by the government or provincial health services. Mothers felt abandoned and ﬁnding help or support for their mental health was a signiﬁcant concern for participants in our study. Mothers told us they were forced to search for and navigate formal support for their own mental health in ways completely unprecedented. [Ollivier 2021]  The qualitative findings indicated a range of negative emotional states in addition to anxiety and stress, including loneliness, irritability, sadness, and depression. During April, when the pandemic was peeking in the United Kingdom, anxiety over contracting COVID-19 was high. Pregnant women expressed anxiety over the impact their contracting the virus might have on their unborn child. Those close to delivery were particularly anxious about contracting COVID-19 in hospital when giving birth. Indeed, over two-thirds of the pregnant web-based survey respondents (152/235, 64.7%) said that staying safe when giving birth was worrying them more than normal at the moment, and over half (125/235, 53.2%) were worried about staying safe at antenatal appointments. [Rhodes 2020]  Interviewees’ narratives suggest that having a newborn at home during the pandemic was challenging for most women (Box 2), and these challenges were compounded by lack of support. This was amplified for those with pre-existing mental health conditions and those recovering from difficult births. For example, the first 2 quotes in Box 2 convey the experience of new motherhood during the pandemic for some women with pre-existing mental health conditions. [Rice 2021a]  Similarly, Rachel (second-time mom, Toronto), was explicit that her anxiety over the potential to go through childbirth and a postpartum hospital stay without her husband prompted her obstetrician to offer a scheduled induction. [Rice 2021b]  Fear was identified throughout interviews as a motivating factor for adhering to social distancing guidance. Fears focused on worries about catching Covid-19 near the birth date, or the partner becoming ill, as well as general risk to self and baby. [Anderson 2021]  Women were particularly hesitant to visit the hospital, or other healthcare clinics, fearing that it was a hotspot for the virus… Fear of COVID-19 was a severe enough that one participant skipped certain postnatal appointments at the hospital. Others described the sadness that it caused them to have to leave their partners and infants at home and visit the hospital alone… A significant source of anxiety for many participants was the risk of themselves or their family being exposed to COVID-19. Women were particularly concerned for their infant’s health, feeling as though they were particularly vulnerable to the virus. [Stirling Cameron 2021]  More than half of the pregnant women were worried and scared of the risk of infection for themselves and their babies… Pregnant healthcare workers who are also married to healthcare workers stated that their adaptation to pregnancy was negatively affected… Most women highlighted that they had no social life and isolated themselves at home. The statements of some of the women are as follows… [Aydin & Aktas 2021]  Before testing positive, most women perceived the hospital as a frightening and dangerous place during pregnancy, when they were worried about COVID-19 transmission. For this reason, they were resistant to accessing healthcare facilities if not extremely necessary. Prior to testing positive a woman requested a home birth, as she feared hospitals during the pandemic emergency. [Fumagalli 2021]  When the ﬁrst case of coronavirus occurred in Turkey and its spread continued, the pregnant women experienced “anxiety” and “fear” ... The most important reason why the participants experienced  fear and anxiety was that they were pregnant. [Mizrak Sahin & Kabakci 2021]  The mothers all reported various degrees of guilt, concern, and stress related to the pandemic. [Spatz & Froh 2021]  Women described their experience as stressful and anxiety provoking. The constant unknowns left them needing to ﬁnd ways to manage these emotions. [Sweet 2021]  Even if they tried to focus on other things and tried to ignore thinking about COVID-19, the effects of the pandemic were always present in everyday life and were therefore hard to overlook… All women were worried about their partner getting sick with COVID-19 or another infection close to the birth of the baby, and thus not being allowed to enter the hospital during the birth. Therefore, some families decided not to take any risk of being exposed to the virus and decided to isolate themselves during the last few weeks of pregnancy. Some women took this in their stride and thought of it as a way to relax before the birth, whilst other women expressed that the last few weeks of the pregnancy were very stressful stripping away the peace and quiet that they would have needed to prepare for the arrival of their baby. Being isolated at home was especially stressful for families with small children who did not understand why they could not attend day-care or see their friends. [Linden 2021]  COVID-19 associated restrictions prevented women  from seeking and having face-to-face access to the support of other mothers, female friends and joining mother and baby groups. This postpartum isolation was an extension of antepartum isolation where many women described conﬁning themselves to their home while pregnant to protect themselves from COVID-19 transmission. Although most women were accepting of this as part of the reality of the pandemic, women were impacted both emotionally (sadness) and physically (being extra careful and isolating) as a result. [Panda 2021]  Service reconﬁgurations extended to the intrapartum period, about which pregnant women were often warned by midwives. Occasionally, the restriction on birth partners meant that non-birthing parents missed the birth of their baby. However, restrictions were often viewed as arbitrary and, therefore, changes to birth plans, including presence of birth partners, were frequently reported as unnecessarily frustrating. [Silverio 2021]  Women were vigilant about the risk of acquiring infection in either the community, hospital or work settings… Some women had ceased their jobs or modiﬁed workplace duties to reduce the risk of acquiring the infection. [Atmuri 2021]  Women received conflicting information about the potential impact of the virus on their health. Many women felt vulnerable and chose to cocoon despite the national recommendation that this was not necessary. While the initial “lockdown” message was clear, the gradual easing of restrictions brought new challenges… Women also feared for the health of their babies and how they would manage to care for other children if they were to become unwell (2^nd^ para is author interpretation only) [Keating 2021]  Participants described varied awareness of their risk of exposure to COVID-19 as they received their healthcare. [Sweet 2022]  Most of these participants feared that their infant would acquire COVID-19 and several of them immediately expressed this concern when initially asked about any worries related to the virus. One participant was concerned about her infant and her other child. She stated that she feared… Another described the emotional toll on pregnant women… Another’s concerns again centered around the impact on the infant… Many of the participants feared the possible effects of COVID-19 on themselves. The participants were afraid of the potential exposure during their hospital stay from healthcare personnel, roommates, or others who might have asymptomatic COVID-19. Even though several indicated on the questionnaire that they only worried a little, they talked about their worries during the interview; one was concerned enough to talk to her physician about it. [Dove-Meadows 2021]  Under the theme concerns related to Covid-19 infection (see Table 3), women reported worrying about potential pregnancy loss and/or any adverse pregnancy outcome… When asked to comment on their concerns linked to the pandemic, many of the women reported fears of contracting COVID-19 and what implications that would have for their health and the health of their infants (see Table 3). (2^nd^ para is author interpretation only) [Meaney 2021] | **[Impact on mental health]** Women reported that living through the pandemic, took a huge toll on their mental health. Anxiety, fear, stress, worry and concern were the main stressors that women expressed. The risk of infection and transmission made women worry throughout their pregnancies and early postnatal periods. This meant that many women isolated themselves, remained home and limited interactions with others for fear of infections. This negatively impacted women’s mental health as they were unable to access supports and felt increasingly worried as they needed to access health services for routine check-ups, important scans and genetic testing. Women reported being uncomfortable when attending hospital appointments, which increased their risk of infection, causing much stress and anxiety. For some women, they reported that their mental health deteriorated significantly and had to seek help and methods to cope during the pandemic. (Davis 2021, Green 2021, Jackson 2021b, John 2021, Kolker 2021, Ollivier 2021, Rhodes 2020, Rice 2021a, Rice 2021b, Stirling Cameron 2021, Anderson 2021, Aydin & Aktas 2021, Mizrak Sahin & Kabakci 2021, Sweet 2021, Keating 2021, Sweet 2022, Dove Meadows 2020, Meaney 2021)  **[Unknown impact on mother and baby]** Early in the pandemic, the lack of information and understanding about the impact of the virus on pregnant and postpartum women, their unborn fetus and newborns significantly increased levels of stress, anxiety, fear and worry. Due to the initial uncertainty, healthcare providers could not provide reassurance for concerned women, which ultimately heightened their anxiety levels. The fear and possibility of infection was repeatedly reported across studies and women remained vigilant to avoid contagion. Women who were infected with the virus were anxious and feared transmitting it to their babies, so they took extra precautions in wearing PPE, increased hand hygiene and limited contact to protect their babies. (Green 2021, Kolker 2021, Rhodes 2020, Stirling Cameron 2021, Anderson 2021, Aydin & Aktas 2021, Fumagalli 2021, Mizrak Sahin & Kabakci 2021, Spatz & Froh 2021, Sweet 2021, Linden 2021, Panda 2021, Atmuri 2021, Sweet 2022, Dove Meadows 2020, Meaney 2021)  **[Anxieties related to healthcare services]** There was increased anxiety and fear associated with healthcare settings. The public health restrictions meant that some women were not able to have their support people present for appointments, labour and childbirth as well as the early postnatal period. Many women reported increased anxiety levels leading up to important scans, genetic testing and appointments with healthcare providers and labour and birth as they feared that they would be alone for these events. Additionally, women felt anxious and uncomfortable entering healthcare settings and hospitals that also cared for COVID-19 patients as they felt it was dangerous and feared infection and transmission. (Farrell 2021, Rice 2021b, Stirling Cameron 2021, Fumagalli 2021, Silverio 2021, Sweet 2022) |
| 4.2. Feeling lonely and isolated | | |
| [Jackson 2021a](https://deakin365-my.sharepoint.com/Users/amanda/Downloads/78d56e5e-fa05-4dc4-afda-bf4dc97139fa)  Reference 1 - 0.18% Coverage  “You haven’t got any friends or family that can necessarily come into your home and support you in case, they, you know, also contract it. Carrying it. It’s kind of like, one of them, rock and a hard place erm, situation. So, erm. Yeah. It is isolation”  Reference 1 - 0.21% Coverage  “Obviously you haven’t got your husband or his family [on the ward], and it’s literally like being in a little prison cell.”  Ollivier 2021  Reference 1 – 0.56% Coverage  “I feel my mental health is suﬀering because of missing connection with other ﬁrst time moms” (Participant #8)  Reference 2 - 0.36% Coverage  “We had one meeting with public health before they reassigned our nurse to the COVID team and we were dumped. The breastfeeding clinic cut her tongue tie but did a phone follow up instead of in person due to COVID and dumped us even though we were still having feeding issues”  Reference 3 - 0.20% Coverage  “I also feel as if moms are being treated as second class citizens as they no longer oﬀer 6 weeks postpartum checkups meaning that we are left to decide if things seem normal or not after giving birth.”  Reference 4  Participants described feeling “lost at sea ” (Participant #8)  Reference 5  “Even in a room full of strangers I felt I could ask for help, and now all help is oﬀ the table ”  Reference 6  “I couldn’t really explain how mentally/emotionally exhausted I was”  Rhodes 2020  Reference 1 - 0.17% Coverage  “My maternity leave has not been what I had hoped. I have not been able to access help with the baby from family or friends because of social distancing. I have not had the benefits of meeting up with other mums and have felt isolated and very lonely at times.”  Reference 2  “I just feel like new moms and parents have just been completely left out of the Government’s mindset and their exit strategy really.” [Baby 3 months, age 30-34 years, White British]  Reference 3  “I don’t think I am being supported. The Government should be giving us more information, like where do you go to get your baby weighed…there were 8 weeks when she wasn’t weighed – quite a worry really.” [Baby 12 weeks, age 30-34 years, White British]  Panda 2021  Reference 1 - 0.15% Coverage  “So I think with it being COVID and then going into labour by myself I think that’s what put the fear into me. A combination of being on my own and then with COVID”  Reference 2  “Going out and doing the food shopping I stopped entirely I was kind of not really getting out because being pregnant I was very conscious that I had to be that little bit more careful.”  Aydin & Aktas 2021  Reference 1 - 0.33% Coverage  “I live in a city far from my family … During that period, I was only with my husband, and since he is a doctor, I was mostly alone at home … I am between four walls at home … Is my psychology broken?, Am I okay?, How Do I Feel?, I asked myself many times … Crying and tantrums … I have gone through a deep depression….”  Reference 2 - 0.44% Coverage  “… I think I was depressed during the pandemic. I came to live with my family living in another city in case my husband working at the pandemic hospital would transmit the virus to me. I've always had contractions … I was psychologically affected … I was laughing at everything nonsense because I felt nervous … My parents were asking me why I was laughing for no reason … What are these? I think the symptoms of depression….”  Reference 3  “I have been negatively affected. Because we are both sociable people, being at home for 7/24 started to disturb us…”  Davis 2021  Reference 1 - 0.14% Coverage  “It’s not until you’re in that deep, dark place that you need that help and someone can tell you, whereas if I’d known about it before, I might not have gotten to that point.”  Reference 2 - 0.16% Coverage  “I was really anxious. I did a calm birth course to try and help me just get some that sense of control back in a world where everything was so uncertain. So, I was just really stressed out . . . I just feel like pregnant women weren’t supported through the pandemic . . . You’re just thrown in the complete deep end because you don’t have regular support that you’d normally have.” (CW6_low)  Green 2021  Reference 1 - 0.06% Coverage  “I never get to leave the house, and this has had a negative impact on my mental health”  Reference 2 - 0.03% Coverage  “COVID-19 has left me feeling quite lonely”  Reference 3  “I worry that the lack of social interactions due to the quarantine will negatively impact my mental health because my social supports have been critical for my psychological well-being” [Postpartum #24].  Reference 4 - 0.18% Coverage  I am worrying about my relationships, losing friendships. I feel like everyone is moving on without me during the pandemic, I am not seeing people in person, so I am feeling very disconnected with people and this is going to go on forever and have a huge effect on my relationships  Saleh 2022  Reference 1 - 0.05% Coverage  “Being cooped up in the house takes a toll on you mentally.”  Reference 1 - 0.08% Coverage  “I didn’t expect to be completely isolated.”  Reference 2 - 0.03% Coverage  “A lot of just more isolation.”  Reference 3 - 0.09% Coverage  “I was just waking up, doing the same thing every day. It’s very mental, I get depressed or I feel very down”  Reference 4  “I need to talk to an adult besides my three year old, who yells at me and then the baby who’s nursing 24/7 and I’m actually tandem nursing both of them so there’s been days, where you know I sit, topless on the couch.”  Rice 2021a  Reference 1 - 0.44% Coverage  “I know [that] a lot of women who gave birth around the same time as me, or during when the restrictions were at the heaviest, they suffered from postpartum depression. I know that I suffered big time from baby blues, and I thought, ‘For sure, I’m going to need to be medicated after this, 100%.” When I got home, I think I cried for 2 weeks straight.’”  Reference 2 - 0.46% Coverage  “The isolation was really hard for me. When I look at what has happened in the last 6 months, I had a baby, I was locked in my house, I couldn’t see my mom, I couldn’t see my grandmother, who’s dying. And here I was trying to just pretend that everything was normal for a 5-year-old so that he’s not scared. Yes, it was a hard few months, thinking about it all like that.”  Reference 3  “[My partner] had to leave 4 hours after [I delivered]. It was terrifying. I’d never had a baby before, and I had the whole night and then the whole next day to take care of a newborn by myself after having a hard labour and delivery. I was there alone, I was on pain medication. It was not set up to help women at all. It was more helpful for the staff than it was for the parent. I felt — not by [my partner], but by our health care system during this time ... — abandoned and forgotten about.” (Interview 7)  Reference 4  “The isolation and not being able to have any help from your family or friends — I think that was the toughest part. You kind of get squirrelly and start losing your mind a little. So, at 7 weeks, we just pulled the plug. I was video-chatting with my sister and she [said], “You know, there’s a balance between what’s going on and mental health, too. Maybe we need to start having these conversations.” But my mom lives with her parents, who are in their 90s, and my sister’s husband has a compromised immune system, so there was a lot of, “What do we do?” (Interview 13)”  Anderson 2021  Reference 1 - 0.48% Coverage  “there are times where I just sit in the house and I cry and I am just like I can’t do this, I just need to get out and see a human being”  Reference 2  “I think the isolation side of the lockdown is probably the most difficult bit, which I think I feel perhaps more acutely being pregnant.”  Reference 3  “I think that’s the big thing, it’s like being in lockdown you’re really not able to connect with other people in your situation”  Jackson 2021b  Reference 1 - 0.09% Coverage  “I felt like I’d been imprisoned. I was just, like, sick and tired of being in this living room. I think that was really hard.”  Reference 2 - 0.31% Coverage  “I do feel like we’ve been let down, to be honest. I do feel like we’ve been let down. That and new mums, ‘cause even from a safeguarding perspective, it’s just not fair, that support. I don’t really understand why it’s not [sigh] yeah, why it’s not been prioritised to this extent.”  Reference 3  “I think they could’ve done a campaign, just something to say, you know, we know it’s hard for you, we’re here. This is where you can go. This is what you can do…I think there are mums out there who do feel lonely, and they don’t know where to go.” (Participant 15, T2).  Reference 4 – 0.47% Coverage  “There’s nothing like just meeting people or, you know, just naturally building friendships when you go to baby groups and things. There was people that you’d see at every group and you’d just start to get, you know, become friends because you were always there and things so erm, just- especially some people would really struggle probably to meet new people, anyway. So erm that must be really challenging… It’d be great if they could start [baby groups] again, especially for non-mobile babies ‘cause you could do the social distancing.”  John 2021  Reference 1 - 0.04% Coverage  “I had to go to all my appointments on my own.”  P2 “So COVID kind of, umm, robbed me of that experience which I’m used to back at home, where I used to attend my clinics…So clinics gave you that…that…that, umm, avenue of socialisation, meeting another mothers, new mothers……creating, umm, friendships…and… and also, you know, a continuance after birth”  P3 “I’m not getting enough support for taking care of my baby, she’s crying day and night, and I couldn’t have my husband or anyone because of COVID”  Meaney 2021  Reference 1 - 0.16% Coverage  “The isolation of cocooning due to covid 19 and not really being able to carry out tasks or have freedom to roam.”  Reference 2 - 0.06% Coverage  “Not being able to be around family and celebrate this happy time. Feeing so alone.”  Reference 3 - 0.27% Coverage  “To have had classes as a ﬁrst time mom, not just have classes cancelled and feel abandoned by the system. Medically I know I’m ﬁne which is one thing, but I’ve felt angry that nothing was put in place to replace the classes online. I would have liked more time with midwives to chat and get to know them or a doula that could be there on the day.”  Dove-Meadows 2020  Reference 1 - 0.89% Coverage  “That one hug, that one kiss on the forehead, that one pat on the back from a family member to tell you that it’s going to be okay and you feel it. And it just heals everything overall. So, I think it may impact a lot of pregnancies. I think ... postpartum depression rates will probably skyrocket because everyone’s isolated and you’re stuck in the house with your children and you can’t get out, you know, if you’re stuck. You, you don’t want to do any harm to your kids and you don’t want to, you know, ignore them or have a mental breakdown, but I really feel like the, the rate of it is going to skyrocket. Because we’re all trapped in a way, you know, trapped in one area.”  Riley 2021  Reference 1 - 0.25% Coverage  “I was very tired about the lockdown situation and I was crying half the day because I was tired of having to do things like face time with the family or things like that. So that emotional side I feel…being isolated”  Reference 2  “the isolation, [that] was the hardest ”  Reference 3  “You just expect to be all excited with your partner and have your family come and visit, as if being in [hospital] for ﬁve days in total wasn’t bad enough but to be stuck there [by] yourself ” (P2, Age 32, PP 6 weeks).  Stirling Cameron 2021  Reference 1 - 0.32% Coverage  “The hospital rules are strict during COVID. Visits are forbidden, friends can’t come, and they could not be there to help me. My husband was allowed to visit me twice a day in the hospital. I stayed for two days. I felt really lonely, it was a hard experience.”  Reference 2 - 0.29% Coverage  “It was really hard during COVID. In Syria I had my family… but to give birth here with no one with me?! It was really hard. I needed someone with me, my neighbours, my friends… I felt like I was drowning.”  Reference 3  “Things haven’t changed because even before COVID I didn’t have any friends here.” (Participant 6).  Reference 4  “I was so tired and fatigued that I didn’t talk to anyone. I didn’t have the energy for anything.” (Participant 7).  Harrison 2021  Reference 1 - 0.07% Coverage  “I was ignored when I told them I was in lots of pain and further along that they thought. They wouldn't examine me until I demanded it (condition of moving to delivery suite). I was 8cm  [dilated]!!! … Devastating, I felt abandoned, unsupported, alone like I was fighting a war alone. Ongoing hurt, enraged.”  Reference 2 - 0.13% Coverage  "It was horrendous. I have never felt so alone and vulnerable in my life. I understand it is a busy time for everyone and stressful due to covid, but we weren't treated with care or kindness afterwards - almost like the midwives were forgetting we'd given birth in a pandemic. I was offered no breastfeeding help, which led to damage to one of my nipples and poor milk supply … I was talked down to, scared, alone, sleep deprived, and offered none to very little help. I could not wait to leave the hospital. I firmly believe the aftercare plays a part in my postnatal depression. It was traumatic, and I do not use that word lightly.”  Reference 3 - 0.11% Coverage  “Maternity ward not enough emotional and physical support and understanding, considering women are without support of partner, friends and family at this time with Covid restrictions. Mothers get no respite whatsoever from the care of their newborns, creating exhaustion and anxiety beyond the norm. Physical support is also lacking, such as help in dressing etc., for mums recovering from stitches or caesareans. Midwives seemed unaware of the full true impact of partners being missing from the ward.”  Reference 4  “On the ward during lockdown was hell. I was starved, dehydrated, lonely, sleep deprived and it wasn’t related to my baby. It was like I was in isolation in prison. No music, no tv, no daylight or fresh air. Just me and baby... curtains closed due to covid. Baby who was slightly unwell. Lots of different people touching my baby when they’ve been in contact with covid positive mums. No communication. No explanation. No daddy.”  Reference 5  “Tested positive for Covid-19 two days before labour, so had to give birth in isolation. I had a panic attack during labour because my birthing partner could not be there. Midwives did not have a clue of how to look after a covid positive woman in labour.”  Keating 2021  Reference 1  “It would have been nice to meet other mums who were due their baby at the same time. So all that social element was totally gone”  Linden 2021  Reference 1  “But then of course I could not travel during the pregnancy anymore and my mother lives in Germany … I was locked up during my pregnancy… so the social part, that you do not get support from the family either [makes] you feel isolated and you feel like ‘I have no friends, we have nothing, how are we going to cope with this’…” (Interview no. 3, I-para).  DeJoy 2021  Reference 1  “Then of course with COVID, you know, there’s not nearly as many visitors as there normally would be. So, you kind of feel isolated, I think. And postpartum can feel isolating anyway, even when we had a lot of visitors, so that was some adjustment, as well” (Participant 14)  Sweet 2022  Reference 1  “I was just really worried that babies were getting dismissed [overlooked], … I know when they [the government] decided that football was going to go back on and I still hadn’t had any word that I could take him to the maternal child health face-to-face at all, I was thinking ‘how the hell is football more important than my baby’s health, like what is going on with the world?’ I was really angry at that, that everyone was prioritising getting sport back on the TV. Whereas I’m sitting here with a newborn baby who hasn’t seen a doctor in 6 weeks and thinking, ‘no’”.  Reference 2 – 0.19% Coverage  “… my social life with friends, it was so important for me, their help and support, because like we don’t have family here and I have only friends, but I can’t see them”  Sweet 2021  Reference 1  “I sort of feel like I am going it alone” (P22).  Reference 2  “it’s been very impersonal, being pregnant for the ﬁrst time, I ﬁnd myself not having anyone to talk to and it does feel very alone. I’ve got my husband but he’s not going through what I’m going through” (P25).  Atmuri 2021  Reference 1 – 0.32% Coverage  ”You can’t attend some yoga or birth classes so you can’t meet some other mums as well. You can’t ask advice or experience from them. Because of this, I have to do it by myself.  Reference 2 - 0.25% Coverage  “Attending maternal child health appointments – I don’t know if they’re over the phone or face to face . . . As well moving forward I’m really keen for my mental health to be part of a mother’s group and I’m concerned that those mother’s groups might not be happening.”  Reference 3 - 0.24% Coverage  “I hope I won’t be going through with depression because of less people around. Sometimes you just need family support or even a friend’s support, just to help you out a little bit. Because your ﬁrst time . . . you don’t really have any idea about anything.”  Fumagalli 2021  Reference 1  “I went out without even saying goodbye to my son cause I though I would go back home, then they actually admitted me to hospital straight away and I took it badly, I burst into tears as I thought all of a sudden my other child would not see me anymore.” (W10)  Reference 2  “My son asked me ‘mummy I miss you a lot, when are you coming back?’ [ . . . ] He told his grandma “you can’t change the bed sheets cause then mummy’s scent goes away and I can’t smell it anymore when I go in her bed’.” (W9)  Snyder & Worlton 2021  Reference 1  ‘‘I just feel really isolated so that kind of discourages me from breastfeeding because I’m just so exhausted because like I have formula to make his food I could just pour him some formula and like be done even though I know that’s not what’s best for him’’ (23, Caucasian, unemployed).  Reference 2  ‘‘I want to be able to share it with like people that I know and love and like my friends but like you can’t go anywhere and it’s just like you just feel really isolated and then it’s exhausting’’ | Most respondents felt the initial lockdown was extremely isolating, due to restrictions on between-household socialising, and fears of spreading and contracting COVID-19. Another distressing experience for women at T2 included partners being excluded from maternity suites, which was perceived as an incredibly isolating experience… Most respondents felt that their postnatal experiences had been significantly more isolated and difficult to manage than they speculated it to have been in the absence of social distancing restrictions. Respondents noted that these exacerbated difficulties were felt due to COVID-19 related disruption to emotional and practical support from maternal social networks. Respondents expressed deep sadness and disappointment in being unable to share their life transition through face-to-face interactions with family, friends, and other new mothers. (2^nd^ para is author interpretation only) [Jackson 2021a]  That being considered, participants told us that in-person health visits for essential health services were not always enough even if they were possible during the ﬁrst wave of the pandemic. This left mothers/parents feeling alone, abandoned, and helpless- and those feelings of isolation were incredibly impactful. One participant described feeling extremely nervous and anxious, saying it was “almost enough to put you in a padded cell”. This quote represents the experiences of many mothers in our study who expressed having mental health issues…  Overall, mothers felt that they were missing out on social connections and commonly described feeling isolated, alone, helpless, like a burden to others, and very exhausted trying to independently manage being a new parent without support from others or the comfort in knowing that others were experiencing similar struggles… Indicating the importance of shared experiences, mutual empathy, and an understanding source to share issues with … Public health orders during COVID-19 made accessing information and support very diﬃcult for mothers in our study. Many participants said they were concerned that they did not have the right information about how to best care for their baby, including how to safely breastfeed. With unclear information, mixed messaging, and unknown reliability of certain information sources, parents had to make diﬃcult decisions regarding what sources and who to trust when it came to caring for their new baby. The issue of navigating shifting resources is exempliﬁed by one participant, who stated. In this quotation, the language of feeling ‘dumped’ is powerful and illustrates how many mothers in our study believed health care providers should be available to support them during the postpartum period. Being dumped suggests the ending of a signiﬁcant relationship, a relationship that many mothers trusted as being reliable, consistent, and available. This signiﬁes that mothers value the support of health care providers and believe that the patient-provider relationship is one of importance; without it, mothers may feel forgotten, abandoned, and vulnerable. Those feelings, especially for a new parent, may impede overall health, wellbeing, and parental conﬁdence, which can have signiﬁcant impacts on the infant and family as well. Mothers also expressed that they did not always feel supported and recognized by health care providers, which added to their stress…  While many parents were creative in making the best of their situation in trying to remain as social as possible, feelings of extreme isolation were a major theme in this study’s ﬁndings. Participants described feeling “lost at sea” (Participant #8) with no one to guide them or to help them ﬁgure out what was ‘normal’ in their journey of becoming a new parent… However, the urgency and meaning of postpartum mental health during a pandemic was not perceived by the participants as being adequately addressed by the government or provincial health services. Mothers felt abandoned and ﬁnding help or support for their mental health was a signiﬁcant concern for participants in our study. Mothers told us they were forced to search for and navigate formal support for their own mental health in ways completely unprecedented… [Ollivier 2021]  For many, the loss of face-to-face interaction with family and friends created feelings of isolation and loneliness. For those with pre-existing mental health issues, this could be particularly challenging… A lack of clear guidelines from the government led some respondents to feel that they were a forgotten sector of the population. [Rhodes 2020]  This theme highlights the scenario of being alone which many women experienced during early labour as a direct result of COVID19 and associated practice changes, and the strong emotions this triggered in women as a result. Many women used the language of ‘alone’, ‘lonely’, ‘just me’ and ‘isolated’ when recounting their experience of early labour in the hospital when their birth partner was prohibited from being present. Women spoke about feeling teary, emotional, anxious and fearful when entering the maternity hospital on their own… Furthermore, COVID-19 associated restrictions prevented women from seeking and having face-to-face access to the support of other mothers, female friends and joining mother and baby groups. This postpartum isolation was an extension of antepartum isolation where many women described conﬁning themselves to their home while pregnant to protect themselves from COVID-19 transmission. Although most women were accepting of this as part of the reality of the pandemic, women were impacted both emotionally (sadness) and physically (being extra careful and isolating) as a result. (Last para is author interpretation only) [Panda 2021]  Some of the women living with their nuclear families indicated that they got into depression because of the lack of social support and loneliness during the pandemic… Among the reasons why those whose spousal relations were negatively affected were economic reasons, having to spend a lot of time together or their husbands’ not paying attention to hygiene rules are as follows. [Aydin & Aktas 2021]  Some women recognised that they needed mental health support but were unsure how and where to access it... The lack of clear pregnancy communication was a source of considerable frustration for most of the women in both the high and low groups. [Davis 2021]  The majority of impact statements in this category noted that COVID had led to an overall increase in negative emotions (e.g., stress, depression, loneliness, and unease) for oneself and others. Perinatal women also reported that COVID has had a negative impact on their mental health and that the uncertainty of the pandemic had exacerbated pre-existing anxiety… Supports [2 (8%), 7 (15.9%)]. Worries in this category were related to a potential lack of social and medical supports because of COVID. Specifically, postpartum women endorsed concern about being unable to see family and friends and being unable to make and maintain relationships with others. A few perinatal women also worried about being unable to access medical care and resources as readily. [Green 2021]  Mental distress was the most common theme across the transcripts. Mental distress was described best by the participants as hard, tough, lonely, depersonalized, anxious, terrified, paranoid, fear, depressed, isolated, judged, guilt, shame, and baby blues. One woman summarized it best that she felt she was “left holding the bag on the whole thing!” The common theme here was that many of the women in the study expressed a good deal of emotional distress, some to the point of seeking professional support and medication. Mental health was recognized when women were  forced to not only overcome but learn how to live a new normal and through this growth, they found inner strength and well-being. Mental health was tied to proximity of support from others either physical (bringing meals) or emotional. [Saleh 2021]  Interviewees’ narratives suggest that having a newborn at home during the pandemic was challenging for most women (Box 2), and these challenges were compounded by lack of support. This was amplified for those with pre-existing mental health conditions and those recovering from difficult births. For example, the first 2 quotes in Box 2 convey the experience of new motherhood during the pandemic for some women with pre-existing mental health conditions… many interviewees eventually did allow support persons into the home. This speaks to a third theme that was common across many interviews: seeking out help even where public health policies did not permit it (Box 3). For most interviewees, this decision was accompanied by stress about the risks associated with close contact and was usually done only when the mother had become desperate. [Rice 2021a]  Psychological capability includes emotional ability to adhere to social distancing. While some women reported positive lockdown experiences (e.g. family time at home), many reported feeling isolated, low, and suffering loss of joy, which impacted on their perceived ability to sustain the behaviour. This was particularly acute when living alone and fully shielding… Pregnancy was seen as a time when women would normally seek out connection with others (friends/ family/other pregnant women). In this context, the isolation and loss of social contact during pregnancy was experienced as an acute loss… Pregnant women living alone (or only with small children) were particularly vulnerable to isolation and mental health effects. (Last para is author interpretation only) [Anderson 2021]  For women at T2, there was a great sense of frustration around not being able to lead a normal postnatal lifestyle… Many participants reported struggling with the long-term effects of social isolation and restrictions on independence… There was a consensus that women had been over-looked and marginalised by the Governmental response to the COVID-19 pandemic, which left women feeling abandoned and alone… Many of the participants acknowledged that the perinatal period is a particularly vulnerable and disruptive time in a woman’s life and expressed that they would have liked a more active Governmental response to informing new mothers of where to find support during this difficult time. [Jackson 2021b]  Most women reported feeling isolated during their pregnancy due to features specific to the SARS- CoV-2 pandemic. This was particularly a problem for those who felt that they would have benefitted from the presence of a companion when important information relating to their pregnancy was being relayed to them (box 4: Isolation, P5). Although most women were understanding of the limitations posed by the pandemic, a significant proportion expressed loneliness exacerbated by not being able to engage with their usual pregnancy support networks, and did not feel that virtual groups mitigated this effect [John 2021]  Although policies varied at national and subnational level, the majority of public health measures implemented by Governments restricted women’s movement from their households and their contact with those outside of their direct households. Consequently, many women reported feelings of isolation and loneliness during their pregnancy … Under the theme maternity care impacted by COVID-19 some women reported dissatisfaction with changes to the maternity services which were implemented to reduce the risks of transmission of the virus. Women reported, “how the virus has ruined everything and changed maternity care”, whereby routine antenatal appointments and antenatal/parenthood preparation classes were postponed, cancelled or telemedicine clinics were provided in lieu of in-person appointments. Women reported that antenatal care is “vital” with antenatal classes considered “an essential service” that needed to be promoted in order to empower women to maintain their health and wellbeing during pregnancy. [Meaney 2021]  Participants also voiced concern about feeling isolated during the postpartum period as a result of having to be home alone with the infant without the full range of support from family and friends. [Dove-Meadows 2021]  Prolonged isolation from friends and family led to considerable emotional distress… Isolation, felt as a result of the restrictions to reduce the spread of infection, was challenging for participants. Whilst friendships were formed with other women when in hospital, the extended stay and separation from friends and family was diﬃcult for some participants and did not match their expectations of their postpartum experience. [Riley 2021]  Visitation after birth was also limited, which was difficult for several participants who had hoped they could see their families or friends after delivery. Participant 3 described how emotionally challenging and isolating it was to not have her partner visit after her birth… Participant 7 described how difficult it was to be separated from her family and local friends, comparing her emotions to the experience of drowning…. Several other participants had limited social connections even before COVID-19. For these women, nothing had changed between prior births in Canada and their birth during COVID-19. These women were already extremely isolated and lonely, having been separated from their family in the Middle East, and having limited or no support people in Canada. Their pre-COVID postpartum experiences were no different than the isolating periods women endured during the pandemic lockdowns… Another participant described the social withdrawal that she felt while postpartum during COVID-19 as a result of the physical exhaustion she was feeling. [Stirling Cameron 2021]  The lack of clear pregnancy communication was a source of considerable frustration for most of the women in both the high and low groups. [Davis 2021]  Some women reported that when they had described their contractions and asked to be examined to establish whether they were in active labour, midwives had disbelieved them, and continued to exclude the birth partners. This had resulted in some partners missing the birth of their child… However, many more women described the intense loneliness, anxiety and emotional strain of being separated from their partner shortly after birth (often within an hour), and the sadness this had caused their partners who missed out on their child’s earliest days. Many women said they had found it hard to cope physically without the support of their partner and other visitors, particularly if they were recovering from a difficult birth or a caesarean section. They described the staff on postnatal wards as too busy to give practical help with baby care or to assist post-operative mothers with personal care such as showering. Some first-time mothers whose antenatal classes had been cancelled were disappointed that staff were not willing to educate them about baby care and breastfeeding… By contrast, many women summarised their experience of postnatal care in the community as feeling “forgotten” or “abandoned”, and described the reduction of professional support as increasing their stress as new parents. (3^rd^ para is author interpretation only) [Harrison 2021]  Women described the “new normal” of a pregnancy during a pandemic. They found it difficult to engage with other pregnant women which added to a sense of isolation. [Keating 2021]  Although mothers connected to other mothers at the unit, some sort of emotional loneliness arose through the lack of a present father with whom she could share common experiences and have the possibility of becoming a family. (Author interpretation only) [Kyno 2021]  A major strategy to decrease the spread of COVID-19 was to practice social distancing. Women described how their social circles shrank drastically in a matter of a few days and how social distancing amplified feelings of loneliness in their pregnancies… Feelings of sorrow and tiredness due to the pandemic changed on a daily basis, which was difficult to manage for some women as they did not know in which mood they would wake up in the mornings. This was especially hard for women who worked from home as they expressed that the isolation of only being in the same space, seeing only very few people could affect them differently from day to day… Feelings of sorrow and tiredness due to the pandemic changed on a daily basis, which was difficult to manage for some women as they did not know in which mood they would wake up in the mornings. This was especially hard for women who worked from home as they expressed that the isolation of only being in the same space, seeing only very few people could affect them differently from day to day. (2^nd^ para is author interpretation only) [Linden 2021]  Some participants noted that receiving prenatal, postpartum, and intrapartum care in their home kept them safe in their quarantine bubbles. However, others participants noted that the pandemic contributed to the isolation of the postpartum period. [DeJoy 2021]  All three mothers reported social isolation from not being able to go out and spend time with friends, families, or other new mothers. (Author interpretation only) [Spatz & Froh 2021]  The changes in postnatal healthcare caused much frustration and dismay. [Sweet 2022]  ‘Going it alone’ – having a baby was an isolating experience All participants described having an isolating maternity experience, encapsulated by one woman, This sense of ‘going it alone’ was evidenced through the sub-themes of ‘attending care alone’, ‘experiencing government restrictions’, and ‘desiring social support’… Attending on their own created the sense of isolation as their partners did not have the shared experience. Participants spoke of how the social restrictions in the home and in health care settings reduced the social support received from family and friends and how this negatively impacted their experience. [Sweet 2021]  Many women described that they valued peer support from other mothers during pregnancy and post-partum, together with intergenerational support from parents. [Atmuri 2021]  Multiparous women reported the difﬁculties in maintaining and managing the relationship with other kids at home, especially in the case of young children. The required prolonged separation ampliﬁed the common worries in case of a sibling’s birth, especially when the sudden hospitalisation was unexpected and not accompanied by any explanation or goodbye from the mothers to the children. Not being able to see other kids increased the women’s sense of loneliness [Fumagilli 2021]  Mothers receive encouragement and appraisal from their family, friends, and coworkers. Two women who worked in health care were able to receive this support in person from co-workers; however, the remaining women reported primarily receiving this over the phone. Most mothers reported being unsatisfied with the amount of appraisal support they received… Another mother reported similar lack of  support once home from the hospital [Snyder & Worlton 2021] | **[Impact on mental health]** Public health restrictions across the globe were similar in that restrictions were placed on movement and lockdowns occurred to minimise transmission and spread. Ultimately the forced isolation significantly impacted women’s mental health, with many describing how their mental health suffered, with an increase in depression and loneliness. Not being able to leave the house or see family and friends meant that they felt increasingly disconnected to their loved ones and unable to access their physical supports, with some describing this experience as “being in a little prison cell”. In addition, the inability to connect with peers and mothers in similar situations impacted their social connectedness, deteriorating their mental health and causing some women to withdraw from society. As a result many women felt that their pregnancy, childbirth and postnatal period was incredibly isolating and lonely. (Jackson 2021a, Ollivier 2021, Rhodes 2021, Panda 2021, Aydin & Aktas 2021, Davis 2021, Green 2021, Saleh 2022, Rice 2021a, Anderson 2021, Jackson 2021b, John 2021, Meaney 2021, Dove-Meadows 2020, Riley 2021, Stirling Cameron 2021, Harrison 2021, Keating 2021, Linden 2021, DeJoy 2021, Sweet 2021, Snyder & Worlton 2021)  **[Feeling abandoned by the public health system**] The lack of support by the government and health care providers in ensuring women were able to navigate public health restrictions with adequate support was evident in women’s descriptions about feeling abandoned. For women with a previous child birthing experience prior to the pandemic, those not able to have support people present during their labour, women who received inadequate care post-birth (e.g. for breastfeeding support and postnatal checks) and could not attend mother’s groups felt strongest about the abandonment by the health care system. The disruptions by the pandemic to provide continuous care and more support for mothers’ post-birth negatively impacted women’s experiences. (Ollivier 2021, Davis 2021, Jackson 2021b, John 2021, Meaney 2021, Harrison 2021, Rhodes 2020, Rice 2021a, Sweet 2022, Atmuri 2021) |
| Managing the new and changing information5.1. Constantly changing advice and information | | |
| DeJoy 2021  Reference 1 - 0.38% Coverage  “It’s just so many unknowns. I think with COVID that really, you know, we’re getting new guidelines every single day on how we’re handling the whole situation and then, there’s just too many unknowns and I just don’t want to risk anything if I don’t have to.”  Kolker 2021  Reference 1 - 0.37% Coverage  “I’ve been trying to avoid looking at the numbers as of late … Looking back I wish I hadn’t known. I think it would’ve been better for me to be kind of ignorant about it, because like it just gave me more anxiety than I was already feeling.”  Reference 2 - 0.08% Coverage  “I’m an information seeker for sure but I found, with COVID, it was just almost too much information.”  Riley 2021  Reference 1 - 0.19% Coverage  “very non-speciﬁc… what you looked at one night was diﬀerent the following day”  Reference 2 - 0.14% Coverage  This was particularly felt “right at the beginning of lockdown, things were changing… on a daily basis, about delivery ”  Reference 3 - 0.24% Coverage  However, online resources were also perceived to be “dangerous… my mum and husband told me to stop googling things in the early days… I wouldn’t recommend it as a resource for an anxious new mum in a pandemic”  Reference 4  Some forums were useful as they had “diﬀerent people’s experiences on there” but also had “its negatives as well, because you get a lot of…judgemental people now” (P7, Age 33, PP 4 weeks).  Silverio 2021  Reference 1 - 0.36% Coverage  “I was conscious, people were adapting as they went along. There was no rule book about what to do here. It wasn’t like right, we’re in this protocol; let’s get it out of the cupboard and dust it oﬀ. People were looking at it and thinking well for this week, on this day, this is the guidance, therefore we’ll do this. It might all be diﬀerent in a week’s time. So, I thought, appointment-wise, everyone handled it pretty well. ”  Reference 2 - 0.22% Coverage  “I guess the fact that the guidelines nationally and also within the hospital felt like they were constantly changing, and possibly adding an extra layer of panic almost, or not fully explaining as to why things have been put in place or haven’t been put in place.”  Sweet 2022  Reference 1 - 0.09% Coverage  “The rules kept changing kind of minute by minute as they got more information, and it was really unsettling”.  Reference 2 - 0.08% Coverage  “The hospital seemed to be in complete chaos, … no one knew what the other hand was doing all the time”  Reference 3 - 0.11% Coverage  “Different people’s adaptations of the rules or the recommendations, I found some to be quite relaxed and some to be quite extreme”.  Reference 4 - 0.31% Coverage  “When I got to the hospital, they didn’t know about the restrictions having been lifted. … That was really frustrating because I was like why? Why does this hospital not know?”.  Reference 1 - 0.22% Coverage  “I got sent all this information after that first telephone appointment and it was a bit overwhelming, information overload … a lot that wasn’t explained to me over the phone maybe because of the limitations of the phone”.  Costa 2021  Reference 1 - 0.22% Coverage  “I actually found (Facebook) really awful. There are some really good and helpful pieces of advice. Everyone is in the same boat trying to help each other out. But it really heightened the anxiety and (made me) compare… It’s all just more worrying.”  Ollivier 2021  Reference 1 - 0.30% Coverage  “I don’t remember trying to look because I ﬁgured it would be pointless & stressful… just didn’t need the added stress. ”  Reference 2 - 0.27% Coverage  “I have become more concerned about the implications for children contacting the virus and the potential long term impacts it may have based on more recent research.”  Sweet 2021  Reference 1 - 0.17% Coverage  “The information was very rapidly changing, and I was obviously keeping up to date on Facebook and that seemed like everyone was sharing diﬀerent updates”  Reference 2 - 0.22% Coverage  “I think my obstetrician –she has like a social media page, she would publish [from] the college of obstetricians, she would post their latest guidance, because she has lots of anxious people”  Mizrak Sahin & Kabakci 2021  Reference 1  “I've been watching it a lot at ﬁrst, frankly, but I've stopped watching the news for a month, I am just looking at the daily number of cases or check if the Minister of Health makes a statement, otherwise I'm not watching the news.” (P8)  Reference 2  “I'm not watching the news, I get stressed out because everyone says something. I listen only to the statements of the Minister of Health, so I like his explanations, you know, it's promising.” (P14)  Charvat 2021  Reference 1  “Confused. Nothing set in stone . . . Yeah, the information I get is all over the place. And so, I mean, the best I can do is keep myself protected and hope to God that it keeps me protected.”  Fumugalli 2021  Reference 1  “I was very scared and started to search for information but obviously the media weren’t helpful due to the overall alarmism. The news circulating were always uncertain and this uncertainty was terrifying.”  Kyno 2021  Reference 1  “It was the nurses who gave us information, but there was often a slight discrepancy between what the different nurses said. We wanted to get information from [a] person with authority, a leader perhaps. It is really serious when the father is not allowed to be present, more than we ask a random nurse and that says: “No, he is not allowed to come in.” (Mother S)  Reference 2  “…. also there was a slight difference in personal attitudes between the nurses, what was okay [to do] and not.”  Harrison 2021  Reference 1  “My biggest fear that caused me a lot of anxiety and depression was whether I would have to give birth alone … I didn’t even know until the day whether my partner was allowed in.”  Meaney 2021  Reference 1  “Restrictions have still not been lifted in ‘Hospital A’ whereas they have been eased in both ‘Hospital B’ and ‘Hospital C’.” (P160; Ireland, 35–39 yrs, multiparous, third trimester) | Particularly at the beginning of the pandemic, some participants were unsure what to believe, trying to sift through and evaluate information from the news media and social media. Some participants expressed confusion about how dangerous the virus was, their risks of catching it at the hospital, or which hospital protocols would apply to their birth. One participant explained the effect of changing knowledge on their risk calculations [DeJoy 2021]  Conversely, most participants reported they avoided  information overload to protect their mental health. As one participant described, information in the media was causing her heightened anxiety and she found she was better off not knowing… Strategies for coping with pandemic stress🡪 Approach to pandemic information 🡪 avoid information [Kolker 2021]  Due to the changing nature of the pandemic, information for pregnant women was… [Riley 2021]  Whilst many women understood why appointments were changing so rapidly, concern often arose about the lack of in-person care, in relation to the growth and wellbeing of the baby during pregnancy or of their newborn infant… Lockdown restrictions and subsequent maternity service reconﬁgurations aﬀected routine appointment schedules and the frequency of those appointments. Antenatal care visits were found to have been either altered frequently or cancelled altogether, with women discussing their perceptions of virtual care appointments as having less value or importance: [Silverio 2021]  All participants recognised the constantly changing landscape in maternity care services. This created a level of uncertainty for them and their families. Some participants praised the staff in managing this constant uncertainty, as P3 said, “the midwives and health professionals … did the best that they could with the information that they had, I think they did a brilliant job, I think it was a really uncertain time”. Whilst others described disarray… The ways in which the constant changes impacted on care varied. P11 explained, “different people’s adaptations of the rules or the recommendations, I found some to be quite relaxed and some to be quite extreme”. One participant found minimal change in her health care experience…. Participants spoke of following the regular daily updates on physical distancing rules from the government. This caused much frustration when the changes were not implemented in healthcare straight away... Telehealth was described as impersonal and incomplete. [Sweet 2022]  However, not all accounts of utilising social media in this way were positive [Costa 2021]  Another participant also believed the amount of information was overwhelming and diﬃcult to navigate; however, she chose not to engage in information-seeking as it was too stressful…. Agency in knowing what they needed (or didn’t) in relation to health information was apparent, though enacted in diﬀerent ways by diﬀerent parents. Some mothers speciﬁcally spoke about their fears of COVID-19. They  generally sought information from sources they deemed reliable, such as the provincial public health oﬃce. That being said, others still felt confused or misinformed about how to care for their baby during the pandemic [Ollivier 2021]  Many participants accessed information on social media, most commonly Facebook… [Sweet 2021]  Some of the pregnant women said they refused to watch the news in order to avoid anxietndemic process. [Mizrak Sahin & Kabakci 2021]  The experience of being pregnant during the COVID-19 pandemic was characterised by being in an information echo where information about the virus seemed to be everywhere and nowhere at the same time. [Linden 2021]  Many participants were stressed with the unclear and constantly changing information, which indicated ineffective informational support. When asked how she feels about the information she has received from her doctors, Becka said… hospital resources to dedicate to these pregnant women. Regardless, inaccurate or incomplete knowledge about the coronavirus hindered participants’ ability to make sense of their experience and to make informed decisions about their and their baby’s health [Charvat 2021]  The confusion was ampliﬁed by the over-information, misinformation and disinformation provided by mass-media, mainly coming from TV news, newspapers and the internet. Some women described their need of searching and selecting reliable information on COVID-19 speciﬁcally related to childbirth, whilst others stopped following news as this was causing increased anxiety (W21) [Fumagilli 2021]  The nurses provided information to parents, and a common frustration related to the regulation evolved. Parents recounted how nurses tried to help them to speak up for their needs. Despite the nurse’s efforts, a lack of organization and leadership was questioned. Parents also questioned how and on what basis health personnel could impose restrictions on parents… The parents reported inconsistent enforcement of regulations resulting in frustration related to both the enforcement of the COVID-19 regulations and the overall lack of information. Mother R told… The parental confidence in the health personnel weathered by the wear and tear of living with the measures over time [Kyno 2021]  Frequently changing policies regarding face-to-face healthcare checks were a source of confusion and alienation for interviewees. Respondents also frequently experienced anxieties about attending hospital and GP appointments, which appeared contradictory to national advice for new mothers to ‘shield’ (author interpretation only) [Jackson 2021a]  Policies were ever-changing such that there were times when change would occur during a patient’s labor and delivery hospitalization. Despite the connectedness of society, providers and hospitals struggled with finding a reliable way to relay these changes effectively to patients. (author interpretation only) [Saleh 2022]  However, many women described how their pregnancies had been overshadowed by anxiety about whether their partner would be allowed to be present and whether they would be able to give birth at the place and in the manner they had chosen. Their stress was increased by uncertainty: policies changed over time and were inconsistent between different providers. [Harrison 2021]  Women were also aware of how restrictions on access and care differed between hospitals within the same geographic area, which added to their frustrations. [Meaney 2021]  When asked about where they received their information, many relied on HCPs, websites, and social media. However, many noted they would have preferred a central repository of up-to-date information, so they were not forced to vet the bombardment of information, and make major decisions that impacted their health alone. Due to the novelty of the virus, there often was a lack of information, conflicting information, and unfortunately misinformation. As new information about COVID-19 came out, healthcare providers struggled to keep up with the latest data as well as find best practices to pass along the information to patients. This often left patients feeling uninformed and confused about the information they did and sometimes did not receive. (Author interpretation only) [Saleh 2022] | **[Adapting to the constantly changing rules and public health restrictions]** At the beginning of the pandemic, there was the constant development of public health guidelines and changes to hospital policies. Women described their experiences of the different public health restrictions that seemed to change daily, or by the minute creating uncertainty. These feelings resonated throughout healthcare settings, as many hospitals were changing their protocols constantly, meaning women had little control over their birthing plans, causing increased stress and panic levels, as it was not made clear why such changes were to be made, e.g. banning of waterbirths and nitrous oxide. Many described that the information they received was very non-specific and difficult to plan for. Women also mentioned how different health services seemed to have different rules and implementation and adherence levels which ranged from being relaxed, to extreme, as well as some being inconsistent or slow to adopt government updates to public health restrictions, causing increased frustration. However, some women acknowledged that this was a challenging experience for all, and information, policy and guidelines were adapted adequately in response to the changing environments. (DeJoy 2021, Kolker 2021, Riley 2021, Silverio 2021, Sweet 2022, Sweet 2021, Charvat 2021, Kyno 2021, Harrison 2021, Meaney 2021)  **[Overload of information]** Although women sought relevant information for themselves, many struggled with the onslaught of information, updates from the government, hospitals, and media outlets. Many described following COVID updates, case numbers, and the constant changing restrictions to be unsettling, increasing anxiety and stress levels, meaning women ultimately avoided these updates where possible. Navigating reputable sources of information was also an overwhelming task, this added another layer of stress that women did not want, thus some decided it was best not to search for it. An overload of information was also described in the setting in which information was provided over the phone which was not an effective means of communicating large amounts of information. This was generally overwhelming and difficult to comprehend. (Kolker 2021, Riley 2021, Sweet 2022, Costa 2021, Ollivier 2021, Mizrak Sahin & Kabakci 2021, Fumagalli 2021, Saleh 2021) |
| 5.2. Inadequate information from healthcare providers | | |
| Riley 2021  Reference 1 - 0.10% Coverage  “That lack of information, on top of this situation, will probably make you quite anxious”  Reference 2 - 0.27% Coverage  Participants talked about how they “weren’t given any information on which [health] service(s) [they] should contact and for what period of time they would support”  Silverio 2021  Reference 1 - 0.34% Coverage  “I think there was a lot of confusion; the one negative was there was no good communication about what was happening to appointments. You weren’t really sure; were they happening on the phone, when were you going to get the call, were you going to get a letter now stating that it was now going to be a phone-call? Which no, there was very little communication. So, I always felt a bit uneasy about that…”  Reference 1  “…at 34 weeks I had a telephone appointment and I tried to ask what changes are there in the hospital, because of COVID and talk about the birth plan. She basically said, ‘Everything is changing so quickly there is no point in us even talking about that now. Wait until your next appointment.’ My next appointment wasn’t until 38 weeks and I remember thinking that was really strange because I could technically have had the baby by 38 weeks. It felt like I was being brushed oﬀ a bit and that made me quite anxious because it felt like I hadn’t had the chance to have that conversation. At 38 weeks I had a face to face appointment and that was supposed to be where we talk about the birth plan, but when she checked for the baby’s heart rate, she said it was a bit faster than it should be. She sent me straight to the assessment unit again, so we never had the chance to talk about the birth plan.” (Participant-022)  Sweet 2022  Reference 1 - 0.15% Coverage  “I wasn’t told about mother’s groups, I did find about them myself, but I know that quite a few of the others in my mother’s group had to search for themselves as well, they weren’t told about it”.  Reference 2 - 0.34% Coverage  “because everything was so busy and the midwives and doctors were so unsure of what was going on and they hadn’t been given a lot of information I could sense that everyone was a little bit stressed and a bit like – ‘well we don’t know what’s going on’ they weren’t outwardly trying to show that, you could sense that everyone was a little bit stressed, so it wasn’t a nice feeling – it kind of made me feel a little bit like – not safe”  Reference 3 - 0.09% Coverage  “There was utter confusion and chaos generally at the hospital, like I could never count on anything being the truth”.  Reference 4 - 0.19% Coverage  P16 did not have continuity of care and said, “there’s disconnect in information, I’ve found the disconnect between the information that my GP [general practitioner] was getting and that the [hospital] was getting they weren’t getting the same”.  Reference 5 - 0.25% Coverage  “Towards the end it was basically just like ‘[the] birth plan’s out the window let’s just go with the flow’, because I had an obstetrician saying you absolutely have to have a c section and when I got to the hospital and they were like “are you sure you want this c-section?”.  Reference 6  “Different people’s adaptations of the rules or the recommendations, I found some to be quite relaxed and some to be quite extreme”  Reference 7  “there’s disconnect in information, I’ve found the disconnect between the information that my GP [general practitioner] was getting and that the [hospital] was getting they weren’t getting the same”.  Meaney 2021  Reference 1 - 0.21% Coverage  “COVID- risk of infection and how little is known about how it affects pregnancy/new studies showing that it impacts the placenta. Worried about visitors after baby is here and if she gets it her suffering long term effects that we still don’t know that much about.”  Reference 2 - 0.10% Coverage  “Restrictions have still not been lifted in ‘Hospital A’ whereas they have been eased in both ‘Hospital B’ and ‘Hospital C’.”  Reference3 - 0.10% Coverage  “Conﬂicting advice and recommendations about covid 19 in pregnancy. Would have preferred more cautious guidance given lack of evidence.”  Reference 4  “The unknown about what it will look like in January when I give birth. Hoping my husband and mom can be there. Also worried if I were to have the virus they would keep the baby from me. Just a lot of unknowns, especially with pregnant women being in the high risk category now.  Rhodes 2020  Reference 1 - 0.11% Coverage  “I have had very little contact with my midwife and I really like how much information I can get from the app given that I can’t seem to get any support from anywhere else.”  Reference 2 - 0.09% Coverage  “I have enjoyed using Baby Buddy throughout pregnancy and these early months and find it even more reassuring now that external input is so limited.”  Reference 3 - 0.09% Coverage  “I’m not the best at researching stuff and reading a big book in advance so this gives me nice little bite-size chunks of things I need to know.”  Reference 4  “I don’t think I am being supported. The Government should be giving us more information, like where do you go to get your baby weighed…there were 8 weeks when she wasn’t weighed – quite a worry really.” [Baby 12 weeks, age 30-34 years, White British]  Reference 5  “I just feel like new moms and parents have just been completely left out of the Government’s mindset and their exit strategy really.” [Baby 3 months, age 30-34 years, White British]  Anderson 2021  Reference 1 - 1.30% Coverage  “I just find them a bit vague when it comes specifically to pregnancy, I don’t think the messaging is really clear. So it suggests that we’re vulnerable but then we’re not in the really vulnerable group, and it’s a bit grey, so I am not quite sure once I tell me employers I am pregnant how I will be treated, I don’t know if they will say I need to stay at home and shield.”  Reference 2 - 1.62% Coverage  “I don’t know, even though I am pregnant and I am classed as high risk category I don’t feel like I am a risk, and I don’t feel scared about it. I don’t know, maybe I should be more, but I think if it was really explained a bit more that no actually you have… there’s a very high risk you could have a miscarriage if you got it then I would be really… I don’t really understand why. So I think if I understood it a bit more I might maybe take it a bit more seriously.”  Davis 2021  Reference 1 - 0.27% Coverage  “It would have been useful to maybe have some generic information that went out to women in that situation, if you were pregnant, [for example], “It’s early days. We don’t know what the impact could be.” . . . Statements from a medical professional to put people’s minds at ease.”  Reference 2 - 0.26% Coverage  “When they cancelled everything, it took weeks for them to do anything online and then it was pages of reading, there’s no videos, no nothing, and then you have all these questions, and you try to call to ﬁnd out some answers and no one can give you any answers and there was no one to talk to.”  Reference 3 - 0.41% Coverage  “You shouldn’t have to be going private to get this information because it’s really just standard information that anyone should be getting . . . you shouldn’t have to pay that amount of money to get what’s really a basic human right to understand and to get.”  Reference 1 - 0.57% Coverage  “It was the nurses who gave us information, but there was often a  slight discrepancy between what the different nurses said. We wanted to get information from [a] person with authority, a leader perhaps. It is really serious when the father is not allowed to be present, more than we ask a random nurse and that says: “No, he is not allowed to come in.”  Reference 4 - 0.15% Coverage  [The information] . . . it’s just too complicated for a normal person to understand it. We’re not medics, we’re not in that ﬁeld, so you’re just like, “What exactly are you trying to say?”  Charvat 2021  Reference 1 - 0.25% Coverage  “For the virus, honestly, I bring it up . . . and they’re very, er, not [open] about it. They just tell me that, “Oh, you’re going to have to wear a mask. We’re going to have to test you.” But they don’t really tell me [anything]. And [my obstetrician] makes me feel comfortable as he tells me not to worry. He says I’m not high risk, etc. But no . . . my OB doesn’t talk much about the actual virus.”  Jackson 2021a  Reference 1  “I think one thing that hasn’t been made clear to me, but I’d kind of found out through other sources is that in terms of my six-week check…apparently they’re doing it all [in one appointment] when [youngest] has his eight-week jab, and that hasn’t been made clear”  Reference 2  “What I deem is essential as an expectant mother and what [healthcare professionals] actually think is essential might be completely different. So why don’t [they] tell me what [their] definition of essential is?”  Reference 3 - 0.44% Coverage  “You’re told you’re vulnerable and you have to isolate but then you still have to go to hospitals or, you know, health facilities for your appointments, which don’t feel as safe...it just sends a bit of a mixed message you know? Oh gosh, I’m having to go to the lion’s den to have this appointment.”  Keating 2021  Reference 1 - 0.14% Coverage  “the phrasing used was.. your birth partner will be allowed in when you’re in active labor… like what does that mean?  Reference 2 - 0.04% Coverage  “I’m getting different messages”  Reference 3  “Initially they were saying ‘no pregnant women aren’t any more high risk than anybody else’ but then.. the HSE are saying to their own pregnant staff ‘you shouldn’t be working near coronavirus patients’”  Kyno 2021  Reference 1  “They should have tried to coordinate [the information]. . .. There was a lot of divergent information. I wish they had written something down. . . I asked is there a place I can write, or a place I can read”  Reference 2 - 0.24% Coverage  “. . .. also there was a slight difference in personal attitudes between the nurses, what was okay [to do] and not.”  Reference 3 - 0.41% Coverage  “That is, they place a stronger burden on parents who are already under great pressure than they themselves are able to follow, and this is where it begins to break, that it is not logical, and it is not something they live by themselves—and there I think the management must come in and have a very clear tightening of the staff, where they clearly say what applies—personal opinions are not welcome.”  Linden 2021  Reference 1 - 0.40% Coverage  “I think there should have been a little more from the midwives when you were pregnant… a little more information… that you did not constantly needed to, you know, chase them and ask - How is the situation now?”  Mizrak Sahin & Kabakci 2021  Reference 1 - 0.55% Coverage  “I learned it by myself; I don't think it's enough as we need more  information.”  Reference 2 - 0.15% Coverage  “. . . no, I learned this information by myself as I couldn't go to the hospital due to this disease, and I didn't get any information.”  Ollivier 2021  Reference 1  “I had to research and learn myself how to ﬁx my issues - again it has been diﬃcult ﬁnding support but proud that I got breastfeeding ﬁgured out now! All myself too.”  Reference 2 - 0.30% Coverage  “I don’t remember trying to look because I ﬁgured it would be pointless & stressful… just didn’t need the added stress.”  Reference 3 - 0.27% Coverage  “I have become more concerned about the implications for children contracting the virus and the potential long term impacts it may have based on more recent research.”  Reference 4  “We had one meeting with public health before they reassigned our nurse to the COVID team and we were dumped. The breastfeeding clinic cut her tongue tie but did a phone follow up instead of in person due to COVID and dumped us even though we were still having feeding issues.” (Participant #8)  Sweet 2021  Reference 1 - 0.33% Coverage  “Because there wasn’t a lot of information around whether COVID was more dangerous for people that were pregnant or not. There was contradicting statements saying yes it can get to your baby through the womb and no it can’t, and so there was not much information that was 100% certain”  Reference 2  “I think it was just that anxiety. … [so I was] trying to ﬁnd out as much information as I could. …I think there was a lot of anxiety on my part …I’d ask questions like ‘is this going to aﬀect my pregnancy?’ and they’d say, ‘we just don’t know’”  Reference 3  “It was all negative kind of how you had to deal with everything and the stresses of all of that. I think seeing a maternal nurse in person and having a chat is very important”  Reference 4  “I feel like I’ve needed to ask for more information, around… the early signs of labour and all that sort of stuﬀ, just because I am a ﬁrst-time mum and I don’t know what to expect ” (P26).  Stirling Cameron 2021  Reference 1  “Sometimes they explained things to me by using signs and I understand a little English but it’s hard to understand medical terms and they didn’t use an interpreter for this.” (Participant 6)  Atmuri 2021  Reference 1  “What happens when you come in? Because obviously there’s not been hospital tours . . . I wouldn’t even know where to go. A video online on the website or something that you can go on and get a tour may be helpful, starting from outside so you know where you’re going.”  Reference 2  “You do feel a little bit stressed in there, and I think probably one thing that maybe could be improved is just that extra information of what you are doing with the COVID stuff in terms of precautions, what it’s going to look like when I come in to have bubs, just what to expect.” TB  Kolker 2021  Reference 1  “it would be great if they had a better idea of what your stay is going to look like, so you’re a bit more mentally prepared for what’s to come.” [P10]  Reference 2  One participant put it plainly, “there was a lot of confusion … try to be as clear to the patients as you can.” [P5]  John 2021  Reference 1  “I don’t know if that’s the pandemic causing people to be a bit more relaxed and, you know, like okay, here’s the leaflet, you can go and do the reading.”  Harrison 2021  Reference 1  “When we went into lockdown I couldn't get hold of the midwife, all my appointments and training was cancelled, the hospital didn't have any information, and everybody was panicking. It took about 4 weeks for information to get to me. I didn't see my midwife from week 24 and had different ones every time because of COVID.”  Fumugalli 2021  Reference 1  “Can’t fault them, information was good and they sent all the links to join the online antenatal classes.” (W15)  Costa 2021  Reference 1  “(The nurse) knows about the anxiety I’m having, but I’ve still had to ask about everything […]. The communication has not been very good […]. They haven’t bothered to get in touch at all […] and see if I’m doing OK.”  Reference 2  “(The CL/P team has) always been good at communicating when I’ve asked a question, but not necessarily coming forward and telling me things.” | Due to the changing nature of the pandemic, information for pregnant women was “very non-speciﬁc… what you looked at one night was diﬀerent the following day” … This uncertainty and lack of information was still an issue postpartum… Lack of contact following the birth of their child. This was diﬃcult for participants… [Riley 2021]  Lockdown restrictions and subsequent maternity service reconﬁgurations aﬀected routine appointment schedules and the frequency of those appointments. Antenatal care visits were found to have been either altered frequently or cancelled altogether, with women discussing their perceptions of virtual care appointments as having less value or importance… In addition, women discussed how reduced frequency of antenatal care appointments made them feel, with provision of virtual care not equated to the in-person care they had either expected or wanted: [Silverio 2021]  The changes in postnatal healthcare caused much frustration and dismay… The changing landscape of the pandemic and healthcare provision left women feeling ill-informed and uncertain. They experienced delays in health service policy information being transferred to their care providers, and then on to them. This uncertainty and poor communication led to a sense of anxiety… The ways in which the constant changes impacted on care varied… P16 did not have continuity of care and said [Sweet 2022]  Women’s responses indicated how the dearth of  evidence and lack of information related to outcomes exacerbated these fears. Many of the women conveyed issues of control whereby the lack of information related to COVID-19 during the antenatal period worried them leading to uncertainties around pregnancy and birth, irrespective of parity. Women’s preparedness was adversely impacted leaving women with a sense of powerlessness… Women also indicated how important good communication was during the antenatal period, especially to allay any fears, concerns or anxiety they may be experiencing during pregnancy. When asked to comment on their concerns linked to the pandemic, many of the women reported fears of contracting COVID-19 and what implications that would have for their health and the health of their infants (see Table 3). Women were aware that they were deemed to be a vulnerable population who may be at risk if they were to be infected, but that little was known or communicated to them about these risks. Women’s responses indicated how the dearth of evidence and lack of information related to outcomes exacerbated these fears. Many of the women conveyed issues of control whereby the lack of information related to COVID-19 during the antenatal period worried them leading to uncertainties around pregnancy and birth, irrespective of parity. Women’s preparedness was adversely impacted leaving women with a sense of powerlessness. [Meaney 2021]  Comments from the free text boxes and telephone interviewees showed that many women were finding Baby Buddy to be particularly valuable in the absence of support from health care professionals and baby groups… Similarly, interview respondents consistently praised the Today’s Information feature for being interesting, useful, and time appropriate. Indeed, several respondents remarked on the seemingly uncanny timing of the messages. [Rhodes 2020]  Women reported making efforts to access “credible” and “reputable” sources for information including government advice, BBC (British Broadcasting Corporation) news, Royal College of Obstetricians and Gynaecologists (RCOG)/ midwives, NHS, pregnancy apps/emails (e.g. Bounty), Tommy’s (online), select social media groups, contacts, scientific sources, and newspapers. While mainly reporting good understanding of, and confidence in adhering to the guidelines, women reported a lack of clarity about why pregnant women were “at risk” and what it meant. Some felt it was precautionary, many wanted more explanation. Uncertainty around the risk category was evident in women’s different interpretations of what was expected of them: some interpreted it as requiring them to shield due to the ‘at risk’ status of being pregnant, while others stated that the guidance was no different for pregnant women from the rest of the population. Many felt the advice was confusing initially, though seemed clearer by the time of interview. Some details of the behavioural recommendations remained unclear, such as how to handle shared parenting between households, how to stay safe at work and whether to attend healthcare appointments for minor issues… Maternity care changes were a major concern for most of our participants. While recognising that midwives were doing their best in a difficult situation, many women had experienced not only a loss of care, but a lack of communication about changes to their healthcare, with reports that midwives have been ‘hard to get hold of’. The loss was particularly acute for women in their first pregnancy, who did not know what they were missing out on. (Last para is author interpretation only) [Anderson 2021]  Additionally, some information would have been preferable to no information … Women had varied experiences with the newly imposed hospital restrictions; generally, the high group were satisﬁed with the information and support from the hospital: “I loved my experience there. I received heaps of support and it was really great.” (CW9_high) Several women in the ‘low’ group struggled with the immediate impacts… One participant was especially aggravated by the lack of antenatal care information from the hospital so moved to a private hospital where she could access online information videos that met her care needs. However, she recognised the inequity in this situation… Information needs to be communicated that is easy to understand: [Davis 2021]  Second, many participants’ stories included a lack of informational support from their healthcare providers regarding how the COVID-19 virus would affect both their pregnancy and the birth. Grace discussed that her obstetrician did not give her concrete information about how the virus could affect her or her developing fetus… The lack of informational support is likely due to a general lack of understanding about the virus in the medical community. At the time of these interviews, many hospitals were not conducting elective procedures, and medical staff may have been overwhelmed with the changes to their job and the challenge of treating coronavirus patients. Therefore, medical personnel may not have the time, energy, or hospital resources to dedicate to these pregnant women. Regardless, inaccurate or incomplete knowledge about the coronavirus hindered participants’ ability to make sense of their experience and to make informed decisions about their and their baby’s health (last para is author interpretation only) [Charvat 2021]  The majority of respondents mentioned lack of clarity and associated feelings of distress and frustration regarding the six- and eight-week postnatal check-ups. Lack of consistency and clarity from healthcare professionals was a source of anxiety and confusion… For these women, advice was only given if actively sought, otherwise, many were left confused and questioning whether their concerns warranted medical attention… Contradictory advice concerning national guidelines for mothers to adhere with social distancing guidelines, whilst also being invited to attend hospital appointments, where the risk of contracting COVID-19 was perceived to be higher, was a source of confusion and distress [Jackson 2021a]  Balancing information 🡪 lack of up-to-date pregnancy information: The lack of up to-date pregnancy-specific information about the virus and its potential impact on pregnancy was a source of anxiety. Women also feared for the health of their babies and how they would manage to care for other children if they were to become unwell. They looked at hospital websites, but this was often not their primary source of information. The lack of relevant information meant that women were less likely to trust their healthcare providers as a source as they realized that the medical community was uncertain about the effects of COVID-19 in pregnancy. Conflicting information was a source of anxiety for women. Some of the messaging and language used around restrictions by the hospitals were unclear which caused stress amongst women, especially when information related to partners’ presence on delivery suite. [Keating 2021]  As times goes by, the parents described an experience of dissatisfaction regarding the apparent novelty of the COVID-19 pandemic situation to both staff and leadership… The parents reported inconsistent enforcement of regulations resulting in frustration related to both the enforcement of the COVID-19 regulations and the overall lack of information… The parental confidence in the health personnel weathered by the wear and tear of living with the measures over time… The nurses provided information to parents, and common frustration related to the regulation evolved. Parents recounted how nurses tried to help them to speak up for their needs. Despite the nurse’s efforts, a lack of organization and leadership was questioned. Parents also questioned how and on what basis health personnel could impose restrictions on parents. [Kyno 2021]  A common experience described by all the women was a general lack of information from health-care providers. Women expressed that even if knowledge about COVID-19 at the time was limited, that existing information was not communicated adequately from health care providers to them. This lack of dialogue and the fact that women had to actively ask their midwife about the latest development and guidelines was experienced as stressful for some women resulting in feelings of uncertainty… The absence of pregnancy related information led to some women searching news outlets and internet sites for the latest developments. [Linden 2021]  The most important sources of information of pregnant women during this process were internet and television. Speciﬁcally, pregnant women who could not attend controls for pregnancy monitoring were receiving information via internet and television when they could not reach their doctor, midwife and nurse. Pregnant women who had access to information through their own learning thought that their knowledge was not enough. [Mizrak Sahin & Kabakci 2021]  The complexities of navigating postpartum health information on one’s own was overwhelming for many mothers. Many participants utilized agency to solve their issues and seek out the information they needed… . Another participant also believed the amount of information was overwhelming and diﬃcult to navigate; however, she chose not to engage in information-seeking as it was too stressful… Agency in knowing what they needed (or didn’t) in relation to health information was apparent, though enacted in diﬀerent ways by diﬀerent parents. Some mothers speciﬁcally spoke about their fears of COVID-19. They generally sought information from sources they deemed reliable, such as the provincial public health oﬃce. That being said, others still felt confused or misinformed about how to care for their baby during the pandemic… Public health orders during COVID-19 made accessing information and support very diﬃcult for mothers in our study. Many participants said they were concerned that they did not have the right information about how to best care for their baby, including how to safely breastfeed. With unclear information, mixed messaging, and unknown reliability of certain information sources, parents had to make diﬃcult decisions regarding what sources and who to trust when it came to caring for their new baby. [Ollivier 2021]  Despite the available information, participants spoke of the uncertainty of the impact COVID-19 may have on them and their developing baby. This uncertainty left some women seeking more reliable information… Seeking information and reassurance were ways women described to manage their situations... Having the ability to have face-to-face healthcare was helpful to overcome stress… Participants described varied experiences about their decision making throughout their maternity care. Some felt supported and given choice, others felt their options were very limited, P21 found conflicting advice concerning… Whilst most participants spoke of seeking information about the pandemic and its possible impact, some participants spoke of more general information needs about pregnancy and birth, as they felt these were not adequately covered in their telehealth appointments. [Sweet 2021]  Women who gave birth during early COVID-19 experience observed growing fear and concerns by HCPs, lack of or confusion over personal protective equipment (PPE) use, social distancing, and community and school closings. Furthermore, women in the early groupings had to struggle with lack of vaccine availability, ongoing news and research, and concern and distress noted in both laypersons and healthcare communities. Key health informants (the Centers for Disease Control and Prevention, the National Institutes of Health, and local providers) were publicly disbelieved causing confusion and anxiety for healthcare consumers. Late COVID-19 experience was identified by vaccine distribution and confusion related to its impact on pregnancy, growing research data availability, statements coming from key health information sites readily available, and an increase in organized misinformation sites causing confusion to laypersons… Women experienced COVID-19 confusion no matter where they fell in the COVID-19 timeline. Education and dissemination of information that would decrease anxiety and prepare women for birth was deemed not as critical as implementation of policies to reduce transmission. Therefore, confusion over appointment changes or hospital policies was described by many women…. For any number of reasons, healthcare policies were enacted unevenly and haphazardly and not necessarily based on public health science. The notion of who should mask and when, who should test and why, and what was the plan/policy for the mother/infant dyad caused great concern for the mothers in this study. These policies were impacted by where in the pandemic cycle they occurred as well as where they gave birth geographically. (All author interpretation only) [Saleh 2022]  Several participants indicated that they were not given any interpretation at all and were forced to navigate  healthcare interactions in English during COVID-19… This participant felt particularly frustrated by this experience and did not feel like she fully understood the health information shared by her care providers. Moreover, information shared online about changing public health restrictions and hospital-based restrictions was predominantly provided in English. This language barrier made it difficult for women to keep up with the constantly evolving guidelines and restrictions related to COVID-19 and their understanding of access to postnatal care. [Stirling Cameron 2021]  Mothers reported that there were ever-changing recommendations which made it difﬁcult to navigate the health care system. The mothers experienced conﬂicting advice about whether or not partners were able to be present at follow-up paediatric visits. One mother was speciﬁcally told that she could not bring her partner to the first newborn visit and this caused great stress to the mother as the weather was terrible that day and she had to navigate the car seat, doors, all while holding an umbrella. When she got to the waiting area, she saw that other mothers had partners present. She thought about texting her husband but decided against it because she felt she had to follow the rules. (Author interpretation only) [Spatz & Froh 2021]  With the cancellation of face-to-face education and hospital tours, women sought guidance on alternate sources of information to prepare for birth. They also requested clariﬁcation on the impact on delivery of postnatal services in the community… Many women desired ofﬁcial communication from the hospital  about the various uncertainties of the pandemic, such as the risk of acquiring the infection and what precautions were being taken to reduce this risk. [Atmuri 2021]  With the ever-changing recommendations and suggested precautions during a pandemic, participants felt there should be clear communication and up to date information coming from their HCPs. Specifically, they suggested clear messaging regarding hospital policies and what care would look like during their hospital stay…. Moreover, participants wanted the health care team to have consistent messaging regarding in person versus virtual visit timing. [Kolker 2021]  Women expressed uncertainty surrounding the accuracy of information relayed via telephone… Although all participants reported some level of information provision from healthcare providers regarding clinical decision making, almost everyone agreed that they would benefit from more thorough discussions. Most participants received information about their pregnancy in the form of signposting to books or websites but they expressed that their individual information needs would have been better met by one- to- one discussions Women who felt that their questions remained unanswered did not feel involved in shared decision making. (Last para is author interpretation only) [John 2021]  Other aspects of the organisation of pregnancy care that had caused women stress were the cancellation of appointments at short (or no) notice, appointments being moved out of the community to hospitals which were more difficult to reach, appointments lasting only a few minutes with no opportunity to ask questions, the loss of continuity of care, and the lack of clear information about changes. [Harrison 2021]  In the context of abrupt and continuous alterations to midwifery care, most women felt they had received clear and timely information from healthcare professionals, helping them adapt to and cope with chaotic changes… However, some participants reported less positive experiences, particularly in regard to communication… Some participants felt they had to make a considered effort to stay in touch themselves. [Fumagilli 2021]  However, some participants reported less positive experiences, particularly in regard to communication… Some participants felt they had to make a considered effort to stay in touch themselves [Costa 2021] | **[Lack of information caused uncertainty and worry]** Many women described the inadequate information on the impact of COVID on their pregnancy and unborn baby, which meant that women did not know if they were categorised as ‘high-risk’ or should be concerned about infection. These unknowns placed a greater concern about both short-term and long-term impacts of the virus. Clarity on hospital policies for labour and birth was also lacking. Additionally, minimal information about how and where to access care during their pregnancy and post birth caused increased levels of uncertainty and worry. This was also highlighted in the healthcare setting, as some described the “utter confusion and chaos”, as healthcare providers could not provide adequate information and reassurance for women. For some women, they decided that to obtain the information they required and needed, they transitioned their care to the private sector, acknowledging the privilege that they had but also the unfairness of needing to do so to stay informed. (Riley 2021, Silverio 2021, Sweet 2022, Meaney 2021, Rhodes 2020, Anderson 2021, Davis 2021, Jackson 2021a, Mizrak Sahin & Kabakci 2021, Ollivier 2021, Sweet 2021, Atmuri 2021, Kolker 2021, Charvat 2021, Saleh 2022, John 2021, Linden 2021)  **[Poor communication with women]** Women described the lack of contact and minimal information that they received from their healthcare providers. The lack of communication from hospitals and healthcare providers in when appointments would be scheduled, how they would be attended given the public health restrictions caused anxiety for families. In response to the poor communication, many women obtained their information from other online resources, peers that were going through the health system and family members, placing a greater distrust in health services. In addition, some women described the complex nature of information that was released, stating that it was difficult to understand and needed the information to be delivered in lay terms for the general public to understand. (Riley 2021, Silverio 2021, Sweet 2022, Meaney 2021, Rhodes 2020, Charvat 2021, Jackson 2021a, Kyno 2021, Linden 2021, Ollivier 2021, Stirling Cameron 2021, Kolker 2021, John 2021, Harrison 2021, Costa 2021, Fumagalli 2021, Anderson 2021, Saleh 2022)  **[Navigating contradictory information]** At the beginning of the pandemic the limited information about the adverse effects of infection on pregnancy created concern for women. Their worries were amplified when there was contradictory information about the impact, with women recounting different statements about vertical transmission, and how it would affect their pregnancy. In the healthcare setting, women also described being concerned by the discrepancies in information provided by the hospital and at the community level, by General Practitioners. A subset of women with newborns in special care and neonatal intensive care units also had to navigate the discrepancies in information provided to mothers about visitation and whether or not partners were allowed into the units. Women who were navigating hospital policies leading up to their delivery date experienced mixed messages by different healthcare providers, suggesting that they had to be prepared, yet flexible for adaptations to their birth plan. (Sweet 2022, Meaney 2021, Davis 2021, Jackson 2021a, Keating 2021, Kyno 2021, Sweet 2021, Spatz & Froh 2021,  **[Strategies for navigating information]** Some women provided advice on how to better communicate the uncertainty. Women suggested that their healthcare providers could have been more proactive in disseminating information with women, making it clear without the use of medical jargon. They would also have preferred if the uncertainty was acknowledged by a medical professional and communicated so that the information could be accessed, and easily understandable to put their minds at ease. They also requested that healthcare providers be clear internally what the current guidance was before disseminating this to mothers. The inconsistent messaging created confusion, and great uncertainty and distrust towards healthcare providers. Women also used trusted apps to obtain their information. (Meaney 2021, Rhodes 2020, Davis 2021, Keating 2021, Kyno 2021, Ollivier 2021, Sweet 2021, Kolker 2021, Fumagalli 2021) |
| Being resilient and optimistic6.1. Self-help strategies to overcome the challenges of the pandemic | | |
| Kolker 2021  Reference 1 - 0.31% Coverage  Participants stressed the importance of advocating for oneself in order to ensure adequate care, “I think that everyone’s doing a really good job with doing the best care they can, but I think it’s important to ask questions and seek out information about pregnancy or postpartum things, if you need it. I think that’s normal anyway, but I think, with the pandemic, it just makes it so much more necessary.”  Reference 2 - 0.33% Coverage  Participants focused on the importance of reducing isolation, “keep socializing as much as possible, bundle up and be outdoors as much as possible. Keep talking to people – friends, family, whoever. I mean those are strategies that worked for me, but I mean maternity leave can be a very isolating – I found it isolating without a pandemic at the same time. So even more so this time, I feel like we just have to rely on each other.”  Reference 3 - 0.28% Coverage  Despite pandemic restrictions, participants emphasized the importance of reaching out for help, whether from family, friends, or health professionals: “having people that you can talk to, whether it’s a partner or family member, friend, a mental health professional … you don’t want to be holding those emotions inside of you, you want to find a release.”  Reference 4 - 0.29% Coverage  “You have to be your own best advocate because nobody is really advocating for you which sounds really depressing – because I don’t even mean it in a depressing way, I just don’t think anybody has time. You know, and I had, like – yeah, and just sort of be mindful of the fact that the system is overstretched so you have to really advocate for yourself and don’t be afraid to be annoying.”  Reference 5 - 0.29% Coverage  “I think you need help at this time. So if you don’t have that, figuring out of you can have a friend or family member kind of come stay with you and be part of your bubble or … like if you feel you need help, find a way to get it right now. […] But I think if people are in that situation, you can’t just never see anyone and do this by yourself all the time. You need help somehow”  Reference 6 - 0.13% Coverage  “Every day I try to list what I’m grateful for. I get a lot  of joy just out of the baby, like why have a baby if you’re not going to find her endlessly fascinating.”  Reference 7 - 0.25% Coverage  “We decided it was more important to have people around, like COVID was a lower risk I guess than my mental health at that point.”  Reference 8 - 0.28% Coverage  “We decided that the risk for my mental health was greater than the risk of them catching COVID from me, so I ended up going to stay with them [family] … If I didn’t have the ability to go and stay with them, I think that that would have been really detrimental.”  Reference 9 - 0.05% Coverage  “I’m on the phone all the time, I guess that’s my coping mechanism.”  Reference 10  “At around two months I had to leave the city and go stay with my parents … it was definitely a feeling of unfairness, this isn’t fair. I wanted to have family, I wanted help.” [P9]  Anderson 2021  Reference 1 - 1.65% Coverage  “Routine wise I try and get outside everyday if I can, especially as we’ve got a park nearby, for a walk or a run, but I am doing less running these days, and just I find that helps to keep me sane really getting outdoors and exercising. I think planning ahead for shopping really helps, so I have been doing a lot more making shopping lists before I go out rather than just seeing what I find when I get there, and that really helps to reduce the number of times I have to go.”  Reference 2 - 1.11% Coverage  “I went to a little shop just because I just felt I needed some control over what I was going to eat, so just a small excursion to a small shop. But we feel the risk benefit of going and doing a long walk out in the open or going somewhere quite remote the benefit massively outweighs the risk for me and my mental health.  Reference 1 - 0.68% Coverage  “The only thing that we have done is we have actually moved in with our parents now, so they isolated for about two weeks and we isolated for a week and then we moved in together, some more support.”  Reference 1 - 0.98% Coverage  “I have had to go without food because I wasn’t put on the risk list I have had long gaps between supermarket deliveries […] and nobody to help me, and that’s meant that I haven’t been able to get fresh food. […] So I have been trying to compensate for that by growing my own food.”  Davis 2021  Reference 1 - 0.29% Coverage  “I was really anxious. I did a calm birth course to try and help me just get some that sense of control back in a world where everything was so uncertain. So, I was just really stressed out . . . I just feel like pregnant women weren’t supported through the pandemic . . . You’re just thrown in the complete deep end because you don’t have regular support that you’d normally have.”  Reference 2  “limiting the access to social media as well, that would be one of the ongoing things to [my] wellbeing.”  Reference 3 - 0.15% Coverage  “Helping wind down and getting to sleep.”  Reference 4  “I’ve made [exercise] more of a lifestyle that I just do every day rather than going to the gym or going to a class which I think the pandemic had made us do that kind of thing a bit more.” (CW14_low)  Reference 5  “Everyone’s been wonderful. We’re just loving life at the moment. Yeah, absolutely. 2020 has been different, it’s had its moments, but overall, it’s been a great year for us.” (CW3_high)  Fumagalli 2021  Reference 1 - 0.31% Coverage  “Yes I have already contacted a psychologist. I’m not afraid of asking for help, I’m afraid of going out from home.”  Reference 2 - 0.23% Coverage  “It reassured me a little bit when they ﬁnally understood that in most cases COVID-19 positive women would not transmit the disease to the fetus.”  Reference 3 - 0.11% Coverage  “I was reassured by the fact that the checks, scans and fetal monitoring were good. My baby was okay. And my symptoms were mild, I’ve never had severe symptoms”  Reference 4  “At the beginning I imagined myself crying from morning to evening during the hospital stay. I actually reacted well instead.” (W10)  Reference 5  “Yes, I am not a very chatty person and I must say I am sharing this experience with everyone. It’s useful to talk about it cause it’s a trauma, we’ve lived some traumatic moments so it’s important to vent somehow.” (W11)  Reference 6  “I’m starting now to talk about it cause until the last few days it’s not been easy [ . . . ]. It’s true it’s all gone now but it’s still difﬁcult.” (W15)  Jackson 2021b  Reference 1 - 0.39% Coverage  “My two best friends and I as well, have been very naughty and met and gone for walks together, because none of us are coping very well mentally…so that has helped tremendously [laughter] erm and I[Voice wavering] I feel terrible er because I obviously am not trying to do the wrong thing but I think the problem with the way the Government’s handled this is they haven’t been very…I don’t know. It just sounds like they haven’t thought it through very well, which I’m sure they have, but I don’t know. It doesn’t feel like it. It feels like they’ve just done all the wrong things [laughter].”  Reference 2 - 0.32% Coverage  “I think that’s what a lot of mums are doing behind closed doors, sort of weighing up…you’ve gotta weigh up risks, a lot of people at this point now. It’s just like erm some people have moved in with their mums.”  Reference 3  “You’re constantly mindful that [grandparents are] still a risk and a danger and you certainly don’t wanna put family members at risk. At the same time, you kinda feel like you need the support, they want to give it and they want to see their new grandchild so it…yeah…it’s difficult to kind of get that balance.” (Participant 20, T2).  Reference 4  “There’s no right or wrong way. You know, at the end of the day the ultimate goal is that my baby needs to be fed. End of. Erm you know, feed him breast milk. Breast milk, er formula. He’s fed. He’s happy. Sweet. That’s done. Job done! You know what I mean? The important thing is actually be kind to yourself, you know?” (Participant 5, T1).  Rice 2021a  Reference 1 - 1.54% Coverage  “I have depression and anxiety, for which I am on Prozac and I have a therapist. And, thankfully, they are both fine and under control, but it was really, really difficult for the first few weeks being home and isolated and being afraid of catching COVID. And knowing that our baby doesn’t have an immune system, we don’t want to jeopardize his health or his life by letting people into our house, so we were in essentially full quarantine, and that was really difficult for me. And I know that a lot of the hormonal swings are very normal, but there wasn’t that support for when you’re crying and exhausted. My mom wasn’t there to hand the baby over to so that I could take a nap. After [my husband] went back to work, it was very difficult, physically and emotionally. And I found that, by week 6, I was crying a lot, the baby was crying, I was crying and exhausted, and really feeling the guilt of feeling like I needed help and not wanting to risk our family. How much struggle to endure for their safety versus knowing that our mental health would be so much better if we let one of our parents into the house? And that wound up being the decision that we made with my therapist because it was the simple solution. I needed my mom”  Reference 2 - 0.44% Coverage  “At some point, you have to look at what’s more important, your mental health or a risk, which [in our community] was so low. ... Are you sacrificing your mental health, which is, in my mind, just as serious as COVID? That’s how we started to feel toward the end. … The mental health thing was a big factor [in our decision to let someone in], for sure.”  Reference 3 - 0.45% Coverage  “I hemmed and hawed and was very stressed over [letting my dad in to help], because I knew it was opening up to a whole other household. My dad was even popping in and out of work then still, so I did know it was a bigger bubble than what we were allowed. But I also knew that I didn’t really have a choice. I just did what worked for my family and me at the time.”  Aydin & Aktas 2021  Reference 1 - 0.29% Coverage  “We couldn't go out, and we never met with anyone. My psychology deteriorated, so I went to our village. The house in the village has a garden. You are sitting among the trees. You talk to your relatives keeping social distance and communicate with people that you think are not sick….”  Reference 2 - 0.22% Coverage  “I tried to cope with it by talking to my baby … I tried to boost myself up, I talked to it, I said “These days will end, and better days will come … you will be born in a beautiful world.” … I relaxed in this way…”  Reference 3 - 0.19% Coverage  “…I read a lot of books, watched movies, played sports, redecorated the house. I knitted something at home, I attended online training programs, I did Plates, and breathe exercises…”  Reference 4  “Well, I was able to overcome it with my husband's support. I mean I dealt with it … We went through this period well as my husband was always with me psychologically, spiritually and financially…” (P10)  Reference 5  “I spent time with my husband … he always supported me … I can say that I coped with the stress and problems brought about by the pandemic thanks to him.” (P2)  Green 2021  Reference 1 - 0.07% Coverage  “On a positive note, I have been able to quarantine with family and so I am set up with a strong support network”  Silverio 2021  Reference 1 - 0.43% Coverage  “Trying to talk to people. Nothing can beat face-to-face in terms of being able to go somewhere and talk to someone face-to-face, someone coming into your house and having a cup of tea or you going in someone’s house and having a cup of tea, but if that is not possible trying to talk to people, talking to people on the phone. When I came oﬀ the phone from particularly my friends and my mum –anyone who had had a baby, who understood what some of these diﬃculties felt or looked like –I always felt reassured.”  Reference 2 - 0.46% Coverage  “The advice my mum kept giving me was: Try and stay relaxed about it. Maternity services are never going to stop. People are going to be giving birth. My mum kept saying to me, ‘Every hospital prioritises pregnant women and maternity services.’ They are never going to compromise on the welfare of you. If they are restricting your visitors, that is only to protect you……… Clearly in my experience maternity services were being prioritised just as much and there was no question of compromising on the level of care. Try and stay relaxed.”  Reference 3 - 0.42% Coverage  “…try to keep on top of how you are feeling about things, because there’s a lot of being in pregnancy, when you feel like everyone else knows what is best for you and you don’t feel that your own feelings, your own views, are heard. And sometimes it is hard to even identify how you actually feel about something. And I guess the other thing I would say is don’t be afraid to –and I think this is easier for some people than others –don’t be afraid to ask for things: advocate for yourself.”  Reference 4 - 0.31% Coverage  “This would be my advice to any mum. Take every day as it comes and ease up on yourself because it is hard with the pandemic and without the pandemic. It is diﬀerent hard with diﬀerent babies, I can tell you now, and it is diﬀerent hard with diﬀerent phases in your relationship with other people, your family and your spouse. Every day is diﬀerent. This is my theory now”  Rhodes 2020  Reference 1 - 0.08% Coverage  “I’m due to go back in November, but I am thinking of taking the full 12 months as I haven’t been able to do stuff with her.”  Charvat 2021  Reference 1 - 0.31% Coverage  “They have all been very emotionally supportive. When I’ve just been upset about things, and “Oh my husband can’t come to my appointment” and they just listen to me complain and . . . even just being there. Just knowing that they love our baby, and they’re excited for us. That just helps, with the resilience, like we were talking about earlier, that also helps fill that resilience of there’s so many people who love us and love our baby. And you know, we will get through this. It will be alright.”  Reference 2 - 0.14% Coverage  “My faith in God . . . gets me through just knowing there’s a bigger picture, a bigger plan at work. That definitely helps me, like I said, put the negative things . . . they’re not so negative when you look at the overall picture.”  Reference 3  “Just having other pregnant friends who were feeling the same things and kind of being affected in some of the same ways, being able to talk to them throughout it all was a huge coping mechanism. ‘Cause like my husband was great support too, but to have someone who is more in your shoes is helpful.”  Reference 4  “be “content in the simplicity of things, with not having extra activities . . . I feel like I’ve just really enjoyed things like going for a walk or reading a book... it’s really helped me be content.”  Reference 5  “Confused. Nothing set in stone . . . Yeah, the information I get is all over the place. And so, I mean, the best I can do is keep myself protected and hope to God that it keeps me protected.”  Sweet 2021  Reference 1 - 0.39% Coverage  “And when you’re not being supported it does amp [amplify] up that anxiety as well, about what’s happening in the world, and how’s it going to be when I go to hospital, can my husband come with me? What am I going to do with the other children?, they’re not allowed to go to their grandparents at the moment… it just changes all the time … we’ll just roll with it and wing it ”  Reference 2 - 0.33% Coverage  Similarly, another participant spoke of her adjustment as a reframing; “we knew that my daughter wouldn’t be able to visit in hospital which is what we had envisioned …but instead we kind of reframed all of that to be like it will just be us and we’ll sort of see how we go from there”  Reference 3 - 0.37% Coverage  “I try to stay oﬄine as much as possible because I noticed that there was a lot of negativity. …I felt other people’s experiences is not the experience or journey that I’m going through …I have had quite a few people say to me ‘are you nervous, are you anxious about this whole pandemic and being pregnant?’, and I’m like ‘no’”  Reference 4 - 0.32% Coverage  “I joined that group [on Facebook] and then found that me and other mums that had other babies were having to advise these ﬁrst time mums, … ‘hey that’s not cool’, like ‘you need to call up the clinic and go get assessed if that’s happening ‘… don’t let them brush you oﬀ” (P14).  Reference 5 - 0.38% Coverage  This participant said, “I felt like I always had to be on top of everything, and if I wasn’t going to be on top of it then things were going to get missed. So, I constantly felt like I had to advocate for me and the baby to make sure that things didn’t go awry …if it’d been my ﬁrst, I could’ve got lost in the system quite easily”  Reference 6 - 0.23% Coverage  “I had to push quite hard to get what was going to work for us and just even to have it seems common sense to me, to have support in place, but apparently that’s optional or not a necessity to them”  Reference 7 - 0.37% Coverage  “we were looking at the numbers and what’s happening in other countries around the world, and we just weren’t sure that there was going to be a hospital to go to …So, in preparation …we bought our own IV ﬂuids and cannulas and respiratory equipment, so that if the baby wasn’t breathing then we could do something about it”  Reference 8 - 0.31% Coverage  “I ended up ﬁnding some old friends that were living in [town] and they came around to the AirBnB and slept the night”  Kyno 2021  Reference 1 - 0.47% Coverage  “I have a little naively trusted that the hospital gives me the information I need. . . Then I realized afterwards that there were many moms who were much angrier than me and said much more; insisted much more. I did not because I thought. . .. . .for those who work there give me what I need. But they do not. If you don’t bother, you must in a way howl and scream to get enough attention, and I simply did not; I regret it a bit”  Sweet 2022  Reference 1 - 0.21% Coverage  “I know enough about the health system that I thought I don’t have to do this, I can say no. And they didn’t make me feel like I was being really awkward saying no, but I think if I didn’t have the knowledge, I had I could quite easily have just gone yeah okay I’ll have it”.  Reference 1 - 0.13% Coverage  “it’s such an evolving landscape you just have to sort of roll with the recommendations and you know if this is what it is this week that’s what we’re doing kind of thing”.  Atmuri 2021  Reference 1  “What happens when you come in? Because obviously there’s not been hospital tours . . . I wouldn’t even know where to go. A video online on the website or something that you can go on and get a tour may be helpful, starting from outside so you know where  you’re going.”  Reference 1 - 0.28% Coverage  “Maybe setting up something like a support group, maybe an online support group . . . for mums that have babies in a pandemic that probably do feel isolated.”  Keating 2021  Reference 1 - 0.36% Coverage  “I think.. one of the advantages to being pregnant.. is that you… feel like there’s an end to it… psychological end… my family and friends are like it’s groundhog day and I’m like no it’s not because I’ve got a new milestone every week or a new thing to look forward to.. so that kind of helps”  Mizrak Sahin & Kabakci 2021  Reference 1 - 0.12% Coverage  “I try to relax myself, I read a book, trying to distract myself with  other things, and then it goes away.”  Reference 2 - 0.17% Coverage  “I started thinking about more positive aspects, I pray, I realized that there were people in need of help, and I felt a little better when I tried to help them.”  Reference 3  “I've been watching it a lot at ﬁrst, frankly, but I've stopped watching the news for a month, I am just looking at the daily number of cases, or check if the Minister of Health makes a statement, otherwise I'm not watching the news.” (P8)  Reference 4  “I'm not watching the news, I get stressed out because everyone says something. I listen only to the statements of the Minister of Health, so I like his explanations, you know, it's promising.” (P14)  Panda 2021  Reference 1 - 0.48% Coverage  “Because it kind of gave me this ﬁnite time where I knew I was going to be going in. …I knew I was having a section. And …going in for at least three days. So I knew …I’m going to have to deal with being in there, after a section, on my own.  Reference 2  “I knew he [my partner] wouldn’t be able to wait around long afterwards. But yea I was prepared for all that. …I knew exactly what was ahead of me. You know for ﬁrst time mums …it must’ve been much harder.”  Reference 3  “And you know if you were kind of just worried, but you were able to talk to each other. And just comfort each other.” (ID 16)  Linden 2021  Reference 1  “But it’s also because we followed the news… And I told my husband all the time too and he also checked… but we discussed the slightest development together. Which was maybe not the best, but at the same time it was necessary for us” (Interview no. 10, 0-para).  Reference 2  “Having diabetes [type 1] and being pregnant has been tougher than handling [the worry about] corona for me, because (…) it is a fulltime job in itself, for the blood sugar to be right … So that made it more like I did not have time to think about much else (…) That quota is filled… and was filled even before corona came…” (Interview no. 4, I-para)  Ollivier 2021  Reference 1  “I had to research and learn myself how to ﬁx my issues - again it has been diﬃcult ﬁnding support but proud that I got breastfeeding ﬁgured out now! All myself too.” (Participant #21).  Reference 2  “I don’t remember trying to look because I ﬁgured it would be pointless & stressful… just didn’t need the added stress.” (Participant #45). | The participants had several pieces of advice for other pregnant and postpartum individuals during a stressful period such as a pandemic. This advice included advocating for oneself and avoiding isolation... Some participants decided to expand their bubble despite the risk of infection to themselves or their parents in order to surround themselves with the support they needed. One participant explained if she had not taken these actions, her mental health would have suffered… Many participants reported positive benefits on their mood and general well-being when they exercised. Some were able to find creative ways to stay active while maintaining social distancing protocols. Their strategies included online personal training, walking outside, using home gym equipment or meeting others at a park for an activity while maintaining social distancing protocols. Participants described breaking pandemic public health directives to access the emotional and physical family support that they needed… Participants developed different coping strategies to manage pandemic stress. Some described how they relied on or avoided information, while others participated in both adaptive and maladaptive behaviours… Participants described adaptive behaviours to help them cope, which included seeking support, exercise, selfcare and finding a way to balance the risks and benefits of visiting with friends and family. (Last three paras are author interpretation only [Kolker 2021]  Decision-making and planning around social distancing  behaviours often involved weighing up relative  risks. Women described difficult decisional processes  such as being unsure whether it was safer to attend the midwife appointment or avoid it, or whether exposure risk outweighed the mental/physical health effects of outdoor exercise. This was compounded by a lack of understanding around the reasons for being in the ‘at risk’ group. Planning arrangements around the birth were fraught as women grappled with how to plan for a parent to help look after existing children during labour or for support with the new-born, or if their partner became ill... Women reported few  instances of breaching the social distancing rules, and  breaches were minimal and carefully considered (e.g.  driving a short distance to take exercise safely; isolation of home and parents’ households prior to moving in together for support). [Anderson 2021]  Many women reported changes in their mental health and wellbeing since the start of the pandemic, in particular increased psychological distress (stress, anxiety, and/or depression). The effects were exacerbated for those with other young children to care for and those who experienced immediate restrictions in support services and access to family care… Online social groups were useful for some women, especially via Facebook, but others  found the discussions overwhelming and had to shut them down… Few participants reported using online wellbeing applications, with the exception of one participant who used a mindfulness app almost every night… Many participants drew positive aspects from their experience during the pandemic period, in both the high and low groups. Women adjusted their lives to the new situation, in particular their exercise routines… For some women, particularly in the high group, the COVID pandemic did not appear to have impacted their experience of having a baby [Davis 2021]    [NB: study about covid positive women] At the time of the interviews, some mothers were in the process of reacting to the negative experience by: a) seeking professional psychological help to elaborate events; b) trying to ‘forget’ the past and build a new peaceful normality; c) desiring redemption from a ‘lost’ experience by having another child in the future… Despite the persisting mass-media info infoemic, after giving birth the women’s attention shifted from the need of receiving up-to date news to the care of the newborn and their maternal role. When the macro-level knowledge about the phenomenon progressively increased and clearer information were provided to the population, some women perceived an overall lower level of uncertainty… The participants were reassured by the fact they were feeling well, both in terms of maternal and neonatal general wellbeing during childbirth (e.g. normal ﬁndings at checks, cardiotocography, scans and tests) and in regard to COVID-19 disease (e.g. being classed as a population not at risk due to age and health history, not having severe symptoms, improvement of symptomatology and recovery)… All women reported to have respected the arrangements and strict rules in place including wearing appropriate PPE, the partner’s absence, isolation during the hospital stay and self-distancing when returning home. Despite these measures often causing feelings of loneliness and sadness, both in and out of hospital the mothers reacted and adapted to the ‘new ordinary’ which was seen as a situation beyond their control. The desire of meeting their baby empowered the women to see themselves as strong and competent mothers… One woman reported that she felt stronger that she thought during the hospital stay… Over the course of time, the women perceived that recalling and sharing events was increasingly helpful in order to elaborate and positively react to the emotionally strong lived experience. [Fumagalli 2021]  Breaking social distancing guidelines was a source of guilt for new mothers who felt conflicted between needing emotional support and wanting to respect social distancing guidelines… All participants talked about rationalising the breaking of social distancing restrictions, within the context of weighing up the perceived costs of limited social interaction with the perceived benefits of receiving much needed support during the immediate postnatal period… Despite increased comfort with breaking social distancing restrictions at T2, moral conflict was still expressed concerning the opposing motivations to protect loved ones from the dangers of COVID-19 and missing receiving support and intimacy… For such women it was deemed essential to stay kind to yourself. [Jackson 2021b]  Interviewees’ narratives suggest that having a newborn at home during the pandemic was challenging for most women (Box 2), and these challenges were compounded by lack of support. This was amplified for those with pre-existing mental health conditions and those recovering from difficult births. For example, the first 2 quotes in Box 2 convey the experience of new motherhood during the pandemic for some women with pre-existing mental health conditions… As illustrated in the second quote in Box 2, we found that many interviewees eventually did allow support persons into the home. This speaks to a third theme that was common across many interviews: seeking out help even where public health policies did not permit it (Box 3). For most interviewees, this decision was accompanied by stress about the risks associated with close contact and was usually done only when the mother had become desperate. [Rice 2021a]  Staying home because of coronavirus negatively affected the psychology of some of the women and changed their social habits or the social environment… Some of the women expressed that they coped with stress by talking to the baby and making preparations for it… The expressions of some of the women who coped with the stress and anxiety caused by the pandemic by strengthening their relationship with their spouse are as follows. [Aydin & Aktas 2021]  Some positive changes to support were also reported, as some women reported that partners and family were around more to help out. [Green 2021]  The ﬁnal theme of this analysis covered women’s advice for other pregnant and birthing women. Largely, advice centred around replacing the face-to-face support with virtual forms of social support, and a noticeable under-reliance on healthcare professionals for this type of support… In addition, there was a ﬁrm belief amongst women that whilst managing COVID-19 was currently the immediate priority of all healthcare services, pregnant women were never going to be forgotten, and, more so, were always privy to prioritisation amongst healthcare services and professionals… And ﬁnally, many women recognised the importance of mental health as well as physical health during pregnancy, childbirth, the postnatal period, and during the COVID-19 pandemic [Silverio 2021]  Some respondents thought a UK-wide extension to maternity leave was needed, while those who were able to extend their maternity leave were considering doing so. [Rhodes 2020]  Those with positive tone narratives acknowledged the critical importance of social support for coping with a pandemic pregnancy. Narratives with a positive tone featured 81.0% of all instances of social support within participant stories. Breaking this down further, emotional support was the most prevalent within positive tone narratives, encompassing 37.1% of social support instances. Instrumental support was the next most prevalent within positive tone narratives at 30.3%, and informational support was the least common, consisting of 13.6% of social support instances in positive tone narratives. Tori gives an example of emotional support in her positively toned narrative… The participants were able to relate to the lives of these other pregnant women in a way that could not be replicated with non-pregnant others, as they were able to understand and commiserate with the complex emotions they had regarding their pregnancies. Their shared experiences greatly improved the quality of support participants received from these friend and pregnancy groups… These storytellers often demonstrated a growth mind-set regarding their pandemic pregnancy, in that they thought something good would emerge from these experiences. For example, Mary was able to use her pregnancy during a pandemic as an opportunity for personal growth… Some women put their faith in God or the universe to make sense of the chaos of the time [Charvat 2021]  Women described their experience as stressful and anxiety provoking. The constant unknowns left them needing to ﬁnd ways to manage these emotions… Over time, this woman found a suitable birthplace with a midwife, but still had to arrange childcare for her toddler when she went into labour…. Descriptions of women’s resourcefulness came mostly in the form of  seeking clariﬁcation, following up test results, or gaining additional appointments. One woman, a farmer living in a rural area, went to the extremes to prepare for her birth… Advocating for self or others The sense of ‘going it alone’ found women feeling the need to advocate for themselves or others. Some participants found this easier to do than others. This sense of advocating for self or others was evidenced through the sub-themes of ‘ﬁnding reliable information’, ‘valuing peer support’, and ‘having to be resourceful’…. Having to be resourceful Many participants spoke of advocating for themselves to achieve the care they desired and/or needed given the constant changes occurring in the health system… Finding a way through The constant changes to the social situation at home, in the work- place, and in health services left women having to make constant adjustments to their maternity experience to accommodate the restrictions, whilst managing their own anxiety and those of others around them. This sense of ‘ﬁnding a way through’ was evidenced through the subthemes of ‘a changed experience for all’, ‘managing stress and anxiety’, ‘requiring constant adjustment’, and ‘managing fear mongering’ (last para is author interpretation only) [Sweet 2021]  Despite questioning the rationale of the regulations, the mothers trusted the nurses and doctor’s medical expertise. However, mothers also believed that health personnel would attend to their need as parents, but discovered that it was "survival of the fittest" as regards… After weeks of hospitalization, a mother met her young son and mother outside the hospital; she felt disobedient to the regulations, but happy…  Parents used different and multiple coping mechanisms to ease their extreme experiences and function in daily life while taking care of their families. Social media was widely used, but bonding with peers/other parents and health personnel became important coping strategies. (last two paras are author interpretation only) [Kyno 2021]  Participants described varied experiences about their decision making throughout their maternity care. Some felt supported and given choice, others felt their options were very limited. P17 said, “it’s such an evolving landscape you just have to sort of roll with the recommendations and you know if this is what it is this week that’s what we’re doing kind of thing”. P4 felt empowered to advocate for herself, [Sweet 2022]    With the cancellation of face-to-face education and hospital tours, women sought guidance on alternate sources of information to prepare for birth. They also requested clariﬁcation on the impact on delivery of postnatal services in the community… Women were solution focused and proposed greater utilisation of the internet medium for online video hospital tours and support groups. [Atmuri 2021]  One woman described the positive milestones in her pregnancy as a welcome distraction from the pandemic… Many women felt that by keeping informed, they would feel empowered but also appreciated that having too much access to news could feel overwhelming. The sources of information ranged from mainstream news and social media to peer-reviewed medical journals. (2^nd^ para is author interpretation only) [Keating 2021]  The majority of pregnant women expressed that they acquired different hobbies at home and preferred to look at the positive aspects of the process in order to cope with the anxiety and worries due to the pandemic process… Some of the pregnant women said they refused to watch the news in order to avoid anxiety pandemic process. [Mizrak Sahin & Kabakci 2021]  Women who were booked for induction of labour or elective caesarean section described this in positive terms, whereby it minimised uncertainty to a degree in oﬀering some control over a situation that was uncontrollable. While arguably women with scheduled induction or planned birth dates outside of a global pandemic may equally experience this sense of control, it appeared heightened for women during COVID-19 as it oﬀered them the opportunity to prepare mentally in advance for being on their own… Preparing for and adapting to the uncertain times caused by the pan- demic emerged in many women’s narratives and stories. Some women described themselves as being ‘ adaptive ’ and ‘ prepared ’ in facing the changing circumstances presented by the global pandemic. Although sad and emotional about going to the hospital without their partner, a few women were mentally prepared for it and had accepted the reality that they were going to be on their own for most of their stay in the hospital… In such narratives, women conveyed a sense of resilience and strong coping abilities amidst uncertainty, although multiparous women described how they were glad it was not their ﬁrst birth while nulliparous women were stoic in saying that they had nothing to compare it to… Being reliant on other women was another way in which women adapted to the circumstances following birth. Women drew comfort and support from each other during their postnatal hospitalisation, emotionally and practically, more than they might have had if the visiting restriction had not been in place. Curtains around individual bedside environments were open and women shared their birth stories and their concerns freely. New friendships were forged between women that continued to the time of interview. A number of women appeared to cope with their own diﬃcult postnatal situation by comparing themselves to others in the room, insomuch as they were not as badly oﬀ as other women who had a sick baby or who had a diﬃcult birth and no support person. [Panda 2021]  Women read new newspaper articles almost daily about how COVID-19 could possibly affect the fetus. Some women disregarded these articles since they believed that maternal health care providers would communicate any relevant information. For these women, constant information about COVID-19 did not affect their pregnancy experience substantially. Other women felt the need to find out more information on their own about COVID-19 in pregnancy and therefore read newspaper articles, government information and other sources that they deemed trustworthy. This caused a lot of stress in these women and had a big impact on their experience of pregnancy. Yet, finding out information on their own was not solely negative to most of them as it also acted as a strategy to regain control in a difficult situation… The shadow from the COVID-19 pandemic was not equally prominent for all women. Pregnant women handled this “new” way of life in different ways. Some became more worried and others acknowledged the presence of the shadow but did not dwell too much upon its existence…. Different women handled uncertainties in different ways. Some women carried on with their everyday life and took the approach that COVID-19 could take a back seat to everything else that they had going on. These women kept busy with work, some had children from before, which allowed them to shift focus. In other cases, there were other concerns that were greater than the fear of contracting COVID-19… The additional unknowns and uncertainties of the COVID-19 pandemic together with the constant reporting in the news increased the women’s worries. Other women acknowledged the pandemic but took the new situation in their stride and did not worry too much. (last para is author interpretation only) [Linden 2021]  Some participants noted difficulty negotiating the balance between limiting the spread of COVID-19 and needing help and rest immediately after giving birth. They expressed feeling torn between wanting family and loved ones to be with them postpartum and wanting both their newborns and older or more vulnerable family members to stay safe. [Dove-Meadows 2020]  One mother was speciﬁcally told that she could not bring her partner to the ﬁrst newborn visit and this caused great stress to the mother as the weather was terrible that day and she had to navigate the car seat, doors, all while holding an umbrella. When she got to the waiting area, she saw that other mothers had partners present. She thought about texting her husband but decided against it because she “felt she had to follow the rules.” (author interpretation only) [Spatz & Froh 2021]  Mental health was recognized when women were forced to not only overcome but learn how to live a new normal and through this growth, they found inner strength and well-being. (author interpretation only) [Saleh 2022]  The complexities of navigating postpartum health information on one’s own was overwhelming for many mothers. Many participants utilized agency to solve their issues and seek out the information they needed... Another participant also believed the amount of information was overwhelming and diﬃcult to navigate; however, she chose not to engage in information-seeking as it was too stressful. Agency in knowing what they needed (or didn’t) in relation to health information was apparent, though enacted in diﬀerent ways by diﬀerent parents. [Ollivier 2021] | **[Being more conscious of their mental health]** Many women were conscious of the need to maintain and acknowledge the impact of the pandemic on their mental health. Women described the different actions they took to protect their mental health as well as recommendations for others, such as reinforcing the need to avoid isolation, seek the support and engage with family, friends and peers where possible, doing a calm course, avoiding the news and social media due to the abundance of misinformation and negativity experienced online, and finally seeking professional help when required. Women were also conscious of the link between their physical and mental health, ensuring they were able to take time to go outdoors, or exercise. (Kolker 2021, Anderson 2021, Davis 2021, Fumagalli 2021, Rice 2021a, Aydin & Aktas 2021, Silverio 2021, Charvat 2021, Sweet 2021, Mizrak Sahin & Kabakci 2021, Ollivier 2021, Keating 2021, Linden 2021)  **[Self-advocacy is key in a pandemic]** Women that reflected on their pregnancy and postpartum experience shared that self-advocacy was something that they had either done, or wished they had done more of to ensure that they were able to receive care in a way that was safe and responsive manner to their needs. Women encouraged others to ask questions, speak out and seek resources to better enable them to advocate for themselves. Some women realised that differences in care occurred when others “spoke louder”, feeling regret that they did not advocate for themselves more strongly. Some women were conscious about the lack of preparation and education they received about the birthing experience in the hospital, for first time mothers, they desired more videos and information to indicate what they should be expecting and reduce stress. (Kolker 2021, Silverio 2021, Sweet 2021, Kyno 2021, Sweet 2022, Panda 2021, Ollivier 2021, Spatz & Froh 2021)  **[Breaking public health restrictions– weighing up the risks]** The social distancing rules impacted some women more significantly than others. Whilst this was a sensitive time to be adhering to public health measures, some women heavily debated the risk of infection and transmission and the deterioration of their mental health. This was reflected in some women’s responses as they deliberated whether or not they should access family and friends for additional support and break social distancing rules. Many women made the decision for themselves and their family unit to allow extended family into their homes or entered family member’s homes to allow for additional support. Some of these women took extra measures to ensure there were quarantine periods, to minimise risk of infection. Ultimately this was the best decision for women’s mental and physical health and wellbeing. (Kolker 2021, Anderson 2021, Jackson 2021b, Rice 2021a, Sweet 2021, Kyno 2021)  **[Coping strategies]** Women were resilient throughout the pandemic, overcoming challenges and encouraging positive thoughts to make it through the difficult time. The coping strategies that women adopted ranged from an individual internal response to an interpersonal and community level response, accessing support from family and friends and the wider community. On an intrapersonal level, women reframed their mindset to the pandemic, choosing to ignore the severity of covid and its impact, “just rolling with it”, connecting online with others, relying on their faith, and choosing to focus on their pregnancy, the newborn baby and spending more time to bond as a family unit. Many chose to take the day as it came, and not to dwell too much on the stressors of the pandemic. Other women accessed reliable sources of information to manage their stress levels, e.g. regarding transmission from mother to fetus or baby, as well as seeking information to be well-prepared for their birth plan, but also be and ready for the worst in the event of drastic COVID measures. Women also relied on their partners and family to cope with the challenges. One woman decided to grow her own food to cope with the stressors of the pandemic, and food shortages. At the interpersonal level, some women relocated out of urban areas to manage the stressors of the pandemic. (Kolker 2021, Anderson 2021, Davis 2021, Fumagalli 2021, Jackson 2021b, Aydin & Aktas 2021, Green 2021, Silverio 2021, Rhodes 2020, Charvat 2021, Sweet 2021, Sweet 2022, Atmuri 2021, Keating 2021, Mizrak Sahin & Kabakci 2021, Panda 2021, Linden 2021, Kyno 2021) |
| 6.2. Making the most of the positive situations | | |
| Anderson 2021  Reference 1 - 0.84% Coverage  “I think maybe there’s some nice time that we have had with our toddler and with my partner which we wouldn’t normally have had this much time together before the baby comes, so there’s definitely trying to see some nice things with it.”  Reference 2 - 0.29% Coverage  “Now everyone has got their masks on and it’s in and out as soon as possible.”  Davis 2021  Reference 1 - 0.14% Coverage  “It’s quite nice being less sociable . . . It’s quite nice not having all these different appointments booked in to see people. I think it just made everyone slow down a little bit.”  Reference 2 - 0.25% Coverage  “Everyone’s been wonderful. We’re just loving life at the moment. Yeah, absolutely. 2020 has been different, it’s had its moments, but overall, it’s been a great year for us.”  Reference 3 - 0.12% Coverage  “I know a lot of us saw the beneﬁts of a reduction in visitors . . . We were able to use coronavirus as an excuse just to stay in our little bubble.”  Reference 4 - 0.23% Coverage  “In fact, if anything I didn’t feel any pressure to go out and see people, I could just relax at home . . . I suppose the one good thing about not having everyone come over and visit was having that time to adjust to having a baby without lots of visitors and lots of expectations to host for people.”  Reference 5  “Working from home is quite a lot better and it deﬁnitely made my pregnancy a lot easier working from home.” (CW11_low)  Reference 6  “It’s been really great that my husband had been able to work from home a lot throughout the year . . . So, if that’s one thing that we could keep, would be him to continue to work from home.” (CW3_high)  Rhodes 2020  Reference 1 - 0.04% Coverage  “Not having to go out the house as much has made me more relaxed.”  Reference 2 - 0.07% Coverage  “My baby is having the best start to life! Full undivided access to her mummy and her daddy!! For 2 months!”  Reference 3 - 0.12% Coverage  “No pressure to go to groups, no need to go places at specific times, no stress of public feeding or changing nappies in public toilets. He has 100% of my attention and is very happy and settled.”  Panda 2021  Reference 1 - 0.58% Coverage  “We were well distanced in the waiting area. …A lot of things went a lot quicker, the wait times and stuﬀ… you were in and out a lot quicker whereas in a normal day it would be quite packed and you’d be, you could be waiting there a while.”  Reference 2 - 0.48% Coverage  “It was lovely, we had our own little bubble, me and my husband and my baby; because my husband was oﬀ from work as well. So… it was quite calm and relaxed. Because that was kind of the atmosphere we wanted.”  Reference 3  “…the whole experience with not having …gangs of … visitors coming in and out …it was just so pleasant. ” (ID 01)  Reference 4  “… it was an absolute blessing in disguise really with COVID. Because you couldn’t have people in your house. People weren’t rocking up, arriving and you’d to like feed them, or make tea and coﬀees and all that.” (ID 03)  Reference 5  “I was able to breastfeed without feeling really exposed and thinking about it now I think if there had been people around, strangers marching in and out of the ward I would have had the curtains pulled all the time and I wouldn’t have put my foot outside you know.” (ID 13)  Riley 2021  Reference 1 - 0.24% Coverage  “We are not standing around queuing for half-an-hour, to an hour after your scan like you are in and out”.  Reference 2 - 0.27% Coverage  “Stopped loads of people coming to the house and staying for a ridiculous amount of time and having to clean the house”.  Reference 3 - 0.33% Coverage  “PPE, cleaning, everything like that, so yes I don’t think it concerned me that I would come out with anything, you know, the virus or anything ”  Reference 4  “Thanks to lockdown my partner is here 24-7…even though he is working…we actually had the beauty of sharing both of our love with the baby, so if I am tired I can take a nap and he can take care of the baby for half-an-hour” (P16, Age 33, PP 17 weeks)  Jackson 2021b  Reference 1 - 0.60% Coverage  “I guess it’s one of the things like my husband was saying as more of a positive to come up is, because we haven’t had any visitors and any pressure on going to see anybody or people coming round, I have just been able to kind of get on with it…he was saying especially with breastfed babies, they normally lose weight but [our baby’s] just been gaining weight ever since she’s been born. I think that’s ‘cause we’ve just been able to just get on with the feeding, especially some days when she’s been like constantly  wanting to feed. I haven’t had to worry about people being ‘round or any of that.”  Reference 2 - 0.26% Coverage  “We can literally adapt our lives around the baby’s schedule. So erm without worrying about ‘Oh I’m getting up for work, I haven’t got enough sleep. I’m not going to be able to actually function at work with like only two hours sleep’ so yeah, don’t have to worry about that.” (Participant 5, T1).  Reference 3 - 0.30% Coverage  “It seems to be the same like… everyday, but it’s like it’s good ‘cause like if it was just me on my own like I don’t think I’d manage to get in to a routine. But with husband’s like we’ve gotta stick to it, ‘cause it just keeps you, you know what’s coming-my eldest’s better when he’s got a routine.”  Reference 4 - 0.38% Coverage  “I could just recover on my own [laughter] just recover in the house. Didn’t have to change out my pyjamas, I didn’t have to have the house clean…and have to put up a front and to see guests. So, I had that time really, alone.”  Reference 5 - 0.21% Coverage  “It’s enabled us to instil a routine with her. So, she’s a great sleeper now and a happy baby and we because you don’t get that stream of visitors.”  Reference 6 - 0.27% Coverage  “It was lovely to-when we came home from the hospital; it was just me, my husband, my little girl and the baby. It was just the 4 of us, and it gave [eldest] chance to get used to the baby before other people started coming round, kinda thing.”  Reference 7  “I think even…especially from a breastfeeding point of view it’s kind of like I don’t feel like I have to rush. You know I- there’s nothing to rush for. So, it’s kind of like she’s…she’s gaining weight well.” (Participant 1, T1).  Reference 8  “One good thing about lockdown is that had all the time in the world to be able to breastfeed, get it right and erm yeah because I wasn’t doing anything else.” (Participant 24, T2).  Joy 2020  Reference 1 - 0.33% Coverage  “It’s been great . . . we have this opportunity to bond as a family and he [partner] is here for every moment during the newborn stage! It has been amazing not having to worry about visitors coming and going and cleaning out home and me worrying about breastfeeding in front of others - instead we have a very relaxed atmosphere for everything!”  Reference 2 - 0.43% Coverage  COVID has been a blessing and a nightmare for our new family . . . [our baby] gets a lot more daddy time every day and I get help during the day if I need it . . . I now feel no pressure to be a “super mom”. I just focus on spending time with her [baby] and enjoying her baby days. I know if the pandemic hadn’t happened, I’d be out doing “mommy and me” classes, doing visits, and generally trying to be more productive.  Reference 3 - 0.19% Coverage  “We were able to spend so much time alone as a family, and constantly being with our new baby has made us able to learn so much about her and enjoy spending our time with her without any distractions.”  Reference 4  “my husband is home constantly which is positive and negative”. The blessing of partners included more support and help with the daily activities of their homes, including cooking and cleaning, as well as baby duties, such as changing diapers and holding.  Reference 5  “he is available to help with diaper changes and to hold the baby when I need a quick break, but it isn’t what I pictured for my maternity leave”.  Keating 2021  Reference 1 - 0.12% Coverage  “We brought him home and my plan was to… lock the door, stay in… We definitely benefited from that.”  Reference 2 - 0.17% Coverage  “…not being overwhelmed with visitors. I’m quite glad that we can kind of have an excuse to say to people ‘actually, not at the moment’ “  Kyno 2021  Reference 1 - 0.23% Coverage  “. . . then I think the child benefits from more peace and quiet. As these infection control measures entailed.”  Reference 2 - 0.24% Coverage  "At NICU, we also got a peace then, that is, a peace to be with the child, only with the child when I was there with the time we had"  Reference 3 - 0.33% Coverage  ” . . . of course, we understood the principles of reducing the number of people in the unit, we were at least as concerned as the staff. . ..”  Rice 2021a  Reference 1 - 0.39% Coverage  “Looking back, it was nice to not have any visitors and just be with the baby. I think that was something that a lot of people didn’t really consider when they found out that they weren’t allowed to have any visitors. I don’t think people really realized the benefit of just having that alone time with the baby.”  Sweet 2022  Reference 1 - 0.10% Coverage  “The good part about no visitors in the hospital was that we got lots of lovely time with baby, it was really quiet and uninterrupted”  Reference 2 - 0.17% Coverage  “It was nice that there wasn’t that sort of hustle and bustle of people constantly coming in and out of the hospital … and it was nice that I could say nobody’s allowed to come, and it wasn’t just me being [nasty]”.  Reference 3 - 0.22% Coverage  “I felt very safe, it didn’t really cross my mind because you know the checks at the door were so stringent, like there was hardly anybody around, the hospital, it didn’t feel like a hospital, the corridors were empty, so I didn’t feel like I was crossing paths with a lot of people”.  Reference 4  “My partner’s Mum was insistent on actually being in the delivery room and I was a bit like ‘yeah no! I don’t think I want you to see me in that state’, I’d rather just be with my partner. So, I mean it’s not too bad for me really” P26.  Aydin & Aktas 2021  Reference 1 - 0.16% Coverage  “I had time for myself and my baby … I'm sure I would be more stressed if I were working. Now I can walk more, eat healthier … These are positive effects of the pandemic for me, and I can say that it facilitated my adaptation to pregnancy….”  Reference 1 - 0.23% Coverage  “We could spend time together … We had breakfast together. Before the pandemic, I used to leave home early so we couldn't have breakfast together, but we are all together in this period, so it affected me positively.”  Costa 2021  Reference 1 - 0.25% Coverage  “In some respects it was good, because you don’t feel like everyone’s dropping in all the time and you and can really get to know the baby, and get to know their routine and what they’re like.”  Reference 2  “We certainly felt supported. I can’t thank (the CNS) enough. She’s always been at the end of the phone and she’s always communicated things […]. She didn’t always know what was happening either, and she was completely honest about that.”  Reference 3  Sandra also stated, “I really don’t feel like I have been abandoned or anything […]. I deﬁnitely feel (the CL/P team) has been (on top of it).”  Sweet 2021  Reference 1 - 0.20% Coverage  “At the same time it was also really nice to have a reason not to leave the house and to be able to say we’re just going to stay home and have this quiet time as a family ”  Reference 2 - 0.24% Coverage  Similarly, “we just stayed in our little bubble, and we all slept when he [baby] slept, and we watched movies and cuddled …and –we probably wouldn’t have done all that …the pandemic made that part a lot nicer”  Reference 3 - 0.21% Coverage  “As I entered the hospital all the safety procedures were followed, temperature check, confirmation that I hadn’t been in touch with anyone from overseas or COVID infected, I didn’t have an issue”. The safety measures were also evident in private consulting rooms.  Reference 4 - 0.09% Coverage  “Social distancing, they spaced out their appointments, so you never saw another patient whilst I was there”.  Reference 5  “it was good to have COVID as an excuse not to have people over … because of the restrictions it’s meant that I haven’t had to politely decline people coming over, and everyone seeing the baby and wanting to cuddle her and all of that, I didn’t have to deal with any of that ”(P6) .  Atmuri 2021  Reference 1 - 0.34% Coverage  “It’s been really good, in that, I got to work from home instead of going into the ofﬁce, so I’ve been able to, yeah, take a rest when I need a rest and pick my own hours.”  Reference 2 - 0.35% Coverage  “They’re very careful with everything. Like asking where have I been . . . and checking the temperatures. Even if I go to the ultrasound, they always have a social distance . . . It’s actually pretty good. They are taking extra care for their patients. They’re just making everyone safe . . . you know, so this Coronavirus won’t spread. Which is understandable actually.  Brown & Shenker 2021  Reference 1 - 0.21% Coverage  “I'm due back at work next week but I do not actually know what this will entail although I am unlikely to have to go in. This has meant an ease in the anxiety of continuing to breastfeed as I will still be at home after maternity leave has ended so I do not need to worry about childcare provider and expressing for bottles etc.”  Reference 2  “I've found breastfeeding quite difficult due to problems with latching, nipple tears/bleeding and lots of pain. Being able to stay home and concentrate on getting the feeding right is the only reason I persisted. If I'd had lots of visitors or pressure to meet other mums/family/friends, I'm not sure I would have managed as I've only just got the hang of feeding 5 weeks in!”  Reference 3  “I was inundated with visitors with my first child and often could not feed responsively due to their discomfort with feeding or them wanting to comfort my daughter when she was upset. She had poor growth and I felt enormous pressure from my in laws in particular to supplement with formula. With my second child, there is none of that pressure and I can really see an enormous difference both is his feeding and in my mental health.”  Reference 4  “Not being able to go out has allowed me to gain more confidence in bf. I still do not feel confident enough to feed in public and feel I need support with positioning to be able to do this. Not having lots of visitors also has allowed me to be able to feed how it works for us without having to worry about people coming round.”  Reference 5  “During lock down I have had more time to focus on feeding my baby on demand and not feel rushed because I need to be anywhere.”  Fumagilli 2021  Reference 1  “I was calm cause we have a small garden and my daughter could go out and have fun [ . . . ] it was like a holiday at the beginning.” (W16)  Saleh 2022  Reference 1  “We had a lot more time to just hang out altogether.”  Spatz & Froh 2021  Reference 1  They reported that having to be home was a ‘plus for breastfeeding’. Stella stated ‘you can just be at home and only in a bra all the time’”. | Psychological capability includes emotional ability to  adhere to social distancing. While some women reported positive lockdown experiences (e.g. family time at home), many reported feeling isolated, low, and suffering loss of joy, which impacted on their perceived ability to sustain the behaviour. This was particularly acute when living alone and fully shielding. [Anderson 2021]  Women in both the high and low groups expressed the beneﬁts of less socialising and uninterrupted time to bond with their baby. Enforced social isolation positively contributed to several women’s general feelings of wellbeing… For some women, particularly in the high group, the COVID pandemic did not appear to have impacted their experience of having a baby… Women in both the high and low groups expressed the beneﬁts of less socialising and uninterrupted time to bond with their baby. Enforced social isolation positively contributed to several women’s general feelings of wellbeing… Increased work ﬂexibility was viewed as a positive aspect of the situation, both for  women who had the opportunity to work from home during pregnancy and, especially, for their partners. [Davis 2021]  A small minority of respondents painted a more positive picture of their state of mind, finding their home-based life relaxing and less stressful than “normal” life… Here, experiences were divisive; many respondents with a partner working from home really appreciated the additional time spent together. Postnatal respondents pointed out the practical benefits of sharing childcare and household tasks as well as the emotional benefits of the greater opportunity to bond as a family. [Rhodes 2020]  The infrastructural and process changes described by women included eﬃcient COVID-19 screening at the hospital entrance, use of personal protective equipment (PPE) by healthcare professionals, quiet waiting areas, exposure to limited numbers of healthcare professionals and short consultations. Many women positively referred to these changes and how they were implemented and were reassured by them… Following discharge home from the hospital a few women described their feelings of ‘ bonding’ with the baby in their ‘ perfect little bubble ’ with other siblings and their partner. Many of these narratives centred on guests not arriving unexpectedly as might have occurred had COVID restrictions not been in place… many drew comfort from the peace and quietness offered by less crowded postnatal wards. The visiting restrictions while in the hospital were valued by many women and were described as ‘ pleasant ’ because they provided women with the time and space to ‘ bond ’ with their baby, without any distraction or disruption from visitors… ‘ blessing in disguise’ for women whereby the task of entertaining visitors, as one woman described it, was removed… In particular, women who were breastfeeding narrated feeling more relaxed, comfortable and less exposed as a result of the visiting restrictions in the hospital. [Panda 2021]  Reassurance that the baby was doing well and the opportunity to spontaneously seek support was perceived to be important for ﬁrst-time mothers. Yet, when the few face-to-face appointments were attended, participants commented on the speed of the service. The eﬃciency of face-to-face care was considered “a plus point ” (Participant 7, Age 33, PP 4 weeks) as it reduced the degree of contact with others and the risk of infection… Participants appreciated the additional time they were able to spend as a family and suggested the restrictions… Participants felt safe in the hospital setting and suggested hospital staﬀ were doing everything they could to manage the risk of infection… Partners who worked from home during lockdown were able to provide a degree of respite for participants [Riley 2021]  Despite lockdown restrictions exacerbating feelings of distress, isolation, and loss of self-identity, eight of the women interviewed also spoke of positive outcomes having arisen from lockdown restrictions. Lockdown restrictions allowed for a more relaxed postnatal period, without experiencing pressures from social obligations… Participants spoke of appreciating having the opportunity to settle into their new parenting roles more mindfully… Additionally, many women revelled in receiving additional parenting support from their partners which allowed for the establishment of a routine… Despite frustrations and setbacks caused by COVID-19 restrictions, all interviewees expressed that there had been some positive outcomes which had arisen from imposed restrictions. Many interviewees talked about feeling less pressure from visitors and having the unique opportunity to be more mindful and to relax into their new parenting roles… Additionally, mothers appreciated having the opportunity to establish a clear and responsive routine with their infants, without disruption from visitors… For some participants, restrictions on visitation during the initial lockdown allowed mothers to response-feed their infants easier, and thus breastfeeding was easier to establish… Breastfeeding practice was reportedly easier for some mothers due to reduced visitations, allowing women to more easily persevere and response-feed their babies… Positive outcomes which were derived from lockdown restrictions concerned having fewer visitors in the early postnatal period, which allowed mothers and maternal partners to be more present and mindful in adapting to new parenting roles, allowed greater paternal involvement in the family unit, and made breastfeeding easier to establish. (last para is author interpretation only) [Jackson 2021b]  One such blessing was participants’ freedom to enjoy their babies. A common experience for many  mothers was the feeling of freedom from social expectations, including fulﬁlling the dominant socially constructed roles and identities set before many new moms, as illustrated in the following quote… Similar to the participants above, many of the participants in this study, found the period during the COVID-19 pandemic to be a time for quiet enjoyment of their baby and their new family as they were required to stay at home. Using the lens of feminist poststructuralism, we can see how these mothers were challenging social norms and expectations of new mothers. Challenging social ideals was a way of using their agency as they clearly articulated their beliefs about how being at home with their babies was very positive and for some a ‘blessing’. Many participants believed that this was a time for more personal and intimate family enjoyment. A time where the immediate family could not only enjoy each other but also their new baby… The COVID-19 pandemic not only provided an opportunity for intimate family bonding and freedom from the social pressures of new parenthood, but it also provided time for many participants to learn. Their learning was focused on their new baby as well as learning about themselves as new parents. For example, one participant believed that they [parents] “had this time to support each other and see each other grow as parents”. This time was valued as a time in which they could come to understand their new identities as parents - a time for her and her partner to grow together in these new roles and identities. Other participants also noted similar experiences, as illustrated in the quote below… Experiences with partners were often viewed by many participants as both blessings and curses… The blessing of partners included more support and help with the daily activities of their homes, including cooking and cleaning, as well as baby duties, such as changing diapers and holding. [Joy 2020]  The lockdown has some unexpected benefits. It gave  many women the opportunity to work from home which made it easier to manage normal pregnancy symptoms such as fatigue or nausea and gave more time to devote to exercise, rest, or spending with their partners. This was also valuable after the birth as many women felt this extra time allowed for bonding and there was no pressure to see people or bring their new baby out. Many women’s partners continued working from home after the birth which allowed for additional support in the “fourth trimester.” Many women appreciated quieter waiting rooms and benefitted from more community-based care including early transfer home teams. One woman described the positive milestones in her pregnancy as a welcome distraction from the pandemic. [Keating 2021]  They described an overall social quietness, peace, and ability to solely focus on the infant without welcoming visitors, such as eager family members or friends... They reported that due to a generally increased compliance with isolation and infection control, there was no need to argue why one could not receive visitors… In the beginning, when social distancing was generally implemented in the society, the parents’ acceptance and understanding for the strict access policy due to the COVID-19 regulation was… The hospital appeared as a safe zone from a more dangerous unknown outside world. [Kyno 2021]  Reference to a positive postpartum experience in hospital for this quote… A few participants felt that the pandemic had little impact on their hospital stay. These women were generally second- or third-time mothers without pre-existing mental health conditions whose pregnancies and births had been free of medical complications. Although some were affected by policies that required their birth partners to leave the hospital shortly after delivery, these women needed limited in-hospital support postpartum and welcomed the opportunity for undisturbed rest with their newborns (last para is author interpretation only) [Rice 2021a]  For some women however, these visitor restrictions had unexpected advantages… many participants noticed and appreciated measures to reduce risk of exposure… For a few, the imposed restrictions were advantageous, for example. [Sweet 2022]  Some pregnant women noted that the COVID- 19 pandemic affected their adaptation to pregnancy positively and some negatively. Being at home during the pandemic allowed some of the working women to rest, eat regularly and spare time for their babies and themselves, and thus affected their adaptation to pregnancy positively. They expressed their feelings as follows… Half of the women, most of whom were employed, highlighted that the COVID- 19 pandemic had a positive effect on their spousal relationships, while the other half stated just the opposite. The statements of those whose spousal relations were positively affected revealed that they had the opportunity to spend more time with their husbands. [Aydin & Aktas 2021]  Nonetheless, some participants were able to ﬁnd positives during this period of isolation… Most participants reported positive experiences of the support they had received from the CL/P teams, in spite of the pandemic [Costa 2021]  There were some positive outcomes of the restrictions… [Sweet 2021]  Some women expressed that there had been unexpected positive beneﬁts arising from the restrictions due to ﬂexible workplace arrangements and the opportunity to physically rest at home. [Atmuri 2021]  For mothers with a slightly older baby, a number found that their return to work coincided with the lockdown. This meant that some were furloughed or were working from home, which meant that they did not have to put their baby in childcare. This translated into more contact with their baby, more feeds and less need to express, which meant some babies got more breast milk instead of formula. It also helped mothers feel more relaxed… Many participants talked about a slower pace of life and nowhere to go or need to be having a positive impact on how much time they could spend focusing on feeding their baby. This was particularly helpful for some mothers who were struggling with issues such as latch. They believed that if they had been pressured to be out and about meeting people, they would still have been struggling or in pain and would have stopped breastfeeding… For some mothers, fewer visitors meant that they were more relaxed and had more time to focus on their baby and their own recovery rather than hosting a stream of people wanting to see their baby. For others, it meant fewer unwanted comments. This helped get breastfeeding off to a much better start… Related closely to the previous factor was the enhanced privacy mothers had. A common reason for stopping breastfeeding sooner than planned is embarrassment about feeding in front of others. Mothers who felt this way reported feeling more confident being at home and not having to feed out and about, feeling they would not have breastfed so long in public or in front of visitors. Some sat around topless, having lots of skin-to-skin contact. This enabled them to practice and gain confidence in latching their baby on, discretely, if desired… Another common experience was that the additional time and lack of pressure meant that it was easier to feed responsively, that is, responding to infant cues of hunger and satiety rather than following a mother led routine (WHO, 2003). Mothers reported feeding babies more often, to less of a routine because they did not need to plan around things like school runs and spotting earlier feeding cues. This impacted positively on perceptions of milk supply and played out in increased early weight gain. [Brown & Shenker 2021]  The reaction to isolation and social distancing rules was described by some women as calm and peaceful, taking uncertainties day by day towards a new ‘family balance’ and adaptation to the ‘new ordinary’. Having a garden was seen as a facilitator, especially in case of other children. [Fumagalli 2021]  Mental health was tied to proximity of support from others either physical (bringing meals) or emotional. Many women recognized that COVID-19 policies allowed them to spend a greater amount of time one-on-one with their neonate and families than typical maternity leave allowed due to changes in work expectations (virtual work and social distancing). Cherished time with families and finding a new norm allowed for development of healthy coping mechanisms and reframing of the negatives of COVID-19 by some mothers. [Saleh 2022]  Silver Linings  Despite challenges that the mothers experienced, there were a few positives. A great beneﬁt to all the mothers was having their partners at home with them due to social distancing. All had partners who were working from home due to the pandemic. They reported that this was positive because the partners got to see and experience all the ﬁrsts [Spatz & Froh 2021]  Moreover, three participants (all second or third-time mothers who had smooth and uncomplicated vaginal births) welcomed the uninterrupted time with their newborns that hospital visitor restrictions enabled, but otherwise, the impact of the pandemic on participants’ experiences of pregnancy, childbirth, and early postpartum was overwhelmingly negative. (author interpretation only) [Rice 2021b] | **[Spending more time to bond as a family unit]** Postpartum women reflected on the increased opportunity to bond with the newborn. Public health measures reduced the pressures on mothers to receive visits from family and friends, or to be more social with their newborn. Partner presence was also positively reflected upon as working from home arrangements allowed them to be more inclusively involved with the newborn, and to provide support for mothers when required. However, for some mothers the increased interactions put a strain on relationships and was described as difficult from a practical perspective after baby arrived. (Anderson 2021, Davis 2021, Rhodes 2020, Panda 2021, Jackson 2021b, Joy 2020, Keating 2021, Rice 2021a, Sweet 2022, Aydin & Aktas 2021, Costa 2021, Sweet 2021, Green 2021, Saleh 2022)  **[Fewer disruptions to routine]** Due to public health restrictions on visitation on postnatal wards, isolation and lockdowns, many women described fewer disruptions to their routine. On the postnatal ward, there were fewer visitors allowing mothers to rest and recover, bond with their baby and establish breastfeeding without having to be concerned and conscious of people visiting. In the early postnatal period, the lack of visitors was also positively experienced, as mothers described this period as relaxing, calm, and quiet, which was beneficial to the family unit as it allowed more opportunities to bond, breastfeed the baby on demand, and prevent any social obligations that mothers would have participated in pre-COVID. This slower and calmer time allowed mothers to settle into their parenting roles, respond to their babies actively and put less strain on mothers to be ready for guests during the early postnatal period. Multiparous mothers welcomed the “newfound quiet time” and benefited from reduced social requirements post-birth. (Davis 2021, Rhodes 2020, Panda 2021, Riley 2021, Jackson 2021b, Joy 2020, Keating 2021, Kyno 2021, Rice 2021a, Sweet 2022, Costa 2021, Sweet 2021, Atmuri 2021, Brown 2021, Fumagalli 2021, Spatz & Froh 2021)  **[Peaceful health services]** Some women described the different atmosphere in healthcare services during the pandemic, with increased social distancing rules, more PPE requirements and fewer people in hospitals, creating a safe and protective atmosphere for women attending appointments and for labour and childbirth. Women attending appointments were seen more promptly, limiting the time needed to be spent in waiting rooms, and long queues. Women also received compassionate care from healthcare providers, receiving adequate information, and reassurance that they were going to be okay. Giving birth with their chosen support person (if they were permitted) and fewer visitors to the postnatal ward added to the sense of calmness women described. (Anderson 2021, Panda 2021, Riley 2021, Jackson 2021b, Kyno 2021, Sweet 2022, Sweet 2021, Atmuri 2021, Costa 2021) |
| 6.3. *Information seeking and desire for more information* | | |
| Atmuri 2021  Reference 1 - 0.27% Coverage  “You do feel a little bit stressed in there, and I think probably one thing that maybe could be improved is just that extra information of what you are doing with the COVID stuff in terms of precautions, what it’s going to look like when I come in to have bubs, just what to expect.”  Reference 2 - 0.49% Coverage  “What happens when you come in? Because obviously there’s not been hospital tours . . . I wouldn’t even know where to go. A video online on the website or something that you can go on and get a tour may be helpful, starting from outside so you know where  you’re going.”  Costa 2021  Reference 1 - 0.30% Coverage  “I browsed the CLAPA website. It gives a lot of information, it’s packed with knowledge in terms of what to expect and […] there is also a family community where you can reach out.”  Reference 2 - 0.24% Coverage  “We have been using the […] online coffee chats that (CLAPA) do […]. I’ve listened to the diagnosis ones and the feeding ones and the surgery one. […] When I’ve listened to other people’s experiences it helps me […] think ‘OK, yeah, they’ve dealt with it, that’s what happens’.”  John 2021  Reference 1 - 0.10% Coverage  “But it was mainly up to me as well to read and ask questions… rather than getting, um, like information.”  Kolker 2021  Reference 1 - 0.17% Coverage  “So paying more attention to the news, it was kind of like a distracter … I think that I’d rather know as much as I can.” [P9]  Reference 2 - 0.18% Coverage  “What helped me is I do my own research. I read a lot. Sometimes it is good. Sometimes it is not good ... Yeah, because what I do is I panic, because, yeah, I seek information. I like reading from the reliable sources. That helps me a lot”  Reference 3  “It would be great if they had a better idea of what your stay is going to look like, so you’re a bit more mentally prepared for what’s to come.” [P10]  Reference 4  “there was a lot of confusion … try to be as clear to the patients as you can.”  Linden 2021  Reference 1 - 1.01% Coverage  “But it’s also because we followed the news… And I told my husband all the time too and he also checked… but we discussed the slightest development together. Which was maybe not the best, but at the same time it was necessary for us”  Silverio 2021  Reference 1 - 0.26% Coverage  “…it’s just a bit more on you that it maybe would have been otherwise, because all these clinics are not running and the regular appointments are not happening, or at least not face-to-face. Maybe that’s what I would say, is that the support is there and really good, but I think generally it is on you to enquire”  Sweet 2021  Reference 1 - 0.13% Coverage  “Just really on the news, which maybe isn’t the best source but just looking at what was coming up on the news ”  Reference 2 - 0.12% Coverage  “Watching the live updates from the premier …I followed the government’s Facebook website updates daily”.  Reference 3 - 0.11% Coverage  “I tend to sort of self-research a lot and I’d read the WHO [World Health Organization] guidelines”.  Reference 4 - 0.27% Coverage  “The college of obstetrics and gynaecology, I’ve kept sort of up to date with their guidelines ” (P8). Similarly, “I looked at the academic analysis that had been done on these six cases in Wuhan and I looked at things like [RANZCOG] ”  Reference 6 - 0.17% Coverage  “There was a Facebook group that I was on that did a live interview with an obstetric professor answering questions about COVID which was really helpful”.  Reference 7 - 0.28% Coverage  “Because you just can’t trust them –like you’ve got to decipher through what’s true and what’s not …Is that actually having a positive inﬂuence on me, and my mental and physical health, or not? And if it’s a no well why am I engaging in this?”  Reference 8  “I think my obstetrician –she has like a social media page, she would publish [from] the college of obstetricians, she would post their latest guidance, because she has lots of anxious people” (P4).  Riley 2021  Reference 1 - 0.18% Coverage  “I consider my husband and I to have enough about us to do the research and things like that, but it has been so hard, it has just put so much extra pressure on”  Mizrak Sahin & Kabakci 2021  Reference 1  “. . . but there's probably a lot of research going on right now, so there's no full information.”  Reference 2  ‘Not enough. But I also understand why it's not enough. We are just in the beginning of the process; there cannot be more statistical research.’ (P4)  Reference 3  “I learned it by myself; I don't think it's enough as we need more information.’ (P1) ‘… no, I learned this information by myself as I couldn't go to the hospital due to this disease, and I didn't get any information.”  Charvat 2021  Reference 1  “[The video] said you could have three people [in the birthing room], then two people at a time, and no . . . I literally get one person. So, it’s just that kind of [that] tangible information, nothing really related to health.”  Reference 2  “I was very appreciative to have my peer pregnancy group. Thankfully, [we] had set up, like, a text chain right before everything shut down and a Facebook group. And so [I] was able to communicate with them still throughout everything . . . and having them as a resource was super helpful. We were, you know, always updating people whenever we heard new information. That was really great, just because of course there was new information coming out all the time and having kind of that filter of like, okay, this is actually really relevant for our situation.”  Davis 2021  Reference 1  “[The information] . . . it’s just too complicated for a normal person to understand it. We’re not medics, we’re not in that ﬁeld, so you’re just like, “What exactly are you trying to say?” (CW4_low)  Rhodes 2020  Reference 1  “Without groups to go to I’ve found having these apps to be even more useful and helpful.” [Free text]  Reference 2  “I have enjoyed using Baby Buddy throughout pregnancy and these early months and find it even more reassuring now that external input is so limited.” [Baby 7 weeks, age 30-34 years, White British]  Reference 3  “I look forward to the little bit of advice every day. It is written in such a non-judgemental way, encouraging but not patronising. I feel like it’s giving me some of the general knowledge I’d be picking up from other mums at baby groups.” [Free text]  Reference 4  “Because it’s linked to the NHS it’s not like googling.” [Baby 3 months, age 30-34 years, Black, Asian, minority ethnicity  Reference 5  “Things to do with him when you are stuck inside – keeping him entertained, what’s best for his development.” [Baby 5 months, age 25-29 years, White British]  Ollivier 2021  Reference 1  “I had to research and learn myself how to ﬁx my issues - again it has been diﬃcult ﬁnding support but proud that I got breastfeeding ﬁgured out now! All myself too.” (Participant #21).  Reference 2  “I have become more concerned about the implications for children contacting the virus and the potential long term impacts it may have based on more recent research.” (Participant #61).  Reference 3  “I also understand the importance of social distancing and just want to do what’s right to keep my family healthy.”  Anderson 2021  Reference 1  “I am very careful about reading stuff on reputable sources sort of thing”  Reference 2  “From what I have seen on the news or the NHS guidelines it has been advised that pregnant women should social distance for 12 weeks, self-isolate, so I live with mum and dad and so we’ve not really been going out at all”  Reference 3  “I don’t know, even though I am pregnant and I am classed as high risk category I don’t feel like I am a risk, and I don’t feel scared about it. I don’t know, maybe I should be more, but I think if it was really explained a bit more that no actually you have… there’s a very high risk you could have a miscarriage if you got it then I would be really… I don’t really understand why. So I think if I understood it a bit more I might maybe take it a bit more seriously.”  Jackson 2021a  Reference 1  “I didn’t have any contact details for health visitors. I didn’t feel like I could go to them, either. Just felt like I was being intrusive. So I just used Google all the time, which is good and bad [laughter] because there’s a lot of diagnosis things on there that might not be useful.” (Respondent 22, T2).  Snyder & Worlton 2021  Reference 1  ‘‘Um honestly I look up a lot on Pinterest just for articles. I know that’s probably not the most reliable’’ (27, Caucasian, Church Director). | Many women desired ofﬁcial communication from the hospital about the various uncertainties of the pandemic, such as the risk of acquiring the infection and what precautions were being taken to reduce this risk… With the cancellation of face-to-face education and hospital tours, women sought guidance on alternate sources of information to prepare for birth. They also requested clariﬁcation on the impact on delivery of postnatal services in the community… Women were solution focused and proposed greater utilisation of the internet medium for online video hospital tours and support groups. [Atmuri 2021]  Participants also reported receiving remote support from community-based organisations, such as CLAPA, which appeared to be a source of information and advice [Costa 2021]  Although all participants reported some level of information provision from healthcare providers regarding clinical decision making, almost everyone agreed that they would benefit from more thorough discussions. Most participants received information about their pregnancy in the form of signposting to books or websites but they expressed that their individual information needs would have been better met by one- to- one discussions (box 2: Informed decision making: P6). Women who felt that their questions remained unanswered did not feel involved in shared decision making (box 2: Informed decision making: P3). [John 2021]  Some participants described themselves as information seekers… Strategies for Coping with Pandemic Stress - Approach to Pandemic Information… With the ever-changing recommendations and suggested precautions during a pandemic, participants felt there should be clear communication and up to date information coming from their HCPs. Specifically, they suggested clear messaging regarding hospital policies and what care would look like during their hospital stay… Moreover, participants wanted the health care team to have consistent messaging regarding in person versus virtual visit timing. [Kolker 2021]  Women read new newspaper articles almost daily about how COVID-19 could possibly affect the fetus. Some women disregarded these articles since they believed that maternal health care providers would communicate any relevant information. For these women, constant information about COVID-19 did not affect their pregnancy experience substantially. Other women felt the need to find out more information on their own about COVID-19 in pregnancy and therefore read newspaper articles, government information and other sources that they deemed trustworthy. This caused a lot of stress in these women and had a big impact on their experience of pregnancy. Yet, finding out information on their own was not solely negative to most of them as it also acted as a strategy to regain control in a difficult situation. [Linden 2021]  Disruption to In-Person Care and Increased Virtual Care Provision [Silverio 2021]  All participants spoke of the many resources they accessed to try to ﬁnd reliable information about the COVID-19 virus, protecting themselves and their families, accessing health care services, keeping up to date with the government restrictions, and ﬁnding sources of support. The mainstream media was mentioned… Some participants sought information from professional websites… Another participant used a medical college website for information… Some participants were told about RANZCOGs website by their doctor. Some groups were supported by health professionals… Some participants were sceptical of the accuracy of social media information and its impact on their wellbeing… Whilst most participants spoke of seeking information about the pandemic and its possible impact, some participants spoke of more general information needs about pregnancy and birth, as they felt these were not adequately covered in their telehealth appointments… Many participants accessed information on social media, most commonly Facebook. [Sweet 2021]  This uncertainty and lack of information was still an issue postpartum. Participants talked about how they “weren’t given any information on which [health] service(s) [they] should contact and for what period of time they would support” (P14, Age 32, PP 15 weeks) following the birth of their child. This was diﬃcult for participants… The ‘fear of the unknown’ from a lack of information and subsequent uncertainty around birth appeared to be worse than the actual experience itself, for those that had given birth… Whilst information from non-medical sources was perceived to be their only way to keep on top of the latest developments and caring for their baby, it led to increased fear, anxiety and concern for many participants. (last two paras are author interpretation only) [Riley 2021]  Some of the pregnant women who were not able to reach sufﬁcient sources of information on this topic argued that this was normal as it was a new virus… The most important sources of information of pregnant women during this process were internet and television. Speciﬁcally, pregnant women who could not attend controls for pregnancy monitoring were receiving information via internet and television when they could not reach their doctor, midwife and nurse. Pregnant women who had access to information through their own learning thought that their knowledge was not enough. [Mizrak Sahin & Kabakci 2021]  Tina discussed being unsure that the information in the hospital tour video was accurate and updated… The participants not only had to contend with changes in their health due to their pregnancy, but also with the rapidly shifting medical and social landscape surrounding the COVID-19 virus. Thus, informational support provided clarity and helped these pregnant women make important health decisions. For example, Isabella described feeling grateful for her support group that helped her screen virus-related news and learn information that was most applicable to her as a pregnant woman [Charvat 2021]  Information needs to be communicated that is easy to understand [Davis 2021]  A lack of clear guidelines from the government led some respondents to feel that they were a forgotten sector of the population. Although initial guidelines had suggested that pregnant women should shield, there was confusion as to whether this meant staying indoors or being allowed out to exercise and whether it was still applicable (by May) or had been superseded by new advice. Postnatal respondents were not aware of any advice directed at them as parents of young babies, and many were critical of its absence…Several respondents identified additional information and advice needs as a consequence of the pandemic. This included how to ensure your baby is gaining the right amount of weight and meeting developmental milestones (in the absence of health visitors); ideas for stimulating and entertaining babies (in the absence of classes); ways to calm a crying or fractious baby; ways to reduce one’s own anxiety and stay calm; and making sure you are getting enough vitamin D with limited time outdoors… telephone interviewees frequently remarked that the main strength of the app was the provision of trustworthy and easy-to-understand information. Most interview respondents had either been recommended Baby Buddy by their midwife or had seen a poster or leaflet for the app at an antenatal appointment. Therefore, they felt confident that the app met with NHS approval and that its content would be accurate and trustworthy. [Rhodes 2020]  The complexities of navigating postpartum health information on one’s own was overwhelming for many mothers. Many participants utilized agency to solve their issues and seek out the information they needed… Some mothers speciﬁcally spoke about their fears of COVID-19. They generally sought information from sources they deemed reliable, such as the provincial public health oﬃce. That being said, others still felt confused or misinformed about how to care for their baby during the pandemic… Due to the lack of information and evolving evidence about how COVID-19 was spread, how it presented in young children and infants, and how it might be treated, parents prioritized keeping their family safe [Ollivier 2021]  Women reported making efforts to access “credible” and “reputable” sources for information including government advice, BBC (British Broadcasting Corporation) news, Royal College of Obstetricians and Gynaecologists (RCOG)/ midwives, NHS, pregnancy apps/emails (e.g. Bounty), Tommy’s (online), select social media groups, contacts, scientific sources, and newspapers… Women wanted to know more information about their Covid-19 related risk during pregnancy (e.g. why were they in the vulnerable category, what relative risk was associated with each trimester, what is the evidence), their personal risk factors (e.g. comorbidities, ethnicity), clarity on aspects of the guidelines and clarity on changes to their maternity care. Women wanted written information from credible sources, with clear messages presented in an accessible format. Some women reported a desire for lots of detail, while some were experiencing information overload. [Anderson 2021]  Respondents who felt ill supported and burdensome about help- seeking were left to find information themselves from internet sources. For such women, concerns were raised about fears of potential exposure to misinformation… COVID-19 related restrictions on access to healthcare professional support led to T2 respondents feeling burdensome and abandoned. Extra reliance was thus placed on sourcing information from the internet. For women who did source information from online sources, virtual healthcare was perceived as being an insufficient replacement for essential face-to-face healthcare appointments in that virtual care failed to meet maternal and infant wellbeing needs (last para is author interpretation only) [Jackson 2021a]  When asked about where they received their information, many relied on HCPs, websites, and social media. However, many noted they would have preferred a central repository of up-to-date information, so they were not forced to vet the bombardment of information, and make major decisions that impacted their health alone (author interpretation only) [Saleh 2022]  Many women felt that by keeping informed, they would feel empowered but also appreciated that having too much access to news could feel overwhelming. The sources of information ranged from mainstream news and social media to peer-reviewed medical journals. (author interpretation only) [Keating 2021]  When asked where they obtained informational support the most common responses focused on social media (i.e., Pinterest and Facebook) followed by lactation counsellors. Related to social media use one mother noted, [Snyder & Worlton 2021] | **[Desire for more information]** Many women felt empowered and described themselves as information-seekers, as they sought official communication and information from the hospitals and their healthcare providers about the impact of the virus on pregnancy and their unborn babies, hospital protocol and regulations, and how public health measures would affect them. They were also described as solutions-focused and sought their own information and clarity on areas of concern or uncertainty. There was increased desire for accurate and up to date information that did not include medical jargon and would be understood by all. Women felt that the onus was placed on themselves to obtain up to date and additional information as they felt that they would not be told otherwise. Some described the increased pressure in obtaining accurate and reliable information, making this process a lot harder than expected. The ability to obtain and comprehend the information was seen as both positive and negative; the desire to be informed, but not overwhelmed was also another balance that many women had to manage. Some women expressed that there could have been more information and clear advice for expecting mothers to navigate health settings, where to get additional support and what to expect as COVID precautions changed. (Atmuri 2021, John 2021, Kolker 2021, Linden 2021, Silverio 2021, Sweet 2021, Mizrak Sahin & Kabakci 2021, Charvat 2021, Davis 2021, Rhodes 2020, Ollivier 2021, Anderson 2021, Riley 2021, Saleh 2022, Keating 2021)  **[Utilising online and public resources]** Many women resorted to using different online and public resources to keep themselves updated and informed. Whilst some relied on the media coverage of the pandemic, others used social media, online platforms, organisation run websites, reading from reliable sources, professional colleges and platforms run by well known and respected academics. Additionally, women received information from informal sources such as mother’s groups that were formed pre-COVID. This helped women stay informed, however, some acknowledged that some information was not useful and that the burden of over-information, was sometimes detrimental to their mental health wellbeing. (Costa 2021, Linden 2021, Sweet 2021, Charvat 2021, Davis 2021, Rhodes 2020, Jackson 2021a, Riley 2021, Saleh 2022, Snyder & Worlton 2021) |
